# *Panstrongylus geniculatus* and four other species of triatomine bug involved in the *Trypanosoma cruzi* enzootic cycle: high risk factors for Chagas’ disease transmission in the Metropolitan District of Caracas, Venezuela

**DOI:** 10.1186/s13071-014-0602-7

**Published:** 2014-12-23

**Authors:** Hernán J Carrasco, Maikell Segovia, Juan C Londoño, Jaire Ortegoza, Marlenes Rodríguez, Clara E Martínez

**Affiliations:** Laboratorio de Biología Molecular de Protozoarios, Sección de Epidemiología Molecular, Instituto de Medicina Tropical, Facultad de Medicina, Universidad Central de Venezuela, Caracas, Venezuela

**Keywords:** Chagas’ disease, *Trypanosoma cruzi*, Triatomine bugs, Risk factors, Caracas, Venezuela

## Abstract

**Background:**

Chagas' disease is caused by the protozoan *Trypanosoma cruzi* and is autochthonous to the Americas. Its distribution depends on triatomine bugs that are responsible for the transmission of the disease. In 2005, we reported the presence of *Panstrongylus geniculatus* as a risk for Chagas' disease transmission in Caracas and neighboring areas. Three massive oral outbreaks occurred in the following years. Here we report the results of a 7-year study on triatomine species found in the Metropolitan District of Caracas (MDC), Venezuela.

**Methods:**

Triatomine species collected by inhabitants of Caracas during 7 years were analyzed for parasite infection and blood meal. Triatomines were found in 31 of the 32 parishes surveyed. Traitomines were examined for the presence of blood and parasites in the digestive tract. Molecular techniques were used for the typing of parasites.

**Results:**

A total of 3551 triatomines were captured from 31 of the 32 parishes surveyed. The vast majority of these were identified as *P. geniculatus* (98.96%), followed by *Triatoma nigromaculata* (0.59%), *Triatoma maculata* (0.39%) and *Rhodnius prolixus* (0.06%). Triatomines were always most abundant between April and June, and 2010 showed the highest number. We found that 54% of the specimens were females, 42.5% males and 3.5% nymphs. Overall, 75.2% of the insects were naturally infected with *T. cruzi* and 48.7% had fed on blood. Analysis of the adult forms showed that 60% of the females and 31.9 % of the males had blood in their stomachs, and 77.5% of the females and 73.3% of the males were naturally infected with *T. cruzi*. Nearly all, 99.6% of the *T. cruzi* isolates analyzed belonged to the TcI genotype.

**Conclusions:**

Blood-fed triatomine bugs infected with *T. cruzi* were distributed throughout Caracas. Four different species of triatomines were identified of which *P. geniculatus* was by far the most predominant. Our previous report of *Eratyrus mucronatus* raises the number of triatomine species in the MDC to 5. Dramatic modifications to the surrounding natural habitats have led to the establishment of a *T. cruzi* urban enzootic cycle, resulting in a high risk for Chagas' disease transmission in this capital city.

**Electronic supplementary material:**

The online version of this article (doi:10.1186/s13071-014-0602-7) contains supplementary material, which is available to authorized users.

## Background

American Trypanosomiasis or Chagas’ disease is caused by the protozoan *Trypanosoma cruzi.* This species is autochthonous to the Americas and has a distribution from the southern United States to northern Argentina and Chile, overlapping that of the insect vectors that transmit the disease. The blood sucking insects responsible for the vectorial transmission of Chagas’ disease belong to the order Hemiptera, family Reduviidae, subfamily Triatominae and comprise a total of 136 species divided into 18 genera and 6 tribes [1,2]. Only a few genera are involved in human transmission of the parasite, however, with the widely distributed *Triatoma*, *Panstrongylus* and *Rhodnius* being the most important from an epidemiological point of view. Other Chagas’ disease transmission routes are from infected mothers to their newborns (congenital), from infected organ transplant and blood transfusion donors, and the ingestion of food or drink contaminated with feces of triatomine bugs or the blood or raw meat of infected mammals [3]. A total of 73 mammalian genera belonging to the orders Didelphidomorphia, Lagomorpha, Chiroptera, Rodentia, Pilosa, Cingulata, Carnivora, Primata and Perisodactyla that share a habitat with the triatomine vectors have been found to be naturally infected with *T. cruzi* [4]. Overall, 22 species of triatomine bugs in the genera *Alberprosenia* Martínez & Carcaballo (1sp), *Belminus* Stål (2spp), *Cavernicola* Barber (1sp), *Eratyrus* Stål (2spp), *Microtriatoma* Prosen & Martínez (1sp), *Panstrongylus* Berg (4spp), *Psammolestes* Bergroth (1sp), *Rhodnius* Stål (6spp) and *Triatoma* Laporte (4spp) have been identified from Venezuela [5]. The principal triatomine species implicated in Chagas’ disease transmission cycles is *Rhodnius prolixus*. This species shows a high capacity to invade and reproduce in human dwellings that have conditions that favor its colonization, i.e. houses made from mud walls and thatched roofs: the classic type of rural housing in Venezuela. In Caracas, population growth and urban development that has encroached into the surrounding forest have facilitated the interaction of human settlements with wild transmission cycles of the parasite involving essentially sylvatic triatomine species such as *Panstrogylus geniculatus* [6]. Several authors have suggested that this species could be involved in the human transmission of Chagas’ disease [7-11]. In a previous study undertaken in Caracas and neighboring regions, we reported a high percentage of *P. geniculatus* infected with *T. cruzi* as well as the presence of human blood in the digestive tract of the insect, indicating that it could constitute an important risk factor for Chagas’ disease transmission in this north-central area of Venezuela [12]. Unfortunately, in 2007 there was an outbreak of orally transmitted Chagas’ disease in a middle-class primary school in Caracas; this was followed two years later in 2009 by a second outbreak of oral transmission in a primary school in Vargas state and a third in 2010 at a secondary school in a poor neighborhood in Caracas [13-15]. In order to identify the primary source of parasites that contaminated the food in the last two of these outbreaks (2009 and 2010), we identified the parasite populations of infected patients. These were then shown to be the same as the parasite populations obtained from *P. geniculatus* and animal reservoirs cohabiting in the areas where each outbreak took place. This analysis was performed on 246 *T. cruzi* isolates obtained from humans, triatomine bugs and reservoirs using 23 microsatellite markers [16]. For the present study we analyzed triatomines collected and brought to the Institute of Tropical Medicine by inhabitants of Caracas over a period of 7 years. The insects came from all over the city from very wealthy to very poor neighborhoods. The citywide infestation of infected triatomine bugs and the presence of blood in their digestive tracts confirm the establishment of an enzootic *Trypanosoma cruzi* cycle in an urban ecotope, and suggest that there is a high risk of Chagas’ disease transmission in the Capital District of Venezuela. These results have important epidemiological implications and demonstrate the need to develop and implement surveillance and prevention campaigns for the control of Chagas’ disease in Caracas, issues which require urgent attention from the health authorities.

## Methods

### Distribution of triatomines in Caracas

**Figure 1:**
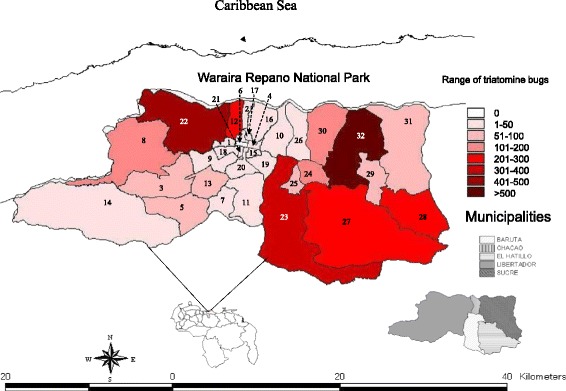
**Map of Caracas showing the distribution of triatomines among the 32 parishes surveyed.**

**Figure 2:**
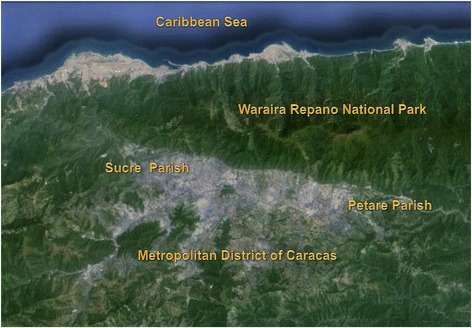
**Satellite map of Caracas.** Google Earth. 2014.

A total of 3551 triatomine bugs were caught and brought to the out-patients unit at the Institute of Tropical Medicine (IMT), Universidad Central de Venezuela (UCV), by inhabitants of Caracas between 2007 and 2013 (Table 1, Additional file 1: Table S1). Although some information was not formally recorded, most people reported finding insects indoors including inside bedrooms, and in many cases had been bitten by triatomines. This study only included the examination of triatomine bugs from the Metropolitan District of Caracas (MDC). The MCD supports a total population of 2,904,376 inhabitants and covers an area of 810 km^2^ (Figure 1, Additional file 2: Table S2) [17]. It is comprised of 32 parishes that make up the Libertador, Chacao, Baruta, Sucre and El Hatillo municipalities (the last four of which form part of Miranda State). Caracas lies 800 m above sea level, and has an average annual temperature of 18-22°C and mean annual rainfall 870 mm [18]. The urban areas are surrounded by pre-montane forest (Figure 2) [19].

**Table 1:** **Species of triatomine bugs found in the metropolitan district of caracas between 2007 and 2013**

**Species**	**Sex/stage**	**2007**	**2008**	**2009**	**2010**	**2011**	**2012**	**2013**	**Total**
*P. geniculatus*	Female	44	220	340	611	264	197	222	1898
	Male	27	164	263	616	172	136	114	1492
	NIII	1	2	10	8	1	4	1	27
	NIV	5	11	19	12	4	9	11	71
	NV	1	1	10	5	4	5	0	26
	Sub-total	78	398	642	1252	445	351	348	3514
*T. maculata*	Female	0	0	1	4	1	1	1	8
	Male	0	1	0	1	2	1	1	6
	Sub-total	0	1	1	5	3	2	2	14
*T. nigromaculata*	Female	0	1	3	7	0	2	0	13
	Male	0	0	0	7	1	0	0	8
	Sub-total	0	1	3	14	1	2	0	21
*R. prolixus*	Male	0	0	2	0	0	0	0	2
	Sub-total	0	0	2	0	0	0	0	2
Total number of triatomines		78	400	648	1271	449	355	350	3551
Triatomines analyzed for blood ingestion		78	394	564	1169	386	303	324	3218
Triatomines analyzed for *T. cruzi* infection		74	394	463	979	355	271	265	2801

NIII to NV: nymphal stages III to V.

### Identification of the triatomines and *T. cruzi* genotyping

Each of the triatomines collected was identified to species following Lent and Wygodzinsky [1]. Specimens were then further examined according to the conditions in which the insects had been preserved by the collectors. The stomach content and intestines of insects in good condition were examined for the presence of blood and parasites, respectively. Samples of stomach and intestinal contents were diluted in isotonic saline solution (0.85%), smeared onto slides, stained with Giemsa and then examined under a microscope. Characteristic morphological forms of *T. cruzi* were identified as described by Hoare [4]. When parasites were observed, triatomine feces were inoculated intraperitoneally into balb/c mice to obtain *T. cruzi* isolates as reported by Carrasco *et al*. [12]. Parasites obtained from mice infected with triatomine feces were initially grown in biphasic blood-agar medium and then transferred to RPMI liquid medium for further expansion. An aliquot was preserved in liquid nitrogen and the remaining culture was used for the extraction of parasite genomic DNA. *T. cruzi* genotyping was done by applying the RAPD technique using primers A1, A2, L4 and L5 as described by Carrasco et al. [20]. In our experience this technique has been shown to be reliable, reproducible and very accurate for classifying Venezuelan *T. cruzi* isolates into one of the six genotypes or discrete typing units (DTU) currently recognized [21,22]. The RAPD technique was applied to classify 277 *T. cruzi* isolates obtained from five different species of triatomines found in 26 of the parishes located in the five municipalities of the Metropolitan District of Caracas. The identification of the *T. cruzi* genotypes was confirmed by using the PCR-RFLP technique [20,22].

### Statistical analysis

The relationship between the sex of the triatomines and the presence of blood in the stomach content or parasites in the gut was estimated using a Chi Square analysis. We also used this analysis to test whether the presence of blood was associated with the insect developmental stage (adult or nymph). All analyses were done with a test significance level p = 0.05 and critical *X*^*2*^ value = 3.8415.

## Results

### Geographical distribution of triatomine bugs in Caracas

**Figure 3:**
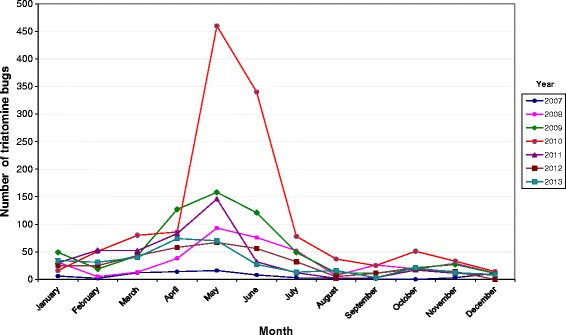
**Monthly distributions of triatomine bugs found in the Metropolitan District of Caracas, 2007–2013.**

Of the 3551 triatomines captured by the inhabitants of Caracas, 3514 were taxonomically identified as *Panstrongylus geniculatus* (98.96%), 21 as *Triatoma nigromaculata* (0.59%), 14 as *Triatoma maculata* (0.39%) and 2 as *Rhodnius prolixus* (0.06%) (Table 1, Additional file 1: Table S1). Of the adult specimens collected, 56% were female and 44% male. The geographical distribution of the triatomine bugs across the city during the study period of 7 years is shown in Table 2 and Figure 1. It is worth noting that *P. geniculatus* was found in 31 of the 32 parishes surveyed, showing that this species is widely distributed and highly predominant in Caracas. The highest numbers of *P. geniculatus* were collected in Petare parish, located to the northeast of the city (Figures 1 and 2, Table 2). This was followed by Sucre parish (Figure 1, Table 2), located to the northwest (Figures 1 and 2). A more detailed examination of the monthly triatomine distribution patterns over the study period revealed that the highest numbers of triatomines were collected between April and June of any given year with May being the month of peak collection (Figure 3, Additional file 3: Table S3). The highest number of triatomines collected during these months (by far) was in 2010 when 460 specimens were captured in May followed by 340 specimens in June (Figure 3, Additional file 3: Table S3). Moreover, 2010 was also the year in which the highest cumulative number of *P. geniculatus* was recorded with specimens collected in 25/32 (78.13%) parishes (Table 2). The highest numbers of *T. nigromaculata* (N = 14), a strictly sylvatic species, and *T. maculata* (N = 5), a mainly sylvatic but occasionally peridomestic species, were also reported for 2010 (Tables 1 and 2). In 2009, 642 *P. geniculatus*, 1 *T. nigromaculata*, 3 *T. maculata* and 2 *R. prolixus* were collected and identified; this last species commonly associated with rural areas and the presence of palm trees. *R. prolixus* had been considered to have been eliminated years ago from Caracas due to the extensive urban development and dramatic changes in the environmental conditions within the city, creating an unfavorable habitat. However, we found one *R. prolixus* individual inside a house located in a poor, densely populated area on a hill in Caracas (Antimano parish). The specimen was blood-fed and infected with *T. cruzi*. This neighborhood is located near to the mountains of the Waraira Repano National Park, former known as El Avila. The second *R. prolixus* individual was found in a middle-class district (Chacao parish), characterized with having many trees planted along roads leading to the Waraira Repano National Park .

**Table 2:** **Distribution of the different species of triatomine bugs in the 32 parishes surveyed in Caracas**

Number*	**Parish**	**2007**	**2008**	**2009**	**2010**	**2011**	**2012**	**2013**	**Total of species**	**Total**
1	23 de Enero	0	1	0	3	4	0	0	8	8
2	Altagracia	4	5, (1)	7	15	1	2	2	36,(1)	37
3	Antímano	1	2	43,(6); 1^c^	13,(1)	7	9	6	81,(7); 1^c^	89
4	La Candelaria	1	0	0	0	3	1	0	5	5
5	Caricuao	1	6	8	34	5	5	6,(1)	65,(1)	66
6	Catedral	0	0	0	0	0	0	0	0	0
7	Coche	0	2	6	7; 1^a^	1	0	1	17; 1^a^	18
8	El Junquito	8	12, (1)	16,(1); 1^a^	78,(1), 4^b^	30	11	21; 1^a^	176,(3); 2^a^; 4^b^	185
9	El Paraiso	0	0	3	11	5	0	1	20	20
10	El Recreo	0	4	7	17	6	6	5	45	45
11	El Valle	2	0	1	5	6	0	0	14	14
12	La Pastora	10	32 (1)	29,(1)	100,(4)	39	17	14	241, (6)	247
13	La Vega	1	6	4	21; 2^a^	12	9,(2)	7	60,(2); 2^a^	64
14	Macarao	0	2	2	6		1	0	11	11
15	San Agustin	0	0		10	1	1	1	13	13
16	San Bernardino	0	3	7	3	8	1	2	24	24
17	San José	1	2	14	17	4	3	2	43	43
18	San juan	0	0	2	0	0	2	0	4	4
19	San Pedro	1	9	2	14; 1^a^	1	4	2	33; 1^a^	34
20	Santa Rosalia	0	0	1	6	0	0	2	9	9
21	Santa Teresa	0	2	3	0	0	0	1	6	6
22	Sucre	2 (4)	47, (3); 1^a^	97,(9)	130,(9)	35,(1)	50,(5)	51	412,(31); 1^a^	444
23	Ns. Sra. R. Baruta	4 (1)	47; 1^b^	70,(2); 1^b^	119	47	35,(1); 1^a^	40	362,(4); 1^a^; 2^b^	369
24	El Cafetal	2	9	28,(1)	50	23,(2)	10	16	138,(3)	141
25	Las Min. de Baruta	1 (1)	5 (2)	10,(1)	24, (1)	14,(1)	15	11,(1); 1^a^	80,(7);1^a^	88
26	Chacao	0	7	2; 1^c^	8	6	2 ,(1)	2,(1)	27,(2); 1^c^	30
27	Sta R. P. El Hatillo	4	27,(1)	38(1)	81,(1); 1^a^; 5^b^	19,(1); 1^a^; 1^b^	17,(1); 1^a^; 2^b^	12	198,(5); 3^a^; 8^b^	214
28	Filas de Mariche	8	17,(2)	35,(4)	73,(2); 2^b^	20,(1); 2^a^	30,(2)	14,(3)	197,(14); 2^a^;2^b^	215
29	La Dolorita	6	3	12	24,(1)	5	4,(2)	14	68,(3)	71
31	Caucagüita	1	5	21; 1^b^	30	7	4	5	73; 1^b^	74
30	Leoncio Martínez	3	17	14	50	27	16	20	147	147
32	Petare	10 (1)	112,(3)	121,(13); 1^b^	278,(5); 3^b^	100,(3)	78,(4)	78,(6)	777,(35); 4^b^	816
	Sub-total	71 (7)	384,(14); 1^a^; 1^b^	603,(39); 1^a^; 3^b^: 2^c^	1227,(25); 5^a^; 14^b^	436,(9);3^a^; 1^b^	333,(18); 2^a^; 2^b^	336,(12); 2^a^	3390,(124); 14^a^; 21^b^; 2^c^	3551
	Total	78	400	648	1271	449	355	350	3551	

Numbers correspond to adult triatomines except those in brackets ( ) = nymphs of *P. geniculatus* from stages III to V. Single number = *P. geniculatus*. Superindex: a = *T. maculata*, b = *T. nigromaculata*, c = *R. prolixus*. Parishes 1–22 lie within the Libertador Municipality, 23–25 in the Baruta Municipality, 26 in the Chacao Municipality, 27 in the El Hatillo Municipality, and 28–32 in the Sucre Municipality.

*The numbers of the parishes (1 – 32) are used to identify their location in Figure 1.

### The presence of blood in the stomachs of the triatomine bugs

**Figure 4:**
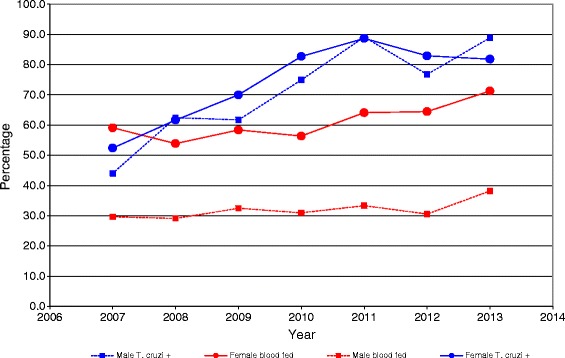
**Annual percentages of triatomine bugs found infected with** ***T. cruzi*** **or blood-fed.**

As shown in Table 1, from a total of 3551 triatomine bugs received by the IMT (Institute of Tropical Medicine) only 3218 were examined for the presence of blood. Of the 3113 adults captured, 1759 were females (56.5%) and 1354 males (43.5%), and of these 1056 females (60.0%) and 430 males (31.9%) were blood-fed (Table 3). Thus, according to the Chi Square analysis, there was an association between the sex of the triatomines and the presence of blood in their stomachs (χ^2^ = 245.19; p = < 0.0001) with females more likely to have had a blood meal than males (Table 3). The percentage of blood in the stomach was also markedly higher in females than in males throughout the entire seven years of the study (Figure 4). This remained relatively constant every year until 2010 for both sexes, when it started to increase gradually until the end of the study in 2013, with this increase being more notable in females.

**Table 3:** **Presence of blood in the digestive tracts of the species of triatomines found in Caracas**

		**2007**		**2008**		**2009**		**2010**		**2011**		**2012**		**2013**		**Sub-total**	
**Species**	**Sex/stage**	**No blood**	**Blood**	**No blood**	**Blood**	**No blood**	**Blood**	**No blood**	**Blood**	**No blood**	**Blood**	**No blood**	**Blood**	**No blood**	**Blood**	**No blood**	**Blood**
*P. geniculatus*	Female	18	26	100	118	125	176	246	320	83	150	62	116	57	144	691	1050
	Male	19	8	116	48	151	72	392	173	97	47	74	33	67	42	916	423
	NIII	0	1	2	0	3	5	1	6	0	1	0	3	0	1	6	17
	NIV	0	5	5	2	3	15	3	7	0	1	2	7	2	9	15	46
	NV	0	1	0	1	3	5	0	5	0	3	0	3	0	0	3	18
	Sub-total	37	41	223	169	285	273	642	511	180	202	138	162	126	196	1631	1554
*T. maculata*	Female	0	0	0	0	0	1	3	0	1	0	1	0	1	0	6	1
	Male	0	0	1	0	0	0	1	0	1	1	1	0	1	0	5	1
	Sub-total	0	0	1	0	0	1	4	0	2	1	2	0	2	0	11	2
*T. nigromaculata*	Female	0	0	1	0	2	1	2	4	0	0	1	0	0	0	6	5
	Male	0	0	0	0	0	0	2	4	0	1	0	0	0	0	2	5
	Sub-total	0	0	1	0	2	1	4	8	0	1	1	0	0	0	8	10
*R. prolixus*	Male	0	0	0	0	1	1	0	0	0	0	0	0	0	0	1	1
	Sub-total	0	0	0	0	1	1	0	0	0	0	0	0	0	0	1	1
Total number of triatomines		37	41	225	169	288	276	650	519	182	204	141	162	128	196	1651	1567

### Natural infection of triatomine bugs with *T. cruzi*

Examination of the gut for parasites was only possible in 2801 triatomines as the remaining 750 insects were either in a state of internal decomposition or extremely dry. Table 4 shows that, of these, 2107 (75.2%) were infected with *T. cruzi* and 1106 (52.5%) were blood-fed (Table 5). Separating the data for females and males we found that 1169 (77.5%) of the 1509 females and 880 (73.3%) of the 1200 males examined were infected with *T. cruzi*, (Table 4). Thus there was also an association between triatomine sex and infection with *T. cruzi* (χ^2^ = 6.20; p = <0.0128) with females more likely to be infected than males (Table 4). From 2007 to 2013 there was a progressive increase in the percentage of *T. cruzi* infections in *P. geniculatus* males and females, reaching its highest value in 2011 and then falling once again in 2012 for both sexes, although this decrease was less in females than males. In 2013, however, males reached the same percentage of infection as they did in 2011, whilst females showed a slight reduction (Figure 4).

**Table 4:** **Presence of** ***T. cruzi*** **in the digestive tracts of the triatomine species found in Caracas**

		**2007**		**2008**		**2009**		**2010**		**2011**		**2012**		**2013**		**Sub-total**	
**Species**	**Sex/Stage**	**Neg**	**Pos**	**Neg**	**Pos**	**Neg**	**Pos**	**Neg**	**Pos**	**Neg**	**Pos**	**Neg**	**Pos**	**Neg**	**Pos**	**Neg**	**Pos**
*P.s geniculatus*	Female	20	22	84	134	73	173	77	379	24	187	26	130	29	135	333	1160
	Male	14	11	61	103	71	115	122	368	13	122	23	75	10	80	314	874
	NIII	0	1	1	1	0	6	2	4	1	0	2	1	0	0	6	13
	NIV	0	5	5	2	6	7	2	8	1	0	4	4	3	7	21	33
	NV	0	1	0	1	3	3	2	3	1	2	1	2	0	0	7	12
	Sub-total	34	40	151	241	153	304	205	762	40	311	56	212	42	222	681	2092
*T. maculata*	Female	0	0	0	0	1	0	1	2	0	1	0	1	1	0	3	4
	Male	0	0	1	0	0	0	0	0	1	1	0	1	0	0	2	2
	Sub-total	0	0	1	0	1	0	1	2	1	2	0	2	1	0	5	6
*T. nigromaculata*	Female	0	0	0	1	1	2	2	2	0	0	1	0	0	0	4	5
	Male	0	0	0	0	0	0	2	3	1	0	0	0	0	0	3	3
	Sub-total	0	0	0	1	1	2	4	5	1	0	1	0	0	0	7	8
*R. prolixus*	Male	0	0	0	0	1	1	0	0	0	0	0	0	0	0	1	1
	Sub-total	0	0	0	0	1	1	0	0	0	0	0	0	0	0	1	1
Total number of triatomines		34	40	152	242	156	307	210	769	42	313	57	214	43	222	694	2107

Neg = No parasites observed under microscope examination.

Pos = *T. cruzi* observed under microscope examination.

**Table 5:** **Presence of blood and** ***T. cruzi*** **in the gut of triatomines found in Caracas**

**Parish**		**2007**		**2008**		**2009**		**2010**		**2011**		**2012**		**2013**		**2007-**	**2013**
		**B**	**NB**	**B**	**NB**	**B**	**NB**	**B**	**NB**	**B**	**NB**	**B**	**NB**	**B**	**NB**	**B**	**NB**
Altagracia	*T cruzi pos*	3	1	2	1	1	2	4	6		1	1	1		1	11	13
	*T cruzi* neg			1	2	1			1						1	2	4
23 de Enero	*T cruzi pos*				1			1	2	1	1					2	4
	*T cruzi neg*															0	0
Antímano	*T cruzi pos*			1		12	7	3	4	3		5	2	2	1	26	14
	*T cruzi neg*		1		1	4	4	3	1	1			1	1		9	8
La Candelaria	*T cruzi pos*									1	2					1	2
	*T cruzi neg*	1														1	0
Caricuao	*T cruzi pos*			2	3	2	1	10	13	2	1	1	1	3	1	20	20
	*T cruzi neg*	1		1		1	2	2	8				1	1		6	11
Catedral	*T cruzi pos*															0	0
	*T cruzi neg*															0	0
Coche	*T cruzi pos*			1			4	3						1		5	4
	*T cruzi neg*			1			1		2							1	3
El Junquito	*T cruzi pos*	2		7	2	5	3	27	31	15	5	2	4	10	5	68	50
	*T cruzi neg*	1	1	3	1	6	3	9	3	2	3		1	1		22	12
El Paraíso	*T cruzi pos*					1	2	5	3		3			1		7	8
	*T cruzi neg*									1						1	0
El Recreo	*T cruzi pos*				2	2	1		10	1	3	1	1	2	3	6	20
	*T cruzi neg*			1	1		1		2				1			1	5
El Valle	*T cruzi pos*	1						1	1	1						3	1
	*T cruzi neg*	1					1	1	2	1						3	3
La Pastora	*T cruzi pos*	3	3	13	9	11	8	31	35	18	12	8	2	6	3	90	72
	*T cruzi neg*		4	3	8	2	5	7	14	1	1	1	4	1		15	36
La Vega	*T cruzi pos*	1			2	1	2	6	7	4	5	4	4	1	4	17	24
	*T cruzi neg*			2	2		1	1	5	1				1		5	8
Macarao	*T cruzi pos*			1				1	4			1				3	4
	*T cruzi neg*			1					1							1	1
San Agustín	*T cruzi pos*							5	2	1			1			6	3
	*T cruzi neg*							1	1							1	1
San Bernardino	*T cruzi pos*				2		3		2	1	6		1	1		2	14
	*T cruzi neg*				1	2			1							2	2
San José	*T cruzi pos*				2	2	3	5	9	1	1		1			8	16
	*T cruzi neg*		1			3	1	1								4	2
San Juan	*T cruzi pos*												1			0	1
	*T cruzi neg*					1							1			1	1
San Pedro	*T cruzi pos*		1	2	1	1		4	4		1	1	1	1	1	9	9
	*T cruzi neg*			2	4		1		2			1				3	7
Santa Rosalía	*T cruzi pos*							3	1							3	1
	*T cruzi neg*								1							0	1
Santa Teresa	*T cruzi pos*			1	1		1									1	2
	*T cruzi neg*					1	1								1	1	2
Sucre	*T cruzi pos*	4		15	13	32	22	51	45	14	10	20	12	25	7	161	109
	*T cruzi neg*		2	9	9	11	14	12	4	1		4	4	1	3	38	36
El Cafetal	*T cruzi pos*	2		3	3	8	9	11	20	6	12	2	4	5	7	37	55
	*T cruzi neg*				3	2	4	3	8		2		2	1		6	19
Las Minas de Baruta	*T cruzi pos*	1	1		1	1	5	7	5	4	6	2	6	5	2	20	26
	*T cruzi neg*				6		3		3	1	1		1	4	1	5	15
Ns. Sra. R. Baruta	*T cruzi pos*	3		9	22	20	14	19	39	14	17	11	13	15	9	91	114
	*T cruzi neg*		2	5	12	8	9	12	15		3	6	2	4	4	35	47
Chacao	*T cruzi pos*			1	1		1	4		3	1			1	2	9	5
	*T cruzi neg*				5		1	1			1	2				3	7
Sta R. P. El Hatillo	*T cruzi pos*	1		2	9	15	5	22	29	6	6	7	6	5	4	58	59
	*T cruzi neg*	1	2	7	10	6	5	6	7	6	2	3	2	1	2	30	30
Caucagüita	*T cruzi pos*			2	2	6	6	13	11	3	2	1		3		28	21
	*T cruzi neg*		1	1		1	3	3					3		1	5	8
Filas de Mariche	*T cruzi pos*	5	1	8	2	12	3	29	23	8	4	9	7	7		78	40
	*T cruzi neg*	1	1	6	2	9	2	10	1	4	1	3	1	2		35	8
La Dolorita	*T cruzi pos*	1			2	3	1	6	6	3	1	2		6	2	21	12
	*T cruzi neg*		5		1		3	1	4		1			1		2	14
Leoncio Martínez	*T cruzi pos*	2		1	7	1	6	10	20	6	17	7	5	5	10	32	65
	*T cruzi neg*		1	4	5		5	2	6				1	1	1	7	19
Petare	*T cruzi pos*	2	2	42	41	39	23	85	71	40	40	34	22	41	14	283	213
	*T cruzi neg*	4	3	9	23	13	15	21	22	4	4	10	2	5	4	66	73
TOTAL	*T cruzi pos*	31	9	113	129	175	132	366	403	156	157	119	95	146	76	1106	1001
	*T cruzi neg*	10	24	56	96	71	85	96	114	23	19	30	27	25	18	311	383

B = blood present in the digestive tract; NB = no blood observed in the digestive tract.

*T. cruzi* pos = *Trypanosoma cruzi* present in the digestive tract.

*T. cruzi* neg = no parasites observed in the digestive tract.

### Study of the nymphal stages of *P. geniculatus*

Only *P. geniculatus* nymphs were identified during the seven years of the study. These were found in 14 of the 32 parishes surveyed (Table 2) and represent 3.5% of the triatomine bugs analyzed. A total of 26 nymphs were in nymphal stage V, 71 in stage IV and 27 in stage III (N = 124; Table 1). Of these, only the stomachs of 105 nymphs were in good enough conditions to be dissected, of which 18/21 stage V (85.7%), 46/61 stage IV (75.4%) and 17/23 stage III (73.9%) nymphs contained blood (Table 3). Overall, 77.1% of the nymphs examined over the seven year study period were blood-fed compared to 47.8% of the adults (both sexes). This difference was highly significant (*χ*^2^ = 34.93; p = < 0.0001) suggesting a strong association between nymphal stage and the presence of blood in the stomach in this species (Table 3). Only the guts of 92 of the nymphs captured were in good enough condition for *T. cruzi* examination and of these, 12/19 stage V (63.2%), 33/54 stage IV (61.1%), and 13/19 stage III nymphs (68.4%) were infected. The overall percentage of infection during the nymphal stages was 63.0%, significantly lower than that of adults (75.9%; both sexes) (*χ*^2^ = 7.90; p =<0.0050) (Table 4).

### Genotype of *T. cruzi* isolates

Genotype analysis of 277 *T. cruzi* isolates obtained from the 5 triatomine species identified from 26 of the parishes within the MDC, showed that they were all infected with parasites belonging to the TcI genotype (DTU I) [21], except for one isolate obtained from *P. geniculatus* that was identified as belonging to the TcIII genotype (Additional file 4: Table S4). We also included in this study two further *T. cruzi* isolates obtained from two *Eratyrus mucronatus* specimens found in Sucre Municipality in 2000 and 2001 respectively, which both belong to the TcI genotype. RAPD genotyping of the *T. cruzi* isolates was confirmed by the PCR-RFLP technique as previously reported by Carrasco et al. [20].

## Discussion

In 1986, Pifano [23] reported that of 349 *P. geniculatus* collected in Caracas over a period of 19 years, 38.7% were infected with *T. cruzi* and 50% of 36 specimens analyzed for their blood meal source had fed on human blood. In 2005, we reported natural infection rates of *P. geniculatus* with *T. cruzi* in Caracas and two neighboring states (Miranda and Vargas). Of 80 *P. geniculatus* adults examined, 52 were females (65%) and 28 males (35%). Infection with *T. cruzi* was detected in 76.1% (67/88) specimens and all the isolates were identified as belonging to the TcI genotype [12]. Analysis of the blood meal source showed that 60.2% (53/88) of the bugs had fed on human blood and 40.9% (36/88) of these were also infected with *T. cruzi*. For the present study we undertook a longitudinal survey of triatomine bugs brought by inhabitants of the Metropolitan District of Caracas to the Institute of Tropical Medicine over a period of 7 years. From January 2007 to December 2013 we gathered 3551 triatomine bugs which were taxonomically identified as *P. geniculatus* (98.96%), *T. nigromaculata* (0.59%), *T. maculata* (0.39%) and *R. prolixus* (0.06%). These results clearly show that the vast majority of triatomines that interact with humans or enter their homes in the Metropolitan District of Caracas are *P. geniculatus*. This species has thus become an urban species very well adapted to the ecological and habitat conditions throughout the city of Caracas, including high, middle and poor neighborhoods. Of the 32 parishes that make up the MDC, *P. geniculatus* was found in 31 (96.9%), with many of the specimens collected infected with *T. cruzi* and blood-fed. Besides the clear predominance of *P. geniculatus* in Caracas, we also identified *T. maculata*: a mainly sylvatic species although also reported from peridomestic and domestic habitats from the northeast coast of Venezuela (Morocoima *et al*., manuscript in preparation). Similarly, *T. nigromaculata* is a sylvatic species also reported to be undergoing a process of adaptation to human dwellings in southwestern Venezuela [24]. Although we identified only two *R. prolixus* specimens over the study period, one of these was found to be infected with *T. cruzi* and also contained blood in its stomach. This specimen was captured inside a house located in a densely populated poor area on a hill in Antimano parish, the same sector where the oral outbreak of Chagas’ disease occurred in 2010 [16]. The second *R. prolixus* specimen was found outside a house in a middle class sector in Chacao parish where an oral outbreak of Chagas’ disease was reported in 2007 [13]. The presence of *R. prolixus* in Caracas was an unexpected finding as this species is more commonly found in houses in rural parts of the country, with palm trees being its natural ecotope. In addition to these species, we have previously reported the presence of *Eratyrus mucronatus* in Filas de Mariche parish, Sucre Municipality [20], a species considered, up until now, to be strictly sylvatic in Venezuela. Overall, and including the present study, we have found 5 different species of triatomine bugs interacting with inhabitants in the MDC. Despite the extensive deforestation and large urban development that has taken place in Caracas during the last decades, forest islands still remain across the city. In addition, bordering the city is the Waraira Repano National Park, a protected forested, mountainous region, which separates Caracas from the Caribbean Sea. The National Park acts as a natural reserve for triatomines which explains the existence of species, traditionally considered sylvatic, in the urban environment. The very large number of *P. geniculatus* specimens found in the parishes of Petare and Sucre located in northeast and northwest Caracas, respectively, could be explained by several factors all of which provide favorable conditions for the successful colonization and proliferation of this species: both sectors are densely populated, both include areas of urban development in poor conditions, such as sewage disposal in precariously covered channels and the accumulation of garbage, which has led to the proliferation of rodents, and both are bordered by Waraira Repano. The monthly frequency of triatomine bugs collected in this study started to increase from the end of the Venezuelan dry season, showing a peak between April and July with maximum values in May when the rainy season begins. This behavior was shown to be consistent over the 7 year study and can be related to a seasonal proliferation of these insects. Similar results for both P. *geniculatus* and other triatomine species have been previously reported [25-28]. Of special interest is the monthly distribution of triatomines during 2010. The numbers of these bugs reached remarkably high values in May and June of this year compared those of the other six years. There are several possible explanations for this. One of these is the fact that 2010 was the driest and hottest dry season in decades, conditions that led to many forest fires in the Waraira Repano National Park thus forcing the displacement of mammals and insects and their migration out of the park. This severe dry season was followed by an extremely intense rainy season in May. Another possibility is that the second oral outbreak of Chagas’ disease occurred in Caracas in May 2010. The news of this spread rapidly and widely through the social media networks, generating great alarm and concern among the inhabitants of Caracas. This situation is a clear example of the role of social media in warning about potential risks. Perhaps it was a combination of these two factors that produced the capture of such high numbers of triatomines during May and June 2010.

Another important finding was that much higher numbers of females were collected than males coupled with a clear predominance of blood-fed females such that, according to the Chi Square analysis, there was an association between the sex of the triatomines and the presence of blood in their stomachs. The greater avidity for a blood meal exhibited by the females could be explained as a natural response to ensure oogenesis: an instinctive behavior for safeguarding the preservation of the species. In addition, female triatomines play an essential role in the invasion and colonization of new habitats. The percentage of blood in the stomach of triatomines of both sexes was relatively constant during the first 4 years of the study and then started to increase over the following 3 years. This may be due to the evolution of a more effective strategy by the insects for interacting with domestic mammals or humans to obtain a blood meal and/or a more efficient method of invading human dwellings in search of blood. Although not formally evaluated, we can assume that the blood meal source was predominantly human, especially considering that most of the individuals that brought the triatomines to the Institute of Tropical Medicine reported that they had been bitten by them and/or had found the insects inside their bedrooms. This assumption is supported by a previous study we conducted in 2005 [12] where we found that 60.2% of *P. geniculatus* had fed on human blood, as well as our more recent research using cytochrome b sequence analysis for the identification of the blood meal source, which shows that 67.4% of the *P. geniculatus* individuals analyzed had fed on humans (data not shown). Another very important observation is the fact that 75.2% of the triatomine bugs captured were found to be naturally infected with *T. cruzi* and 52.5% of these contained blood in their stomachs. The natural infection of triatomines with *T. cruzi* was higher in females than males and, according to the Chi Square analysis, there was an association between the triatomine sex and natural infection with *T. cruzi*. The percentage of infection with *T. cruzi* for both sexes showed a steady increase from 2007 to 2012 and then tended to stabilize until the end of the study in 2013. We do not know the reason for this, however, contributing factors could be a worsening of hygiene conditions in some parishes, deficiencies in the garbage collection service, the presence of synanthropic mammals such as *Didelphis marsupialis* and rodents, in particular *Rattus rattus*, and the proliferation and natural infection rates of the triatomines, which together could favor the establishment of conditions for the enzootic transmission of *T. cruzi*. This situation is particularly true for Petare parish where the highest number of triatomine bugs was found. The immature stages of the triatomines captured were all identified as III to V instar *P. geniculatus* nymphs. We found that 77.1% of the nymphs had had a blood meal and 63% were infected with *T. cruzi*. Immature stages of *P. geniculatus* were found in 14 of the 32 parishes surveyed. Although the specific site within the houses where the nymphs were found was not registered, most people reported that they had collected the insects from inside the houses or very close to them. This suggests that *P. geniculatus* has a.) become adapted to and colonized human dwellings as previously reported for the MDC [12,29] and/or b.) colonized rat burrows using this synanthropic host (often infected with *T. cruzi*) as a blood meal source, both of which have led to the successful proliferation and infection of this triatomine species. This could explain why the percentage of adults infected with *T. cruzi* was higher than that of the nymphal stages. The establishment of secondary vector species in an urban environment and their potential role for the transmission of Chagas’ disease has been reported in Salvador, Bahia State, Brazil, by Santana, et. al. [26], who identified 988 insects during a 3 year study period with *Triatoma tibiamaculata* being the predominant species (98.3%) and *P. geniculatus* (0.6%) found only occasionally. The city of Salvador is located on the coast of the South Atlantic Ocean and has a mean altitude of 50 m.a.s.l. Santana, et. al. [26] also reported that over the 3-year study period, the highest numbers of triatomines were captured in 2007 and were more abundant in January than any other month, this last probably a consequence of the seasonal proliferation of insect vectors (September-March were the months with least rain). The great majority of the *T. cruzi* isolates we identified from the five different triatomine species captured in the MDC belonged to the TcI genotype (99.6%) and were widely distributed across all sectors of the city. It is notable that the TcI strain was the genotype identified as being responsible for the orally transmitted acute cases of Chagas’ disease reported in Caracas [16,30]. Using 23 microsatellite markers, we have previously found that the same population of parasites circulating within the same areas of Caracas is responsible for infecting natural reservoirs, triatomine vectors and humans (oral transmission) [16]. In addition, using the hemoculture technique as the detection method, we found that 44 of the 95 rats (*Rattus rattus*) captured in the same area where the oral outbreak took place were infected with *T. cruzi*, and that all the *T. cruzi* isolates belonged to the TcI genotype [16,20]. All these factors clearly point towards a high potential risk of Chagas disease transmission in Caracas where the *T. cruzi* enzootic cycle has been shown to be very well established, with *P. geniculatus* as the main vector and *R. rattus* the principal reservoir for the parasite. *T. maculata*, *T. nigromaculata*, *E. mucronatus* and *R. prolixus*, although present at very low proportions in Caracas, still represent an additional risk for the vector transmission of the disease. This situation may be responsible for the deaths of three children with acute chagasic myocarditis, two of whom were treated in public hospitals in 1999 and 2005, and a third in a private hospital in 2000 (Dr. A. Maekelt and Dr. R. Espinosa, personal communication). The large urban development that has taken place over the last few decades in the Metropolitan District of Caracas has led to the deforestation of and dramatic changes to the natural habitats of vectors and hosts, and the displacement or disappearance of wild mammal fauna - the natural reservoirs of *T. cruzi* and blood meal sources for sylvatic triatomine bug species. This may have forced the triatomines into the city in the search for more stable habitats such as human dwellings, leading to their adaption to the urban habitat [31,32]. In this study we have shown clear evidence of high risk factors for the transmission of Chagas’ disease in Caracas. This requires the urgent attention of the health authorities and the implementation of campaigns for the surveillance and control of triatomines, rodents and synanthropic wild mammals such as *D. marsupialis*. In addition, permanent educational programs should be established in order to train the population to correctly identify triatomine vector species and advise them of methods to prevent these insects from entering their homes or the peridomicile.

## Conclusions

A total of five species of triatomine bug, with a high predominance of *P. geniculatus*, have been identified throughout the Metropolitan District of Caracas independent of the social class of the neighborhoods. Evidence of a blood-meal and infection by *T. cruzi* was detected in the digestive tracts of these insects in 31 of the 32 parishes surveyed. We found that more females than males were infected with *T. cruzi* and/or were blood-fed, this last being perhaps a natural response of these insects for the preservation of the species, particularly considering that females play an essential role in the invasion and colonization of new habitats. In addition, immature stages of *P. geniculatus*, most of these infected with *T. cruzi* and blood-fed, were found inside or very close to human dwellings, suggesting that this species is undergoing a domiciliation process. The increase in the triatomine population was shown to be seasonally dependent, and the differences in triatomine abundance between parishes could be associated with poor hygiene and urban development. The anthropophilic behavior of *P. geniculatus*, where humans have become an abundant blood meal source, is strongly supported by the fact that most people who brought the triatomines to the Institute of Tropical Medicine reported having being bitten or had found the insects inside their bedrooms. All these factors, in combination with our previous results of rats infected with the same population of *T. cruzi* found in chagasic patients from oral outbreaks and triatomine bugs within the same area in Caracas, demonstrate the successful establishment of a *T. cruzi* urban enzootic cycle. Our results show that there is a high risk for Chagas’ disease transmission in this capital city. Urgent actions from health authorities are required in order to deal with this alarming situation.

## Additional files

Additional file 1: Table S1. Specimen list of the triatomine bugs found in the Metropolitan District of Caracas. Additional file 2: Table S2. Population and area of the 32 parishes that constitute the Metropolitan District of Caracas. Additional file 3: Table S3. Number of triatomine bugs collected per month in the Metropolitan District of Caracas, 2007 to 2013. Additional file 4: Table S4. Genotypes of *T. cruzi* isolates obtained from the 5 species of triatomines found in Caracas.
