# Supplementary material for: Panstrongylus geniculatus and four other species of triatomine bug involved in the Trypanosoma cruzi enzootic cycle: high risk factors for Chagas’ disease transmission in the Metropolitan District of Caracas, Venezuela
Source: Parasit Vectors. 2014 Dec 23;7:602. doi: 10.1186/s13071-014-0602-7 (PMC4307744; doi:10.1186/s13071-014-0602-7)
Supplement: Additional file 1: Table S1. — Specimen list of the triatomine bugs found in the Metropolitan District of Caracas. [file 13071_2014_602_MOESM1_ESM.pdf]

Table S1 List of different specimens of triatomine bugs found in the Metropolitan District of Caracas

Number	Code	State	Municipality	Parish	Specie	Year	Sex/Stage	*T. cruzi* infection	Blood fed
1	VE1507	DC	Libertador	Altagracia	*P. geniculatus*	2007	Female	Positive	Yes
2	VE5107A	DC	Libertador	Altagracia	*P. geniculatus*	2007	Female	Positive	Yes
3	VE5107B	DC	Libertador	Altagracia	*P. geniculatus*	2007	Female	Positive	Yes
4	VE2407	DC	Libertador	Altagracia	*P. geniculatus*	2007	Female	Positive	No
5	VE4707	DC	Libertador	Antímano	*P. geniculatus*	2007	Female	Negative	No
6	VE0807	DC	Libertador	Candelaria	*P. geniculatus*	2007	Female	Negative	Yes
7	VE7207	DC	Libertador	Caricuao	*P. geniculatus*	2007	Female	Negative	Yes
8	VE8307	DC	Libertador	El Junquito	*P. geniculatus*	2007	Female	Positive	Yes
9	VE5907A	DC	Libertador	El Junquito	*P. geniculatus*	2007	Female	NA	No
10	VE5907B	DC	Libertador	El Junquito	*P. geniculatus*	2007	Female	NA	No
11	VE5907C	DC	Libertador	El Junquito	*P. geniculatus*	2007	Male	NA	No
12	VE5907D	DC	Libertador	El Junquito	*P. geniculatus*	2007	Male	NA	No
13	VE4207	DC	Libertador	El Junquito	*P. geniculatus*	2007	Male	Positive	Yes
14	VE6407	DC	Libertador	El Junquito	*P. geniculatus*	2007	Male	Negative	Yes
15	VE4307	DC	Libertador	El Junquito	*P. geniculatus*	2007	Male	Negative	No
16	VE0907	DC	Libertador	El Valle	*P. geniculatus*	2007	Female	Negative	Yes
17	VE1007	DC	Libertador	El Valle	*P. geniculatus*	2007	Male	Positive	Yes
18	VE3307A	DC	Libertador	La Pastora	*P. geniculatus*	2007	Female	Positive	Yes
19	VE3307B	DC	Libertador	La Pastora	*P. geniculatus*	2007	Male	Positive	No
20	VE3307C	DC	Libertador	La Pastora	*P. geniculatus*	2007	Male	Positive	No
21	VE3307D	DC	Libertador	La Pastora	*P. geniculatus*	2007	Male	Negative	No
22	VE3307E	DC	Libertador	La Pastora	*P. geniculatus*	2007	Male	Positive	No
23	VE6207	DC	Libertador	La Pastora	*P. geniculatus*	2007	Female	Negative	No
24	VE2507A	DC	Libertador	La Pastora	*P. geniculatus*	2007	Female	Positive	Yes
25	VE2507B	DC	Libertador	La Pastora	*P. geniculatus*	2007	Female	Positive	Yes
26	VE6507	DC	Libertador	La Pastora	*P. geniculatus*	2007	Male	Negative	No
27	VE3007	DC	Libertador	La Pastora	*P. geniculatus*	2007	Female	Negative	No
28	VE1107	DC	Libertador	La Vega	*P. geniculatus*	2007	Female	Positive	Yes
29	VE0207	DC	Libertador	San José	*P. geniculatus*	2007	Female	Negative	No
30	PgHC07	DC	Libertador	San Pedro	*P. geniculatus*	2007	Male	Positive	No
31	VE3607	DC	Libertador	Sucre	*P. geniculatus*	2007	Male	Negative	No
32	VE8607A	DC	Libertador	Sucre	*P. geniculatus*	2007	Nymph III	Positive	Yes
33	VE8607C	DC	Libertador	Sucre	*P. geniculatus*	2007	Nymph IV	Positive	Yes
34	VE8607D	DC	Libertador	Sucre	*P. geniculatus*	2007	Nymph IV	Positive	Yes
35	VE8607E	DC	Libertador	Sucre	*P. geniculatus*	2007	Nymph IV	Positive	Yes
36	VE1407	DC	Libertador	Sucre	*P. geniculatus*	2007	Male	Negative	No
37	VE4407	Miranda	Sucre	Caucagüita	*P. geniculatus*	2007	Female	Negative	No
38	VE3707	Miranda	Baruta	El Cafetal	*P. geniculatus*	2007	Female	Positive	Yes
39	VE0107	Miranda	Baruta	El Cafetal	*P. geniculatus*	2007	Female	Positive	Yes
40	VE3807	Miranda	Sucre	Filas de mariche	*P. geniculatus*	2007	Male	Positive	Yes

41	VE6907	Miranda	Sucre	Filas de mariche	*P. geniculatus*	2007	Female	Negative	No
42	VE1607	Miranda	Sucre	Filas de mariche	*P. geniculatus*	2007	Female	Negative	Yes
43	VE3107	Miranda	Sucre	Filas de mariche	*P. geniculatus*	2007	Female	Positive	Yes
44	VE3207	Miranda	Sucre	Filas de mariche	*P. geniculatus*	2007	Male	Positive	Yes
45	VE2707	Miranda	Sucre	Filas de mariche	*P. geniculatus*	2007	Female	Positive	No
46	VE2907	Miranda	Sucre	Filas de mariche	*P. geniculatus*	2007	Female	Positive	Yes
47	VE5007	Miranda	Sucre	Filas de mariche	*P. geniculatus*	2007	Female	Positive	Yes
48	VE6607	Miranda	Sucre	La Dolorita	*P. geniculatus*	2007	Male	Negative	No
49	VE6807	Miranda	Sucre	La Dolorita	*P. geniculatus*	2007	Female	Positive	Yes
50	VE8207	Miranda	Sucre	La Dolorita	*P. geniculatus*	2007	Female	Negative	No
51	VE0407	Miranda	Sucre	La Dolorita	*P. geniculatus*	2007	Female	Negative	No
52	VE0307	Miranda	Sucre	La Dolorita	*P. geniculatus*	2007	Female	Negative	No
53	VE3907	Miranda	Sucre	La Dolorita	*P. geniculatus*	2007	Female	Negative	No
54	VE2307	Miranda	Baruta	Las Minas de Baruta	*P. geniculatus*	2007	Male	Positive	No
55	VE8507	Miranda	Baruta	Las Minas de Baruta	*P. geniculatus*	2007	Nymph IV	Positive	Yes
56	VE8707	Miranda	Sucre	Leoncio Martínez	*P. geniculatus*	2007	Female	Positive	Yes
57	VE1707	Miranda	Sucre	Leoncio Martínez	*P. geniculatus*	2007	Female	Positive	Yes
58	VE1907	Miranda	Sucre	Leoncio Martínez	*P. geniculatus*	2007	Male	Negative	No
59	VE2107	Miranda	Baruta	Nuestra Señora del Rosario de Baruta	*P. geniculatus*	2007	Male	Negative	No
60	VE5807	Miranda	Baruta	Nuestra Señora del Rosario de Baruta	*P. geniculatus*	2007	Female	Positive	Yes
61	VE8607B	Miranda	Baruta	Nuestra Señora del Rosario de Baruta	*P. geniculatus*	2007	Nymph IV	Positive	Yes
62	VE5407	Miranda	Baruta	Nuestra Señora del Rosario de Baruta	*P. geniculatus*	2007	Female	Positive	Yes
63	VE0607	Miranda	Baruta	Nuestra Señora del Rosario de Baruta	*P. geniculatus*	2007	Male	Negative	No
64	VE2607	Miranda	Sucre	Petare	*P. geniculatus*	2007	Female	Negative	Yes
65	VE3507	Miranda	Sucre	Petare	*P. geniculatus*	2007	Male	Negative	Yes
66	VE1807	Miranda	Sucre	Petare	*P. geniculatus*	2007	Female	Negative	Yes
67	VE2207	Miranda	Sucre	Petare	*P. geniculatus*	2007	Female	Negative	Yes
68	VE4807A	Miranda	Sucre	Petare	*P. geniculatus*	2007	Male	Positive	Yes
69	VE4807B	Miranda	Sucre	Petare	*P. geniculatus*	2007	Female	Positive	No
70	VE4807C	Miranda	Sucre	Petare	*P. geniculatus*	2007	Male	Positive	No
71	VE4907	Miranda	Sucre	Petare	*P. geniculatus*	2007	Female	Negative	No
72	VE7907	Miranda	Sucre	Petare	*P. geniculatus*	2007	Female	Negative	No
73	VE4507	Miranda	Sucre	Petare	*P. geniculatus*	2007	Male	Negative	No
74	VE8407	Miranda	Sucre	Petare	*P. geniculatus*	2007	Nymph V	Positive	Yes
75	VE4607	Miranda	El Hatillo	Santa Rosalía de Palermo	*P. geniculatus*	2007	Female	Positive	Yes
76	VE0707	Miranda	El Hatillo	Santa Rosalía de Palermo	*P. geniculatus*	2007	Male	Negative	No
77	VE1207	Miranda	El Hatillo	Santa Rosalía de Palermo	*P. geniculatus*	2007	Male	Negative	Yes
78	VE7407	Miranda	El Hatillo	Santa Rosalía de Palermo	*P. geniculatus*	2007	Female	Negative	No
79	VE43508	DC	Libertador	23 de Enero	*P. geniculatus*	2008	Male	Positive	No
80	VE51008	DC	Libertador	Altagracia	*P. geniculatus*	2008	Female	Negative	No
81	VE53008	DC	Libertador	Altagracia	*P. geniculatus*	2008	Nymph IV	Negative	No
82	VE47308	DC	Libertador	Altagracia	*P. geniculatus*	2008	Male	Negative	Yes

83	VE29008	DC	Libertador	Altagracia	*P. geniculatus*	2008	Female	Positive	No
84	VE41108	DC	Libertador	Altagracia	*P. geniculatus*	2008	Female	Positive	Yes
85	VE49408	DC	Libertador	Altagracia	*P. geniculatus*	2008	Female	Positive	Yes
86	VE18008	DC	Libertador	Antímano	*P. geniculatus*	2008	Male	Negative	No
87	VE32008	DC	Libertador	Antímano	*P. geniculatus*	2008	Female	Positive	Yes
88	VE8808	DC	Libertador	Caricuao	*P. geniculatus*	2008	Female	Negative	Yes
89	VE31608	DC	Libertador	Caricuao	*P. geniculatus*	2008	Female	Positive	No
90	VE49108	DC	Libertador	Caricuao	*P. geniculatus*	2008	Male	Positive	No
91	VE46308A	DC	Libertador	Caricuao	*P. geniculatus*	2008	Female	Positive	No
92	VE42208	DC	Libertador	Caricuao	*P. geniculatus*	2008	Female	Positive	Yes
93	VE11708	DC	Libertador	Caricuao	*P. geniculatus*	2008	Female	Positive	Yes
94	VE10108	DC	Libertador	Coche	*P. geniculatus*	2008	Female	Negative	Yes
95	VE54408	DC	Libertador	Coche	*P. geniculatus*	2008	Female	Positive	Yes
96	VE41908	DC	Libertador	El Junquito	*P. geniculatus*	2008	Female	Negative	No
97	VE4908	DC	Libertador	El Junquito	*P. geniculatus*	2008	Male	Negative	Yes
98	VE12908	DC	Libertador	El Junquito	*P. geniculatus*	2008	Male	Negative	Yes
99	VE49808	DC	Libertador	El Junquito	*P. geniculatus*	2008	Nymph IV	Negative	Yes
100	VE0908	DC	Libertador	El Junquito	*P. geniculatus*	2008	Female	Positive	No
101	VE11508	DC	Libertador	El Junquito	*P. geniculatus*	2008	Female	Positive	No
102	VE58608	DC	Libertador	El Junquito	*P. geniculatus*	2008	Female	Positive	Yes
103	VE47008	DC	Libertador	El Junquito	*P. geniculatus*	2008	Male	Positive	Yes
104	VE46808	DC	Libertador	El Junquito	*P. geniculatus*	2008	Female	Positive	Yes
105	VE58108	DC	Libertador	El Junquito	*P. geniculatus*	2008	Female	Positive	Yes
106	VE10708	DC	Libertador	El Junquito	*P. geniculatus*	2008	Female	Positive	Yes
107	VE12708	DC	Libertador	El Junquito	*P. geniculatus*	2008	Female	Positive	Yes
108	VE36308	DC	Libertador	El Junquito	*P. geniculatus*	2008	Female	Positive	Yes
109	VE42808B	DC	Libertador	El Recreo	*P. geniculatus*	2008	Male	Negative	No
110	VE39108	DC	Libertador	El Recreo	*P. geniculatus*	2008	Female	Negative	Yes
111	VE51308	DC	Libertador	El Recreo	*P. geniculatus*	2008	Female	Positive	No
112	VE12508	DC	Libertador	El Recreo	*P. geniculatus*	2008	Female	Positive	No
113	VE44308	DC	Libertador	La Pastora	*P. geniculatus*	2008	Male	Negative	No
114	VE415A08	DC	Libertador	La Pastora	*P. geniculatus*	2008	Female	Negative	No
115	VE415B08	DC	Libertador	La Pastora	*P. geniculatus*	2008	Female	Negative	No
116	VE54508	DC	Libertador	La Pastora	*P. geniculatus*	2008	Nymph IV	Negative	No
117	VE49508	DC	Libertador	La Pastora	*P. geniculatus*	2008	Male	Negative	No
118	VE19508	DC	Libertador	La Pastora	*P. geniculatus*	2008	Female	Negative	No
119	VE51508	DC	Libertador	La Pastora	*P. geniculatus*	2008	Male	Negative	No
120	VE57408	DC	Libertador	La Pastora	*P. geniculatus*	2008	Male	Negative	No
121	VE58908	DC	Libertador	La Pastora	*P. geniculatus*	2008	Female	Negative	Yes
122	VE6908	DC	Libertador	La Pastora	*P. geniculatus*	2008	Female	Negative	Yes
123	VE57208	DC	Libertador	La Pastora	*P. geniculatus*	2008	Female	Negative	Yes
124	VE7508B	DC	Libertador	La Pastora	*P. geniculatus*	2008	Male	Positive	No

125	VE0308	DC	Libertador	La Pastora	*P. geniculatus*	2008	Female	Positive	No
126	VE0708	DC	Libertador	La Pastora	*P. geniculatus*	2008	Female	Positive	No
127	VE32108	DC	Libertador	La Pastora	*P. geniculatus*	2008	Male	Positive	No
128	VE5908	DC	Libertador	La Pastora	*P. geniculatus*	2008	Male	Positive	No
129	VE20208	DC	Libertador	La Pastora	*P. geniculatus*	2008	Male	Positive	No
130	VE8308	DC	Libertador	La Pastora	*P. geniculatus*	2008	Female	Positive	No
131	VE9608	DC	Libertador	La Pastora	*P. geniculatus*	2008	Male	Positive	No
132	VE5508	DC	Libertador	La Pastora	*P. geniculatus*	2008	Male	Positive	No
133	VE43708	DC	Libertador	La Pastora	*P. geniculatus*	2008	Female	Positive	Yes
134	VE7508A	DC	Libertador	La Pastora	*P. geniculatus*	2008	Female	Positive	Yes
135	VE56408	DC	Libertador	La Pastora	*P. geniculatus*	2008	Female	Positive	Yes
136	VE24008	DC	Libertador	La Pastora	*P. geniculatus*	2008	Female	Positive	Yes
137	VE2708	DC	Libertador	La Pastora	*P. geniculatus*	2008	Female	Positive	Yes
138	VE38208	DC	Libertador	La Pastora	*P. geniculatus*	2008	Female	Positive	Yes
139	VE38508A	DC	Libertador	La Pastora	*P. geniculatus*	2008	Female	Positive	Yes
140	VE19408	DC	Libertador	La Pastora	*P. geniculatus*	2008	Female	Positive	Yes
141	VE45608	DC	Libertador	La Pastora	*P. geniculatus*	2008	Male	Positive	Yes
142	VE12008	DC	Libertador	La Pastora	*P. geniculatus*	2008	Female	Positive	Yes
143	VE28308	DC	Libertador	La Pastora	*P. geniculatus*	2008	Female	Positive	Yes
144	VE24308	DC	Libertador	La Pastora	*P. geniculatus*	2008	Male	Positive	Yes
145	VE37908	DC	Libertador	La Pastora	*P. geniculatus*	2008	Male	Positive	Yes
146	VE55208	DC	Libertador	La Vega	*P. geniculatus*	2008	Male	Negative	No
147	VE47408	DC	Libertador	La Vega	*P. geniculatus*	2008	Male	Negative	No
148	VE34908	DC	Libertador	La Vega	*P. geniculatus*	2008	Female	Negative	Yes
149	VE54808	DC	Libertador	La Vega	*P. geniculatus*	2008	Female	Negative	Yes
150	VE36208	DC	Libertador	La Vega	*P. geniculatus*	2008	Male	Positive	No
151	VE50208	DC	Libertador	La Vega	*P. geniculatus*	2008	Male	Positive	No
152	VE10008	DC	Libertador	Macarao	*P. geniculatus*	2008	Female	Negative	Yes
153	VE13408	DC	Libertador	Macarao	*P. geniculatus*	2008	Male	Positive	Yes
154	VE55408	DC	Libertador	San Bernardino	*P. geniculatus*	2008	Male	Negative	No
155	VE54108	DC	Libertador	San Bernardino	*P. geniculatus*	2008	Male	Positive	No
156	VE15208	DC	Libertador	San Bernardino	*P. geniculatus*	2008	Male	Positive	No
157	VE5208	DC	Libertador	San José	*P. geniculatus*	2008	Male	Positive	No
158	VE1908	DC	Libertador	San José	*P. geniculatus*	2008	Female	Positive	No
159	VE19308	DC	Libertador	San Pedro	*P. geniculatus*	2008	Female	Negative	No
160	VE50608	DC	Libertador	San Pedro	*P. geniculatus*	2008	Female	Negative	No
161	VE46608	DC	Libertador	San Pedro	*P. geniculatus*	2008	Male	Negative	No
162	VE11908	DC	Libertador	San Pedro	*P. geniculatus*	2008	Male	Negative	No
163	VE54708	DC	Libertador	San Pedro	*P. geniculatus*	2008	Female	Negative	Yes
164	VE59608	DC	Libertador	San Pedro	*P. geniculatus*	2008	Female	Negative	Yes
165	VE5408	DC	Libertador	San Pedro	*P. geniculatus*	2008	Male	Positive	No
166	VE7608	DC	Libertador	San Pedro	*P. geniculatus*	2008	Female	Positive	Yes

167	VE7008	DC	Libertador	San Pedro	*P. geniculatus*	2008	Male	Positive	Yes
168	VE7208	DC	Libertador	Santa Teresa	*P. geniculatus*	2008	Male	Positive	No
169	VE23908	DC	Libertador	Santa Teresa	*P. geniculatus*	2008	Male	Positive	Yes
170	VE3708A	DC	Libertador	Sucre	*P. geniculatus*	2008	Female	NA	NA
171	VE3708B	DC	Libertador	Sucre	*P. geniculatus*	2008	Female	NA	NA
172	VE3708C	DC	Libertador	Sucre	*P. geniculatus*	2008	Nymph IV	NA	NA
173	VE3708D	DC	Libertador	Sucre	*P. geniculatus*	2008	Nymph IV	NA	NA
174	VE3708E	DC	Libertador	Sucre	*P. geniculatus*	2008	Nymph IV	NA	NA
175	VE1408A	DC	Libertador	Sucre	*T. maculata*	2008	Male	Negative	No
176	VE26808	DC	Libertador	Sucre	*P. geniculatus*	2008	Male	Negative	No
177	VE22108	DC	Libertador	Sucre	*P. geniculatus*	2008	Female	Negative	No
178	VE30108	DC	Libertador	Sucre	*P. geniculatus*	2008	Male	Negative	No
179	VE9508A	DC	Libertador	Sucre	*P. geniculatus*	2008	Male	Negative	No
180	VE57108	DC	Libertador	Sucre	*P. geniculatus*	2008	Female	Negative	No
181	VE42108	DC	Libertador	Sucre	*P. geniculatus*	2008	Female	Negative	No
182	VE39608	DC	Libertador	Sucre	*P. geniculatus*	2008	Female	Negative	No
183	VE40508	DC	Libertador	Sucre	*P. geniculatus*	2008	Female	Negative	Yes
184	VE43308	DC	Libertador	Sucre	*P. geniculatus*	2008	Female	Negative	Yes
185	VE21508A	DC	Libertador	Sucre	*P. geniculatus*	2008	Female	Negative	Yes
186	VE50408	DC	Libertador	Sucre	*P. geniculatus*	2008	Female	Negative	Yes
187	VE45308	DC	Libertador	Sucre	*P. geniculatus*	2008	Female	Negative	Yes
188	VE8707	DC	Libertador	Sucre	*P. geniculatus*	2008	Male	Negative	Yes
189	VE47708	DC	Libertador	Sucre	*P. geniculatus*	2008	Male	Negative	Yes
190	VE53108	DC	Libertador	Sucre	*P. geniculatus*	2008	Female	Negative	Yes
191	VE50308	DC	Libertador	Sucre	*P. geniculatus*	2008	Female	Negative	Yes
192	VE53508	DC	Libertador	Sucre	*P. geniculatus*	2008	Female	Negative	No
193	VE56308	DC	Libertador	Sucre	*P. geniculatus*	2008	Male	Positive	No
194	VE30608	DC	Libertador	Sucre	*P. geniculatus*	2008	Female	Positive	No
195	VE2308	DC	Libertador	Sucre	*P. geniculatus*	2008	Female	Positive	No
196	VE18708	DC	Libertador	Sucre	*P. geniculatus*	2008	Male	Positive	No
197	VE17208	DC	Libertador	Sucre	*P. geniculatus*	2008	Female	Positive	No
198	VE2008	DC	Libertador	Sucre	*P. geniculatus*	2008	Female	Positive	No
199	VE21108	DC	Libertador	Sucre	*P. geniculatus*	2008	Male	Positive	No
200	VE43608	DC	Libertador	Sucre	*P. geniculatus*	2008	Female	Positive	No
201	VE9508B	DC	Libertador	Sucre	*P. geniculatus*	2008	Male	Positive	No
202	VE59908	DC	Libertador	Sucre	*P. geniculatus*	2008	Male	Positive	No
203	VE47808	DC	Libertador	Sucre	*P. geniculatus*	2008	Male	Positive	No
204	VE12108	DC	Libertador	Sucre	*P. geniculatus*	2008	Female	Positive	No
205	VE52408	DC	Libertador	Sucre	*P. geniculatus*	2008	Female	Positive	No
206	VE9108	DC	Libertador	Sucre	*P. geniculatus*	2008	Male	Positive	Yes
207	VE9408	DC	Libertador	Sucre	*P. geniculatus*	2008	Female	Positive	Yes
208	VE42008	DC	Libertador	Sucre	*P. geniculatus*	2008	Male	Positive	Yes

209	VE6008	DC	Libertador	Sucre	*P. geniculatus*	2008	Female	Positive	Yes
210	VE0108	DC	Libertador	Sucre	*P. geniculatus*	2008	Female	Positive	Yes
211	VE14408	DC	Libertador	Sucre	*P. geniculatus*	2008	Female	Positive	Yes
212	VE3208	DC	Libertador	Sucre	*P. geniculatus*	2008	Male	Positive	Yes
213	VE3708F	DC	Libertador	Sucre	*P. geniculatus*	2008	Female	Positive	Yes
214	VE54008	DC	Libertador	Sucre	*P. geniculatus*	2008	Female	Positive	Yes
215	VE14308	DC	Libertador	Sucre	*P. geniculatus*	2008	Male	Positive	Yes
216	VE14108	DC	Libertador	Sucre	*P. geniculatus*	2008	Male	Positive	Yes
217	VE10408	DC	Libertador	Sucre	*P. geniculatus*	2008	Female	Positive	Yes
218	VE58708	DC	Libertador	Sucre	*P. geniculatus*	2008	Male	Positive	Yes
219	VE39508	DC	Libertador	Sucre	*P. geniculatus*	2008	Female	Positive	Yes
220	VE9908	DC	Libertador	Sucre	*P. geniculatus*	2008	Female	Positive	Yes
221	VE30308	Miranda	Baruta	El Cafetal	*P. geniculatus*	2008	Female	Negative	No
222	VE46108	Miranda	Baruta	El Cafetal	*P. geniculatus*	2008	Male	Negative	No
223	VE46908	Miranda	Baruta	El Cafetal	*P. geniculatus*	2008	Male	Negative	No
224	VE28008	Miranda	Baruta	El Cafetal	*P. geniculatus*	2008	Male	Positive	No
225	VE31808	Miranda	Baruta	El Cafetal	*P. geniculatus*	2008	Male	Positive	No
226	VE48308B	Miranda	Baruta	El Cafetal	*P. geniculatus*	2008	Male	Positive	No
227	VE45708	Miranda	Baruta	El Cafetal	*P. geniculatus*	2008	Male	Positive	Yes
228	VE48308A	Miranda	Baruta	El Cafetal	*P. geniculatus*	2008	Male	Positive	Yes
229	VE55008	Miranda	Baruta	El Cafetal	*P. geniculatus*	2008	Female	Positive	Yes
230	VE40308	Miranda	Baruta	Las Minas de Baruta	*P. geniculatus*	2008	Male	Negative	No
231	VE44108	Miranda	Baruta	Las Minas de Baruta	*P. geniculatus*	2008	Male	Negative	No
232	VE45408	Miranda	Baruta	Las Minas de Baruta	*P. geniculatus*	2008	Male	Negative	No
233	VE55608(A)	Miranda	Baruta	Las Minas de Baruta	*P. geniculatus*	2008	Nymph IV	Negative	No
234	VE55608(B)	Miranda	Baruta	Las Minas de Baruta	*P. geniculatus*	2008	Nymph IV	Negative	No
235	VE49008B	Miranda	Baruta	Las Minas de Baruta	*P. geniculatus*	2008	Male	Negative	No
236	VE49008A	Miranda	Baruta	Las Minas de Baruta	*P. geniculatus*	2008	Male	Positive	No
237	VE1108A	Miranda	Baruta	Nuestra Señora del Rosario de Baruta	*P. geniculatus*	2008	Male	Negative	No
238	VE15008	Miranda	Baruta	Nuestra Señora del Rosario de Baruta	*P. geniculatus*	2008	Male	Negative	No
239	VE16908	Miranda	Baruta	Nuestra Señora del Rosario de Baruta	*P. geniculatus*	2008	Male	Negative	No
240	VE21708	Miranda	Baruta	Nuestra Señora del Rosario de Baruta	*P. geniculatus*	2008	Female	Negative	No
241	VE24908	Miranda	Baruta	Nuestra Señora del Rosario de Baruta	*P. geniculatus*	2008	Female	Negative	No
242	VE34408	Miranda	Baruta	Nuestra Señora del Rosario de Baruta	*P. geniculatus*	2008	Male	Negative	No
243	VE37008	Miranda	Baruta	Nuestra Señora del Rosario de Baruta	*P. geniculatus*	2008	Male	Negative	No
244	VE41408	Miranda	Baruta	Nuestra Señora del Rosario de Baruta	*P. geniculatus*	2008	Male	Negative	No
245	VE50508	Miranda	Baruta	Nuestra Señora del Rosario de Baruta	*P. geniculatus*	2008	Female	Negative	No
246	VE54208	Miranda	Baruta	Nuestra Señora del Rosario de Baruta	*P. geniculatus*	2008	Male	Negative	No
247	VE56808	Miranda	Baruta	Nuestra Señora del Rosario de Baruta	*P. geniculatus*	2008	Female	Negative	No
248	VE51208	Miranda	Baruta	Nuestra Señora del Rosario de Baruta	*P. geniculatus*	2008	Female	Negative	No
249	VE23008	Miranda	Baruta	Nuestra Señora del Rosario de Baruta	*P. geniculatus*	2008	Female	Negative	Yes
250	VE38708	Miranda	Baruta	Nuestra Señora del Rosario de Baruta	*P. geniculatus*	2008	Female	Negative	Yes

251	VE39008	Miranda	Baruta	Nuestra Señora del Rosario de Baruta	*P. geniculatus*	2008	Female	Negative	Yes
252	VE43208	Miranda	Baruta	Nuestra Señora del Rosario de Baruta	*P. geniculatus*	2008	Female	Negative	Yes
253	VE47608	Miranda	Baruta	Nuestra Señora del Rosario de Baruta	*P. geniculatus*	2008	Male	Negative	Yes
254	VE10808A	Miranda	Baruta	Nuestra Señora del Rosario de Baruta	*P. geniculatus*	2008	Female	Positive	No
255	VE10808B	Miranda	Baruta	Nuestra Señora del Rosario de Baruta	*P. geniculatus*	2008	Female	Positive	No
256	VE14808	Miranda	Baruta	Nuestra Señora del Rosario de Baruta	*P. geniculatus*	2008	Female	Positive	No
257	VE15508	Miranda	Baruta	Nuestra Señora del Rosario de Baruta	*P. geniculatus*	2008	Male	Positive	No
258	VE1708	Miranda	Baruta	Nuestra Señora del Rosario de Baruta	*P. geniculatus*	2008	Female	Positive	No
259	VE19208	Miranda	Baruta	Nuestra Señora del Rosario de Baruta	*P. geniculatus*	2008	Male	Positive	No
260	VE20508	Miranda	Baruta	Nuestra Señora del Rosario de Baruta	*P. geniculatus*	2008	Male	Positive	No
261	VE20608A	Miranda	Baruta	Nuestra Señora del Rosario de Baruta	*P. geniculatus*	2008	Male	Positive	No
262	VE25408	Miranda	Baruta	Nuestra Señora del Rosario de Baruta	*P. geniculatus*	2008	Male	Positive	No
263	VE26608	Miranda	Baruta	Nuestra Señora del Rosario de Baruta	*P. geniculatus*	2008	Male	Positive	No
264	VE27308	Miranda	Baruta	Nuestra Señora del Rosario de Baruta	*P. geniculatus*	2008	Male	Positive	No
265	VE28608	Miranda	Baruta	Nuestra Señora del Rosario de Baruta	*P. geniculatus*	2008	Female	Positive	No
266	VE35208	Miranda	Baruta	Nuestra Señora del Rosario de Baruta	*P. geniculatus*	2008	Male	Positive	No
267	VE38008	Miranda	Baruta	Nuestra Señora del Rosario de Baruta	*P. geniculatus*	2008	Male	Positive	No
268	VE44008	Miranda	Baruta	Nuestra Señora del Rosario de Baruta	*P. geniculatus*	2008	Male	Positive	No
269	VE48208	Miranda	Baruta	Nuestra Señora del Rosario de Baruta	*P. geniculatus*	2008	Female	Positive	No
270	VE49708	Miranda	Baruta	Nuestra Señora del Rosario de Baruta	*P. geniculatus*	2008	Female	Positive	No
271	VE51608	Miranda	Baruta	Nuestra Señora del Rosario de Baruta	*P. geniculatus*	2008	Female	Positive	No
272	VE51908	Miranda	Baruta	Nuestra Señora del Rosario de Baruta	*P. geniculatus*	2008	Female	Positive	No
273	VE52108	Miranda	Baruta	Nuestra Señora del Rosario de Baruta	*P. geniculatus*	2008	Female	Positive	No
274	VE52508	Miranda	Baruta	Nuestra Señora del Rosario de Baruta	*P. geniculatus*	2008	Female	Positive	No
275	VE31408	Miranda	Baruta	Nuestra Señora del Rosario de Baruta	*T. nigromaculata*	2008	Female	Positive	No
276	VE32508	Miranda	Baruta	Nuestra Señora del Rosario de Baruta	*P. geniculatus*	2008	Female	Positive	Yes
277	VE18108	Miranda	Baruta	Nuestra Señora del Rosario de Baruta	*P. geniculatus*	2008	Female	Positive	Yes
278	VE19808	Miranda	Baruta	Nuestra Señora del Rosario de Baruta	*P. geniculatus*	2008	Male	Positive	Yes
279	VE20008	Miranda	Baruta	Nuestra Señora del Rosario de Baruta	*P. geniculatus*	2008	Female	Positive	Yes
280	VE24508	Miranda	Baruta	Nuestra Señora del Rosario de Baruta	*P. geniculatus*	2008	Female	Positive	Yes
281	VE28108	Miranda	Baruta	Nuestra Señora del Rosario de Baruta	*P. geniculatus*	2008	Male	Positive	Yes
282	VE38308	Miranda	Baruta	Nuestra Señora del Rosario de Baruta	*P. geniculatus*	2008	Male	Positive	Yes
283	VE58208	Miranda	Baruta	Nuestra Señora del Rosario de Baruta	*P. geniculatus*	2008	Female	Positive	Yes
284	VE8508	Miranda	Baruta	Nuestra Señora del Rosario de Baruta	*P. geniculatus*	2008	Female	Positive	Yes
285	VE20408	Miranda	Chacao	Chacao	*P. geniculatus*	2008	Female	Negative	No
286	VE27708	Miranda	Chacao	Chacao	*P. geniculatus*	2008	Male	Negative	No
287	VE33308A	Miranda	Chacao	Chacao	*P. geniculatus*	2008	Male	Negative	No
288	VE45008	Miranda	Chacao	Chacao	*P. geniculatus*	2008	Male	Negative	No
289	VE57008	Miranda	Chacao	Chacao	*P. geniculatus*	2008	Female	Negative	No
290	VE18808	Miranda	Chacao	Chacao	*P. geniculatus*	2008	Male	Positive	No
291	VE31108	Miranda	Chacao	Chacao	*P. geniculatus*	2008	Female	Positive	Yes
292	VE2408B	Miranda	El Hatillo	Santa Rosalía de Palermo	*P. geniculatus*	2008	Male	Negative	No

293	VE12308	Miranda	El Hatillo	Santa Rosalía de Palermo	*P. geniculatus*	2008	Female	Negative	No
294	VE20908	Miranda	El Hatillo	Santa Rosalía de Palermo	*P. geniculatus*	2008	Male	Negative	No
295	VE23108	Miranda	El Hatillo	Santa Rosalía de Palermo	*P. geniculatus*	2008	Female	Negative	No
296	VE41308	Miranda	El Hatillo	Santa Rosalía de Palermo	*P. geniculatus*	2008	Male	Negative	No
297	VE50908	Miranda	El Hatillo	Santa Rosalía de Palermo	*P. geniculatus*	2008	Nymph III	Negative	No
298	VE51408(A)	Miranda	El Hatillo	Santa Rosalía de Palermo	*P. geniculatus*	2008	Male	Negative	No
299	VE51408(B)	Miranda	El Hatillo	Santa Rosalía de Palermo	*P. geniculatus*	2008	Male	Negative	No
300	VE51708	Miranda	El Hatillo	Santa Rosalía de Palermo	*P. geniculatus*	2008	Female	Negative	No
301	VE55308	Miranda	El Hatillo	Santa Rosalía de Palermo	*P. geniculatus*	2008	Male	Negative	No
302	VE24108	Miranda	El Hatillo	Santa Rosalía de Palermo	*P. geniculatus*	2008	Female	Negative	Yes
303	VE25008	Miranda	El Hatillo	Santa Rosalía de Palermo	*P. geniculatus*	2008	Female	Negative	Yes
304	VE2908	Miranda	El Hatillo	Santa Rosalía de Palermo	*P. geniculatus*	2008	Female	Negative	Yes
305	VE35608	Miranda	El Hatillo	Santa Rosalía de Palermo	*P. geniculatus*	2008	Female	Negative	Yes
306	VE45108	Miranda	El Hatillo	Santa Rosalía de Palermo	*P. geniculatus*	2008	Female	Negative	Yes
307	VE48708	Miranda	El Hatillo	Santa Rosalía de Palermo	*P. geniculatus*	2008	Female	Negative	Yes
308	VE59308	Miranda	El Hatillo	Santa Rosalía de Palermo	*P. geniculatus*	2008	Male	Negative	Yes
309	VE0408	Miranda	El Hatillo	Santa Rosalía de Palermo	*P. geniculatus*	2008	Female	Positive	No
310	VE1308	Miranda	El Hatillo	Santa Rosalía de Palermo	*P. geniculatus*	2008	Male	Positive	No
311	VE22608	Miranda	El Hatillo	Santa Rosalía de Palermo	*P. geniculatus*	2008	Female	Positive	No
312	VE26508	Miranda	El Hatillo	Santa Rosalía de Palermo	*P. geniculatus*	2008	Female	Positive	No
313	VE27008A	Miranda	El Hatillo	Santa Rosalía de Palermo	*P. geniculatus*	2008	Male	Positive	No
314	VE29108	Miranda	El Hatillo	Santa Rosalía de Palermo	*P. geniculatus*	2008	Female	Positive	No
315	VE36508	Miranda	El Hatillo	Santa Rosalía de Palermo	*P. geniculatus*	2008	Male	Positive	No
316	VE38908	Miranda	El Hatillo	Santa Rosalía de Palermo	*P. geniculatus*	2008	Female	Positive	No
317	VE46208	Miranda	El Hatillo	Santa Rosalía de Palermo	*P. geniculatus*	2008	Female	Positive	No
318	VE24608	Miranda	El Hatillo	Santa Rosalía de Palermo	*P. geniculatus*	2008	Female	Positive	Yes
319	VE31208A	Miranda	El Hatillo	Santa Rosalía de Palermo	*P. geniculatus*	2008	Female	Positive	Yes
320	VE15708	Miranda	Sucre	Caucagüita	*P. geniculatus*	2008	Female	Negative	Yes
321	VE16208	Miranda	Sucre	Caucagüita	*P. geniculatus*	2008	Female	Positive	No
322	VE23708	Miranda	Sucre	Caucagüita	*P. geniculatus*	2008	Female	Positive	No
323	VE16108	Miranda	Sucre	Caucagüita	*P. geniculatus*	2008	Male	Positive	Yes
324	VE35508	Miranda	Sucre	Caucagüita	*P. geniculatus*	2008	Male	Positive	Yes
325	VE55708	Miranda	Sucre	Filas de mariche	*P. geniculatus*	2008	Nymph IV	NA	NA
326	VE52208	Miranda	Sucre	Filas de mariche	*P. geniculatus*	2008	Female	Negative	No
327	VE57708	Miranda	Sucre	Filas de mariche	*P. geniculatus*	2008	Male	Negative	No
328	VE11608	Miranda	Sucre	Filas de mariche	*P. geniculatus*	2008	Female	Negative	Yes
329	VE36708	Miranda	Sucre	Filas de mariche	*P. geniculatus*	2008	Female	Negative	Yes
330	VE40208	Miranda	Sucre	Filas de mariche	*P. geniculatus*	2008	Female	Negative	Yes
331	VE45508	Miranda	Sucre	Filas de mariche	*P. geniculatus*	2008	Female	Negative	Yes
332	VE55108	Miranda	Sucre	Filas de mariche	*P. geniculatus*	2008	Female	Negative	Yes
333	VE57308	Miranda	Sucre	Filas de mariche	*P. geniculatus*	2008	Female	Negative	Yes
334	VE59708	Miranda	Sucre	Filas de mariche	*P. geniculatus*	2008	Female	Positive	Yes

335	VE1008	Miranda	Sucre	Filas de mariche	*P. geniculatus*	2008	Female	Positive	No
336	VE20108	Miranda	Sucre	Filas de mariche	*P. geniculatus*	2008	Female	Positive	No
337	VE12808	Miranda	Sucre	Filas de mariche	*P. geniculatus*	2008	Male	Positive	Yes
338	VE34108A	Miranda	Sucre	Filas de mariche	*P. geniculatus*	2008	Male	Positive	Yes
339	VE4008	Miranda	Sucre	Filas de mariche	*P. geniculatus*	2008	Female	Positive	Yes
340	VE4808	Miranda	Sucre	Filas de mariche	*P. geniculatus*	2008	Female	Positive	Yes
341	VE53308	Miranda	Sucre	Filas de mariche	*P. geniculatus*	2008	Female	Positive	Yes
342	VE53408	Miranda	Sucre	Filas de mariche	*P. geniculatus*	2008	Nymph V	Positive	Yes
343	VE56008	Miranda	Sucre	Filas de mariche	*P. geniculatus*	2008	Female	Positive	Yes
344	VE19908	Miranda	Sucre	La Dolorita	*P. geniculatus*	2008	Female	Negative	No
345	VE33208A	Miranda	Sucre	La Dolorita	*P. geniculatus*	2008	Female	Positive	No
346	VE9808	Miranda	Sucre	La Dolorita	*P. geniculatus*	2008	Male	Positive	No
347	VE37308	Miranda	Sucre	Leoncio Martínez	*P. geniculatus*	2008	Male	Negative	No
348	VE43408	Miranda	Sucre	Leoncio Martínez	*P. geniculatus*	2008	Female	Negative	No
349	VE48908	Miranda	Sucre	Leoncio Martínez	*P. geniculatus*	2008	Male	Negative	No
350	VE51108	Miranda	Sucre	Leoncio Martínez	*P. geniculatus*	2008	Female	Negative	No
351	VE53608	Miranda	Sucre	Leoncio Martínez	*P. geniculatus*	2008	Female	Negative	No
352	VE3608	Miranda	Sucre	Leoncio Martínez	*P. geniculatus*	2008	Male	Negative	Yes
353	VE36608A	Miranda	Sucre	Leoncio Martínez	*P. geniculatus*	2008	Female	Negative	Yes
354	VE49308	Miranda	Sucre	Leoncio Martínez	*P. geniculatus*	2008	Female	Negative	Yes
355	VE51808	Miranda	Sucre	Leoncio Martínez	*P. geniculatus*	2008	Female	Negative	Yes
356	VE13708	Miranda	Sucre	Leoncio Martínez	*P. geniculatus*	2008	Female	Positive	No
357	VE15908	Miranda	Sucre	Leoncio Martínez	*P. geniculatus*	2008	Female	Positive	No
358	VE41808	Miranda	Sucre	Leoncio Martínez	*P. geniculatus*	2008	Male	Positive	No
359	VE47508	Miranda	Sucre	Leoncio Martínez	*P. geniculatus*	2008	Male	Positive	No
360	VE53908	Miranda	Sucre	Leoncio Martínez	*P. geniculatus*	2008	Male	Positive	No
361	VE56108	Miranda	Sucre	Leoncio Martínez	*P. geniculatus*	2008	Male	Positive	No
362	VE6508	Miranda	Sucre	Leoncio Martínez	*P. geniculatus*	2008	Male	Positive	No
363	VE18508	Miranda	Sucre	Leoncio Martínez	*P. geniculatus*	2008	Female	Positive	Yes
364	VE5108	Miranda	Sucre	Petare	*P. geniculatus*	2008	Female	Negative	No
365	VE0208(2)	Miranda	Sucre	Petare	*P. geniculatus*	2008	Male	Negative	No
366	VE0208(4)	Miranda	Sucre	Petare	*P. geniculatus*	2008	Male	Negative	No
367	VE17908A	Miranda	Sucre	Petare	*P. geniculatus*	2008	Male	Negative	No
368	VE17908B	Miranda	Sucre	Petare	*P. geniculatus*	2008	Male	Negative	No
369	VE17908C	Miranda	Sucre	Petare	*P. geniculatus*	2008	Female	Negative	No
370	VE17908D	Miranda	Sucre	Petare	*P. geniculatus*	2008	Female	Negative	No
371	VE25208A	Miranda	Sucre	Petare	*P. geniculatus*	2008	Male	Negative	No
372	VE32808	Miranda	Sucre	Petare	*P. geniculatus*	2008	Female	Negative	No
373	VE37508	Miranda	Sucre	Petare	*P. geniculatus*	2008	Female	Negative	No
374	VE39808	Miranda	Sucre	Petare	*P. geniculatus*	2008	Female	Negative	No
375	VE40708	Miranda	Sucre	Petare	*P. geniculatus*	2008	Female	Negative	No
376	VE42308	Miranda	Sucre	Petare	*P. geniculatus*	2008	Female	Negative	No

377	VE4408	Miranda	Sucre	Petare	*P. geniculatus*	2008	Male	Negative	No
378	VE46508	Miranda	Sucre	Petare	*P. geniculatus*	2008	Male	Negative	No
379	VE50708	Miranda	Sucre	Petare	*P. geniculatus*	2008	Female	Negative	No
380	VE52008	Miranda	Sucre	Petare	*P. geniculatus*	2008	Male	Negative	No
381	VE52308	Miranda	Sucre	Petare	*P. geniculatus*	2008	Male	Negative	No
382	VE55908	Miranda	Sucre	Petare	*P. geniculatus*	2008	Female	Negative	No
383	VE57508(A)	Miranda	Sucre	Petare	*P. geniculatus*	2008	Male	Negative	No
384	VE57508(B)	Miranda	Sucre	Petare	*P. geniculatus*	2008	Female	Negative	No
385	VE8908	Miranda	Sucre	Petare	*P. geniculatus*	2008	Male	Negative	No
386	VE25108	Miranda	Sucre	Petare	*P. geniculatus*	2008	Female	Negative	Yes
387	VE2608	Miranda	Sucre	Petare	*P. geniculatus*	2008	Female	Negative	Yes
388	VE28708	Miranda	Sucre	Petare	*P. geniculatus*	2008	Female	Negative	Yes
389	VE392A08	Miranda	Sucre	Petare	*P. geniculatus*	2008	Male	Negative	Yes
390	VE52708	Miranda	Sucre	Petare	*P. geniculatus*	2008	Female	Negative	Yes
391	VE54908	Miranda	Sucre	Petare	*P. geniculatus*	2008	Male	Negative	Yes
392	VE58308	Miranda	Sucre	Petare	*P. geniculatus*	2008	Female	Negative	Yes
393	VE6108	Miranda	Sucre	Petare	*P. geniculatus*	2008	Female	Negative	Yes
394	VE7108	Miranda	Sucre	Petare	*P. geniculatus*	2008	Female	Negative	Yes
395	VE59408	Miranda	Sucre	Petare	*P. geniculatus*	2008	Male	Negative	No
396	VE58008	Miranda	Sucre	Petare	*P. geniculatus*	2008	Female	Positive	No
397	VE59108	Miranda	Sucre	Petare	*P. geniculatus*	2008	Nymph IV	Positive	No
398	VE59208	Miranda	Sucre	Petare	*P. geniculatus*	2008	Nymph IV	Positive	Yes
399	VE0208(10)	Miranda	Sucre	Petare	*P. geniculatus*	2008	Female	Positive	No
400	VE0208(11)	Miranda	Sucre	Petare	*P. geniculatus*	2008	Female	Positive	No
401	VE0208(14)	Miranda	Sucre	Petare	*P. geniculatus*	2008	Male	Positive	No
402	VE0208(15)	Miranda	Sucre	Petare	*P. geniculatus*	2008	Male	Positive	No
403	VE0208(16)	Miranda	Sucre	Petare	*P. geniculatus*	2008	Male	Positive	No
404	VE0208(18)	Miranda	Sucre	Petare	*P. geniculatus*	2008	Male	Positive	No
405	VE0208(5)	Miranda	Sucre	Petare	*P. geniculatus*	2008	Female	Positive	No
406	VE0208(6)	Miranda	Sucre	Petare	*P. geniculatus*	2008	Female	Positive	No
407	VE0208(7)	Miranda	Sucre	Petare	*P. geniculatus*	2008	Female	Positive	No
408	VE0208(9)	Miranda	Sucre	Petare	*P. geniculatus*	2008	Female	Positive	No
409	VE1608A	Miranda	Sucre	Petare	*P. geniculatus*	2008	Male	Positive	No
410	VE1608B	Miranda	Sucre	Petare	*P. geniculatus*	2008	Male	Positive	No
411	VE18208	Miranda	Sucre	Petare	*P. geniculatus*	2008	Male	Positive	No
412	VE21808	Miranda	Sucre	Petare	*P. geniculatus*	2008	Female	Positive	No
413	VE2208	Miranda	Sucre	Petare	*P. geniculatus*	2008	Nymph III	Positive	No
414	VE22908	Miranda	Sucre	Petare	*P. geniculatus*	2008	Male	Positive	No
415	VE23408	Miranda	Sucre	Petare	*P. geniculatus*	2008	Female	Positive	No
416	VE25208A	Miranda	Sucre	Petare	*P. geniculatus*	2008	Female	Positive	No
417	VE25908	Miranda	Sucre	Petare	*P. geniculatus*	2008	Female	Positive	No
418	VE26308	Miranda	Sucre	Petare	*P. geniculatus*	2008	Male	Positive	No

419	VE27508	Miranda	Sucre	Petare	*P. geniculatus*	2008	Female	Positive	No
420	VE27608	Miranda	Sucre	Petare	*P. geniculatus*	2008	Male	Positive	No
421	VE30508	Miranda	Sucre	Petare	*P. geniculatus*	2008	Male	Positive	No
422	VE39908	Miranda	Sucre	Petare	*P. geniculatus*	2008	Male	Positive	No
423	VE40408	Miranda	Sucre	Petare	*P. geniculatus*	2008	Female	Positive	No
424	VE44208	Miranda	Sucre	Petare	*P. geniculatus*	2008	Male	Positive	No
425	VE4708	Miranda	Sucre	Petare	*P. geniculatus*	2008	Female	Positive	No
426	VE48008	Miranda	Sucre	Petare	*P. geniculatus*	2008	Female	Positive	No
427	VE49608	Miranda	Sucre	Petare	*P. geniculatus*	2008	Female	Positive	No
428	VE49908	Miranda	Sucre	Petare	*P. geniculatus*	2008	Male	Positive	No
429	VE50108	Miranda	Sucre	Petare	*P. geniculatus*	2008	Female	Positive	No
430	VE51908(B)	Miranda	Sucre	Petare	*P. geniculatus*	2008	Female	Positive	No
431	VE53708	Miranda	Sucre	Petare	*P. geniculatus*	2008	Male	Positive	No
432	VE54608	Miranda	Sucre	Petare	*P. geniculatus*	2008	Male	Positive	No
433	VE598508	Miranda	Sucre	Petare	*P. geniculatus*	2008	Male	Positive	No
434	VE21608	Miranda	Sucre	Petare	*P. geniculatus*	2008	Female	Positive	No
435	VE6408	Miranda	Sucre	Petare	*P. geniculatus*	2008	Male	Positive	No
436	VE54308	Miranda	Sucre	Petare	*P. geniculatus*	2008	Male	Positive	No
437	VE4308A	Miranda	Sucre	Petare	*P. geniculatus*	2008	Male	Positive	No
438	VE0208(1)	Miranda	Sucre	Petare	*P. geniculatus*	2008	Male	Positive	Yes
439	VE0208(12)	Miranda	Sucre	Petare	*P. geniculatus*	2008	Male	Positive	Yes
440	VE0208(3)	Miranda	Sucre	Petare	*P. geniculatus*	2008	Male	Positive	Yes
441	VE11408	Miranda	Sucre	Petare	*P. geniculatus*	2008	Female	Positive	Yes
442	VE11808	Miranda	Sucre	Petare	*P. geniculatus*	2008	Female	Positive	Yes
443	VE13108	Miranda	Sucre	Petare	*P. geniculatus*	2008	Female	Positive	Yes
444	VE13608A	Miranda	Sucre	Petare	*P. geniculatus*	2008	Female	Positive	Yes
445	VE13608B	Miranda	Sucre	Petare	*P. geniculatus*	2008	Male	Positive	Yes
446	VE13608C	Miranda	Sucre	Petare	*P. geniculatus*	2008	Female	Positive	Yes
447	VE14208	Miranda	Sucre	Petare	*P. geniculatus*	2008	Female	Positive	Yes
448	VE15408A	Miranda	Sucre	Petare	*P. geniculatus*	2008	Male	Positive	Yes
449	VE15408B	Miranda	Sucre	Petare	*P. geniculatus*	2008	Male	Positive	Yes
450	VE15408D	Miranda	Sucre	Petare	*P. geniculatus*	2008	Male	Positive	Yes
451	VE15408E	Miranda	Sucre	Petare	*P. geniculatus*	2008	Male	Positive	Yes
452	VE16008A	Miranda	Sucre	Petare	*P. geniculatus*	2008	Male	Positive	Yes
453	VE16008B	Miranda	Sucre	Petare	*P. geniculatus*	2008	Male	Positive	Yes
454	VE16408A	Miranda	Sucre	Petare	*P. geniculatus*	2008	Female	Positive	Yes
455	VE16408B	Miranda	Sucre	Petare	*P. geniculatus*	2008	Female	Positive	Yes
456	VE16408C	Miranda	Sucre	Petare	*P. geniculatus*	2008	Female	Positive	Yes
457	VE16508	Miranda	Sucre	Petare	*P. geniculatus*	2008	Male	Positive	Yes
458	VE16608	Miranda	Sucre	Petare	*P. geniculatus*	2008	Female	Positive	Yes
459	VE17908E	Miranda	Sucre	Petare	*P. geniculatus*	2008	Male	Positive	Yes
460	VE18608	Miranda	Sucre	Petare	*P. geniculatus*	2008	Female	Positive	Yes

461	VE20808	Miranda	Sucre	Petare	*P. geniculatus*	2008	Female	Positive	Yes
462	VE23808	Miranda	Sucre	Petare	*P. geniculatus*	2008	Female	Positive	Yes
463	VE24408	Miranda	Sucre	Petare	*P. geniculatus*	2008	Female	Positive	Yes
464	VE2808	Miranda	Sucre	Petare	*P. geniculatus*	2008	Female	Positive	Yes
465	VE38108	Miranda	Sucre	Petare	*P. geniculatus*	2008	Male	Positive	Yes
466	VE38408	Miranda	Sucre	Petare	*P. geniculatus*	2008	Female	Positive	Yes
467	VE42408	Miranda	Sucre	Petare	*P. geniculatus*	2008	Female	Positive	Yes
468	VE4308B	Miranda	Sucre	Petare	*P. geniculatus*	2008	Male	Positive	Yes
469	VE43908	Miranda	Sucre	Petare	*P. geniculatus*	2008	Female	Positive	Yes
470	VE44508	Miranda	Sucre	Petare	*P. geniculatus*	2008	Female	Positive	Yes
471	VE44808	Miranda	Sucre	Petare	*P. geniculatus*	2008	Male	Positive	Yes
472	VE46008	Miranda	Sucre	Petare	*P. geniculatus*	2008	Female	Positive	Yes
473	VE47908	Miranda	Sucre	Petare	*P. geniculatus*	2008	Male	Positive	Yes
474	VE5608	Miranda	Sucre	Petare	*P. geniculatus*	2008	Female	Positive	Yes
475	VE56508(A)	Miranda	Sucre	Petare	*P. geniculatus*	2008	Female	Positive	Yes
476	VE57608	Miranda	Sucre	Petare	*P. geniculatus*	2008	Female	Positive	Yes
477	VE57908	Miranda	Sucre	Petare	*P. geniculatus*	2008	Female	Positive	Yes
478	VE7908	Miranda	Sucre	Petare	*P. geniculatus*	2008	Female	Positive	Yes
479	VE37509	DC	Libertador	Altagracia	*P. geniculatus*	2009	Female	NA	No
480	VE25709	DC	Libertador	Altagracia	*P. geniculatus*	2009	Female	Positive	No
481	VE28609	DC	Libertador	Altagracia	*P. geniculatus*	2009	Female	Positive	No
482	VE22809	DC	Libertador	Altagracia	*P. geniculatus*	2009	Female	Positive	Yes
483	VE37009B	DC	Libertador	Altagracia	*P. geniculatus*	2009	Female	NA	No
484	VE37009A	DC	Libertador	Altagracia	*P. geniculatus*	2009	Male	NA	No
485	VE4909	DC	Libertador	Altagracia	*P. geniculatus*	2009	Male	Negative	Yes
486	VE62109b	DC	Libertador	Antímano	*P. geniculatus*	2009	Female	NA	NA
487	VE58109 A	DC	Libertador	Antímano	*P. geniculatus*	2009	Female	NA	NA
488	VE58109 B	DC	Libertador	Antímano	*P. geniculatus*	2009	Female	NA	NA
489	VE58109 C	DC	Libertador	Antímano	*P. geniculatus*	2009	Female	NA	NA
490	VE37609	DC	Libertador	Antímano	*P. geniculatus*	2009	Female	NA	No
491	VE53709	DC	Libertador	Antímano	*P. geniculatus*	2009	Female	NA	Yes
492	VE30809	DC	Libertador	Antímano	*P. geniculatus*	2009	Female	Negative	No
493	VE44909 A	DC	Libertador	Antímano	*P. geniculatus*	2009	Female	Negative	No
494	VE44909 B	DC	Libertador	Antímano	*P. geniculatus*	2009	Female	Negative	Yes
495	VE62109A	DC	Libertador	Antímano	*P. geniculatus*	2009	Female	Positive	No
496	VE63809	DC	Libertador	Antímano	*P. geniculatus*	2009	Female	Positive	No
497	VE27009	DC	Libertador	Antímano	*P. geniculatus*	2009	Female	Positive	No
498	VE13209B	DC	Libertador	Antímano	*P. geniculatus*	2009	Female	Positive	Yes
499	VE49409 A	DC	Libertador	Antímano	*P. geniculatus*	2009	Female	Positive	Yes
500	VE52409	DC	Libertador	Antímano	*P. geniculatus*	2009	Female	Positive	Yes
501	VE62009A	DC	Libertador	Antímano	*P. geniculatus*	2009	Female	Positive	Yes
502	VE50509	DC	Libertador	Antímano	*P. geniculatus*	2009	Female	Positive	Yes

503	VE53809	DC	Libertador	Antímano	*P. geniculatus*	2009	Female	Positive	Yes
504	VE58609	DC	Libertador	Antímano	*P. geniculatus*	2009	Female	Positive	Yes
505	VE58809	DC	Libertador	Antímano	*P. geniculatus*	2009	Female	Positive	Yes
506	VE13209A	DC	Libertador	Antímano	*P. geniculatus*	2009	Male	NA	NA
507	VE62109c	DC	Libertador	Antímano	*P. geniculatus*	2009	Male	NA	NA
508	VE49409 B	DC	Libertador	Antímano	*P. geniculatus*	2009	Male	NA	NA
509	VE49409 C	DC	Libertador	Antímano	*P. geniculatus*	2009	Male	NA	NA
510	VE49409 D	DC	Libertador	Antímano	*P. geniculatus*	2009	Male	NA	NA
511	VE49409 E	DC	Libertador	Antímano	*P. geniculatus*	2009	Male	NA	NA
512	VE49409 F	DC	Libertador	Antímano	*P. geniculatus*	2009	Male	NA	NA
513	VE49409 G	DC	Libertador	Antímano	*P. geniculatus*	2009	Male	NA	NA
514	VE58109 D	DC	Libertador	Antímano	*P. geniculatus*	2009	Male	NA	NA
515	VE58109 E	DC	Libertador	Antímano	*P. geniculatus*	2009	Male	NA	NA
516	VE58109 F	DC	Libertador	Antímano	*P. geniculatus*	2009	Male	NA	NA
517	VE44909 C	DC	Libertador	Antímano	*P. geniculatus*	2009	Male	NA	NA
518	VE63409	DC	Libertador	Antímano	*P. geniculatus*	2009	Male	NA	No
519	VE55609	DC	Libertador	Antímano	*P. geniculatus*	2009	Male	Negative	No
520	VE64609	DC	Libertador	Antímano	*P. geniculatus*	2009	Male	Negative	No
521	VE0909	DC	Libertador	Antímano	*P. geniculatus*	2009	Male	Negative	Yes
522	VE58209	DC	Libertador	Antímano	*P. geniculatus*	2009	Male	Negative	Yes
523	VE63309	DC	Libertador	Antímano	*P. geniculatus*	2009	Male	Positive	No
524	VE29509	DC	Libertador	Antímano	*P. geniculatus*	2009	Male	Positive	No
525	VE34809	DC	Libertador	Antímano	*P. geniculatus*	2009	Male	Positive	No
526	VE42909	DC	Libertador	Antímano	*P. geniculatus*	2009	Male	Positive	No
527	VE13209C	DC	Libertador	Antímano	*P. geniculatus*	2009	Male	Positive	Yes
528	VE58909	DC	Libertador	Antímano	*P. geniculatus*	2009	Male	Positive	Yes
529	VE45909	DC	Libertador	Antímano	*R. prolixus*	2009	Male	Positive	Yes
530	VE60009 A	DC	Libertador	Antímano	*P. geniculatus*	2009	Nymph IV	NA	Yes
531	VE44909 E	DC	Libertador	Antímano	*P. geniculatus*	2009	Nymph V	NA	NA
532	VE60409	DC	Libertador	Antímano	*P. geniculatus*	2009	Nymph V	NA	No
533	VE60009 B	DC	Libertador	Antímano	*P. geniculatus*	2009	Nymph V	NA	Yes
534	VE0409	DC	Libertador	Antímano	*P. geniculatus*	2009	Nymph V	Negative	Yes
535	VE44909 D	DC	Libertador	Antímano	*P. geniculatus*	2009	Nymph V	Positive	Yes
536	VE26309	DC	Libertador	Caricuao	*P. geniculatus*	2009	Female	NA	NA
537	VE56809	DC	Libertador	Caricuao	*P. geniculatus*	2009	Female	Negative	No
538	VE33109	DC	Libertador	Caricuao	*P. geniculatus*	2009	Female	Positive	No
539	VE3209	DC	Libertador	Caricuao	*P. geniculatus*	2009	Female	Positive	Yes
540	VE60809	DC	Libertador	Caricuao	*P. geniculatus*	2009	Male	NA	No
541	VE64709	DC	Libertador	Caricuao	*P. geniculatus*	2009	Male	Negative	No
542	VE31609	DC	Libertador	Caricuao	*P. geniculatus*	2009	Male	Negative	Yes
543	VE6309	DC	Libertador	Caricuao	*P. geniculatus*	2009	Male	Positive	Yes
544	VE49509	DC	Libertador	Coche	*P. geniculatus*	2009	Female	Positive	No

545	VE41109	DC	Libertador	Coche	*P. geniculatus*	2009	Male	NA	No
546	VE36309	DC	Libertador	Coche	*P. geniculatus*	2009	Male	Negative	No
547	VE28709	DC	Libertador	Coche	*P. geniculatus*	2009	Male	Positive	No
548	VE39109	DC	Libertador	Coche	*P. geniculatus*	2009	Male	Positive	No
549	VE53309	DC	Libertador	Coche	*P. geniculatus*	2009	Male	Positive	No
550	VE42709	DC	Libertador	El Junquito	*P. geniculatus*	2009	Female	Negative	Yes
551	VE30709	DC	Libertador	El Junquito	*P. geniculatus*	2009	Female	Negative	Yes
552	VE37909	DC	Libertador	El Junquito	*P. geniculatus*	2009	Female	Negative	Yes
553	VE57709	DC	Libertador	El Junquito	*P. geniculatus*	2009	Female	Negative	Yes
554	VE45409B	DC	Libertador	El Junquito	*T. maculata*	2009	Female	Negative	Yes
555	VE64409	DC	Libertador	El Junquito	*P. geniculatus*	2009	Female	Positive	No
556	VE30009	DC	Libertador	El Junquito	*P. geniculatus*	2009	Female	Positive	Yes
557	VE44509	DC	Libertador	El Junquito	*P. geniculatus*	2009	Female	Positive	Yes
558	VE49809	DC	Libertador	El Junquito	*P. geniculatus*	2009	Female	Positive	Yes
559	VE48309	DC	Libertador	El Junquito	*P. geniculatus*	2009	Female	Positive	Yes
560	VE29309A	DC	Libertador	El Junquito	*P. geniculatus*	2009	Female	Positive	No
561	VE23209	DC	Libertador	El Junquito	*P. geniculatus*	2009	Male	Negative	No
562	VE27809	DC	Libertador	El Junquito	*P. geniculatus*	2009	Male	Negative	No
563	VE46609	DC	Libertador	El Junquito	*P. geniculatus*	2009	Male	Negative	No
564	VE39209	DC	Libertador	El Junquito	*P. geniculatus*	2009	Male	Negative	Yes
565	VE29309B	DC	Libertador	El Junquito	*P. geniculatus*	2009	Male	Positive	No
566	VE52109	DC	Libertador	El Junquito	*P. geniculatus*	2009	Male	Positive	Yes
567	VE42809	DC	Libertador	El Junquito	*P. geniculatus*	2009	Nymph III	NA	No
568	VE13509 B	DC	Libertador	El Paraiso	*P. geniculatus*	2009	Female	Positive	No
569	VE13509 A	DC	Libertador	El Paraiso	*P. geniculatus*	2009	Female	Positive	Yes
570	VE46009B	DC	Libertador	El Paraiso	*P. geniculatus*	2009	Male	Positive	No
571	VE48209 A	DC	Libertador	El Recreo	*P. geniculatus*	2009	Female	NA	No
572	VE48209 B	DC	Libertador	El Recreo	*P. geniculatus*	2009	Male	NA	No
573	VE59309	DC	Libertador	El Recreo	*P. geniculatus*	2009	Male	NA	No
574	VE6809	DC	Libertador	El Recreo	*P. geniculatus*	2009	Male	Negative	No
575	VE28809	DC	Libertador	El Recreo	*P. geniculatus*	2009	Male	Positive	No
576	VE22609	DC	Libertador	El Recreo	*P. geniculatus*	2009	Male	Positive	Yes
577	VE46109	DC	Libertador	El Recreo	*P. geniculatus*	2009	Male	Positive	Yes
578	VE11809	DC	Libertador	El valle	*P. geniculatus*	2009	Male	Negative	No
579	VE18409	DC	Libertador	La Pastora	*P. geniculatus*	2009	Female	NA	NA
580	VE64909	DC	Libertador	La Pastora	*P. geniculatus*	2009	Female	NA	No
581	VE17609	DC	Libertador	La Pastora	*P. geniculatus*	2009	Female	Negative	No
582	VE27709	DC	Libertador	La Pastora	*P. geniculatus*	2009	Female	Negative	No
583	VE30609	DC	Libertador	La Pastora	*P. geniculatus*	2009	Female	Negative	No
584	VE5209	DC	Libertador	La Pastora	*P. geniculatus*	2009	Female	Negative	Yes
585	VE28209	DC	Libertador	La Pastora	*P. geniculatus*	2009	Female	Negative	Yes
586	VE34209A	DC	Libertador	La Pastora	*P. geniculatus*	2009	Female	Positive	No

587	VE1009	DC	Libertador	La Pastora	*P. geniculatus*	2009	Female	Positive	No
588	VE8309	DC	Libertador	La Pastora	*P. geniculatus*	2009	Female	Positive	Yes
589	VE55909	DC	Libertador	La Pastora	*P. geniculatus*	2009	Female	Positive	Yes
590	VE14609	DC	Libertador	La Pastora	*P. geniculatus*	2009	Female	Positive	Yes
591	VE19509A	DC	Libertador	La Pastora	*P. geniculatus*	2009	Female	Positive	Yes
592	VE2409	DC	Libertador	La Pastora	*P. geniculatus*	2009	Female	Positive	Yes
593	VE34409	DC	Libertador	La Pastora	*P. geniculatus*	2009	Female	Positive	Yes
594	VE5909 a	DC	Libertador	La Pastora	*P. geniculatus*	2009	Female	Positive	Yes
595	VE46309	DC	Libertador	La Pastora	*P. geniculatus*	2009	Female	Positive	Yes
596	VE5909 b	DC	Libertador	La Pastora	*P. geniculatus*	2009	Male	Positive	No
597	VE19509B	DC	Libertador	La Pastora	*P. geniculatus*	2009	Male	Positive	Yes
598	VE34309A	DC	Libertador	La Pastora	*P. geniculatus*	2009	Male	NA	Yes
599	VE7609	DC	Libertador	La Pastora	*P. geniculatus*	2009	Male	Negative	No
600	VE43709	DC	Libertador	La Pastora	*P. geniculatus*	2009	Male	Negative	No
601	VE35109	DC	Libertador	La Pastora	*P. geniculatus*	2009	Male	Positive	No
602	VE14809	DC	Libertador	La Pastora	*P. geniculatus*	2009	Male	Positive	No
603	VE50709	DC	Libertador	La Pastora	*P. geniculatus*	2009	Male	Positive	No
604	VE53909	DC	Libertador	La Pastora	*P. geniculatus*	2009	Male	Positive	No
605	VE63609	DC	Libertador	La Pastora	*P. geniculatus*	2009	Male	Positive	No
606	VE49709	DC	Libertador	La Pastora	*P. geniculatus*	2009	Male	Positive	Yes
607	VE34209B	DC	Libertador	La Pastora	*P. geniculatus*	2009	Male	Positive	Yes
608	VE63209	DC	Libertador	La Pastora	*P. geniculatus*	2009	Nymph IV	NA	Yes
609	VE36909	DC	Libertador	La Vega	*P. geniculatus*	2009	Female	Negative	No
610	VE61709	DC	Libertador	La Vega	*P. geniculatus*	2009	Female	Positive	No
611	VE43209B	DC	Libertador	La Vega	*P. geniculatus*	2009	Female	Positive	Yes
612	VE36009	DC	Libertador	La Vega	*P. geniculatus*	2009	Male	Positive	No
613	VE38709A	DC	Libertador	Macarao	*P. geniculatus*	2009	Female	NA	NA
614	VE38709B	DC	Libertador	Macarao	*P. geniculatus*	2009	Female	NA	NA
615	VE9009A	DC	Libertador	San Bernardino	*P. geniculatus*	2009	Female	NA	NA
616	VE9009B	DC	Libertador	San Bernardino	*P. geniculatus*	2009	Female	NA	NA
617	VE16409	DC	Libertador	San Bernardino	*P. geniculatus*	2009	Female	Positive	No
618	VE51109	DC	Libertador	San Bernardino	*P. geniculatus*	2009	Female	Positive	No
619	VE31709	DC	Libertador	San Bernardino	*P. geniculatus*	2009	Male	Negative	Yes
620	VE11709	DC	Libertador	San Bernardino	*P. geniculatus*	2009	Male	Negative	Yes
621	VE40309B	DC	Libertador	San Bernardino	*P. geniculatus*	2009	Male	Positive	No
622	VE29709A	DC	Libertador	San José	*P. geniculatus*	2009	Female	NA	NA
623	VE29709B	DC	Libertador	San José	*P. geniculatus*	2009	Female	NA	NA
624	VE29709C	DC	Libertador	San José	*P. geniculatus*	2009	Female	NA	NA
625	VE36509	DC	Libertador	San José	*P. geniculatus*	2009	Female	NA	No
626	VE4009	DC	Libertador	San José	*P. geniculatus*	2009	Female	NA	No
627	VE6109	DC	Libertador	San José	*P. geniculatus*	2009	Female	Negative	Yes
628	VE24909	DC	Libertador	San José	*P. geniculatus*	2009	Female	Negative	Yes

629	VE11609	DC	Libertador	San José	*P. geniculatus*	2009	Female	Negative	Yes
630	VE2609	DC	Libertador	San José	*P. geniculatus*	2009	Female	Positive	No
631	VE49609	DC	Libertador	San José	*P. geniculatus*	2009	Female	Positive	Yes
632	VE48609	DC	Libertador	San José	*P. geniculatus*	2009	Female	Positive	Yes
633	VE16909	DC	Libertador	San José	*P. geniculatus*	2009	Male	Negative	No
634	VE23409	DC	Libertador	San José	*P. geniculatus*	2009	Male	Positive	No
635	VE29809	DC	Libertador	San José	*P. geniculatus*	2009	Male	Positive	No
636	VE16009	DC	Libertador	San Juan	*P. geniculatus*	2009	Male	Negative	Yes
637	VE57509	DC	Libertador	San Juan	*P. geniculatus*	2009	Male	NA	NA
638	VE5509	DC	Libertador	San Pedro	*P. geniculatus*	2009	Male	Negative	No
639	VE18109	DC	Libertador	San Pedro	*P. geniculatus*	2009	Male	Positive	Yes
640	VE40609	DC	Libertador	Santa Rosalía	*P. geniculatus*	2009	Female	NA	No
641	VE60509	DC	Libertador	Santa Teresa	*P. geniculatus*	2009	Female	Negative	No
642	VE63109	DC	Libertador	Santa Teresa	*P. geniculatus*	2009	Female	Negative	Yes
643	VE36609	DC	Libertador	Santa Teresa	*P. geniculatus*	2009	Male	Positive	No
644	VE12909A	DC	Libertador	Sucre	*P. geniculatus*	2009	Female	NA	NA
645	VE13709B	DC	Libertador	Sucre	*P. geniculatus*	2009	Female	NA	NA
646	VE13709C	DC	Libertador	Sucre	*P. geniculatus*	2009	Female	NA	NA
647	VE17809A	DC	Libertador	Sucre	*P. geniculatus*	2009	Female	NA	NA
648	VE21209A	DC	Libertador	Sucre	*P. geniculatus*	2009	Female	NA	NA
649	VE61309	DC	Libertador	Sucre	*P. geniculatus*	2009	Female	NA	No
650	VE3109	DC	Libertador	Sucre	*P. geniculatus*	2009	Female	NA	No
651	VE45609	DC	Libertador	Sucre	*P. geniculatus*	2009	Female	NA	No
652	VE53009	DC	Libertador	Sucre	*P. geniculatus*	2009	Female	NA	No
653	VE27409	DC	Libertador	Sucre	*P. geniculatus*	2009	Female	NA	Yes
654	VE0109	DC	Libertador	Sucre	*P. geniculatus*	2009	Female	Negative	No
655	VE1109	DC	Libertador	Sucre	*P. geniculatus*	2009	Female	Negative	No
656	VE22009D	DC	Libertador	Sucre	*P. geniculatus*	2009	Female	Negative	Yes
657	VE14509	DC	Libertador	Sucre	*P. geniculatus*	2009	Female	Negative	Yes
658	VE35209	DC	Libertador	Sucre	*P. geniculatus*	2009	Female	Negative	Yes
659	VE0709	DC	Libertador	Sucre	*P. geniculatus*	2009	Female	Negative	Yes
660	VE4509	DC	Libertador	Sucre	*P. geniculatus*	2009	Female	Negative	Yes
661	VE52809	DC	Libertador	Sucre	*P. geniculatus*	2009	Female	Negative	Yes
662	VE5609	DC	Libertador	Sucre	*P. geniculatus*	2009	Female	Positive	No
663	VE17909A	DC	Libertador	Sucre	*P. geniculatus*	2009	Female	Positive	No
664	VE23609	DC	Libertador	Sucre	*P. geniculatus*	2009	Female	Positive	No
665	VE43609	DC	Libertador	Sucre	*P. geniculatus*	2009	Female	Positive	No
666	VE55309	DC	Libertador	Sucre	*P. geniculatus*	2009	Female	Positive	No
667	VE15209	DC	Libertador	Sucre	*P. geniculatus*	2009	Female	Positive	No
668	VE40209	DC	Libertador	Sucre	*P. geniculatus*	2009	Female	Positive	No
669	VE22009C	DC	Libertador	Sucre	*P. geniculatus*	2009	Female	Positive	Yes
670	VE22509	DC	Libertador	Sucre	*P. geniculatus*	2009	Female	Positive	Yes

671	VE35909 A	DC	Libertador	Sucre	*P. geniculatus*	2009	Female	Positive	Yes
672	VE39409B	DC	Libertador	Sucre	*P. geniculatus*	2009	Female	Positive	Yes
673	VE44709 A	DC	Libertador	Sucre	*P. geniculatus*	2009	Female	Positive	Yes
674	VE44709 B	DC	Libertador	Sucre	*P. geniculatus*	2009	Female	Positive	Yes
675	VE45209	DC	Libertador	Sucre	*P. geniculatus*	2009	Female	Positive	Yes
676	VE45709	DC	Libertador	Sucre	*P. geniculatus*	2009	Female	Positive	Yes
677	VE48509B	DC	Libertador	Sucre	*P. geniculatus*	2009	Female	Positive	Yes
678	VE13709A	DC	Libertador	Sucre	*P. geniculatus*	2009	Female	Positive	Yes
679	VE17309A	DC	Libertador	Sucre	*P. geniculatus*	2009	Female	Positive	Yes
680	VE2209	DC	Libertador	Sucre	*P. geniculatus*	2009	Female	Positive	Yes
681	VE24509	DC	Libertador	Sucre	*P. geniculatus*	2009	Female	Positive	Yes
682	VE28309	DC	Libertador	Sucre	*P. geniculatus*	2009	Female	Positive	Yes
683	VE40809	DC	Libertador	Sucre	*P. geniculatus*	2009	Female	Positive	Yes
684	VE42509	DC	Libertador	Sucre	*P. geniculatus*	2009	Female	Positive	Yes
685	VE44309	DC	Libertador	Sucre	*P. geniculatus*	2009	Female	Positive	Yes
686	VE46709	DC	Libertador	Sucre	*P. geniculatus*	2009	Female	Positive	Yes
687	VE59209	DC	Libertador	Sucre	*P. geniculatus*	2009	Female	Positive	Yes
688	VE62709B	DC	Libertador	Sucre	*P. geniculatus*	2009	Female	Positive	Yes
689	VE40009A	DC	Libertador	Sucre	*P. geniculatus*	2009	Female	Positive	Yes
690	VE36109	DC	Libertador	Sucre	*P. geniculatus*	2009	Female	NA	Yes
691	VE49309 A	DC	Libertador	Sucre	*P. geniculatus*	2009	Female	Negative	No
692	VE22009B	DC	Libertador	Sucre	*P. geniculatus*	2009	Male	NA	NA
693	VE12209A	DC	Libertador	Sucre	*P. geniculatus*	2009	Male	NA	NA
694	VE12209B	DC	Libertador	Sucre	*P. geniculatus*	2009	Male	NA	NA
695	VE49309 B	DC	Libertador	Sucre	*P. geniculatus*	2009	Male	NA	NA
696	VE12909B	DC	Libertador	Sucre	*P. geniculatus*	2009	Male	NA	NA
697	VE9209	DC	Libertador	Sucre	*P. geniculatus*	2009	Male	NA	NA
698	VE23309	DC	Libertador	Sucre	*P. geniculatus*	2009	Male	NA	No
699	VE3109	DC	Libertador	Sucre	*P. geniculatus*	2009	Male	NA	No
700	VE33809	DC	Libertador	Sucre	*P. geniculatus*	2009	Male	NA	No
701	VE40509	DC	Libertador	Sucre	*P. geniculatus*	2009	Male	NA	No
702	VE5409	DC	Libertador	Sucre	*P. geniculatus*	2009	Male	NA	No
703	VE21709	DC	Libertador	Sucre	*P. geniculatus*	2009	Male	NA	No
704	VE61409B	DC	Libertador	Sucre	*P. geniculatus*	2009	Male	NA	No
705	VE3809	DC	Libertador	Sucre	*P. geniculatus*	2009	Male	NA	NA
706	VE21909	DC	Libertador	Sucre	*P. geniculatus*	2009	Male	Negative	No
707	VE10009	DC	Libertador	Sucre	*P. geniculatus*	2009	Male	Negative	No
708	VE10509	DC	Libertador	Sucre	*P. geniculatus*	2009	Male	Negative	No
709	VE12709	DC	Libertador	Sucre	*P. geniculatus*	2009	Male	Negative	No
710	VE13809	DC	Libertador	Sucre	*P. geniculatus*	2009	Male	Negative	No
711	VE26509	DC	Libertador	Sucre	*P. geniculatus*	2009	Male	Negative	No
712	VE6909	DC	Libertador	Sucre	*P. geniculatus*	2009	Male	Negative	No

713	VE47509	DC	Libertador	Sucre	*P. geniculatus*	2009	Male	Negative	No
714	VE61909	DC	Libertador	Sucre	*P. geniculatus*	2009	Male	Negative	No
715	VE62409	DC	Libertador	Sucre	*P. geniculatus*	2009	Male	Negative	No
716	VE14009	DC	Libertador	Sucre	*P. geniculatus*	2009	Male	Negative	Yes
717	VE40409A	DC	Libertador	Sucre	*P. geniculatus*	2009	Male	Negative	Yes
718	VE25609	DC	Libertador	Sucre	*P. geniculatus*	2009	Male	Negative	Yes
719	VE11509	DC	Libertador	Sucre	*P. geniculatus*	2009	Male	Negative	Yes
720	VE39709	DC	Libertador	Sucre	*P. geniculatus*	2009	Male	Positive	No
721	VE9809	DC	Libertador	Sucre	*P. geniculatus*	2009	Male	Positive	No
722	VE16509	DC	Libertador	Sucre	*P. geniculatus*	2009	Male	Positive	No
723	VE17909B	DC	Libertador	Sucre	*P. geniculatus*	2009	Male	Positive	No
724	VE21109	DC	Libertador	Sucre	*P. geniculatus*	2009	Male	Positive	No
725	VE23009	DC	Libertador	Sucre	*P. geniculatus*	2009	Male	Positive	No
726	VE33009	DC	Libertador	Sucre	*P. geniculatus*	2009	Male	Positive	No
727	VE35909 B	DC	Libertador	Sucre	*P. geniculatus*	2009	Male	Positive	No
728	VE36209	DC	Libertador	Sucre	*P. geniculatus*	2009	Male	Positive	No
729	VE58409	DC	Libertador	Sucre	*P. geniculatus*	2009	Male	Positive	No
730	VE10409	DC	Libertador	Sucre	*P. geniculatus*	2009	Male	Positive	No
731	VE47609	DC	Libertador	Sucre	*P. geniculatus*	2009	Male	Positive	No
732	VE5109	DC	Libertador	Sucre	*P. geniculatus*	2009	Male	Positive	No
733	VE52209	DC	Libertador	Sucre	*P. geniculatus*	2009	Male	Positive	No
734	VE54609	DC	Libertador	Sucre	*P. geniculatus*	2009	Male	Positive	No
735	VE22109	DC	Libertador	Sucre	*P. geniculatus*	2009	Male	Positive	Yes
736	VE19709	DC	Libertador	Sucre	*P. geniculatus*	2009	Male	Positive	Yes
737	VE28009	DC	Libertador	Sucre	*P. geniculatus*	2009	Male	Positive	Yes
738	VE10309	DC	Libertador	Sucre	*P. geniculatus*	2009	Male	Positive	Yes
739	VE44809	DC	Libertador	Sucre	*P. geniculatus*	2009	Male	Positive	Yes
740	VE46509	DC	Libertador	Sucre	*P. geniculatus*	2009	Male	Positive	Yes
741	VE22009A	DC	Libertador	Sucre	*P. geniculatus*	2009	Nymph III	Positive	Yes
742	VE50309	DC	Libertador	Sucre	*P. geniculatus*	2009	Nymph III	Positive	Yes
743	VE17309B	DC	Libertador	Sucre	*P. geniculatus*	2009	Nymph IV	NA	NA
744	VE56409	DC	Libertador	Sucre	*P. geniculatus*	2009	Nymph IV	NA	Yes
745	VE11409	DC	Libertador	Sucre	*P. geniculatus*	2009	Nymph IV	Negative	Yes
746	VE1709	DC	Libertador	Sucre	*P. geniculatus*	2009	Nymph IV	Negative	No
747	VE58709	DC	Libertador	Sucre	*P. geniculatus*	2009	Nymph IV	Positive	Yes
748	VE65009	DC	Libertador	Sucre	*P. geniculatus*	2009	Nymph IV	Positive	Yes
749	VE7909	DC	Libertador	Sucre	*P. geniculatus*	2009	Nymph IV	Positive	Yes
750	VE4209	Miranda	Sucre	Caucagüita	*P. geniculatus*	2009	Female	NA	No
751	VE54509	Miranda	Sucre	Caucagüita	*P. geniculatus*	2009	Female	NA	No
752	VE26609	Miranda	Sucre	Caucagüita	*P. geniculatus*	2009	Female	NA	No
753	VE44009	Miranda	Sucre	Caucagüita	*P. geniculatus*	2009	Female	NA	Yes
754	VE63709	Miranda	Sucre	Caucagüita	*P. geniculatus*	2009	Female	NA	Yes

755	VE30109	Miranda	Sucre	Caucagüita	*P. geniculatus*	2009	Female	Negative	No
756	VE21309C	Miranda	Sucre	Caucagüita	*P. geniculatus*	2009	Female	Positive	No
757	VE28409	Miranda	Sucre	Caucagüita	*P. geniculatus*	2009	Female	Positive	No
758	VE8809	Miranda	Sucre	Caucagüita	*P. geniculatus*	2009	Female	Positive	No
759	VE44109	Miranda	Sucre	Caucagüita	*P. geniculatus*	2009	Female	Positive	No
760	VE15509A	Miranda	Sucre	Caucagüita	*P. geniculatus*	2009	Female	Positive	Yes
761	VE21309A	Miranda	Sucre	Caucagüita	*P. geniculatus*	2009	Female	Positive	Yes
762	VE21309B	Miranda	Sucre	Caucagüita	*P. geniculatus*	2009	Female	Positive	Yes
763	VE14109B	Miranda	Sucre	Caucagüita	*T. nigromaculata*	2009	Female	Positive	Yes
764	VE10609	Miranda	Sucre	Caucagüita	*P. geniculatus*	2009	Male	Negative	No
765	VE17509	Miranda	Sucre	Caucagüita	*P. geniculatus*	2009	Male	Positive	Yes
766	VE31409	Miranda	Sucre	Caucagüita	*P. geniculatus*	2009	Male	NA	Yes
767	VE2309	Miranda	Sucre	Caucagüita	*P. geniculatus*	2009	Male	Negative	No
768	VE26809	Miranda	Sucre	Caucagüita	*P. geniculatus*	2009	Male	Negative	Yes
769	VE61109	Miranda	Sucre	Caucagüita	*P. geniculatus*	2009	Male	Positive	No
770	VE23909	Miranda	Sucre	Caucagüita	*P. geniculatus*	2009	Male	Positive	No
771	VE19309	Miranda	Sucre	Caucagüita	*P. geniculatus*	2009	Male	Positive	Yes
772	VE15609	Miranda	Chacao	Chacao	*P. geniculatus*	2009	Female	Positive	No
773	VE25309	Miranda	Chacao	Chacao	*P. geniculatus*	2009	Male	NA	No
774	VE24709	Miranda	Chacao	Chacao	*R. prolixus*	2009	Male	Negative	No
775	VE8909	Miranda	Baruta	El Cafetal	*P. geniculatus*	2009	Female	NA	No
776	VE11009	Miranda	Baruta	El Cafetal	*P. geniculatus*	2009	Female	NA	No
777	VE41709	Miranda	Baruta	El Cafetal	*P. geniculatus*	2009	Female	NA	No
778	VE33509B	Miranda	Baruta	El Cafetal	*P. geniculatus*	2009	Female	Negative	No
779	VE45409A	Miranda	Baruta	El Cafetal	*P. geniculatus*	2009	Female	Negative	No
780	VE34009	Miranda	Baruta	El Cafetal	*P. geniculatus*	2009	Female	Negative	Yes
781	VE54209	Miranda	Baruta	El Cafetal	*P. geniculatus*	2009	Female	Positive	No
782	VE28909	Miranda	Baruta	El Cafetal	*P. geniculatus*	2009	Female	Positive	No
783	VE43009	Miranda	Baruta	El Cafetal	*P. geniculatus*	2009	Female	Positive	No
784	VE64209	Miranda	Baruta	El Cafetal	*P. geniculatus*	2009	Female	Positive	No
785	VE56309	Miranda	Baruta	El Cafetal	*P. geniculatus*	2009	Female	Positive	Yes
786	VE2509	Miranda	Baruta	El Cafetal	*P. geniculatus*	2009	Female	Positive	Yes
787	VE35809A	Miranda	Baruta	El Cafetal	*P. geniculatus*	2009	Female	Positive	Yes
788	VE50109	Miranda	Baruta	El Cafetal	*P. geniculatus*	2009	Female	Positive	Yes
789	VE7309	Miranda	Baruta	El Cafetal	*P. geniculatus*	2009	Female	Positive	Yes
790	VE3709	Miranda	Baruta	El Cafetal	*P. geniculatus*	2009	Female	Positive	Yes
791	VE14709	Miranda	Baruta	El Cafetal	*P. geniculatus*	2009	Male	NA	NA
792	VE35809B	Miranda	Baruta	El Cafetal	*P. geniculatus*	2009	Male	NA	Yes
793	VE42109B	Miranda	Baruta	El Cafetal	*P. geniculatus*	2009	Male	Negative	No
794	VE16309	Miranda	Baruta	El Cafetal	*P. geniculatus*	2009	Male	Negative	No
795	VE13609	Miranda	Baruta	El Cafetal	*P. geniculatus*	2009	Male	Negative	Yes
796	VE25209	Miranda	Baruta	El Cafetal	*P. geniculatus*	2009	Male	Positive	No

797	VE51509	Miranda	Baruta	El Cafetal	*P. geniculatus*	2009	Male	Positive	No
798	VE61809	Miranda	Baruta	El Cafetal	*P. geniculatus*	2009	Male	Positive	No
799	VE40109	Miranda	Baruta	El Cafetal	*P. geniculatus*	2009	Male	Positive	No
800	VE45309A	Miranda	Baruta	El Cafetal	*P. geniculatus*	2009	Male	Positive	No
801	VE44609	Miranda	Baruta	El Cafetal	*P. geniculatus*	2009	Male	Positive	Yes
802	VE51909	Miranda	Baruta	El Cafetal	*P. geniculatus*	2009	Male	Positive	Yes
803	VE20909	Miranda	Baruta	El Cafetal	*P. geniculatus*	2009	Nymph IV	NA	No
804	VE54309	Miranda	Sucre	Filas de mariche	*P. geniculatus*	2009	Female	NA	No
805	VE38309A	Miranda	Sucre	Filas de mariche	*P. geniculatus*	2009	Female	NA	No
806	VE43309	Miranda	Sucre	Filas de mariche	*P. geniculatus*	2009	Female	NA	No
807	VE52309	Miranda	Sucre	Filas de mariche	*P. geniculatus*	2009	Female	NA	Yes
808	VE54409	Miranda	Sucre	Filas de mariche	*P. geniculatus*	2009	Female	NA	Yes
809	VE64309	Miranda	Sucre	Filas de mariche	*P. geniculatus*	2009	Female	NA	Yes
810	VE7509B	Miranda	Sucre	Filas de mariche	*P. geniculatus*	2009	Female	NA	Yes
811	VE19909	Miranda	Sucre	Filas de mariche	*P. geniculatus*	2009	Female	Negative	No
812	VE38009	Miranda	Sucre	Filas de mariche	*P. geniculatus*	2009	Female	Negative	No
813	VE20109B	Miranda	Sucre	Filas de mariche	*P. geniculatus*	2009	Female	Negative	Yes
814	VE20109C	Miranda	Sucre	Filas de mariche	*P. geniculatus*	2009	Female	Negative	Yes
815	VE25809	Miranda	Sucre	Filas de mariche	*P. geniculatus*	2009	Female	Negative	Yes
816	VE32809	Miranda	Sucre	Filas de mariche	*P. geniculatus*	2009	Female	Negative	Yes
817	VE53609	Miranda	Sucre	Filas de mariche	*P. geniculatus*	2009	Female	Negative	Yes
818	VE8109	Miranda	Sucre	Filas de mariche	*P. geniculatus*	2009	Female	Negative	Yes
819	VE53409	Miranda	Sucre	Filas de mariche	*P. geniculatus*	2009	Female	Positive	Yes
820	VE3309	Miranda	Sucre	Filas de mariche	*P. geniculatus*	2009	Female	Positive	Yes
821	VE56909	Miranda	Sucre	Filas de mariche	*P. geniculatus*	2009	Female	Positive	Yes
822	VE62309	Miranda	Sucre	Filas de mariche	*P. geniculatus*	2009	Female	Positive	Yes
823	VE7809	Miranda	Sucre	Filas de mariche	*P. geniculatus*	2009	Female	Positive	Yes
824	VE61209A	Miranda	Sucre	Filas de mariche	*P. geniculatus*	2009	Female	NA	Yes
825	VE20109A	Miranda	Sucre	Filas de mariche	*P. geniculatus*	2009	Male	Positive	Yes
826	VE40709	Miranda	Sucre	Filas de mariche	*P. geniculatus*	2009	Male	NA	No
827	VE46409	Miranda	Sucre	Filas de mariche	*P. geniculatus*	2009	Male	NA	No
828	VE38309B	Miranda	Sucre	Filas de mariche	*P. geniculatus*	2009	Male	NA	No
829	VE54709	Miranda	Sucre	Filas de mariche	*P. geniculatus*	2009	Male	NA	No
830	VE61209B	Miranda	Sucre	Filas de mariche	*P. geniculatus*	2009	Male	NA	Yes
831	VE3609A	Miranda	Sucre	Filas de mariche	*P. geniculatus*	2009	Male	Negative	Yes
832	VE3609B	Miranda	Sucre	Filas de mariche	*P. geniculatus*	2009	Male	Negative	Yes
833	VE14409	Miranda	Sucre	Filas de mariche	*P. geniculatus*	2009	Male	Positive	No
834	VE26209	Miranda	Sucre	Filas de mariche	*P. geniculatus*	2009	Male	Positive	No
835	VE24409B	Miranda	Sucre	Filas de mariche	*P. geniculatus*	2009	Male	Positive	Yes
836	VE35709	Miranda	Sucre	Filas de mariche	*P. geniculatus*	2009	Male	Positive	Yes
837	VE1609	Miranda	Sucre	Filas de mariche	*P. geniculatus*	2009	Male	Positive	Yes
838	VE2009	Miranda	Sucre	Filas de mariche	*P. geniculatus*	2009	Male	Positive	Yes

839	VE21009	Miranda	Sucre	Filas de mariche	*P. geniculatus*	2009	Nymph III	Positive	Yes
840	VE29009	Miranda	Sucre	Filas de mariche	*P. geniculatus*	2009	Nymph IV	Positive	Yes
841	VE62609A	Miranda	Sucre	Filas de mariche	*P. geniculatus*	2009	Nymph V	Negative	Yes
842	VE0509	Miranda	Sucre	Filas de mariche	*P. geniculatus*	2009	Nymph V	Positive	No
843	VE18509	Miranda	Sucre	La Dolorita	*P. geniculatus*	2009	Female	NA	NA
844	VE43109A	Miranda	Sucre	La Dolorita	*P. geniculatus*	2009	Female	NA	No
845	VE52709	Miranda	Sucre	La Dolorita	*P. geniculatus*	2009	Female	NA	No
846	VE43109B	Miranda	Sucre	La Dolorita	*P. geniculatus*	2009	Female	Negative	No
847	VE26409A	Miranda	Sucre	La Dolorita	*P. geniculatus*	2009	Female	Negative	No
848	VE34209B	Miranda	Sucre	La Dolorita	*P. geniculatus*	2009	Female	Positive	Yes
849	VE34909	Miranda	Sucre	La Dolorita	*P. geniculatus*	2009	Female	Positive	Yes
850	VE57609	Miranda	Sucre	La Dolorita	*P. geniculatus*	2009	Female	Positive	Yes
851	VE10209	Miranda	Sucre	La Dolorita	*P. geniculatus*	2009	Male	NA	No
852	VE20709	Miranda	Sucre	La Dolorita	*P. geniculatus*	2009	Male	NA	No
853	VE25509	Miranda	Sucre	La Dolorita	*P. geniculatus*	2009	Male	Negative	No
854	VE0609	Miranda	Sucre	La Dolorita	*P. geniculatus*	2009	Male	Positive	No
855	VE19009	Miranda	Baruta	Las Minas de Baruta	*P. geniculatus*	2009	Female	NA	No
856	VE6609	Miranda	Baruta	Las Minas de Baruta	*P. geniculatus*	2009	Female	Negative	No
857	VE13309	Miranda	Baruta	Las Minas de Baruta	*P. geniculatus*	2009	Female	Positive	No
858	VE38509	Miranda	Baruta	Las Minas de Baruta	*P. geniculatus*	2009	Female	Positive	No
859	VE17809B	Miranda	Baruta	Las Minas de Baruta	*P. geniculatus*	2009	Female	Positive	Yes
860	VE19009	Miranda	Baruta	Las Minas de Baruta	*P. geniculatus*	2009	Male	NA	No
861	VE16209	Miranda	Baruta	Las Minas de Baruta	*P. geniculatus*	2009	Male	Positive	No
862	VE39809	Miranda	Baruta	Las Minas de Baruta	*P. geniculatus*	2009	Male	Negative	No
863	VE2809	Miranda	Baruta	Las Minas de Baruta	*P. geniculatus*	2009	Male	Negative	No
864	VE65709	Miranda	Baruta	Las Minas de Baruta	*P. geniculatus*	2009	Male	Positive	No
865	VE28509	Miranda	Baruta	Las Minas de Baruta	*P. geniculatus*	2009	Nymph III	Positive	No
866	VE11109	Miranda	Sucre	Leoncio Martínez	*P. geniculatus*	2009	Female	NA	No
867	VE22409	Miranda	Sucre	Leoncio Martínez	*P. geniculatus*	2009	Female	Positive	No
868	VE45009	Miranda	Sucre	Leoncio Martínez	*P. geniculatus*	2009	Female	Positive	No
869	VE16609	Miranda	Sucre	Leoncio Martínez	*P. geniculatus*	2009	Female	Positive	No
870	VE19409	Miranda	Sucre	Leoncio Martínez	*P. geniculatus*	2009	Female	Positive	No
871	VE14209	Miranda	Sucre	Leoncio Martínez	*P. geniculatus*	2009	Female	Positive	Yes
872	VE12309	Miranda	Sucre	Leoncio Martínez	*P. geniculatus*	2009	Male	NA	NA
873	VE10809	Miranda	Sucre	Leoncio Martínez	*P. geniculatus*	2009	Male	Negative	No
874	VE32109	Miranda	Sucre	Leoncio Martínez	*P. geniculatus*	2009	Male	Negative	No
875	VE35309	Miranda	Sucre	Leoncio Martínez	*P. geniculatus*	2009	Male	Negative	No
876	VE25009A	Miranda	Sucre	Leoncio Martínez	*P. geniculatus*	2009	Male	Negative	No
877	VE39909	Miranda	Sucre	Leoncio Martínez	*P. geniculatus*	2009	Male	Negative	No
878	VE12009	Miranda	Sucre	Leoncio Martínez	*P. geniculatus*	2009	Male	Positive	No
879	VE26109	Miranda	Sucre	Leoncio Martínez	*P. geniculatus*	2009	Male	Positive	No
880	VE37809B	Miranda	Baruta	Nuestra Señora del Rosario de Baruta	*P. geniculatus*	2009	Female	NA	NA

881	VE38209A	Miranda	Baruta	Nuestra Señora del Rosario de Baruta	*P. geniculatus*	2009	Female	NA	NA
882	VE38209B	Miranda	Baruta	Nuestra Señora del Rosario de Baruta	*P. geniculatus*	2009	Female	NA	NA
883	VE52009A	Miranda	Baruta	Nuestra Señora del Rosario de Baruta	*P. geniculatus*	2009	Female	NA	NA
884	VE52009B	Miranda	Baruta	Nuestra Señora del Rosario de Baruta	*P. geniculatus*	2009	Female	NA	NA
885	VE52009C	Miranda	Baruta	Nuestra Señora del Rosario de Baruta	*P. geniculatus*	2009	Female	NA	NA
886	VE52009D	Miranda	Baruta	Nuestra Señora del Rosario de Baruta	*P. geniculatus*	2009	Female	NA	NA
887	VE52009E	Miranda	Baruta	Nuestra Señora del Rosario de Baruta	*P. geniculatus*	2009	Female	NA	NA
888	VE47409	Miranda	Baruta	Nuestra Señora del Rosario de Baruta	*P. geniculatus*	2009	Female	NA	No
889	VE41509	Miranda	Baruta	Nuestra Señora del Rosario de Baruta	*P. geniculatus*	2009	Female	NA	No
890	VE47209	Miranda	Baruta	Nuestra Señora del Rosario de Baruta	*P. geniculatus*	2009	Female	NA	No
891	VE56709	Miranda	Baruta	Nuestra Señora del Rosario de Baruta	*P. geniculatus*	2009	Female	NA	No
892	VE41909	Miranda	Baruta	Nuestra Señora del Rosario de Baruta	*P. geniculatus*	2009	Female	NA	Yes
893	VE52909	Miranda	Baruta	Nuestra Señora del Rosario de Baruta	*P. geniculatus*	2009	Female	NA	Yes
894	VE60609	Miranda	Baruta	Nuestra Señora del Rosario de Baruta	*P. geniculatus*	2009	Female	Negative	No
895	VE5309	Miranda	Baruta	Nuestra Señora del Rosario de Baruta	*P. geniculatus*	2009	Female	Negative	No
896	VE46909	Miranda	Baruta	Nuestra Señora del Rosario de Baruta	*P. geniculatus*	2009	Female	Negative	No
897	VE46009A	Miranda	Baruta	Nuestra Señora del Rosario de Baruta	*P. geniculatus*	2009	Female	Negative	No
898	VE23509A	Miranda	Baruta	Nuestra Señora del Rosario de Baruta	*P. geniculatus*	2009	Female	Negative	Yes
899	VE4109	Miranda	Baruta	Nuestra Señora del Rosario de Baruta	*P. geniculatus*	2009	Female	Negative	Yes
900	VE45309B	Miranda	Baruta	Nuestra Señora del Rosario de Baruta	*P. geniculatus*	2009	Female	Negative	Yes
901	VE54809	Miranda	Baruta	Nuestra Señora del Rosario de Baruta	*P. geniculatus*	2009	Female	Negative	Yes
902	VE47909	Miranda	Baruta	Nuestra Señora del Rosario de Baruta	*P. geniculatus*	2009	Female	Negative	Yes
903	VE7009	Miranda	Baruta	Nuestra Señora del Rosario de Baruta	*P. geniculatus*	2009	Female	Negative	Yes
904	VE9609	Miranda	Baruta	Nuestra Señora del Rosario de Baruta	*P. geniculatus*	2009	Female	Positive	No
905	VE26709	Miranda	Baruta	Nuestra Señora del Rosario de Baruta	*P. geniculatus*	2009	Female	Positive	No
906	VE48109	Miranda	Baruta	Nuestra Señora del Rosario de Baruta	*P. geniculatus*	2009	Female	Positive	No
907	VE48409	Miranda	Baruta	Nuestra Señora del Rosario de Baruta	*P. geniculatus*	2009	Female	Positive	No
908	VE57209	Miranda	Baruta	Nuestra Señora del Rosario de Baruta	*P. geniculatus*	2009	Female	Positive	No
909	VE52509	Miranda	Baruta	Nuestra Señora del Rosario de Baruta	*T. nigromaculata*	2009	Female	Positive	No
910	VE37809A	Miranda	Baruta	Nuestra Señora del Rosario de Baruta	*P. geniculatus*	2009	Female	Positive	Yes
911	VE27509A	Miranda	Baruta	Nuestra Señora del Rosario de Baruta	*P. geniculatus*	2009	Female	Positive	Yes
912	VE27509B	Miranda	Baruta	Nuestra Señora del Rosario de Baruta	*P. geniculatus*	2009	Female	Positive	Yes
913	VE30409	Miranda	Baruta	Nuestra Señora del Rosario de Baruta	*P. geniculatus*	2009	Female	Positive	Yes
914	VE32609	Miranda	Baruta	Nuestra Señora del Rosario de Baruta	*P. geniculatus*	2009	Female	Positive	Yes
915	VE3509	Miranda	Baruta	Nuestra Señora del Rosario de Baruta	*P. geniculatus*	2009	Female	Positive	Yes
916	VE38609	Miranda	Baruta	Nuestra Señora del Rosario de Baruta	*P. geniculatus*	2009	Female	Positive	Yes
917	VE48909	Miranda	Baruta	Nuestra Señora del Rosario de Baruta	*P. geniculatus*	2009	Female	Positive	Yes
918	VE10109	Miranda	Baruta	Nuestra Señora del Rosario de Baruta	*P. geniculatus*	2009	Female	Positive	Yes
919	VE23709	Miranda	Baruta	Nuestra Señora del Rosario de Baruta	*P. geniculatus*	2009	Female	Positive	Yes
920	VE25009B	Miranda	Baruta	Nuestra Señora del Rosario de Baruta	*P. geniculatus*	2009	Female	Positive	Yes
921	VE26409B	Miranda	Baruta	Nuestra Señora del Rosario de Baruta	*P. geniculatus*	2009	Female	Positive	Yes
922	VE35409A	Miranda	Baruta	Nuestra Señora del Rosario de Baruta	*P. geniculatus*	2009	Female	Positive	Yes

923	VE35409B	Miranda	Baruta	Nuestra Señora del Rosario de Baruta	*P. geniculatus*	2009	Female	Positive	Yes
924	VE57809	Miranda	Baruta	Nuestra Señora del Rosario de Baruta	*P. geniculatus*	2009	Female	Positive	Yes
925	VE59009	Miranda	Baruta	Nuestra Señora del Rosario de Baruta	*P. geniculatus*	2009	Female	Positive	Yes
926	VE15809B	Miranda	Baruta	Nuestra Señora del Rosario de Baruta	*P. geniculatus*	2009	Female	Positive	No
927	VE12809	Miranda	Baruta	Nuestra Señora del Rosario de Baruta	*P. geniculatus*	2009	Male	NA	NA
928	VE30209A	Miranda	Baruta	Nuestra Señora del Rosario de Baruta	*P. geniculatus*	2009	Male	NA	NA
929	VE30209B	Miranda	Baruta	Nuestra Señora del Rosario de Baruta	*P. geniculatus*	2009	Male	NA	NA
930	VE35609B	Miranda	Baruta	Nuestra Señora del Rosario de Baruta	*P. geniculatus*	2009	Male	NA	NA
931	VE35609C	Miranda	Baruta	Nuestra Señora del Rosario de Baruta	*P. geniculatus*	2009	Male	NA	NA
932	VE40909	Miranda	Baruta	Nuestra Señora del Rosario de Baruta	*P. geniculatus*	2009	Male	NA	No
933	VE41309	Miranda	Baruta	Nuestra Señora del Rosario de Baruta	*P. geniculatus*	2009	Male	NA	No
934	VE41809	Miranda	Baruta	Nuestra Señora del Rosario de Baruta	*P. geniculatus*	2009	Male	NA	Yes
935	VE26009	Miranda	Baruta	Nuestra Señora del Rosario de Baruta	*P. geniculatus*	2009	Male	Negative	No
936	VE34709	Miranda	Baruta	Nuestra Señora del Rosario de Baruta	*P. geniculatus*	2009	Male	Negative	No
937	VE39409A	Miranda	Baruta	Nuestra Señora del Rosario de Baruta	*P. geniculatus*	2009	Male	Negative	No
938	VE47809	Miranda	Baruta	Nuestra Señora del Rosario de Baruta	*P. geniculatus*	2009	Male	Negative	No
939	VE10709	Miranda	Baruta	Nuestra Señora del Rosario de Baruta	*P. geniculatus*	2009	Male	Negative	Yes
940	VE65409	Miranda	Baruta	Nuestra Señora del Rosario de Baruta	*P. geniculatus*	2009	Male	Positive	No
941	VE13909	Miranda	Baruta	Nuestra Señora del Rosario de Baruta	*P. geniculatus*	2009	Male	Positive	No
942	VE59809	Miranda	Baruta	Nuestra Señora del Rosario de Baruta	*P. geniculatus*	2009	Male	Positive	No
943	VE3009	Miranda	Baruta	Nuestra Señora del Rosario de Baruta	*P. geniculatus*	2009	Male	Positive	No
944	VE34509	Miranda	Baruta	Nuestra Señora del Rosario de Baruta	*P. geniculatus*	2009	Male	Positive	No
945	VE48509A	Miranda	Baruta	Nuestra Señora del Rosario de Baruta	*P. geniculatus*	2009	Male	Positive	No
946	VE64109	Miranda	Baruta	Nuestra Señora del Rosario de Baruta	*P. geniculatus*	2009	Male	Positive	No
947	VE65509	Miranda	Baruta	Nuestra Señora del Rosario de Baruta	*P. geniculatus*	2009	Male	Positive	Yes
948	VE61609	Miranda	Baruta	Nuestra Señora del Rosario de Baruta	*P. geniculatus*	2009	Male	Positive	Yes
949	VE17409	Miranda	Baruta	Nuestra Señora del Rosario de Baruta	*P. geniculatus*	2009	Male	Positive	Yes
950	VE35609A	Miranda	Baruta	Nuestra Señora del Rosario de Baruta	*P. geniculatus*	2009	Male	Positive	Yes
951	VE60709	Miranda	Baruta	Nuestra Señora del Rosario de Baruta	*P. geniculatus*	2009	Nymph IV	Negative	Yes
952	VE1809	Miranda	Baruta	Nuestra Señora del Rosario de Baruta	*P. geniculatus*	2009	Nymph IV	Negative	No
953	VE18209B	Miranda	Sucre	Petare	*P. geniculatus*	2009	Female	NA	NA
954	VE15109A	Miranda	Sucre	Petare	*P. geniculatus*	2009	Female	NA	NA
955	VE18809	Miranda	Sucre	Petare	*P. geniculatus*	2009	Female	NA	NA
956	VE24209B	Miranda	Sucre	Petare	*P. geniculatus*	2009	Female	NA	NA
957	VE33309A	Miranda	Sucre	Petare	*P. geniculatus*	2009	Female	NA	NA
958	VE33309B	Miranda	Sucre	Petare	*P. geniculatus*	2009	Female	NA	NA
959	VE47309B	Miranda	Sucre	Petare	*P. geniculatus*	2009	Female	NA	NA
960	VE65109	Miranda	Sucre	Petare	*P. geniculatus*	2009	Female	NA	NA
961	VE32409	Miranda	Sucre	Petare	*P. geniculatus*	2009	Female	NA	No
962	VE21809	Miranda	Sucre	Petare	*P. geniculatus*	2009	Female	NA	No
963	VE33909	Miranda	Sucre	Petare	*P. geniculatus*	2009	Female	NA	No
964	VE49009	Miranda	Sucre	Petare	*P. geniculatus*	2009	Female	NA	No

965	VE42109A	Miranda	Sucre	Petare	*P. geniculatus*	2009	Female	NA	Yes
966	VE43209A	Miranda	Sucre	Petare	*P. geniculatus*	2009	Female	NA	Yes
967	VE47709	Miranda	Sucre	Petare	*P. geniculatus*	2009	Female	NA	Yes
968	VE15309	Miranda	Sucre	Petare	*P. geniculatus*	2009	Female	NA	Yes
969	VE48809	Miranda	Sucre	Petare	*P. geniculatus*	2009	Female	NA	Yes
970	VE9409	Miranda	Sucre	Petare	*P. geniculatus*	2009	Female	NA	Yes
971	VE5709	Miranda	Sucre	Petare	*P. geniculatus*	2009	Female	NA	Yes
972	VE45809	Miranda	Sucre	Petare	*T. nigromaculata*	2009	Female	Negative	No
973	VE8409	Miranda	Sucre	Petare	*P. geniculatus*	2009	Female	Negative	No
974	VE63909	Miranda	Sucre	Petare	*P. geniculatus*	2009	Female	Negative	No
975	VE15109B	Miranda	Sucre	Petare	*P. geniculatus*	2009	Female	Negative	No
976	VE33409B	Miranda	Sucre	Petare	*P. geniculatus*	2009	Female	Negative	No
977	VE7209	Miranda	Sucre	Petare	*P. geniculatus*	2009	Female	Negative	No
978	VE40009B	Miranda	Sucre	Petare	*P. geniculatus*	2009	Female	Negative	Yes
979	VE0209	Miranda	Sucre	Petare	*P. geniculatus*	2009	Female	Negative	Yes
980	VE11309	Miranda	Sucre	Petare	*P. geniculatus*	2009	Female	Negative	Yes
981	VE20309	Miranda	Sucre	Petare	*P. geniculatus*	2009	Female	Negative	Yes
982	VE23809	Miranda	Sucre	Petare	*P. geniculatus*	2009	Female	Negative	Yes
983	VE4809	Miranda	Sucre	Petare	*P. geniculatus*	2009	Female	Negative	Yes
984	VE7509A	Miranda	Sucre	Petare	*P. geniculatus*	2009	Female	Negative	Yes
985	VE40409B	Miranda	Sucre	Petare	*P. geniculatus*	2009	Female	Negative	Yes
986	VE51209B	Miranda	Sucre	Petare	*P. geniculatus*	2009	Female	Positive	No
987	VE24209A	Miranda	Sucre	Petare	*P. geniculatus*	2009	Female	Positive	No
988	VE32509	Miranda	Sucre	Petare	*P. geniculatus*	2009	Female	Positive	No
989	VE33409A	Miranda	Sucre	Petare	*P. geniculatus*	2009	Female	Positive	No
990	VE52609	Miranda	Sucre	Petare	*P. geniculatus*	2009	Female	Positive	No
991	VE53209	Miranda	Sucre	Petare	*P. geniculatus*	2009	Female	Positive	No
992	VE55009	Miranda	Sucre	Petare	*P. geniculatus*	2009	Female	Positive	No
993	VE55409	Miranda	Sucre	Petare	*P. geniculatus*	2009	Female	Positive	No
994	VE25909	Miranda	Sucre	Petare	*P. geniculatus*	2009	Female	Positive	No
995	VE59509	Miranda	Sucre	Petare	*P. geniculatus*	2009	Female	Positive	No
996	VE59909	Miranda	Sucre	Petare	*P. geniculatus*	2009	Female	Positive	No
997	VE63509	Miranda	Sucre	Petare	*P. geniculatus*	2009	Female	Positive	No
998	VE51209A	Miranda	Sucre	Petare	*P. geniculatus*	2009	Female	Positive	Yes
999	VE2909A	Miranda	Sucre	Petare	*P. geniculatus*	2009	Female	Positive	Yes
1000	VE33709	Miranda	Sucre	Petare	*P. geniculatus*	2009	Female	Positive	Yes
1001	VE37209B	Miranda	Sucre	Petare	*P. geniculatus*	2009	Female	Positive	Yes
1002	VE6209	Miranda	Sucre	Petare	*P. geniculatus*	2009	Female	Positive	Yes
1003	VE65309	Miranda	Sucre	Petare	*P. geniculatus*	2009	Female	Positive	Yes
1004	VE20009	Miranda	Sucre	Petare	*P. geniculatus*	2009	Female	Positive	Yes
1005	VE20809	Miranda	Sucre	Petare	*P. geniculatus*	2009	Female	Positive	Yes
1006	VE3409B	Miranda	Sucre	Petare	*P. geniculatus*	2009	Female	Positive	Yes

1007	VE36809	Miranda	Sucre	Petare	*P. geniculatus*	2009	Female	Positive	Yes
1008	VE47309A	Miranda	Sucre	Petare	*P. geniculatus*	2009	Female	Positive	Yes
1009	VE55209	Miranda	Sucre	Petare	*P. geniculatus*	2009	Female	Positive	Yes
1010	VE16709	Miranda	Sucre	Petare	*P. geniculatus*	2009	Female	Positive	Yes
1011	VE21509A	Miranda	Sucre	Petare	*P. geniculatus*	2009	Female	Positive	Yes
1012	VE21509B	Miranda	Sucre	Petare	*P. geniculatus*	2009	Female	Positive	Yes
1013	VE47109	Miranda	Sucre	Petare	*P. geniculatus*	2009	Female	Positive	Yes
1014	VE55709	Miranda	Sucre	Petare	*P. geniculatus*	2009	Female	Positive	Yes
1015	VE65209	Miranda	Sucre	Petare	*P. geniculatus*	2009	Female	Positive	Yes
1016	VE7709	Miranda	Sucre	Petare	*P. geniculatus*	2009	Female	Positive	Yes
1017	VE9509	Miranda	Sucre	Petare	*P. geniculatus*	2009	Female	Positive	Yes
1018	VE37209B	Miranda	Sucre	Petare	*P. geniculatus*	2009	Female	Positive	Yes
1019	VE7409	Miranda	Sucre	Petare	*P. geniculatus*	2009	Female	NA	NA
1020	VE15809A	Miranda	Sucre	Petare	*P. geniculatus*	2009	Female	NA	No
1021	VE20209	Miranda	Sucre	Petare	*P. geniculatus*	2009	Male	Positive	No
1022	VE2909C	Miranda	Sucre	Petare	*P. geniculatus*	2009	Male	NA	NA
1023	VE2909D	Miranda	Sucre	Petare	*P. geniculatus*	2009	Male	NA	NA
1024	VE2909E	Miranda	Sucre	Petare	*P. geniculatus*	2009	Male	NA	NA
1025	VE2909F	Miranda	Sucre	Petare	*P. geniculatus*	2009	Male	NA	NA
1026	VE18809	Miranda	Sucre	Petare	*P. geniculatus*	2009	Male	NA	NA
1027	VE34609B	Miranda	Sucre	Petare	*P. geniculatus*	2009	Male	NA	NA
1028	VE34609C	Miranda	Sucre	Petare	*P. geniculatus*	2009	Male	NA	NA
1029	VE55109A	Miranda	Sucre	Petare	*P. geniculatus*	2009	Male	NA	NA
1030	VE55109B	Miranda	Sucre	Petare	*P. geniculatus*	2009	Male	NA	NA
1031	VE55109C	Miranda	Sucre	Petare	*P. geniculatus*	2009	Male	NA	NA
1032	VE32309	Miranda	Sucre	Petare	*P. geniculatus*	2009	Male	NA	No
1033	VE61509	Miranda	Sucre	Petare	*P. geniculatus*	2009	Male	NA	No
1034	VE20509	Miranda	Sucre	Petare	*P. geniculatus*	2009	Male	NA	No
1035	VE31509	Miranda	Sucre	Petare	*P. geniculatus*	2009	Male	NA	No
1036	VE32209	Miranda	Sucre	Petare	*P. geniculatus*	2009	Male	NA	No
1037	VE41209	Miranda	Sucre	Petare	*P. geniculatus*	2009	Male	NA	No
1038	VE62709A	Miranda	Sucre	Petare	*P. geniculatus*	2009	Male	NA	No
1039	VE15009	Miranda	Sucre	Petare	*P. geniculatus*	2009	Male	NA	No
1040	VE8709	Miranda	Sucre	Petare	*P. geniculatus*	2009	Male	NA	Yes
1041	VE1509	Miranda	Sucre	Petare	*P. geniculatus*	2009	Male	Negative	No
1042	VE18209A	Miranda	Sucre	Petare	*P. geniculatus*	2009	Male	Negative	No
1043	VE1909	Miranda	Sucre	Petare	*P. geniculatus*	2009	Male	Negative	No
1044	VE57909	Miranda	Sucre	Petare	*P. geniculatus*	2009	Male	Negative	No
1045	VE2709	Miranda	Sucre	Petare	*P. geniculatus*	2009	Male	Negative	No
1046	VE27609	Miranda	Sucre	Petare	*P. geniculatus*	2009	Male	Negative	No
1047	VE9909	Miranda	Sucre	Petare	*P. geniculatus*	2009	Male	Negative	No
1048	VE19809	Miranda	Sucre	Petare	*P. geniculatus*	2009	Male	Negative	No

1049	VE62809	Miranda	Sucre	Petare	*P. geniculatus*	2009	Male	Negative	Yes
1050	VE29909	Miranda	Sucre	Petare	*P. geniculatus*	2009	Male	Negative	Yes
1051	VE62209	Miranda	Sucre	Petare	*P. geniculatus*	2009	Male	Negative	Yes
1052	VE23109	Miranda	Sucre	Petare	*P. geniculatus*	2009	Male	Positive	No
1053	VE25109	Miranda	Sucre	Petare	*P. geniculatus*	2009	Male	Positive	No
1054	VE64809	Miranda	Sucre	Petare	*P. geniculatus*	2009	Male	Positive	No
1055	VE12109	Miranda	Sucre	Petare	*P. geniculatus*	2009	Male	Positive	No
1056	VE12609B	Miranda	Sucre	Petare	*P. geniculatus*	2009	Male	Positive	No
1057	VE23509B	Miranda	Sucre	Petare	*P. geniculatus*	2009	Male	Positive	No
1058	VE27209A	Miranda	Sucre	Petare	*P. geniculatus*	2009	Male	Positive	No
1059	VE42609	Miranda	Sucre	Petare	*P. geniculatus*	2009	Male	Positive	No
1060	VE54109	Miranda	Sucre	Petare	*P. geniculatus*	2009	Male	Positive	No
1061	VE9709	Miranda	Sucre	Petare	*P. geniculatus*	2009	Male	Positive	No
1062	VE2909B	Miranda	Sucre	Petare	*P. geniculatus*	2009	Male	Positive	Yes
1063	VE62609B	Miranda	Sucre	Petare	*P. geniculatus*	2009	Male	Positive	Yes
1064	VE8609	Miranda	Sucre	Petare	*P. geniculatus*	2009	Male	Positive	Yes
1065	VE14909	Miranda	Sucre	Petare	*P. geniculatus*	2009	Male	Positive	Yes
1066	VE20409	Miranda	Sucre	Petare	*P. geniculatus*	2009	Male	Positive	Yes
1067	VE20609	Miranda	Sucre	Petare	*P. geniculatus*	2009	Male	Positive	Yes
1068	VE24609	Miranda	Sucre	Petare	*P. geniculatus*	2009	Male	Positive	Yes
1069	VE27909	Miranda	Sucre	Petare	*P. geniculatus*	2009	Male	Positive	Yes
1070	VE28109	Miranda	Sucre	Petare	*P. geniculatus*	2009	Male	Positive	Yes
1071	VE3409A	Miranda	Sucre	Petare	*P. geniculatus*	2009	Male	Positive	Yes
1072	VE34609A	Miranda	Sucre	Petare	*P. geniculatus*	2009	Male	Positive	Yes
1073	VE37309	Miranda	Sucre	Petare	*P. geniculatus*	2009	Male	Positive	Yes
1074	VE51609	Miranda	Sucre	Petare	*P. geniculatus*	2009	Male	Positive	Yes
1075	VE0309A	Miranda	Sucre	Petare	*P. geniculatus*	2009	Nymph III	NA	NA
1076	VE0309B	Miranda	Sucre	Petare	*P. geniculatus*	2009	Nymph III	NA	NA
1077	VE64509	Miranda	Sucre	Petare	*P. geniculatus*	2009	Nymph III	NA	No
1078	VE0309C	Miranda	Sucre	Petare	*P. geniculatus*	2009	Nymph III	Positive	Yes
1079	VE29609	Miranda	Sucre	Petare	*P. geniculatus*	2009	Nymph III	Positive	Yes
1080	VE60309	Miranda	Sucre	Petare	*P. geniculatus*	2009	Nymph IV	NA	Yes
1081	VE56109	Miranda	Sucre	Petare	*P. geniculatus*	2009	Nymph IV	Negative	Yes
1082	VE6409	Miranda	Sucre	Petare	*P. geniculatus*	2009	Nymph IV	Negative	Yes
1083	VE61409A	Miranda	Sucre	Petare	*P. geniculatus*	2009	Nymph IV	Positive	Yes
1084	VE59709	Miranda	Sucre	Petare	*P. geniculatus*	2009	Nymph IV	Positive	Yes
1085	VE4609B	Miranda	Sucre	Petare	*P. geniculatus*	2009	Nymph V	NA	NA
1086	VE4609A	Miranda	Sucre	Petare	*P. geniculatus*	2009	Nymph V	Negative	No
1087	VE9109	Miranda	Sucre	Petare	*P. geniculatus*	2009	Nymph V	Positive	Yes
1088	VE37609A	MIRANDA	El Hatillo	Santa Rosalía de Palermo	*P. geniculatus*	2009	Female	NA	NA
1089	VE57309	Miranda	El Hatillo	Santa Rosalía de Palermo	*P. geniculatus*	2009	Female	NA	NA
1090	VE58509B	Miranda	El Hatillo	Santa Rosalía de Palermo	*P. geniculatus*	2009	Female	NA	NA

1091	VE21609	Miranda	El Hatillo	Santa Rosalía de Palermo	*P. geniculatus*	2009	Female	NA	No
1092	VE41409	Miranda	El Hatillo	Santa Rosalía de Palermo	*P. geniculatus*	2009	Female	NA	No
1093	VE24809	Miranda	El Hatillo	Santa Rosalía de Palermo	*P. geniculatus*	2009	Female	Negative	No
1094	VE39509A	Miranda	El Hatillo	Santa Rosalía de Palermo	*P. geniculatus*	2009	Female	Negative	Yes
1095	VE39509B	Miranda	El Hatillo	Santa Rosalía de Palermo	*P. geniculatus*	2009	Female	Negative	Yes
1096	VE5009	Miranda	El Hatillo	Santa Rosalía de Palermo	*P. geniculatus*	2009	Female	Negative	Yes
1097	VE6709	Miranda	El Hatillo	Santa Rosalía de Palermo	*P. geniculatus*	2009	Female	Negative	Yes
1098	VE33209	Miranda	El Hatillo	Santa Rosalía de Palermo	*P. geniculatus*	2009	Female	Negative	Yes
1099	VE31109A	Miranda	El Hatillo	Santa Rosalía de Palermo	*P. geniculatus*	2009	Female	Positive	No
1100	VE31109B	Miranda	El Hatillo	Santa Rosalía de Palermo	*P. geniculatus*	2009	Female	Positive	No
1101	VE30909	Miranda	El Hatillo	Santa Rosalía de Palermo	*P. geniculatus*	2009	Female	Positive	No
1102	VE13109	Miranda	El Hatillo	Santa Rosalía de Palermo	*P. geniculatus*	2009	Female	Positive	Yes
1103	VE55809	Miranda	El Hatillo	Santa Rosalía de Palermo	*P. geniculatus*	2009	Female	Positive	Yes
1104	VE37109	Miranda	El Hatillo	Santa Rosalía de Palermo	*P. geniculatus*	2009	Female	Positive	Yes
1105	VE41609	Miranda	El Hatillo	Santa Rosalía de Palermo	*P. geniculatus*	2009	Female	Positive	Yes
1106	VE46809	Miranda	El Hatillo	Santa Rosalía de Palermo	*P. geniculatus*	2009	Female	Positive	Yes
1107	VE47009	Miranda	El Hatillo	Santa Rosalía de Palermo	*P. geniculatus*	2009	Female	Positive	Yes
1108	VE65609	Miranda	El Hatillo	Santa Rosalía de Palermo	*P. geniculatus*	2009	Female	Positive	Yes
1109	VE18009	Miranda	El Hatillo	Santa Rosalía de Palermo	*P. geniculatus*	2009	Female	Positive	Yes
1110	VE59609	Miranda	El Hatillo	Santa Rosalía de Palermo	*P. geniculatus*	2009	Female	Positive	Yes
1111	VE62509B	Miranda	El Hatillo	Santa Rosalía de Palermo	*P. geniculatus*	2009	Female	Positive	Yes
1112	VE2109	Miranda	El Hatillo	Santa Rosalía de Palermo	*P. geniculatus*	2009	Female	Positive	Yes
1113	VE15909	Miranda	El Hatillo	Santa Rosalía de Palermo	*P. geniculatus*	2009	Female	Negative	No
1114	VE17109	Miranda	El Hatillo	Santa Rosalía de Palermo	*P. geniculatus*	2009	Male	NA	NA
1115	VE37609C	MIRANDA	El Hatillo	Santa Rosalía de Palermo	*P. geniculatus*	2009	Male	NA	NA
1116	VE58509A	Miranda	El Hatillo	Santa Rosalía de Palermo	*P. geniculatus*	2009	Male	NA	NA
1117	VE44409A	Miranda	El Hatillo	Santa Rosalía de Palermo	*P. geniculatus*	2009	Male	Negative	No
1118	VE62509A	Miranda	El Hatillo	Santa Rosalía de Palermo	*P. geniculatus*	2009	Male	Negative	No
1119	VE3909	Miranda	El Hatillo	Santa Rosalía de Palermo	*P. geniculatus*	2009	Male	Negative	No
1120	VE38109	Miranda	El Hatillo	Santa Rosalía de Palermo	*P. geniculatus*	2009	Male	Negative	Yes
1121	VE37609B	MIRANDA	El Hatillo	Santa Rosalía de Palermo	*P. geniculatus*	2009	Male	Positive	No
1122	VE22709	Miranda	El Hatillo	Santa Rosalía de Palermo	*P. geniculatus*	2009	Male	Positive	No
1123	VE18609	Miranda	El Hatillo	Santa Rosalía de Palermo	*P. geniculatus*	2009	Male	Positive	Yes
1124	VE4309	Miranda	El Hatillo	Santa Rosalía de Palermo	*P. geniculatus*	2009	Male	Positive	Yes
1125	VE64009	Miranda	El Hatillo	Santa Rosalía de Palermo	*P. geniculatus*	2009	Male	Positive	Yes
1126	VE63009	Miranda	El Hatillo	Santa Rosalía de Palermo	*P. geniculatus*	2009	Nymph IV	Positive	Yes
1127	VE105510	DC	Libertador	23 de enero	*P. geniculatus*	2010	Male	Positive	No
1128	VE43510	DC	Libertador	23 de enero	*P. geniculatus*	2010	Male	Positive	No
1129	VE78810	DC	Libertador	23 de enero	*P. geniculatus*	2010	Male	Positive	Yes
1130	VE86710	DC	Libertador	Altagracia	*P. geniculatus*	2010	Female	NA	Yes
1131	VE45710	DC	Libertador	Altagracia	*P. geniculatus*	2010	Female	Positive	No
1132	VE55610	DC	Libertador	Altagracia	*P. geniculatus*	2010	Female	Positive	No

1133	VE39210	DC	Libertador	Altagracia	*P. geniculatus*	2010	Female	Positive	Yes
1134	VE33810	DC	Libertador	Altagracia	*P. geniculatus*	2010	Female	Positive	Yes
1135	VE124710	DC	Libertador	Altagracia	*P. geniculatus*	2010	Female	Positive	Yes
1136	VE128710B	DC	Libertador	Altagracia	*P. geniculatus*	2010	Female	NA	NA
1137	VE20810	DC	Libertador	Altagracia	*P. geniculatus*	2010	Male	Negative	No
1138	VE126310	DC	Libertador	Altagracia	*P. geniculatus*	2010	Male	Positive	No
1139	VE128710C	DC	Libertador	Altagracia	*P. geniculatus*	2010	Male	Positive	No
1140	VE95410	DC	Libertador	Altagracia	*P. geniculatus*	2010	Male	Positive	No
1141	VE87810	DC	Libertador	Altagracia	*P. geniculatus*	2010	Male	Positive	No
1142	VE33110	DC	Libertador	Altagracia	*P. geniculatus*	2010	Male	Positive	Yes
1143	VE128710A	DC	Libertador	Altagracia	*P. geniculatus*	2010	Male	NA	NA
1144	VE33410	DC	Libertador	Altagracia	*P. geniculatus*	2010	Male	NA	No
1145	VE76710	DC	Libertador	Antímano	*P. geniculatus*	2010	Female	Negative	Yes
1146	VE142810	DC	Libertador	Antímano	*P. geniculatus*	2010	Female	Positive	Yes
1147	VE12910	DC	Libertador	Antímano	*P. geniculatus*	2010	Female	Positive	Yes
1148	VE41310	DC	Libertador	Antímano	*P. geniculatus*	2010	Female	NA	NA
1149	VE139710	DC	Libertador	Antímano	*P. geniculatus*	2010	Female	NA	Yes
1150	VE14210	DC	Libertador	Antímano	*P. geniculatus*	2010	Female	NA	No
1151	VE106210	DC	Libertador	Antímano	*P. geniculatus*	2010	Male	Negative	No
1152	VE92210	DC	Libertador	Antímano	*P. geniculatus*	2010	Male	Negative	Yes
1153	VE34010	DC	Libertador	Antímano	*P. geniculatus*	2010	Male	Positive	No
1154	VE85810	DC	Libertador	Antímano	*P. geniculatus*	2010	Male	Positive	No
1155	VE19310	DC	Libertador	Antímano	*P. geniculatus*	2010	Male	Positive	No
1156	VE41810	DC	Libertador	Antímano	*P. geniculatus*	2010	Male	Positive	No
1157	VE52510	DC	Libertador	Antímano	*P. geniculatus*	2010	Male	Positive	Yes
1158	VE70310	DC	Libertador	Antímano	*P. geniculatus*	2010	Nymph III	Negative	Yes
1159	VE55910	DC	Libertador	Caricuao	*P. geniculatus*	2010	Female	Negative	No
1160	VE129910	DC	Libertador	Caricuao	*P. geniculatus*	2010	Female	Positive	No
1161	VE78110	DC	Libertador	Caricuao	*P. geniculatus*	2010	Female	Positive	No
1162	VE41710	DC	Libertador	Caricuao	*P. geniculatus*	2010	Female	Positive	No
1163	VE67010	DC	Libertador	Caricuao	*P. geniculatus*	2010	Female	Positive	No
1164	VE95610	DC	Libertador	Caricuao	*P. geniculatus*	2010	Female	Positive	No
1165	VE37910	DC	Libertador	Caricuao	*P. geniculatus*	2010	Female	Positive	No
1166	VE138210B	DC	Libertador	Caricuao	*P. geniculatus*	2010	Female	Positive	Yes
1167	VE40210	DC	Libertador	Caricuao	*P. geniculatus*	2010	Female	Positive	Yes
1168	VE25310	DC	Libertador	Caricuao	*P. geniculatus*	2010	Female	Positive	Yes
1169	VE46310	DC	Libertador	Caricuao	*P. geniculatus*	2010	Female	Positive	Yes
1170	VE65010	DC	Libertador	Caricuao	*P. geniculatus*	2010	Female	Positive	Yes
1171	VE127510	DC	Libertador	Caricuao	*P. geniculatus*	2010	Female	Positive	Yes
1172	VE97610	DC	Libertador	Caricuao	*P. geniculatus*	2010	Female	Positive	Yes
1173	VE109610	DC	Libertador	Caricuao	*P. geniculatus*	2010	Male	NA	No
1174	VE54710	DC	Libertador	Caricuao	*P. geniculatus*	2010	Male	Negative	No

1175	VE11510	DC	Libertador	Caricuao	*P. geniculatus*	2010	Male	Negative	No
1176	VE15210	DC	Libertador	Caricuao	*P. geniculatus*	2010	Male	Negative	No
1177	VE49910A	DC	Libertador	Caricuao	*P. geniculatus*	2010	Male	Negative	No
1178	VE66610	DC	Libertador	Caricuao	*P. geniculatus*	2010	Male	Negative	No
1179	VE88510	DC	Libertador	Caricuao	*P. geniculatus*	2010	Male	Negative	No
1180	VE19410	DC	Libertador	Caricuao	*P. geniculatus*	2010	Male	Negative	Yes
1181	VE83910	DC	Libertador	Caricuao	*P. geniculatus*	2010	Male	Negative	Yes
1182	VE65610	DC	Libertador	Caricuao	*P. geniculatus*	2010	Male	Positive	No
1183	VE91210	DC	Libertador	Caricuao	*P. geniculatus*	2010	Male	Positive	No
1184	VE59210	DC	Libertador	Caricuao	*P. geniculatus*	2010	Male	Positive	No
1185	VE53310	DC	Libertador	Caricuao	*P. geniculatus*	2010	Male	Positive	No
1186	VE69610	DC	Libertador	Caricuao	*P. geniculatus*	2010	Male	Positive	No
1187	VE47610	DC	Libertador	Caricuao	*P. geniculatus*	2010	Male	Positive	No
1188	VE30610	DC	Libertador	Caricuao	*P. geniculatus*	2010	Male	Positive	No
1189	VE119710A	DC	Libertador	Caricuao	*P. geniculatus*	2010	Male	Positive	Yes
1190	VE47710	DC	Libertador	Caricuao	*P. geniculatus*	2010	Male	Positive	Yes
1191	VE2710	DC	Libertador	Caricuao	*P. geniculatus*	2010	Male	Positive	Yes
1192	VE115810	DC	Libertador	Caricuao	*P. geniculatus*	2010	Male	Negative	No
1193	VE121210	DC	Libertador	Coche	*P. geniculatus*	2010	Female	Negative	No
1194	VE14610	DC	Libertador	Coche	*P. geniculatus*	2010	Female	Negative	No
1195	VE16310	DC	Libertador	Coche	*P. geniculatus*	2010	Female	NA	Yes
1196	VE114010	DC	Libertador	Coche	*P. geniculatus*	2010	Male	Positive	Yes
1197	VE60610	DC	Libertador	Coche	*P. geniculatus*	2010	Male	Positive	Yes
1198	VE100810	DC	Libertador	Coche	*P. geniculatus*	2010	Male	Positive	Yes
1199	VE18410	DC	Libertador	Coche	*P. geniculatus*	2010	Male	NA	No
1200	VE75610	DC	Libertador	Coche	*T. maculata*	2010	Female	NA	NA
1201	VE128210	DC	Libertador	El Junquito	*P. geniculatus*	2010	Nymph V	Positive	Yes
1202	VE84410	DC	Libertador	El Junquito	*P. geniculatus*	2010	Female	NA	No
1203	VE106810	DC	Libertador	El Junquito	*P. geniculatus*	2010	Female	NA	No
1204	VE99610	DC	Libertador	El Junquito	*P. geniculatus*	2010	Female	NA	No
1205	VE65810	DC	Libertador	El Junquito	*P. geniculatus*	2010	Female	NA	NA
1206	VE9510	DC	Libertador	El Junquito	*P. geniculatus*	2010	Female	Negative	No
1207	VE136310	DC	Libertador	El Junquito	*P. geniculatus*	2010	Female	Negative	Yes
1208	VE30310	DC	Libertador	El Junquito	*P. geniculatus*	2010	Female	Negative	Yes
1209	VE60910	DC	Libertador	El Junquito	*P. geniculatus*	2010	Female	Negative	Yes
1210	VE116810	DC	Libertador	El Junquito	*P. geniculatus*	2010	Female	Positive	No
1211	VE32910	DC	Libertador	El Junquito	*P. geniculatus*	2010	Female	Positive	No
1212	VE122910	DC	Libertador	El Junquito	*P. geniculatus*	2010	Female	Positive	No
1213	VE98810A	DC	Libertador	El Junquito	*P. geniculatus*	2010	Male	Positive	No
1214	VE98810B	DC	Libertador	El Junquito	*P. geniculatus*	2010	Female	Positive	No
1215	VE55810	DC	Libertador	El Junquito	*P. geniculatus*	2010	Female	Positive	No
1216	VE51410	DC	Libertador	El Junquito	*P. geniculatus*	2010	Female	Positive	No

1217	VE60010	DC	Libertador	El Junquito	*P. geniculatus*	2010	Female	Positive	No
1218	VE59010	DC	Libertador	El Junquito	*P. geniculatus*	2010	Female	Positive	No
1219	VE59110	DC	Libertador	El Junquito	*P. geniculatus*	2010	Female	Positive	No
1220	VE13810	DC	Libertador	El Junquito	*P. geniculatus*	2010	Female	Positive	No
1221	VE45310A	DC	Libertador	El Junquito	*P. geniculatus*	2010	Male	Positive	No
1222	VE45310B	DC	Libertador	El Junquito	*P. geniculatus*	2010	Male	Positive	No
1223	VE45310C	DC	Libertador	El Junquito	*P. geniculatus*	2010	Male	Positive	No
1224	VE45310D	DC	Libertador	El Junquito	*P. geniculatus*	2010	Female	Positive	Yes
1225	VE120410	DC	Libertador	El Junquito	*P. geniculatus*	2010	Female	Positive	Yes
1226	VE122510	DC	Libertador	El Junquito	*P. geniculatus*	2010	Female	Positive	Yes
1227	VE47410	DC	Libertador	El Junquito	*P. geniculatus*	2010	Female	Positive	Yes
1228	VE57010	DC	Libertador	El Junquito	*P. geniculatus*	2010	Female	Positive	Yes
1229	VE8510	DC	Libertador	El Junquito	*P. geniculatus*	2010	Female	Positive	Yes
1230	VE61210	DC	Libertador	El Junquito	*P. geniculatus*	2010	Female	Positive	Yes
1231	VE68110	DC	Libertador	El Junquito	*P. geniculatus*	2010	Female	Positive	Yes
1232	VE52710	DC	Libertador	El Junquito	*P. geniculatus*	2010	Female	Positive	Yes
1233	VE69010	DC	Libertador	El Junquito	*P. geniculatus*	2010	Female	Positive	Yes
1234	VE103010	DC	Libertador	El Junquito	*P. geniculatus*	2010	Female	Positive	Yes
1235	VE83010	DC	Libertador	El Junquito	*P. geniculatus*	2010	Female	Positive	Yes
1236	VE20210	DC	Libertador	El Junquito	*P. geniculatus*	2010	Female	Positive	Yes
1237	VE111110	DC	Libertador	El Junquito	*P. geniculatus*	2010	Female	Positive	Yes
1238	VE62610	DC	Libertador	El Junquito	*P. geniculatus*	2010	Female	Positive	Yes
1239	VE52610	DC	Libertador	El Junquito	*P. geniculatus*	2010	Female	Positive	Yes
1240	VE23010	DC	Libertador	El Junquito	*P. geniculatus*	2010	Female	NA	No
1241	VE112110	DC	Libertador	El Junquito	*P. geniculatus*	2010	Female	NA	No
1242	VE108510	DC	Libertador	El Junquito	*P. geniculatus*	2010	Female	NA	No
1243	VE54610	DC	Libertador	El Junquito	*P. geniculatus*	2010	Male	NA	Yes
1244	VE76310	DC	Libertador	El Junquito	*P. geniculatus*	2010	Male	NA	Yes
1245	VE23410	DC	Libertador	El Junquito	*P. geniculatus*	2010	Male	Negative	No
1246	VE72510	DC	Libertador	El Junquito	*P. geniculatus*	2010	Male	Negative	No
1247	VE73410	DC	Libertador	El Junquito	*P. geniculatus*	2010	Male	Negative	Yes
1248	VE96710	DC	Libertador	El Junquito	*P. geniculatus*	2010	Male	Negative	Yes
1249	VE34410	DC	Libertador	El Junquito	*P. geniculatus*	2010	Male	Negative	Yes
1250	VE51810	DC	Libertador	El Junquito	*P. geniculatus*	2010	Male	Negative	Yes
1251	VE140610	DC	Libertador	El Junquito	*P. geniculatus*	2010	Male	Positive	No
1252	VE85410	DC	Libertador	El Junquito	*P. geniculatus*	2010	Male	Positive	No
1253	VE64210	DC	Libertador	El Junquito	*P. geniculatus*	2010	Male	Positive	No
1254	VE2610A	DC	Libertador	El Junquito	*P. geniculatus*	2010	Male	Positive	No
1255	VE2610B	DC	Libertador	El Junquito	*P. geniculatus*	2010	Male	Positive	No
1256	VE27110	DC	Libertador	El Junquito	*P. geniculatus*	2010	Male	Positive	No
1257	VE53410	DC	Libertador	El Junquito	*P. geniculatus*	2010	Male	Positive	No
1258	VE103710	DC	Libertador	El Junquito	*P. geniculatus*	2010	Male	Positive	No

1259	VE72710	DC	Libertador	El Junquito	*P. geniculatus*	2010	Male	Positive	No
1260	VE40910	DC	Libertador	El Junquito	*P. geniculatus*	2010	Male	Positive	No
1261	VE2110(LBMP)	DC	Libertador	El Junquito	*P. geniculatus*	2010	Male	Positive	No
1262	VE36410	DC	Libertador	El Junquito	*P. geniculatus*	2010	Male	Positive	No
1263	VE39410	DC	Libertador	El Junquito	*P. geniculatus*	2010	Male	Positive	No
1264	VE58010	DC	Libertador	El Junquito	*P. geniculatus*	2010	Male	Positive	No
1265	VE53010	DC	Libertador	El Junquito	*P. geniculatus*	2010	Male	Positive	Yes
1266	VE90610	DC	Libertador	El Junquito	*P. geniculatus*	2010	Male	Positive	Yes
1267	VE61510	DC	Libertador	El Junquito	*P. geniculatus*	2010	Male	Positive	Yes
1268	VE34110	DC	Libertador	El Junquito	*P. geniculatus*	2010	Male	Positive	Yes
1269	VE66410	DC	Libertador	El Junquito	*P. geniculatus*	2010	Male	Positive	Yes
1270	VE72910	DC	Libertador	El Junquito	*P. geniculatus*	2010	Male	Positive	Yes
1271	VE44210	DC	Libertador	El Junquito	*P. geniculatus*	2010	Male	Positive	Yes
1272	VE43610A	DC	Libertador	El Junquito	*P. geniculatus*	2010	Female	Positive	No
1273	VE43610B	DC	Libertador	El Junquito	*P. geniculatus*	2010	Male	Positive	Yes
1274	VE112010A	DC	Libertador	El Junquito	*P. geniculatus*	2010	Male	Positive	No
1275	VE112010B	DC	Libertador	El Junquito	*P. geniculatus*	2010	Male	Positive	Yes
1276	VE39810	DC	Libertador	El Junquito	*P. geniculatus*	2010	Male	Positive	Yes
1277	VE125110	DC	Libertador	El Junquito	*P. geniculatus*	2010	Male	NA	NA
1278	VE33910	DC	Libertador	El Junquito	*P. geniculatus*	2010	Male	NA	No
1279	VE51110B	DC	Libertador	El Junquito	*P. geniculatus*	2010	Male	NA	NA
1280	VE8710	DC	Libertador	El Junquito	*T. nigromaculata*	2010	Female	Negative	Yes
1281	VE113610	DC	Libertador	El Junquito	*T. nigromaculata*	2010	Female	Negative	Yes
1282	VE64510	DC	Libertador	El Junquito	*T. nigromaculata*	2010	Female	Positive	No
1283	VE116710	DC	Libertador	El Junquito	*T. nigromaculata*	2010	Male	NA	NA
1284	VE141510	DC	Libertador	El Paraiso	*P. geniculatus*	2010	Female	Positive	Yes
1285	VE36110	DC	Libertador	El Paraiso	*P. geniculatus*	2010	Female	Positive	Yes
1286	VE63110	DC	Libertador	El Paraiso	*P. geniculatus*	2010	Female	Positive	Yes
1287	VE106010	DC	Libertador	El Paraiso	*P. geniculatus*	2010	Male	NA	No
1288	VE133110	DC	Libertador	El Paraiso	*P. geniculatus*	2010	Male	Positive	No
1289	VE94510	DC	Libertador	El Paraiso	*P. geniculatus*	2010	Male	Positive	No
1290	VE74310A	DC	Libertador	El Paraiso	*P. geniculatus*	2010	Female	NA	NA
1291	VE74310B	DC	Libertador	El Paraiso	*P. geniculatus*	2010	Male	Positive	No
1292	VE11610	DC	Libertador	El Paraiso	*P. geniculatus*	2010	Male	Positive	Yes
1293	VE18210	DC	Libertador	El Paraiso	*P. geniculatus*	2010	Male	Positive	Yes
1294	VE119510	DC	Libertador	El Paraiso	*P. geniculatus*	2010	Male	NA	NA
1295	VE 126110	DC	Libertador	El Recreo	*P. geniculatus*	2010	Female	Negative	No
1296	VE83710	DC	Libertador	El Recreo	*P. geniculatus*	2010	Female	Positive	No
1297	VE106310	DC	Libertador	El Recreo	*P. geniculatus*	2010	Female	Positive	No
1298	VE5210	DC	Libertador	El Recreo	*P. geniculatus*	2010	Female	Positive	No
1299	VE64010A	DC	Libertador	El Recreo	*P. geniculatus*	2010	Male	NA	NA
1300	VE64010B	DC	Libertador	El Recreo	*P. geniculatus*	2010	Female	Positive	No

1301	VE126010	DC	Libertador	El Recreo	*P. geniculatus*	2010	Female	Positive	No
1302	VE54910	DC	Libertador	El Recreo	*P. geniculatus*	2010	Female	Positive	No
1303	VE117410	DC	Libertador	El Recreo	*P. geniculatus*	2010	Female	NA	NA
1304	VE133810B	DC	Libertador	El Recreo	*P. geniculatus*	2010	Male	NA	No
1305	VE108610	DC	Libertador	El Recreo	*P. geniculatus*	2010	Male	Negative	No
1306	VE133810A	DC	Libertador	El Recreo	*P. geniculatus*	2010	Male	Positive	No
1307	VE30510	DC	Libertador	El Recreo	*P. geniculatus*	2010	Male	Positive	No
1308	VE117510	DC	Libertador	El Recreo	*P. geniculatus*	2010	Male	Positive	No
1309	VE126610	DC	Libertador	El Recreo	*P. geniculatus*	2010	Male	Positive	No
1310	VE124410	DC	Libertador	El Recreo	*P. geniculatus*	2010	Male	NA	NA
1311	VE74010	DC	Libertador	El Recreo	*P. geniculatus*	2010	Male	NA	No
1312	VE80410	DC	Libertador	El Valle	*P. geniculatus*	2010	Female	Negative	No
1313	VE104410	DC	Libertador	El Valle	*P. geniculatus*	2010	Male	Negative	No
1314	VE75910	DC	Libertador	El Valle	*P. geniculatus*	2010	Male	Negative	Yes
1315	VE47210	DC	Libertador	El Valle	*P. geniculatus*	2010	Male	Positive	No
1316	VE81510	DC	Libertador	El Valle	*P. geniculatus*	2010	Male	Positive	Yes
1317	VE76110A	DC	Libertador	La Pastora	*P. geniculatus*	2010	Nymph IV	Positive	No
1318	VE76110B	DC	Libertador	La Pastora	*P. geniculatus*	2010	Nymph IV	Positive	Yes
1319	VE76110C	DC	Libertador	La Pastora	*P. geniculatus*	2010	Nymph IV	Positive	Yes
1320	VE76110D	DC	Libertador	La Pastora	*P. geniculatus*	2010	Female	Negative	Yes
1321	VE38710	DC	Libertador	La Pastora	*P. geniculatus*	2010	Female	Negative	Yes
1322	VE34610	DC	Libertador	La Pastora	*P. geniculatus*	2010	Female	Negative	Yes
1323	VE98410	DC	Libertador	La Pastora	*P. geniculatus*	2010	Female	Negative	Yes
1324	VE56710	DC	Libertador	La Pastora	*P. geniculatus*	2010	Female	Negative	Yes
1325	VE94010A	DC	Libertador	La Pastora	*P. geniculatus*	2010	Male	NA	NA
1326	VE94010B	DC	Libertador	La Pastora	*P. geniculatus*	2010	Female	Positive	No
1327	VE42510	DC	Libertador	La Pastora	*P. geniculatus*	2010	Female	Positive	No
1328	VE90210	DC	Libertador	La Pastora	*P. geniculatus*	2010	Female	Positive	No
1329	VE55310	DC	Libertador	La Pastora	*P. geniculatus*	2010	Female	Positive	No
1330	VE93110	DC	Libertador	La Pastora	*P. geniculatus*	2010	Female	Positive	No
1331	VE76010	DC	Libertador	La Pastora	*P. geniculatus*	2010	Female	Positive	No
1332	VE53710	DC	Libertador	La Pastora	*P. geniculatus*	2010	Female	Positive	No
1333	VE95210	DC	Libertador	La Pastora	*P. geniculatus*	2010	Female	Positive	No
1334	VE117610	DC	Libertador	La Pastora	*P. geniculatus*	2010	Female	Positive	Yes
1335	VE137610	DC	Libertador	La Pastora	*P. geniculatus*	2010	Female	Positive	Yes
1336	VE132910	DC	Libertador	La Pastora	*P. geniculatus*	2010	Female	Positive	Yes
1337	VE134110	DC	Libertador	La Pastora	*P. geniculatus*	2010	Female	Positive	Yes
1338	VE127710	DC	Libertador	La Pastora	*P. geniculatus*	2010	Female	Positive	Yes
1339	VE63610	DC	Libertador	La Pastora	*P. geniculatus*	2010	Female	Positive	Yes
1340	VE104010	DC	Libertador	La Pastora	*P. geniculatus*	2010	Female	Positive	Yes
1341	VE28210	DC	Libertador	La Pastora	*P. geniculatus*	2010	Female	Positive	Yes
1342	VE38310	DC	Libertador	La Pastora	*P. geniculatus*	2010	Female	Positive	Yes

1343	VE52010	DC	Libertador	La Pastora	*P. geniculatus*	2010	Female	Positive	Yes
1344	VE104710	DC	Libertador	La Pastora	*P. geniculatus*	2010	Female	Positive	Yes
1345	VE114910	DC	Libertador	La Pastora	*P. geniculatus*	2010	Female	Positive	Yes
1346	VE118310	DC	Libertador	La Pastora	*P. geniculatus*	2010	Female	Positive	Yes
1347	VE122010	DC	Libertador	La Pastora	*P. geniculatus*	2010	Female	Positive	Yes
1348	VE7610	DC	Libertador	La Pastora	*P. geniculatus*	2010	Female	Positive	Yes
1349	VE22610	DC	Libertador	La Pastora	*P. geniculatus*	2010	Female	Positive	Yes
1350	VE62810	DC	Libertador	La Pastora	*P. geniculatus*	2010	Female	Positive	Yes
1351	VE44610	DC	Libertador	La Pastora	*P. geniculatus*	2010	Female	Positive	Yes
1352	VE119810B	DC	Libertador	La Pastora	*P. geniculatus*	2010	Female	Positive	Yes
1353	VE23110	DC	Libertador	La Pastora	*P. geniculatus*	2010	Female	Positive	Yes
1354	VE116510	DC	Libertador	La Pastora	*P. geniculatus*	2010	Female	NA	NA
1355	VE10010	DC	Libertador	La Pastora	*P. geniculatus*	2010	Female	Negative	No
1356	VE18510	DC	Libertador	La Pastora	*P. geniculatus*	2010	Female	Negative	No
1357	VE9610	DC	Libertador	La Pastora	*P. geniculatus*	2010	Female	Negative	No
1358	VE30810	DC	Libertador	La Pastora	*P. geniculatus*	2010	Female	Negative	No
1359	VE22110	DC	Libertador	La Pastora	*P. geniculatus*	2010	Female	Negative	Yes
1360	VE115110	DC	Libertador	La Pastora	*P. geniculatus*	2010	Female	NA	No
1361	VE46710	DC	Libertador	La Pastora	*P. geniculatus*	2010	Female	NA	No
1362	VE98010A	DC	Libertador	La Pastora	*P. geniculatus*	2010	Male	NA	No
1363	VE98010B	DC	Libertador	La Pastora	*P. geniculatus*	2010	Female	NA	No
1364	VE86210	DC	Libertador	La Pastora	*P. geniculatus*	2010	Female	NA	Yes
1365	VE76210	DC	Libertador	La Pastora	*P. geniculatus*	2010	Female	NA	Yes
1366	VE46010	DC	Libertador	La Pastora	*P. geniculatus*	2010	Male	NA	Yes
1367	VE11810	DC	Libertador	La Pastora	*P. geniculatus*	2010	Male	Negative	No
1368	VE86810	DC	Libertador	La Pastora	*P. geniculatus*	2010	Male	Negative	No
1369	VE103610	DC	Libertador	La Pastora	*P. geniculatus*	2010	Male	Negative	No
1370	VE12010	DC	Libertador	La Pastora	*P. geniculatus*	2010	Male	Negative	No
1371	VE19610	DC	Libertador	La Pastora	*P. geniculatus*	2010	Male	Negative	No
1372	VE139410	DC	Libertador	La Pastora	*P. geniculatus*	2010	Male	Positive	No
1373	VE136810	DC	Libertador	La Pastora	*P. geniculatus*	2010	Male	Positive	No
1374	VE132110	DC	Libertador	La Pastora	*P. geniculatus*	2010	Male	Positive	No
1375	VE123110	DC	Libertador	La Pastora	*P. geniculatus*	2010	Male	Positive	No
1376	VE91810	DC	Libertador	La Pastora	*P. geniculatus*	2010	Male	Positive	No
1377	VE48110	DC	Libertador	La Pastora	*P. geniculatus*	2010	Male	Positive	No
1378	VE42210	DC	Libertador	La Pastora	*P. geniculatus*	2010	Male	Positive	No
1379	VE63410	DC	Libertador	La Pastora	*P. geniculatus*	2010	Male	Positive	No
1380	VE79210	DC	Libertador	La Pastora	*P. geniculatus*	2010	Male	Positive	No
1381	VE63810A	DC	Libertador	La Pastora	*P. geniculatus*	2010	Male	NA	NA
1382	VE63810B	DC	Libertador	La Pastora	*P. geniculatus*	2010	Male	Positive	No
1383	VE123010	DC	Libertador	La Pastora	*P. geniculatus*	2010	Male	Positive	No
1384	VE18310	DC	Libertador	La Pastora	*P. geniculatus*	2010	Male	Positive	No

1385	VE84210	DC	Libertador	La Pastora	*P. geniculatus*	2010	Male	Positive	No
1386	VE50210	DC	Libertador	La Pastora	*P. geniculatus*	2010	Male	Positive	No
1387	VE85110	DC	Libertador	La Pastora	*P. geniculatus*	2010	Male	Positive	No
1388	VE62410A	DC	Libertador	La Pastora	*P. geniculatus*	2010	Male	Negative	No
1389	VE62410B	DC	Libertador	La Pastora	*P. geniculatus*	2010	Male	Positive	No
1390	VE79310	DC	Libertador	La Pastora	*P. geniculatus*	2010	Male	Positive	No
1391	VE59310	DC	Libertador	La Pastora	*P. geniculatus*	2010	Male	Positive	No
1392	VE61610A	DC	Libertador	La Pastora	*P. geniculatus*	2010	Female	Negative	No
1393	VE61610B	DC	Libertador	La Pastora	*P. geniculatus*	2010	Male	Positive	No
1394	VE16810	DC	Libertador	La Pastora	*P. geniculatus*	2010	Male	Positive	No
1395	VE53810	DC	Libertador	La Pastora	*P. geniculatus*	2010	Male	Positive	No
1396	VE90110	DC	Libertador	La Pastora	*P. geniculatus*	2010	Male	Positive	No
1397	VE67910	DC	Libertador	La Pastora	*P. geniculatus*	2010	Male	Positive	No
1398	VE18810	DC	Libertador	La Pastora	*P. geniculatus*	2010	Male	Positive	No
1399	VE100110	DC	Libertador	La Pastora	*P. geniculatus*	2010	Male	Positive	No
1400	VE52310	DC	Libertador	La Pastora	*P. geniculatus*	2010	Male	Positive	Yes
1401	VE101310	DC	Libertador	La Pastora	*P. geniculatus*	2010	Male	Positive	Yes
1402	VE84910A	DC	Libertador	La Pastora	*P. geniculatus*	2010	Male	Positive	Yes
1403	VE84910B	DC	Libertador	La Pastora	*P. geniculatus*	2010	Male	Positive	Yes
1404	VE95310	DC	Libertador	La Pastora	*P. geniculatus*	2010	Male	Positive	Yes
1405	VE59710A	DC	Libertador	La Pastora	*P. geniculatus*	2010	Female	Positive	No
1406	VE59710B	DC	Libertador	La Pastora	*P. geniculatus*	2010	Male	Positive	Yes
1407	VE99210	DC	Libertador	La Pastora	*P. geniculatus*	2010	Male	Positive	Yes
1408	VE50710	DC	Libertador	La Pastora	*P. geniculatus*	2010	Male	Positive	Yes
1409	VE119810A	DC	Libertador	La Pastora	*P. geniculatus*	2010	Male	Positive	Yes
1410	VE6710	DC	Libertador	La Pastora	*P. geniculatus*	2010	Male	NA	Yes
1411	VE23910	DC	Libertador	La Pastora	*P. geniculatus*	2010	Male	NA	Yes
1412	VE120310A	DC	Libertador	La Pastora	*P. geniculatus*	2010	Male	NA	NA
1413	VE140810	DC	Libertador	La Pastora	*P. geniculatus*	2010	Male	NA	Yes
1414	VE13610	DC	Libertador	La Pastora	*P. geniculatus*	2010	Male	Negative	No
1415	VE18710	DC	Libertador	La Pastora	*P. geniculatus*	2010	Male	Negative	No
1416	VE37510	DC	Libertador	La Pastora	*P. geniculatus*	2010	Male	Negative	No
1417	VE93810	DC	Libertador	La Pastora	*P. geniculatus*	2010	Male	NA	No
1418	VE53510	DC	Libertador	La Pastora	*P. geniculatus*	2010	Male	NA	NA
1419	VE51110A	DC	Libertador	La Pastora	*P. geniculatus*	2010	Male	NA	NA
1420	VE27410	DC	Libertador	La Pastora	*P. geniculatus*	2010	Nymph III	Negative	Yes
1421	VE82010	DC	Libertador	La Vega	*P. geniculatus*	2010	Female	Negative	No
1422	VE54110	DC	Libertador	La Vega	*P. geniculatus*	2010	Female	Negative	Yes
1423	VE137810	DC	Libertador	La Vega	*P. geniculatus*	2010	Female	Positive	No
1424	VE95110	DC	Libertador	La Vega	*P. geniculatus*	2010	Female	Positive	No
1425	VE25810A	DC	Libertador	La Vega	*P. geniculatus*	2010	Male	Negative	No
1426	VE25810B	DC	Libertador	La Vega	*P. geniculatus*	2010	Female	Positive	Yes

1427	VE5910	DC	Libertador	La Vega	*P. geniculatus*	2010	Female	Positive	Yes
1428	VE84110	DC	Libertador	La Vega	*P. geniculatus*	2010	Female	Positive	Yes
1429	VE21010	DC	Libertador	La Vega	*P. geniculatus*	2010	Female	Positive	Yes
1430	VE5410	DC	Libertador	La Vega	*P. geniculatus*	2010	Female	NA	No
1431	VE54810	DC	Libertador	La Vega	*P. geniculatus*	2010	Female	NA	No
1432	VE61110A	DC	Libertador	La Vega	*P. geniculatus*	2010	Male	NA	Yes
1433	VE61110B	DC	Libertador	La Vega	*P. geniculatus*	2010	Male	NA	Yes
1434	VE114810	DC	Libertador	La Vega	*P. geniculatus*	2010	Male	Negative	No
1435	VE80510	DC	Libertador	La Vega	*P. geniculatus*	2010	Male	Positive	No
1436	VE108810	DC	Libertador	La Vega	*P. geniculatus*	2010	Male	Positive	No
1437	VE55410	DC	Libertador	La Vega	*P. geniculatus*	2010	Male	Positive	No
1438	VE129210	DC	Libertador	La Vega	*P. geniculatus*	2010	Male	Positive	No
1439	VE137910	DC	Libertador	La Vega	*P. geniculatus*	2010	Male	Positive	Yes
1440	VE25010L224	DC	Libertador	La Vega	*P. geniculatus*	2010	Male	Positive	Yes
1441	VE35310	DC	Libertador	La Vega	*P. geniculatus*	2010	Male	Negative	No
1442	VE87610A	DC	Libertador	La Vega	*T. maculata*	2010	Female	Negative	No
1443	VE87610B	DC	Libertador	La Vega	*T. maculata*	2010	Female	Positive	No
1444	VE63210	DC	Libertador	Macarao	*P. geniculatus*	2010	Female	Positive	No
1445	VE54410	DC	Libertador	Macarao	*P. geniculatus*	2010	Female	Positive	No
1446	VE49910B	DC	Libertador	Macarao	*P. geniculatus*	2010	Female	Positive	No
1447	VE74610	DC	Libertador	Macarao	*P. geniculatus*	2010	Female	Positive	Yes
1448	VE63510	DC	Libertador	Macarao	*P. geniculatus*	2010	Male	Negative	No
1449	VE61710	DC	Libertador	Macarao	*P. geniculatus*	2010	Male	Positive	No
1450	VE89310	DC	Libertador	San Agustín	*P. geniculatus*	2010	Female	Negative	Yes
1451	VE69410	DC	Libertador	San Agustín	*P. geniculatus*	2010	Female	Positive	No
1452	VE129810	DC	Libertador	San Agustín	*P. geniculatus*	2010	Female	Positive	Yes
1453	VE57110	DC	Libertador	San Agustín	*P. geniculatus*	2010	Female	Positive	Yes
1454	VE0310	DC	Libertador	San Agustín	*P. geniculatus*	2010	Female	Positive	Yes
1455	VE126710	DC	Libertador	San Agustín	*P. geniculatus*	2010	Female	Positive	Yes
1456	VE106410	DC	Libertador	San Agustín	*P. geniculatus*	2010	Male	NA	No
1457	VE0210	DC	Libertador	San Agustín	*P. geniculatus*	2010	Male	Negative	No
1458	VE50010	DC	Libertador	San Agustín	*P. geniculatus*	2010	Male	Positive	No
1459	VE118510	DC	Libertador	San Agustín	*P. geniculatus*	2010	Male	Positive	Yes
1460	VE35910	DC	Libertador	San Bernardino	*P. geniculatus*	2010	Female	Positive	No
1461	VE20610	DC	Libertador	San Bernardino	*P. geniculatus*	2010	Female	Negative	No
1462	VE97510	DC	Libertador	San Bernardino	*P. geniculatus*	2010	Male	Positive	No
1463	VE56910	DC	Libertador	San José	*P. geniculatus*	2010	Female	NA	Yes
1464	VE20310	DC	Libertador	San José	*P. geniculatus*	2010	Female	Negative	Yes
1465	VE99710	DC	Libertador	San José	*P. geniculatus*	2010	Female	Positive	No
1466	VE119910	DC	Libertador	San José	*P. geniculatus*	2010	Female	Positive	No
1467	VE84010	DC	Libertador	San José	*P. geniculatus*	2010	Female	Positive	Yes
1468	VE110210	DC	Libertador	San José	*P. geniculatus*	2010	Female	Positive	Yes

1469	VE26010	DC	Libertador	San José	*P. geniculatus*	2010	Female	Positive	Yes
1470	VE94110	DC	Libertador	San José	*P. geniculatus*	2010	Female	Positive	Yes
1471	VE57710	DC	Libertador	San José	*P. geniculatus*	2010	Female	Positive	Yes
1472	VE128410	DC	Libertador	San José	*P. geniculatus*	2010	Male	Positive	No
1473	VE107110	DC	Libertador	San José	*P. geniculatus*	2010	Male	Positive	No
1474	VE96010	DC	Libertador	San José	*P. geniculatus*	2010	Male	Positive	No
1475	VE84610	DC	Libertador	San José	*P. geniculatus*	2010	Male	Positive	No
1476	VE42010	DC	Libertador	San José	*P. geniculatus*	2010	Male	Positive	No
1477	VE60410	DC	Libertador	San José	*P. geniculatus*	2010	Male	Positive	No
1478	VE49410	DC	Libertador	San José	*P. geniculatus*	2010	Male	Positive	No
1479	VE6210	DC	Libertador	San José	*P. geniculatus*	2010	Male	NA	No
1480	VE55710	DC	Libertador	San Pedro	*P. geniculatus*	2010	Female	NA	No
1481	VE106710	DC	Libertador	San Pedro	*P. geniculatus*	2010	Female	Positive	Yes
1482	VE100410	DC	Libertador	San Pedro	*P. geniculatus*	2010	Female	Positive	Yes
1483	VE119110	DC	Libertador	San Pedro	*P. geniculatus*	2010	Female	Positive	Yes
1484	VE15110A	DC	Libertador	San Pedro	*P. geniculatus*	2010	Female	NA	Yes
1485	VE15110B	DC	Libertador	San Pedro	*P. geniculatus*	2010	Male	NA	Yes
1486	VE19010	DC	Libertador	San Pedro	*P. geniculatus*	2010	Female	NA	No
1487	VE3110	DC	Libertador	San Pedro	*T. maculata*	2010	Female	Positive	No
1488	VE131210	DC	Libertador	San Pedro	*P. geniculatus*	2010	Male	Negative	No
1489	VE49810	DC	Libertador	San Pedro	*P. geniculatus*	2010	Male	Negative	No
1490	VE143210	DC	Libertador	San Pedro	*P. geniculatus*	2010	Male	Positive	No
1491	VE114210	DC	Libertador	San Pedro	*P. geniculatus*	2010	Male	Positive	No
1492	VE49010	DC	Libertador	San Pedro	*P. geniculatus*	2010	Male	Positive	No
1493	VE70410	DC	Libertador	San Pedro	*P. geniculatus*	2010	Male	Positive	Yes
1494	VE36610	DC	Libertador	San Pedro	*P. geniculatus*	2010	Male	NA	Yes
1495	VE62110	DC	Libertador	Santa Rosalía	*P. geniculatus*	2010	Female	Negative	No
1496	VE80710	DC	Libertador	Santa Rosalía	*P. geniculatus*	2010	Female	Positive	No
1497	VE107210	DC	Libertador	Santa Rosalía	*P. geniculatus*	2010	Female	Positive	Yes
1498	VE68510	DC	Libertador	Santa Rosalía	*P. geniculatus*	2010	Female	Positive	Yes
1499	VE114510	DC	Libertador	Santa Rosalía	*P. geniculatus*	2010	Female	Positive	Yes
1500	VE37110	DC	Libertador	Santa Rosalía	*P. geniculatus*	2010	Male	NA	No
1501	VE65110	DC	Libertador	Sucre	*P. geniculatus*	2010	Male	NA	NA
1502	VE64810	DC	Libertador	Sucre	*P. geniculatus*	2010	Nymph III	Positive	No
1503	VE82410	DC	Libertador	Sucre	*P. geniculatus*	2010	Female	NA	Yes
1504	VE76410	DC	Libertador	Sucre	*P. geniculatus*	2010	Female	NA	Yes
1505	VE19510	DC	Libertador	Sucre	*P. geniculatus*	2010	Female	Negative	No
1506	VE141210	DC	Libertador	Sucre	*P. geniculatus*	2010	Female	Negative	Yes
1507	VE17710	DC	Libertador	Sucre	*P. geniculatus*	2010	Female	Negative	Yes
1508	VE28110	DC	Libertador	Sucre	*P. geniculatus*	2010	Female	Negative	Yes
1509	VE99410	DC	Libertador	Sucre	*P. geniculatus*	2010	Female	Negative	Yes
1510	VE131410	DC	Libertador	Sucre	*P. geniculatus*	2010	Female	Positive	No

1511	VE30710	DC	Libertador	Sucre	*P. geniculatus*	2010	Female	Positive	No
1512	VE44010	DC	Libertador	Sucre	*P. geniculatus*	2010	Female	Positive	No
1513	VE13310	DC	Libertador	Sucre	*P. geniculatus*	2010	Female	Positive	No
1514	VE58210	DC	Libertador	Sucre	*P. geniculatus*	2010	Female	Positive	No
1515	VE124010	DC	Libertador	Sucre	*P. geniculatus*	2010	Female	Positive	No
1516	VE115610	DC	Libertador	Sucre	*P. geniculatus*	2010	Female	Positive	No
1517	VE83810	DC	Libertador	Sucre	*P. geniculatus*	2010	Female	Positive	No
1518	VE111710B	DC	Libertador	Sucre	*P. geniculatus*	2010	Female	Positive	No
1519	VE133910	DC	Libertador	Sucre	*P. geniculatus*	2010	Female	Positive	Yes
1520	VE129610	DC	Libertador	Sucre	*P. geniculatus*	2010	Female	Positive	Yes
1521	VE141010	DC	Libertador	Sucre	*P. geniculatus*	2010	Female	Positive	Yes
1522	VE138410B	DC	Libertador	Sucre	*P. geniculatus*	2010	Female	Positive	Yes
1523	VE130210	DC	Libertador	Sucre	*P. geniculatus*	2010	Female	Positive	Yes
1524	VE21710	DC	Libertador	Sucre	*P. geniculatus*	2010	Female	Positive	Yes
1525	VE89110	DC	Libertador	Sucre	*P. geniculatus*	2010	Female	Positive	Yes
1526	VE75410	DC	Libertador	Sucre	*P. geniculatus*	2010	Female	Positive	Yes
1527	VE82310	DC	Libertador	Sucre	*P. geniculatus*	2010	Female	Positive	Yes
1528	VE36910	DC	Libertador	Sucre	*P. geniculatus*	2010	Female	Positive	Yes
1529	VE110710	DC	Libertador	Sucre	*P. geniculatus*	2010	Female	Positive	Yes
1530	VE85010	DC	Libertador	Sucre	*P. geniculatus*	2010	Female	Positive	Yes
1531	VE29910	DC	Libertador	Sucre	*P. geniculatus*	2010	Female	Positive	Yes
1532	VE4310A	DC	Libertador	Sucre	*P. geniculatus*	2010	Male	Positive	No
1533	VE83310A	DC	Libertador	Sucre	*P. geniculatus*	2010	Male	Positive	No
1534	VE83310B	DC	Libertador	Sucre	*P. geniculatus*	2010	Female	Positive	Yes
1535	VE83310C	DC	Libertador	Sucre	*P. geniculatus*	2010	Female	Positive	Yes
1536	VE83310D	DC	Libertador	Sucre	*P. geniculatus*	2010	Female	Positive	Yes
1537	VE78910	DC	Libertador	Sucre	*P. geniculatus*	2010	Female	Positive	Yes
1538	VE50810A	DC	Libertador	Sucre	*P. geniculatus*	2010	Female	NA	NA
1539	VE50810B	DC	Libertador	Sucre	*P. geniculatus*	2010	Female	NA	NA
1540	VE50810C	DC	Libertador	Sucre	*P. geniculatus*	2010	Female	Positive	Yes
1541	VE126210	DC	Libertador	Sucre	*P. geniculatus*	2010	Female	Positive	Yes
1542	VE26110A	DC	Libertador	Sucre	*P. geniculatus*	2010	Female	NA	Yes
1543	VE26110B	DC	Libertador	Sucre	*P. geniculatus*	2010	Female	Positive	Yes
1544	VE19110	DC	Libertador	Sucre	*P. geniculatus*	2010	Female	Positive	Yes
1545	VE24710	DC	Libertador	Sucre	*P. geniculatus*	2010	Female	Positive	Yes
1546	VE32510A	DC	Libertador	Sucre	*P. geniculatus*	2010	Male	Negative	Yes
1547	VE32510B	DC	Libertador	Sucre	*P. geniculatus*	2010	Female	Positive	Yes
1548	VE32510C	DC	Libertador	Sucre	*P. geniculatus*	2010	Female	Positive	Yes
1549	VE7110	DC	Libertador	Sucre	*P. geniculatus*	2010	Female	Positive	Yes
1550	VE108410	DC	Libertador	Sucre	*P. geniculatus*	2010	Female	Positive	Yes
1551	VE119610	DC	Libertador	Sucre	*P. geniculatus*	2010	Female	Positive	Yes
1552	VE102510	DC	Libertador	Sucre	*P. geniculatus*	2010	Female	Positive	Yes

1553	VE51510	DC	Libertador	Sucre	*P. geniculatus*	2010	Female	Positive	Yes
1554	VE51610	DC	Libertador	Sucre	*P. geniculatus*	2010	Female	Positive	Yes
1555	VE109310	DC	Libertador	Sucre	*P. geniculatus*	2010	Female	Positive	Yes
1556	VE111910	DC	Libertador	Sucre	*P. geniculatus*	2010	Female	Positive	Yes
1557	VE119410	DC	Libertador	Sucre	*P. geniculatus*	2010	Female	NA	Yes
1558	VE28810A	DC	Libertador	Sucre	*P. geniculatus*	2010	Female	NA	No
1559	VE28810B	DC	Libertador	Sucre	*P. geniculatus*	2010	Female	NA	No
1560	VE28010	DC	Libertador	Sucre	*P. geniculatus*	2010	Female	NA	Yes
1561	VE22510	DC	Libertador	Sucre	*P. geniculatus*	2010	Female	NA	Yes
1562	VE44810	DC	Libertador	Sucre	*P. geniculatus*	2010	Female	NA	Yes
1563	VE135510	DC	Libertador	Sucre	*P. geniculatus*	2010	Female	NA	NA
1564	VE127310B	DC	Libertador	Sucre	*P. geniculatus*	2010	Female	NA	NA
1565	VE5010	DC	Libertador	Sucre	*P. geniculatus*	2010	Female	NA	No
1566	VE36210	DC	Libertador	Sucre	*P. geniculatus*	2010	Female	NA	No
1567	VE20010	DC	Libertador	Sucre	*P. geniculatus*	2010	Female	NA	No
1568	VE3210A	DC	Libertador	Sucre	*P. geniculatus*	2010	Female	Positive	Yes
1569	VE3210B	DC	Libertador	Sucre	*P. geniculatus*	2010	Female	NA	NA
1570	VE60110	DC	Libertador	Sucre	*P. geniculatus*	2010	Female	NA	NA
1571	VE69710	DC	Libertador	Sucre	*P. geniculatus*	2010	Male	NA	No
1572	VE132710	DC	Libertador	Sucre	*P. geniculatus*	2010	Male	NA	NA
1573	VE46910	DC	Libertador	Sucre	*P. geniculatus*	2010	Male	Negative	No
1574	VE100210	DC	Libertador	Sucre	*P. geniculatus*	2010	Male	Negative	No
1575	VE62210	DC	Libertador	Sucre	*P. geniculatus*	2010	Male	Negative	No
1576	VE31410	DC	Libertador	Sucre	*P. geniculatus*	2010	Male	Negative	Yes
1577	VE97710	DC	Libertador	Sucre	*P. geniculatus*	2010	Male	Negative	Yes
1578	VE10110	DC	Libertador	Sucre	*P. geniculatus*	2010	Male	Negative	Yes
1579	VE40610	DC	Libertador	Sucre	*P. geniculatus*	2010	Male	Negative	Yes
1580	VE121410	DC	Libertador	Sucre	*P. geniculatus*	2010	Male	Negative	Yes
1581	VE138110	DC	Libertador	Sucre	*P. geniculatus*	2010	Male	Positive	No
1582	VE143110	DC	Libertador	Sucre	*P. geniculatus*	2010	Male	Positive	No
1583	VE131810	DC	Libertador	Sucre	*P. geniculatus*	2010	Male	Positive	No
1584	VE143710	DC	Libertador	Sucre	*P. geniculatus*	2010	Male	Positive	No
1585	VE83110	DC	Libertador	Sucre	*P. geniculatus*	2010	Male	Positive	No
1586	VE55210	DC	Libertador	Sucre	*P. geniculatus*	2010	Male	Positive	No
1587	VE67610	DC	Libertador	Sucre	*P. geniculatus*	2010	Male	Positive	No
1588	VE56310	DC	Libertador	Sucre	*P. geniculatus*	2010	Male	Positive	No
1589	VE100510	DC	Libertador	Sucre	*P. geniculatus*	2010	Male	Positive	No
1590	VE48610	DC	Libertador	Sucre	*P. geniculatus*	2010	Male	Positive	No
1591	VE87010	DC	Libertador	Sucre	*P. geniculatus*	2010	Male	Positive	No
1592	VE113410	DC	Libertador	Sucre	*P. geniculatus*	2010	Male	Positive	No
1593	VE116010	DC	Libertador	Sucre	*P. geniculatus*	2010	Male	Positive	No
1594	VE52210	DC	Libertador	Sucre	*P. geniculatus*	2010	Male	Positive	No

1595	VE110010	DC	Libertador	Sucre	*P. geniculatus*	2010	Male	Positive	No
1596	VE60710	DC	Libertador	Sucre	*P. geniculatus*	2010	Male	Positive	No
1597	VE42110	DC	Libertador	Sucre	*P. geniculatus*	2010	Male	Positive	No
1598	VE99110	DC	Libertador	Sucre	*P. geniculatus*	2010	Male	Positive	No
1599	VE80210	DC	Libertador	Sucre	*P. geniculatus*	2010	Male	Positive	No
1600	VE83610A	DC	Libertador	Sucre	*P. geniculatus*	2010	Male	Positive	No
1601	VE83610B	DC	Libertador	Sucre	*P. geniculatus*	2010	Male	Positive	No
1602	VE115510	DC	Libertador	Sucre	*P. geniculatus*	2010	Male	Positive	No
1603	VE54210	DC	Libertador	Sucre	*P. geniculatus*	2010	Male	Positive	No
1604	VE123810	DC	Libertador	Sucre	*P. geniculatus*	2010	Male	Positive	No
1605	VE12510A	DC	Libertador	Sucre	*P. geniculatus*	2010	Female	Positive	No
1606	VE12510B	DC	Libertador	Sucre	*P. geniculatus*	2010	Male	Positive	No
1607	VE31810	DC	Libertador	Sucre	*P. geniculatus*	2010	Male	Positive	No
1608	VE63310	DC	Libertador	Sucre	*P. geniculatus*	2010	Male	Positive	No
1609	VE67110	DC	Libertador	Sucre	*P. geniculatus*	2010	Male	Positive	No
1610	VE104110	DC	Libertador	Sucre	*P. geniculatus*	2010	Male	Positive	No
1611	VE114410	DC	Libertador	Sucre	*P. geniculatus*	2010	Male	Positive	No
1612	VE121510	DC	Libertador	Sucre	*P. geniculatus*	2010	Male	Positive	Yes
1613	VE25410	DC	Libertador	Sucre	*P. geniculatus*	2010	Male	Positive	Yes
1614	VE77510	DC	Libertador	Sucre	*P. geniculatus*	2010	Male	Positive	Yes
1615	VE60310	DC	Libertador	Sucre	*P. geniculatus*	2010	Male	Positive	Yes
1616	VE88910	DC	Libertador	Sucre	*P. geniculatus*	2010	Male	Positive	Yes
1617	VE98110A	DC	Libertador	Sucre	*P. geniculatus*	2010	Male	NA	NA
1618	VE98110B	DC	Libertador	Sucre	*P. geniculatus*	2010	Male	Positive	Yes
1619	VE91310	DC	Libertador	Sucre	*P. geniculatus*	2010	Male	Positive	Yes
1620	VE60210	DC	Libertador	Sucre	*P. geniculatus*	2010	Male	Positive	Yes
1621	VE102410	DC	Libertador	Sucre	*P. geniculatus*	2010	Male	Positive	Yes
1622	VE2010	DC	Libertador	Sucre	*P. geniculatus*	2010	Male	Positive	Yes
1623	VE111210	DC	Libertador	Sucre	*P. geniculatus*	2010	Male	Positive	Yes
1624	VE81910	DC	Libertador	Sucre	*P. geniculatus*	2010	Male	Positive	Yes
1625	VE35810	DC	Libertador	Sucre	*P. geniculatus*	2010	Male	Positive	Yes
1626	VE110310	DC	Libertador	Sucre	*P. geniculatus*	2010	Male	Positive	Yes
1627	VE30110	DC	Libertador	Sucre	*P. geniculatus*	2010	Male	Positive	No
1628	VE2810	DC	Libertador	Sucre	*P. geniculatus*	2010	Male	NA	Yes
1629	VE41010	DC	Libertador	Sucre	*P. geniculatus*	2010	Male	NA	NA
1630	VE127310A	DC	Libertador	Sucre	*P. geniculatus*	2010	Male	NA	NA
1631	VE70210	DC	Libertador	Sucre	*P. geniculatus*	2010	Male	NA	No
1632	VE118810A	DC	Libertador	Sucre	*P. geniculatus*	2010	Nymph III	Positive	Yes
1633	VE118810B	DC	Libertador	Sucre	*P. geniculatus*	2010	Nymph III	Positive	Yes
1634	VE125310	DC	Libertador	Sucre	*P. geniculatus*	2010	Nymph IV	Negative	Yes
1635	VE10710	DC	Libertador	Sucre	*P. geniculatus*	2010	Nymph III	NA	Yes
1636	VE44510	DC	Libertador	Sucre	*P. geniculatus*	2010	Nymph V	Negative	Yes

1637	VE4010	DC	Libertador	Sucre	*P. geniculatus*	2010	Nymph V	Positive	Yes
1638	VE99510	DC	Libertador	Sucre	*P. geniculatus*	2010	Nymph V	Positive	Yes
1639	VE105310	DC	Libertador	Sucre	*P. geniculatus*	2010	Nymph IV	Positive	No
1640	VE110110	Miranda	Sucre	Caucagüita	*P. geniculatus*	2010	Female	Positive	No
1641	VE82810	Miranda	Sucre	Caucagüita	*P. geniculatus*	2010	Female	Negative	Yes
1642	VE132510	Miranda	Sucre	Caucagüita	*P. geniculatus*	2010	Female	Positive	No
1643	VE105810	Miranda	Sucre	Caucagüita	*P. geniculatus*	2010	Female	Positive	No
1644	VE35210	Miranda	Sucre	Caucagüita	*P. geniculatus*	2010	Female	Positive	No
1645	VE0710	Miranda	Sucre	Caucagüita	*P. geniculatus*	2010	Female	Positive	No
1646	VE118910	Miranda	Sucre	Caucagüita	*P. geniculatus*	2010	Female	Positive	No
1647	VE113310	Miranda	Sucre	Caucagüita	*P. geniculatus*	2010	Female	Positive	No
1648	VE140010	Miranda	Sucre	Caucagüita	*P. geniculatus*	2010	Female	Positive	Yes
1649	VE136010	Miranda	Sucre	Caucagüita	*P. geniculatus*	2010	Female	Positive	Yes
1650	VE66210	Miranda	Sucre	Caucagüita	*P. geniculatus*	2010	Female	Positive	Yes
1651	VE55010A	Miranda	Sucre	Caucagüita	*P. geniculatus*	2010	Male	Negative	Yes
1652	VE55010B	Miranda	Sucre	Caucagüita	*P. geniculatus*	2010	Female	Positive	Yes
1653	VE36010	Miranda	Sucre	Caucagüita	*P. geniculatus*	2010	Female	Positive	Yes
1654	VE12710	Miranda	Sucre	Caucagüita	*P. geniculatus*	2010	Female	Positive	Yes
1655	VE69210	Miranda	Sucre	Caucagüita	*P. geniculatus*	2010	Female	Positive	Yes
1656	VE103110	Miranda	Sucre	Caucagüita	*P. geniculatus*	2010	Female	Positive	Yes
1657	VE14810	Miranda	Sucre	Caucagüita	*P. geniculatus*	2010	Female	NA	Yes
1658	VE131610	Miranda	Sucre	Caucagüita	*P. geniculatus*	2010	Male	Negative	Yes
1659	VE37310	Miranda	Sucre	Caucagüita	*P. geniculatus*	2010	Male	Positive	No
1660	VE84710	Miranda	Sucre	Caucagüita	*P. geniculatus*	2010	Male	Positive	No
1661	VE65310	Miranda	Sucre	Caucagüita	*P. geniculatus*	2010	Male	Positive	No
1662	VE961010	Miranda	Sucre	Caucagüita	*P. geniculatus*	2010	Male	Positive	No
1663	VE139610	Miranda	Sucre	Caucagüita	*P. geniculatus*	2010	Male	Positive	Yes
1664	VE24910A	Miranda	Sucre	Caucagüita	*P. geniculatus*	2010	Female	Positive	Yes
1665	VE24910B	Miranda	Sucre	Caucagüita	*P. geniculatus*	2010	Male	Positive	Yes
1666	VE89510	Miranda	Sucre	Caucagüita	*P. geniculatus*	2010	Male	Positive	Yes
1667	VE39110	Miranda	Sucre	Caucagüita	*P. geniculatus*	2010	Male	Positive	Yes
1668	VE26810	Miranda	Sucre	Caucagüita	*P. geniculatus*	2010	Male	NA	No
1669	VE112710	Miranda	Sucre	Caucagüita	*P. geniculatus*	2010	Male	NA	No
1670	VE120810	Miranda	Chacao	Chacao	*P. geniculatus*	2010	Female	Negative	Yes
1671	VE97010	Miranda	Chacao	Chacao	*P. geniculatus*	2010	Female	Positive	Yes
1672	VE85510	Miranda	Chacao	Chacao	*P. geniculatus*	2010	Female	Positive	Yes
1673	VE40710	Miranda	Chacao	Chacao	*P. geniculatus*	2010	Female	NA	No
1674	VE103910	Miranda	Chacao	Chacao	*P. geniculatus*	2010	Male	NA	No
1675	VE123710	Miranda	Chacao	Chacao	*P. geniculatus*	2010	Male	Positive	Yes
1676	VE100310	Miranda	Chacao	Chacao	*P. geniculatus*	2010	Male	Positive	Yes
1677	VE37610	Miranda	Chacao	Chacao	*P. geniculatus*	2010	Male	NA	NA
1678	VE9110	Miranda	Baruta	El Cafetal	*P. geniculatus*	2010	Female	Negative	No

1679	VE117910	Miranda	Baruta	El Cafetal	*P. geniculatus*	2010	Female	Negative	No
1680	VE40310	Miranda	Baruta	El Cafetal	*P. geniculatus*	2010	Female	Negative	Yes
1681	VE78510	Miranda	Baruta	El Cafetal	*P. geniculatus*	2010	Female	Negative	Yes
1682	VE88010	Miranda	Baruta	El Cafetal	*P. geniculatus*	2010	Female	Positive	No
1683	VE92010	Miranda	Baruta	El Cafetal	*P. geniculatus*	2010	Female	Positive	No
1684	VE19910	Miranda	Baruta	El Cafetal	*P. geniculatus*	2010	Female	Positive	No
1685	VE58710	Miranda	Baruta	El Cafetal	*P. geniculatus*	2010	Female	Positive	No
1686	VE108310A	Miranda	Baruta	El Cafetal	*P. geniculatus*	2010	Female	Positive	No
1687	VE108310B	Miranda	Baruta	El Cafetal	*P. geniculatus*	2010	Female	Positive	No
1688	VE125710	Miranda	Baruta	El Cafetal	*P. geniculatus*	2010	Female	Positive	No
1689	VE91410A	Miranda	Baruta	El Cafetal	*P. geniculatus*	2010	Female	Positive	No
1690	VE91410B	Miranda	Baruta	El Cafetal	*P. geniculatus*	2010	Female	Positive	No
1691	VE58510	Miranda	Baruta	El Cafetal	*P. geniculatus*	2010	Female	Positive	Yes
1692	VE87710	Miranda	Baruta	El Cafetal	*P. geniculatus*	2010	Female	Positive	Yes
1693	VE22410A	Miranda	Baruta	El Cafetal	*P. geniculatus*	2010	Female	Positive	Yes
1694	VE1810	Miranda	Baruta	El Cafetal	*P. geniculatus*	2010	Female	Positive	Yes
1695	VE34210A	Miranda	Baruta	El Cafetal	*P. geniculatus*	2010	Male	Positive	No
1696	VE34210B	Miranda	Baruta	El Cafetal	*P. geniculatus*	2010	Female	Positive	Yes
1697	VE120610	Miranda	Baruta	El Cafetal	*P. geniculatus*	2010	Female	NA	Yes
1698	VE53910	Miranda	Baruta	El Cafetal	*P. geniculatus*	2010	Female	NA	NA
1699	VE8810	Miranda	Baruta	El Cafetal	*P. geniculatus*	2010	Female	NA	No
1700	VE29110	Miranda	Baruta	El Cafetal	*P. geniculatus*	2010	Female	NA	No
1701	VE102710	Miranda	Baruta	El Cafetal	*P. geniculatus*	2010	Female	NA	No
1702	VE137310	Miranda	Baruta	El Cafetal	*P. geniculatus*	2010	Male	Negative	No
1703	VE32110	Miranda	Baruta	El Cafetal	*P. geniculatus*	2010	Male	Negative	No
1704	VE95910	Miranda	Baruta	El Cafetal	*P. geniculatus*	2010	Male	Negative	No
1705	VE22810	Miranda	Baruta	El Cafetal	*P. geniculatus*	2010	Male	Negative	No
1706	VE88610	Miranda	Baruta	El Cafetal	*P. geniculatus*	2010	Male	Negative	No
1707	VE63910	Miranda	Baruta	El Cafetal	*P. geniculatus*	2010	Male	Negative	No
1708	VE29710	Miranda	Baruta	El Cafetal	*P. geniculatus*	2010	Male	Negative	Yes
1709	VE135410	Miranda	Baruta	El Cafetal	*P. geniculatus*	2010	Male	Positive	No
1710	VE139010	Miranda	Baruta	El Cafetal	*P. geniculatus*	2010	Male	Positive	No
1711	VE42710	Miranda	Baruta	El Cafetal	*P. geniculatus*	2010	Male	Positive	No
1712	VE74210	Miranda	Baruta	El Cafetal	*P. geniculatus*	2010	Male	Positive	No
1713	VE92310	Miranda	Baruta	El Cafetal	*P. geniculatus*	2010	Male	Positive	No
1714	VE46810	Miranda	Baruta	El Cafetal	*P. geniculatus*	2010	Male	Positive	No
1715	VE77710	Miranda	Baruta	El Cafetal	*P. geniculatus*	2010	Male	Positive	No
1716	VE4210	Miranda	Baruta	El Cafetal	*P. geniculatus*	2010	Male	Positive	No
1717	VE82710	Miranda	Baruta	El Cafetal	*P. geniculatus*	2010	Male	Positive	No
1718	VE113510	Miranda	Baruta	El Cafetal	*P. geniculatus*	2010	Male	Positive	No
1719	VE133710	Miranda	Baruta	El Cafetal	*P. geniculatus*	2010	Male	Positive	Yes
1720	VE136410	Miranda	Baruta	El Cafetal	*P. geniculatus*	2010	Male	Positive	Yes

1721	VE133410	Miranda	Baruta	El Cafetal	*P. geniculatus*	2010	Male	Positive	Yes
1722	VE82510	Miranda	Baruta	El Cafetal	*P. geniculatus*	2010	Male	Positive	Yes
1723	VE6610A	Miranda	Baruta	El Cafetal	*P. geniculatus*	2010	Male	Positive	Yes
1724	VE3310B	Miranda	Baruta	El Cafetal	*P. geniculatus*	2010	Male	Positive	Yes
1725	VE44410	Miranda	Baruta	El Cafetal	*P. geniculatus*	2010	Male	NA	No
1726	VE14010	Miranda	Baruta	El Cafetal	*P. geniculatus*	2010	Male	NA	No
1727	VE112310	Miranda	Baruta	El Cafetal	*P. geniculatus*	2010	Male	NA	No
1728	VE132310	Miranda	Sucre	Filas de mariche	*P. geniculatus*	2010	Female	NA	NA
1729	VE41510	Miranda	Sucre	Filas de mariche	*P. geniculatus*	2010	Female	Negative	Yes
1730	VE105210	Miranda	Sucre	Filas de mariche	*P. geniculatus*	2010	Female	Negative	Yes
1731	VE8410	Miranda	Sucre	Filas de mariche	*P. geniculatus*	2010	Female	Negative	Yes
1732	VE107510	Miranda	Sucre	Filas de mariche	*P. geniculatus*	2010	Female	Negative	Yes
1733	VE143410A	Miranda	Sucre	Filas de mariche	*P. geniculatus*	2010	Female	Positive	No
1734	VE143410B	Miranda	Sucre	Filas de mariche	*P. geniculatus*	2010	Female	Positive	No
1735	VE61910	Miranda	Sucre	Filas de mariche	*P. geniculatus*	2010	Female	Positive	No
1736	VE52810	Miranda	Sucre	Filas de mariche	*P. geniculatus*	2010	Female	Positive	No
1737	VE116410	Miranda	Sucre	Filas de mariche	*P. geniculatus*	2010	Female	Positive	No
1738	VE79110	Miranda	Sucre	Filas de mariche	*P. geniculatus*	2010	Female	Positive	No
1739	VE125610	Miranda	Sucre	Filas de mariche	*P. geniculatus*	2010	Female	Positive	Yes
1740	VE134310	Miranda	Sucre	Filas de mariche	*P. geniculatus*	2010	Female	Positive	Yes
1741	VE112910	Miranda	Sucre	Filas de mariche	*P. geniculatus*	2010	Female	Positive	Yes
1742	VE38510	Miranda	Sucre	Filas de mariche	*P. geniculatus*	2010	Female	Positive	Yes
1743	VE104610	Miranda	Sucre	Filas de mariche	*P. geniculatus*	2010	Female	Positive	Yes
1744	VE79010	Miranda	Sucre	Filas de mariche	*P. geniculatus*	2010	Female	Positive	Yes
1745	VE57410	Miranda	Sucre	Filas de mariche	*P. geniculatus*	2010	Female	Positive	Yes
1746	VE6010	Miranda	Sucre	Filas de mariche	*P. geniculatus*	2010	Female	Positive	Yes
1747	VE123310	Miranda	Sucre	Filas de mariche	*P. geniculatus*	2010	Female	Positive	Yes
1748	VE4510	Miranda	Sucre	Filas de mariche	*P. geniculatus*	2010	Female	NA	No
1749	VE17810	Miranda	Sucre	Filas de mariche	*P. geniculatus*	2010	Female	NA	Yes
1750	VE134210	Miranda	Sucre	Filas de mariche	*P. geniculatus*	2010	Female	NA	NA
1751	VE11210	Miranda	Sucre	Filas de mariche	*P. geniculatus*	2010	Female	NA	Yes
1752	VE91510	Miranda	Sucre	Filas de mariche	*P. geniculatus*	2010	Female	NA	Yes
1753	VE45610	Miranda	Sucre	Filas de mariche	*P. geniculatus*	2010	Male	Negative	Yes
1754	VE121010	Miranda	Sucre	Filas de mariche	*P. geniculatus*	2010	Male	Negative	Yes
1755	VE70110	Miranda	Sucre	Filas de mariche	*P. geniculatus*	2010	Male	Positive	No
1756	VE96610	Miranda	Sucre	Filas de mariche	*P. geniculatus*	2010	Male	Positive	No
1757	VE51310	Miranda	Sucre	Filas de mariche	*P. geniculatus*	2010	Male	Positive	No
1758	VE79710	Miranda	Sucre	Filas de mariche	*P. geniculatus*	2010	Male	Positive	No
1759	VE12410	Miranda	Sucre	Filas de mariche	*P. geniculatus*	2010	Male	Positive	No
1760	VE13110	Miranda	Sucre	Filas de mariche	*P. geniculatus*	2010	Male	Positive	No
1761	VE79410	Miranda	Sucre	Filas de mariche	*P. geniculatus*	2010	Male	Positive	No
1762	VE43710A	Miranda	Sucre	Filas de mariche	*P. geniculatus*	2010	Male	Positive	No

1763	VE43710B	Miranda	Sucre	Filas de mariche	*P. geniculatus*	2010	Male	Positive	No
1764	VE134910	Miranda	Sucre	Filas de mariche	*P. geniculatus*	2010	Male	Positive	Yes
1765	VE139310	Miranda	Sucre	Filas de mariche	*P. geniculatus*	2010	Male	Positive	Yes
1766	VE108910	Miranda	Sucre	Filas de mariche	*P. geniculatus*	2010	Male	Positive	Yes
1767	VE56510	Miranda	Sucre	Filas de mariche	*P. geniculatus*	2010	Male	Positive	Yes
1768	VE5510	Miranda	Sucre	Filas de mariche	*P. geniculatus*	2010	Male	Positive	Yes
1769	VE70510	Miranda	Sucre	Filas de mariche	*P. geniculatus*	2010	Male	Positive	Yes
1770	VE104210	Miranda	Sucre	Filas de mariche	*P. geniculatus*	2010	Male	Positive	Yes
1771	VE33710	Miranda	Sucre	Filas de mariche	*P. geniculatus*	2010	Male	NA	No
1772	VE45010	Miranda	Sucre	Filas de mariche	*P. geniculatus*	2010	Male	NA	NA
1773	VE113010	Miranda	Sucre	Filas de mariche	*T. nigromaculata*	2010	Male	Negative	Yes
1774	VE91710A	Miranda	Sucre	Filas de mariche	*P. geniculatus*	2010	Male	Positive	No
1775	VE91710B	Miranda	Sucre	Filas de mariche	*T. nigromaculata*	2010	Male	Positive	Yes
1776	VE132210B	Miranda	Sucre	Filas de mariche	*P. geniculatus*	2010	Nymph IV	Positive	Yes
1777	VE132210C	Miranda	Sucre	Filas de mariche	*P. geniculatus*	2010	Nymph IV	Positive	Yes
1778	VE121310	Miranda	Sucre	Filas de mariche	*P. geniculatus*	2010	Female	NA	Yes
1779	VE4410B	Miranda	Sucre	Filas de mariche	*P. geniculatus*	2010	Female	Negative	No
1780	VE102010	Miranda	Sucre	Filas de mariche	*P. geniculatus*	2010	Female	Negative	Yes
1781	VE139510	Miranda	Sucre	Filas de mariche	*P. geniculatus*	2010	Female	Positive	No
1782	VE100910B	Miranda	Sucre	Filas de mariche	*P. geniculatus*	2010	Female	Positive	No
1783	VE49510	Miranda	Sucre	Filas de mariche	*P. geniculatus*	2010	Female	Positive	No
1784	VE109910	Miranda	Sucre	Filas de mariche	*P. geniculatus*	2010	Female	Positive	No
1785	VE132210A	Miranda	Sucre	Filas de mariche	*P. geniculatus*	2010	Female	Positive	Yes
1786	VE135010A	Miranda	Sucre	Filas de mariche	*P. geniculatus*	2010	Female	Positive	Yes
1787	VE136710	Miranda	Sucre	Filas de mariche	*P. geniculatus*	2010	Female	Positive	Yes
1788	VE64610A	Miranda	Sucre	Filas de mariche	*P. geniculatus*	2010	Male	Positive	No
1789	VE64610B	Miranda	Sucre	Filas de mariche	*P. geniculatus*	2010	Female	Positive	No
1790	VE64610C	Miranda	Sucre	Filas de mariche	*P. geniculatus*	2010	Female	Positive	Yes
1791	VE59910	Miranda	Sucre	Filas de mariche	*P. geniculatus*	2010	Female	Positive	Yes
1792	VE5710	Miranda	Sucre	Filas de mariche	*P. geniculatus*	2010	Female	Positive	Yes
1793	VE100910A	Miranda	Sucre	Filas de mariche	*P. geniculatus*	2010	Female	Positive	Yes
1794	VE121910	Miranda	Sucre	Filas de mariche	*P. geniculatus*	2010	Female	Positive	Yes
1795	VE59610	Miranda	Sucre	Filas de mariche	*P. geniculatus*	2010	Female	Positive	Yes
1796	VE8610	Miranda	Sucre	Filas de mariche	*P. geniculatus*	2010	Female	NA	Yes
1797	VE135010B	Miranda	Sucre	Filas de mariche	*P. geniculatus*	2010	Female	NA	NA
1798	VE3410	Miranda	Sucre	Filas de mariche	*P. geniculatus*	2010	Female	NA	No
1799	VE112210	Miranda	Sucre	Filas de mariche	*P. geniculatus*	2010	Male	NA	No
1800	VE20710	Miranda	Sucre	Filas de mariche	*P. geniculatus*	2010	Male	Negative	Yes
1801	VE122710	Miranda	Sucre	Filas de mariche	*P. geniculatus*	2010	Male	Negative	Yes
1802	VE119010	Miranda	Sucre	Filas de mariche	*P. geniculatus*	2010	Male	Positive	No
1803	VE6810	Miranda	Sucre	Filas de mariche	*P. geniculatus*	2010	Male	Positive	Yes
1804	VE5610	Miranda	Sucre	Filas de mariche	*P. geniculatus*	2010	Male	NA	Yes

1805	VE140410	Miranda	Sucre	La Dolorita	*P. geniculatus*	2010	Nymph IV	Positive	Yes
1806	VE39310A	Miranda	Sucre	La Dolorita	*P. geniculatus*	2010	Female	NA	NA
1807	VE39310B	Miranda	Sucre	La Dolorita	*P. geniculatus*	2010	Female	NA	NA
1808	VE39310C	Miranda	Sucre	La Dolorita	*P. geniculatus*	2010	Male	NA	NA
1809	VE39310D	Miranda	Sucre	La Dolorita	*P. geniculatus*	2010	Male	NA	NA
1810	VE10410	Miranda	Sucre	La Dolorita	*P. geniculatus*	2010	Female	Negative	No
1811	VE14410	Miranda	Sucre	La Dolorita	*P. geniculatus*	2010	Female	Negative	No
1812	VE66310	Miranda	Sucre	La Dolorita	*P. geniculatus*	2010	Female	Negative	Yes
1813	VE86910A	Miranda	Sucre	La Dolorita	*P. geniculatus*	2010	Male	NA	NA
1814	VE86910B	Miranda	Sucre	La Dolorita	*P. geniculatus*	2010	Female	Positive	No
1815	VE86910C	Miranda	Sucre	La Dolorita	*P. geniculatus*	2010	Female	Positive	No
1816	VE86910D	Miranda	Sucre	La Dolorita	*P. geniculatus*	2010	Female	Positive	No
1817	VE71810	Miranda	Sucre	La Dolorita	*P. geniculatus*	2010	Female	Positive	Yes
1818	VE26410	Miranda	Sucre	La Dolorita	*P. geniculatus*	2010	Female	Positive	Yes
1819	VE99910	Miranda	Sucre	La Dolorita	*P. geniculatus*	2010	Female	Positive	Yes
1820	VE117710	Miranda	Sucre	La Dolorita	*P. geniculatus*	2010	Female	Positive	Yes
1821	VE7410	Miranda	Sucre	La Dolorita	*P. geniculatus*	2010	Female	NA	No
1822	VE97210	Miranda	Sucre	La Dolorita	*P. geniculatus*	2010	Male	Negative	No
1823	VE14510	Miranda	Sucre	La Dolorita	*P. geniculatus*	2010	Male	Negative	No
1824	VE72010	Miranda	Sucre	La Dolorita	*P. geniculatus*	2010	Male	Positive	No
1825	VE8910	Miranda	Sucre	La Dolorita	*P. geniculatus*	2010	Male	Positive	No
1826	VE45810	Miranda	Sucre	La Dolorita	*P. geniculatus*	2010	Male	Positive	No
1827	VE44710	Miranda	Sucre	La Dolorita	*P. geniculatus*	2010	Male	Positive	Yes
1828	VE10310	Miranda	Sucre	La Dolorita	*P. geniculatus*	2010	Male	NA	No
1829	VE30210	Miranda	Sucre	La Dolorita	*P. geniculatus*	2010	Male	NA	No
1830	VE141410	Miranda	Baruta	Las Minas de Baruta	*P. geniculatus*	2010	Female	Negative	No
1831	VE16510	Miranda	Baruta	Las Minas de Baruta	*P. geniculatus*	2010	Female	Negative	No
1832	VE117010A	Miranda	Baruta	Las Minas de Baruta	*P. geniculatus*	2010	Female	Positive	No
1833	VE89610	Miranda	Baruta	Las Minas de Baruta	*P. geniculatus*	2010	Female	Positive	Yes
1834	VE44910	Miranda	Baruta	Las Minas de Baruta	*P. geniculatus*	2010	Female	Positive	Yes
1835	VE120710	Miranda	Baruta	Las Minas de Baruta	*P. geniculatus*	2010	Female	NA	Yes
1836	VE26710A	Miranda	Baruta	Las Minas de Baruta	*P. geniculatus*	2010	Male	NA	No
1837	VE26710B	Miranda	Baruta	Las Minas de Baruta	*P. geniculatus*	2010	Female	NA	No
1838	VE26710C	Miranda	Baruta	Las Minas de Baruta	*P. geniculatus*	2010	Female	NA	No
1839	VE79510	Miranda	Baruta	Las Minas de Baruta	*P. geniculatus*	2010	Male	NA	No
1840	VE91110	Miranda	Baruta	Las Minas de Baruta	*P. geniculatus*	2010	Male	NA	No
1841	VE58110	Miranda	Baruta	Las Minas de Baruta	*P. geniculatus*	2010	Male	Negative	No
1842	VE93710	Miranda	Baruta	Las Minas de Baruta	*P. geniculatus*	2010	Male	Positive	No
1843	VE117010B	Miranda	Baruta	Las Minas de Baruta	*P. geniculatus*	2010	Male	Positive	No
1844	VE58910	Miranda	Baruta	Las Minas de Baruta	*P. geniculatus*	2010	Male	Positive	No
1845	VE74510	Miranda	Baruta	Las Minas de Baruta	*P. geniculatus*	2010	Male	Positive	Yes
1846	VE64410	Miranda	Baruta	Las Minas de Baruta	*P. geniculatus*	2010	Male	Positive	Yes

1847	VE11310	Miranda	Baruta	Las Minas de Baruta	*P. geniculatus*	2010	Male	Positive	Yes
1848	VE128610	Miranda	Baruta	Las Minas de Baruta	*P. geniculatus*	2010	Male	Positive	Yes
1849	VE41910	Miranda	Baruta	Las Minas de Baruta	*P. geniculatus*	2010	Male	NA	No
1850	VE17510A	Miranda	Baruta	Las Minas de Baruta	*P. geniculatus*	2010	Female	NA	No
1851	VE17510B	Miranda	Baruta	Las Minas de Baruta	*P. geniculatus*	2010	Male	Positive	No
1852	VE110610	Miranda	Baruta	Las Minas de Baruta	*P. geniculatus*	2010	Nymph IV	Positive	Yes
1853	VE32010A	Miranda	Baruta	Las Minas de Baruta	*P. geniculatus*	2010	Male	NA	No
1854	VE32010B	Miranda	Baruta	Las Minas de Baruta	*P. geniculatus*	2010	Female	NA	No
1855	VE91010	Miranda	Sucre	Leoncio Martínez	*P. geniculatus*	2010	Female	NA	No
1856	VE135910A	Miranda	Sucre	Leoncio Martínez	*P. geniculatus*	2010	Female	NA	No
1857	VE139210	Miranda	Sucre	Leoncio Martínez	*P. geniculatus*	2010	Female	NA	Yes
1858	VE81610	Miranda	Sucre	Leoncio Martínez	*P. geniculatus*	2010	Female	Negative	No
1859	VE78610	Miranda	Sucre	Leoncio Martínez	*P. geniculatus*	2010	Female	Positive	No
1860	VE25510	Miranda	Sucre	Leoncio Martínez	*P. geniculatus*	2010	Female	Positive	No
1861	VE28710	Miranda	Sucre	Leoncio Martínez	*P. geniculatus*	2010	Female	Positive	No
1862	VE140910	Miranda	Sucre	Leoncio Martínez	*P. geniculatus*	2010	Female	Positive	Yes
1863	VE131110	Miranda	Sucre	Leoncio Martínez	*P. geniculatus*	2010	Female	Positive	Yes
1864	VE136110	Miranda	Sucre	Leoncio Martínez	*P. geniculatus*	2010	Female	Positive	Yes
1865	VE25610	Miranda	Sucre	Leoncio Martínez	*P. geniculatus*	2010	Female	Positive	Yes
1866	VE120910	Miranda	Sucre	Leoncio Martínez	*P. geniculatus*	2010	Female	Positive	Yes
1867	VE41110	Miranda	Sucre	Leoncio Martínez	*P. geniculatus*	2010	Female	Positive	Yes
1868	VE1110	Miranda	Sucre	Leoncio Martínez	*P. geniculatus*	2010	Female	NA	No
1869	VE35510	Miranda	Sucre	Leoncio Martínez	*P. geniculatus*	2010	Female	NA	Yes
1870	VE136910	Miranda	Sucre	Leoncio Martínez	*P. geniculatus*	2010	Female	NA	No
1871	VE24210	Miranda	Sucre	Leoncio Martínez	*P. geniculatus*	2010	Female	NA	No
1872	VE87510	Miranda	Sucre	Leoncio Martínez	*P. geniculatus*	2010	Female	NA	No
1873	VE109810	Miranda	Sucre	Leoncio Martínez	*P. geniculatus*	2010	Female	NA	No
1874	VE135910B	Miranda	Sucre	Leoncio Martínez	*P. geniculatus*	2010	Male	NA	No
1875	VE88210	Miranda	Sucre	Leoncio Martínez	*P. geniculatus*	2010	Male	Negative	No
1876	VE3310	Miranda	Sucre	Leoncio Martínez	*P. geniculatus*	2010	Male	Negative	No
1877	VE24110	Miranda	Sucre	Leoncio Martínez	*P. geniculatus*	2010	Male	Negative	No
1878	VE25110 Consulta Externa	Miranda	Sucre	Leoncio Martínez	*P. geniculatus*	2010	Male	Negative	No
1879	VE75310	Miranda	Sucre	Leoncio Martínez	*P. geniculatus*	2010	Male	Negative	No
1880	VE45110A	Miranda	Sucre	Leoncio Martínez	*P. geniculatus*	2010	Male	Negative	Yes
1881	VE45110B	Miranda	Sucre	Leoncio Martínez	*P. geniculatus*	2010	Male	Negative	Yes
1882	VE130310	Miranda	Sucre	Leoncio Martínez	*P. geniculatus*	2010	Male	Positive	No
1883	VE131310	Miranda	Sucre	Leoncio Martínez	*P. geniculatus*	2010	Male	Positive	No
1884	VE132810	Miranda	Sucre	Leoncio Martínez	*P. geniculatus*	2010	Male	Positive	No
1885	VE68810	Miranda	Sucre	Leoncio Martínez	*P. geniculatus*	2010	Male	Positive	No
1886	VE114610	Miranda	Sucre	Leoncio Martínez	*P. geniculatus*	2010	Male	Positive	No
1887	VE123610	Miranda	Sucre	Leoncio Martínez	*P. geniculatus*	2010	Male	Positive	No
1888	VE88110	Miranda	Sucre	Leoncio Martínez	*P. geniculatus*	2010	Male	Positive	No

1889	VE2210	Miranda	Sucre	Leoncio Martínez	*P. geniculatus*	2010	Male	Positive	No
1890	VE111710A	Miranda	Sucre	Leoncio Martínez	*P. geniculatus*	2010	Male	Positive	No
1891	VE122210	Miranda	Sucre	Leoncio Martínez	*P. geniculatus*	2010	Male	Positive	No
1892	VE93410	Miranda	Sucre	Leoncio Martínez	*P. geniculatus*	2010	Male	Positive	No
1893	VE142710	Miranda	Sucre	Leoncio Martínez	*P. geniculatus*	2010	Male	Positive	No
1894	VE126410	Miranda	Sucre	Leoncio Martínez	*P. geniculatus*	2010	Male	Positive	No
1895	VE74410	Miranda	Sucre	Leoncio Martínez	*P. geniculatus*	2010	Male	Positive	No
1896	VE103210	Miranda	Sucre	Leoncio Martínez	*P. geniculatus*	2010	Male	Positive	No
1897	VE22710A	Miranda	Sucre	Leoncio Martínez	*P. geniculatus*	2010	Male	Positive	No
1898	VE19210	Miranda	Sucre	Leoncio Martínez	*P. geniculatus*	2010	Male	Positive	No
1899	VE77310	Miranda	Sucre	Leoncio Martínez	*P. geniculatus*	2010	Male	Positive	Yes
1900	VE93010	Miranda	Sucre	Leoncio Martínez	*P. geniculatus*	2010	Male	Positive	Yes
1901	VE27510	Miranda	Sucre	Leoncio Martínez	*P. geniculatus*	2010	Male	Positive	Yes
1902	VE122310	Miranda	Sucre	Leoncio Martínez	*P. geniculatus*	2010	Male	Positive	Yes
1903	VE131510	Miranda	Sucre	Leoncio Martínez	*P. geniculatus*	2010	Male	NA	No
1904	VE49210	Miranda	Sucre	Leoncio Martínez	*P. geniculatus*	2010	Male	NA	NA
1905	VE62710	Miranda	Baruta	Nuestra Señora del Rosario de Baruta	*P. geniculatus*	2010	Female	NA	Yes
1906	VE143510	Miranda	Baruta	Nuestra Señora del Rosario de Baruta	*P. geniculatus*	2010	Female	Negative	No
1907	VE93310	Miranda	Baruta	Nuestra Señora del Rosario de Baruta	*P. geniculatus*	2010	Female	Negative	No
1908	VE131710	Miranda	Baruta	Nuestra Señora del Rosario de Baruta	*P. geniculatus*	2010	Female	Negative	Yes
1909	VE92110	Miranda	Baruta	Nuestra Señora del Rosario de Baruta	*P. geniculatus*	2010	Female	Negative	Yes
1910	VE59510	Miranda	Baruta	Nuestra Señora del Rosario de Baruta	*P. geniculatus*	2010	Female	Negative	Yes
1911	VE50910	Miranda	Baruta	Nuestra Señora del Rosario de Baruta	*P. geniculatus*	2010	Female	Negative	Yes
1912	VE51710	Miranda	Baruta	Nuestra Señora del Rosario de Baruta	*P. geniculatus*	2010	Female	Negative	Yes
1913	VE56110	Miranda	Baruta	Nuestra Señora del Rosario de Baruta	*P. geniculatus*	2010	Female	Negative	Yes
1914	VE127810	Miranda	Baruta	Nuestra Señora del Rosario de Baruta	*P. geniculatus*	2010	Female	Negative	Yes
1915	VE130810	Miranda	Baruta	Nuestra Señora del Rosario de Baruta	*P. geniculatus*	2010	Female	Positive	No
1916	VE136510	Miranda	Baruta	Nuestra Señora del Rosario de Baruta	*P. geniculatus*	2010	Female	Positive	No
1917	VE54510A	Miranda	Baruta	Nuestra Señora del Rosario de Baruta	*P. geniculatus*	2010	Female	Positive	No
1918	VE54510B	Miranda	Baruta	Nuestra Señora del Rosario de Baruta	*P. geniculatus*	2010	Female	Positive	No
1919	VE54510C	Miranda	Baruta	Nuestra Señora del Rosario de Baruta	*P. geniculatus*	2010	Female	NA	NA
1920	VE54510D	Miranda	Baruta	Nuestra Señora del Rosario de Baruta	*P. geniculatus*	2010	Female	NA	NA
1921	VE124110	Miranda	Baruta	Nuestra Señora del Rosario de Baruta	*P. geniculatus*	2010	Female	Positive	No
1922	VE78710	Miranda	Baruta	Nuestra Señora del Rosario de Baruta	*P. geniculatus*	2010	Female	Positive	No
1923	VE110510	Miranda	Baruta	Nuestra Señora del Rosario de Baruta	*P. geniculatus*	2010	Female	Positive	No
1924	VE98610A	Miranda	Baruta	Nuestra Señora del Rosario de Baruta	*P. geniculatus*	2010	Male	NA	No
1925	VE98610B	Miranda	Baruta	Nuestra Señora del Rosario de Baruta	*P. geniculatus*	2010	Male	NA	No
1926	VE98610C	Miranda	Baruta	Nuestra Señora del Rosario de Baruta	*P. geniculatus*	2010	Male	NA	No
1927	VE98610D	Miranda	Baruta	Nuestra Señora del Rosario de Baruta	*P. geniculatus*	2010	Male	NA	No
1928	VE98610E	Miranda	Baruta	Nuestra Señora del Rosario de Baruta	*P. geniculatus*	2010	Female	NA	No
1929	VE98610F	Miranda	Baruta	Nuestra Señora del Rosario de Baruta	*P. geniculatus*	2010	Female	Positive	No
1930	VE51010	Miranda	Baruta	Nuestra Señora del Rosario de Baruta	*P. geniculatus*	2010	Female	Positive	No

1931	VE111410	Miranda	Baruta	Nuestra Señora del Rosario de Baruta	*P. geniculatus*	2010	Female	Positive	No
1932	VE7310	Miranda	Baruta	Nuestra Señora del Rosario de Baruta	*P. geniculatus*	2010	Female	Positive	No
1933	VE22010A	Miranda	Baruta	Nuestra Señora del Rosario de Baruta	*P. geniculatus*	2010	Female	Positive	No
1934	VE22010B	Miranda	Baruta	Nuestra Señora del Rosario de Baruta	*P. geniculatus*	2010	Female	Positive	No
1935	VE71710	Miranda	Baruta	Nuestra Señora del Rosario de Baruta	*P. geniculatus*	2010	Female	Positive	No
1936	VE43810A	Miranda	Baruta	Nuestra Señora del Rosario de Baruta	*P. geniculatus*	2010	Female	NA	No
1937	VE43810B	Miranda	Baruta	Nuestra Señora del Rosario de Baruta	*P. geniculatus*	2010	Male	NA	No
1938	VE43810C	Miranda	Baruta	Nuestra Señora del Rosario de Baruta	*P. geniculatus*	2010	Female	Positive	No
1939	VE75810	Miranda	Baruta	Nuestra Señora del Rosario de Baruta	*P. geniculatus*	2010	Female	Positive	No
1940	VE77910	Miranda	Baruta	Nuestra Señora del Rosario de Baruta	*P. geniculatus*	2010	Female	Positive	No
1941	VE9210	Miranda	Baruta	Nuestra Señora del Rosario de Baruta	*P. geniculatus*	2010	Female	Positive	No
1942	VE52110	Miranda	Baruta	Nuestra Señora del Rosario de Baruta	*P. geniculatus*	2010	Female	Positive	No
1943	VE0910	Miranda	Baruta	Nuestra Señora del Rosario de Baruta	*P. geniculatus*	2010	Female	Positive	No
1944	VE58410	Miranda	Baruta	Nuestra Señora del Rosario de Baruta	*P. geniculatus*	2010	Female	Positive	No
1945	VE77210	Miranda	Baruta	Nuestra Señora del Rosario de Baruta	*P. geniculatus*	2010	Female	Positive	No
1946	VE75210	Miranda	Baruta	Nuestra Señora del Rosario de Baruta	*P. geniculatus*	2010	Female	Positive	No
1947	VE109510	Miranda	Baruta	Nuestra Señora del Rosario de Baruta	*P. geniculatus*	2010	Female	Positive	No
1948	VE3810	Miranda	Baruta	Nuestra Señora del Rosario de Baruta	*P. geniculatus*	2010	Female	Positive	Yes
1949	VE63710	Miranda	Baruta	Nuestra Señora del Rosario de Baruta	*P. geniculatus*	2010	Female	Positive	Yes
1950	VE36710	Miranda	Baruta	Nuestra Señora del Rosario de Baruta	*P. geniculatus*	2010	Female	Positive	Yes
1951	VE16710	Miranda	Baruta	Nuestra Señora del Rosario de Baruta	*P. geniculatus*	2010	Female	Positive	Yes
1952	VE98310	Miranda	Baruta	Nuestra Señora del Rosario de Baruta	*P. geniculatus*	2010	Female	Positive	Yes
1953	VE62510	Miranda	Baruta	Nuestra Señora del Rosario de Baruta	*P. geniculatus*	2010	Female	Positive	Yes
1954	VE2410A	Miranda	Baruta	Nuestra Señora del Rosario de Baruta	*P. geniculatus*	2010	Female	Positive	No
1955	VE2410B	Miranda	Baruta	Nuestra Señora del Rosario de Baruta	*P. geniculatus*	2010	Female	Positive	No
1956	VE2410C	Miranda	Baruta	Nuestra Señora del Rosario de Baruta	*P. geniculatus*	2010	Female	Positive	Yes
1957	VE2410D	Miranda	Baruta	Nuestra Señora del Rosario de Baruta	*P. geniculatus*	2010	Female	Positive	Yes
1958	VE65710	Miranda	Baruta	Nuestra Señora del Rosario de Baruta	*P. geniculatus*	2010	Female	Positive	Yes
1959	VE27010	Miranda	Baruta	Nuestra Señora del Rosario de Baruta	*P. geniculatus*	2010	Female	Positive	Yes
1960	VE80610	Miranda	Baruta	Nuestra Señora del Rosario de Baruta	*P. geniculatus*	2010	Female	Positive	Yes
1961	VE64310	Miranda	Baruta	Nuestra Señora del Rosario de Baruta	*P. geniculatus*	2010	Female	Positive	Yes
1962	VE26510	Miranda	Baruta	Nuestra Señora del Rosario de Baruta	*P. geniculatus*	2010	Female	Positive	Yes
1963	VE127110	Miranda	Baruta	Nuestra Señora del Rosario de Baruta	*P. geniculatus*	2010	Female	Positive	Yes
1964	VE1210	Miranda	Baruta	Nuestra Señora del Rosario de Baruta	*P. geniculatus*	2010	Female	NA	No
1965	VE10910	Miranda	Baruta	Nuestra Señora del Rosario de Baruta	*P. geniculatus*	2010	Female	NA	Yes
1966	VE117210	Miranda	Baruta	Nuestra Señora del Rosario de Baruta	*P. geniculatus*	2010	Female	NA	NA
1967	VE130910	Miranda	Baruta	Nuestra Señora del Rosario de Baruta	*P. geniculatus*	2010	Female	NA	No
1968	VE10810	Miranda	Baruta	Nuestra Señora del Rosario de Baruta	*P. geniculatus*	2010	Female	NA	No
1969	VE11110A	Miranda	Baruta	Nuestra Señora del Rosario de Baruta	*P. geniculatus*	2010	Male	NA	No
1970	VE11110B	Miranda	Baruta	Nuestra Señora del Rosario de Baruta	*P. geniculatus*	2010	Male	NA	No
1971	VE11110C	Miranda	Baruta	Nuestra Señora del Rosario de Baruta	*P. geniculatus*	2010	Female	NA	No
1972	VE11110D	Miranda	Baruta	Nuestra Señora del Rosario de Baruta	*P. geniculatus*	2010	Female	NA	No

1973	VE11110E	Miranda	Baruta	Nuestra Señora del Rosario de Baruta	*P. geniculatus*	2010	Female	NA	No
1974	VE11110F	Miranda	Baruta	Nuestra Señora del Rosario de Baruta	*P. geniculatus*	2010	Female	NA	No
1975	VE35610	Miranda	Baruta	Nuestra Señora del Rosario de Baruta	*P. geniculatus*	2010	Female	NA	No
1976	VE24310	Miranda	Baruta	Nuestra Señora del Rosario de Baruta	*P. geniculatus*	2010	Female	NA	No
1977	VE101710	Miranda	Baruta	Nuestra Señora del Rosario de Baruta	*P. geniculatus*	2010	Female	NA	No
1978	VE87110	Miranda	Baruta	Nuestra Señora del Rosario de Baruta	*P. geniculatus*	2010	Female	NA	No
1979	VE51210	Miranda	Baruta	Nuestra Señora del Rosario de Baruta	*P. geniculatus*	2010	Female	NA	NA
1980	VE80110	Miranda	Baruta	Nuestra Señora del Rosario de Baruta	*P. geniculatus*	2010	Male	NA	No
1981	VE110810	Miranda	Baruta	Nuestra Señora del Rosario de Baruta	*P. geniculatus*	2010	Male	NA	No
1982	VE132010	Miranda	Baruta	Nuestra Señora del Rosario de Baruta	*P. geniculatus*	2010	Male	NA	No
1983	VE138910	Miranda	Baruta	Nuestra Señora del Rosario de Baruta	*P. geniculatus*	2010	Male	Negative	No
1984	VE11010	Miranda	Baruta	Nuestra Señora del Rosario de Baruta	*P. geniculatus*	2010	Male	Negative	No
1985	VE128310	Miranda	Baruta	Nuestra Señora del Rosario de Baruta	*P. geniculatus*	2010	Male	Negative	No
1986	VE6110	Miranda	Baruta	Nuestra Señora del Rosario de Baruta	*P. geniculatus*	2010	Male	Negative	No
1987	VE12310	Miranda	Baruta	Nuestra Señora del Rosario de Baruta	*P. geniculatus*	2010	Male	Negative	No
1988	VE121710	Miranda	Baruta	Nuestra Señora del Rosario de Baruta	*P. geniculatus*	2010	Male	Negative	No
1989	VE57910	Miranda	Baruta	Nuestra Señora del Rosario de Baruta	*P. geniculatus*	2010	Male	Negative	No
1990	VE117310	Miranda	Baruta	Nuestra Señora del Rosario de Baruta	*P. geniculatus*	2010	Male	Negative	No
1991	VE95710	Miranda	Baruta	Nuestra Señora del Rosario de Baruta	*P. geniculatus*	2010	Male	Negative	No
1992	VE57810	Miranda	Baruta	Nuestra Señora del Rosario de Baruta	*P. geniculatus*	2010	Male	Negative	No
1993	VE7910	Miranda	Baruta	Nuestra Señora del Rosario de Baruta	*P. geniculatus*	2010	Male	Negative	No
1994	VE85610	Miranda	Baruta	Nuestra Señora del Rosario de Baruta	*P. geniculatus*	2010	Male	Negative	No
1995	VE72610	Miranda	Baruta	Nuestra Señora del Rosario de Baruta	*P. geniculatus*	2010	Male	Negative	No
1996	VE9710	Miranda	Baruta	Nuestra Señora del Rosario de Baruta	*P. geniculatus*	2010	Male	Negative	Yes
1997	VE101010	Miranda	Baruta	Nuestra Señora del Rosario de Baruta	*P. geniculatus*	2010	Male	Negative	Yes
1998	VE75110	Miranda	Baruta	Nuestra Señora del Rosario de Baruta	*P. geniculatus*	2010	Male	Negative	Yes
1999	VE31510	Miranda	Baruta	Nuestra Señora del Rosario de Baruta	*P. geniculatus*	2010	Male	Negative	Yes
2000	VE138310	Miranda	Baruta	Nuestra Señora del Rosario de Baruta	*P. geniculatus*	2010	Male	Positive	No
2001	VE77410	Miranda	Baruta	Nuestra Señora del Rosario de Baruta	*P. geniculatus*	2010	Male	Positive	No
2002	VE119310	Miranda	Baruta	Nuestra Señora del Rosario de Baruta	*P. geniculatus*	2010	Male	Positive	No
2003	VE86610	Miranda	Baruta	Nuestra Señora del Rosario de Baruta	*P. geniculatus*	2010	Male	Positive	No
2004	VE57610	Miranda	Baruta	Nuestra Señora del Rosario de Baruta	*P. geniculatus*	2010	Male	Positive	No
2005	VE90010	Miranda	Baruta	Nuestra Señora del Rosario de Baruta	*P. geniculatus*	2010	Male	Positive	No
2006	VE66910	Miranda	Baruta	Nuestra Señora del Rosario de Baruta	*P. geniculatus*	2010	Male	Positive	No
2007	VE72810	Miranda	Baruta	Nuestra Señora del Rosario de Baruta	*P. geniculatus*	2010	Male	Positive	No
2008	VE81710	Miranda	Baruta	Nuestra Señora del Rosario de Baruta	*P. geniculatus*	2010	Male	Positive	No
2009	VE73110	Miranda	Baruta	Nuestra Señora del Rosario de Baruta	*P. geniculatus*	2010	Male	Positive	No
2010	VE106110	Miranda	Baruta	Nuestra Señora del Rosario de Baruta	*P. geniculatus*	2010	Male	Positive	No
2011	VE81210	Miranda	Baruta	Nuestra Señora del Rosario de Baruta	*P. geniculatus*	2010	Male	Positive	No
2012	VE35010	Miranda	Baruta	Nuestra Señora del Rosario de Baruta	*P. geniculatus*	2010	Male	Positive	No
2013	VE82210	Miranda	Baruta	Nuestra Señora del Rosario de Baruta	*P. geniculatus*	2010	Male	Positive	Yes
2014	VE121610	Miranda	Baruta	Nuestra Señora del Rosario de Baruta	*P. geniculatus*	2010	Male	Positive	Yes

2015	VE50110	Miranda	Baruta	Nuestra Señora del Rosario de Baruta	*P. geniculatus*	2010	Male	Positive	Yes
2016	VE35410	Miranda	Baruta	Nuestra Señora del Rosario de Baruta	*P. geniculatus*	2010	Male	Positive	Yes
2017	VE124910	Miranda	Baruta	Nuestra Señora del Rosario de Baruta	*P. geniculatus*	2010	Male	Positive	Yes
2018	VE53210	Miranda	Baruta	Nuestra Señora del Rosario de Baruta	*P. geniculatus*	2010	Male	NA	Yes
2019	VE126810	Miranda	Baruta	Nuestra Señora del Rosario de Baruta	*P. geniculatus*	2010	Male	NA	NA
2020	VE130410	Miranda	Baruta	Nuestra Señora del Rosario de Baruta	*P. geniculatus*	2010	Male	NA	NA
2021	VE25110 Lab 224	Miranda	Baruta	Nuestra Señora del Rosario de Baruta	*P. geniculatus*	2010	Male	NA	No
2022	VE112610	Miranda	Baruta	Nuestra Señora del Rosario de Baruta	*P. geniculatus*	2010	Male	NA	No
2023	VE95510	Miranda	Baruta	Nuestra Señora del Rosario de Baruta	*P. geniculatus*	2010	Female	Negative	Yes
2024	VE40110	Miranda	Sucre	Petare	*P. geniculatus*	2010	Male	Positive	No
2025	VE113910	Miranda	Sucre	Petare	*P. geniculatus*	2010	Female	NA	No
2026	VE108210	Miranda	Sucre	Petare	*P. geniculatus*	2010	Female	NA	Yes
2027	VE100710	Miranda	Sucre	Petare	*P. geniculatus*	2010	Female	NA	Yes
2028	VE88310	Miranda	Sucre	Petare	*P. geniculatus*	2010	Female	NA	Yes
2029	VE137010	Miranda	Sucre	Petare	*P. geniculatus*	2010	Female	NA	Yes
2030	VE84510	Miranda	Sucre	Petare	*P. geniculatus*	2010	Female	Negative	No
2031	VE48310	Miranda	Sucre	Petare	*P. geniculatus*	2010	Female	Negative	No
2032	VE16610	Miranda	Sucre	Petare	*P. geniculatus*	2010	Female	Negative	No
2033	VE105910	Miranda	Sucre	Petare	*P. geniculatus*	2010	Female	Negative	No
2034	VE143310	Miranda	Sucre	Petare	*P. geniculatus*	2010	Female	Negative	Yes
2035	VE16110	Miranda	Sucre	Petare	*P. geniculatus*	2010	Female	Negative	Yes
2036	VE53610	Miranda	Sucre	Petare	*P. geniculatus*	2010	Female	Negative	Yes
2037	VE14910	Miranda	Sucre	Petare	*P. geniculatus*	2010	Female	Negative	Yes
2038	VE120510	Miranda	Sucre	Petare	*P. geniculatus*	2010	Female	Negative	Yes
2039	VE68010A	Miranda	Sucre	Petare	*P. geniculatus*	2010	Female	Positive	Yes
2040	VE68010B	Miranda	Sucre	Petare	*P. geniculatus*	2010	Female	Positive	Yes
2041	VE56010A	Miranda	Sucre	Petare	*P. geniculatus*	2010	Female	NA	NA
2042	VE56010B	Miranda	Sucre	Petare	*P. geniculatus*	2010	Female	Negative	Yes
2043	VE31110	Miranda	Sucre	Petare	*P. geniculatus*	2010	Female	Negative	Yes
2044	VE76510	Miranda	Sucre	Petare	*P. geniculatus*	2010	Female	Positive	No
2045	VE66010A	Miranda	Sucre	Petare	*P. geniculatus*	2010	Female	NA	NA
2046	VE66010B	Miranda	Sucre	Petare	*P. geniculatus*	2010	Female	Positive	No
2047	VE37210	Miranda	Sucre	Petare	*P. geniculatus*	2010	Female	Positive	No
2048	VE129510	Miranda	Sucre	Petare	*P. geniculatus*	2010	Female	Positive	No
2049	VE62010A	Miranda	Sucre	Petare	*P. geniculatus*	2010	Male	Positive	No
2050	VE62010B	Miranda	Sucre	Petare	*P. geniculatus*	2010	Female	Positive	No
2051	VE83410A	Miranda	Sucre	Petare	*P. geniculatus*	2010	Male	Positive	No
2052	VE83410B	Miranda	Sucre	Petare	*P. geniculatus*	2010	Female	Positive	No
2053	VE78310	Miranda	Sucre	Petare	*P. geniculatus*	2010	Female	Positive	No
2054	VE4610	Miranda	Sucre	Petare	*P. geniculatus*	2010	Female	Positive	No
2055	VE75710	Miranda	Sucre	Petare	*P. geniculatus*	2010	Female	Positive	No
2056	VE129310A	Miranda	Sucre	Petare	*P. geniculatus*	2010	Female	Positive	No

2057	VE26910	Miranda	Sucre	Petare	*P. geniculatus*	2010	Female	Positive	No
2058	VE8310A	Miranda	Sucre	Petare	*P. geniculatus*	2010	Female	Positive	No
2059	VE8310B	Miranda	Sucre	Petare	*P. geniculatus*	2010	Female	NA	NA
2060	VE99810	Miranda	Sucre	Petare	*P. geniculatus*	2010	Female	Positive	No
2061	VE5310	Miranda	Sucre	Petare	*P. geniculatus*	2010	Female	Positive	No
2062	VE13910	Miranda	Sucre	Petare	*P. geniculatus*	2010	Female	Positive	No
2063	VE105710	Miranda	Sucre	Petare	*P. geniculatus*	2010	Female	Positive	No
2064	VE106510	Miranda	Sucre	Petare	*P. geniculatus*	2010	Female	Positive	No
2065	VE20110	Miranda	Sucre	Petare	*P. geniculatus*	2010	Female	Positive	No
2066	VE80910A	Miranda	Sucre	Petare	*P. geniculatus*	2010	Male	Positive	No
2067	VE80910B	Miranda	Sucre	Petare	*P. geniculatus*	2010	Female	Positive	No
2068	VE45910	Miranda	Sucre	Petare	*P. geniculatus*	2010	Female	Positive	No
2069	VE27310	Miranda	Sucre	Petare	*P. geniculatus*	2010	Female	Positive	No
2070	VE117810	Miranda	Sucre	Petare	*P. geniculatus*	2010	Female	Positive	Yes
2071	VE137410	Miranda	Sucre	Petare	*P. geniculatus*	2010	Female	Positive	Yes
2072	VE139810	Miranda	Sucre	Petare	*P. geniculatus*	2010	Female	Positive	Yes
2073	VE144810	Miranda	Sucre	Petare	*P. geniculatus*	2010	Female	Positive	Yes
2074	VE133610	Miranda	Sucre	Petare	*P. geniculatus*	2010	Female	Positive	Yes
2075	VE143010A	Miranda	Sucre	Petare	*P. geniculatus*	2010	Female	Positive	Yes
2076	VE143010B	Miranda	Sucre	Petare	*P. geniculatus*	2010	Female	Positive	Yes
2077	VE143010C	Miranda	Sucre	Petare	*P. geniculatus*	2010	Female	Positive	Yes
2078	VE137210	Miranda	Sucre	Petare	*P. geniculatus*	2010	Female	Positive	Yes
2079	VE133510	Miranda	Sucre	Petare	*P. geniculatus*	2010	Female	Positive	Yes
2080	VE138210A	Miranda	Sucre	Petare	*P. geniculatus*	2010	Female	Positive	Yes
2081	VE72310A	Miranda	Sucre	Petare	*P. geniculatus*	2010	Male	Positive	Yes
2082	VE72310B	Miranda	Sucre	Petare	*P. geniculatus*	2010	Female	Positive	Yes
2083	VE109010	Miranda	Sucre	Petare	*P. geniculatus*	2010	Female	Positive	Yes
2084	VE109410A	Miranda	Sucre	Petare	*P. geniculatus*	2010	Female	NA	NA
2085	VE109410B	Miranda	Sucre	Petare	*P. geniculatus*	2010	Female	Positive	Yes
2086	VE103310	Miranda	Sucre	Petare	*P. geniculatus*	2010	Female	Positive	Yes
2087	VE56610	Miranda	Sucre	Petare	*P. geniculatus*	2010	Female	Positive	Yes
2088	VE20510	Miranda	Sucre	Petare	*P. geniculatus*	2010	Female	Positive	Yes
2089	VE13710	Miranda	Sucre	Petare	*P. geniculatus*	2010	Female	Positive	Yes
2090	VE101210	Miranda	Sucre	Petare	*P. geniculatus*	2010	Female	Positive	Yes
2091	VE71910A	Miranda	Sucre	Petare	*P. geniculatus*	2010	Nymph IV	NA	NA
2092	VE71910B	Miranda	Sucre	Petare	*P. geniculatus*	2010	Nymph IV	NA	NA
2093	VE71910C	Miranda	Sucre	Petare	*P. geniculatus*	2010	Female	Positive	Yes
2094	VE118610	Miranda	Sucre	Petare	*P. geniculatus*	2010	Female	Positive	Yes
2095	VE50410	Miranda	Sucre	Petare	*P. geniculatus*	2010	Female	Positive	Yes
2096	VE58310	Miranda	Sucre	Petare	*P. geniculatus*	2010	Female	Positive	Yes
2097	VE96410	Miranda	Sucre	Petare	*P. geniculatus*	2010	Female	Positive	Yes
2098	VE106610	Miranda	Sucre	Petare	*P. geniculatus*	2010	Female	Positive	Yes

2099	VE116310	Miranda	Sucre	Petare	*P. geniculatus*	2010	Female	Positive	Yes
2100	VE112410	Miranda	Sucre	Petare	*P. geniculatus*	2010	Female	Positive	Yes
2101	VE126510	Miranda	Sucre	Petare	*P. geniculatus*	2010	Female	Positive	Yes
2102	VE36810A	Miranda	Sucre	Petare	*P. geniculatus*	2010	Female	NA	NA
2103	VE36810B	Miranda	Sucre	Petare	*P. geniculatus*	2010	Female	NA	NA
2104	VE36810C	Miranda	Sucre	Petare	*P. geniculatus*	2010	Female	Positive	Yes
2105	VE29210	Miranda	Sucre	Petare	*P. geniculatus*	2010	Female	Positive	Yes
2106	VE55110	Miranda	Sucre	Petare	*P. geniculatus*	2010	Female	Positive	Yes
2107	VE24810	Miranda	Sucre	Petare	*P. geniculatus*	2010	Female	Positive	Yes
2108	VE52410	Miranda	Sucre	Petare	*P. geniculatus*	2010	Female	Positive	Yes
2109	VE123410	Miranda	Sucre	Petare	*P. geniculatus*	2010	Female	Positive	Yes
2110	VE88710	Miranda	Sucre	Petare	*P. geniculatus*	2010	Female	Positive	Yes
2111	VE74110	Miranda	Sucre	Petare	*P. geniculatus*	2010	Female	Positive	Yes
2112	VE120010	Miranda	Sucre	Petare	*P. geniculatus*	2010	Female	Positive	Yes
2113	VE3510	Miranda	Sucre	Petare	*P. geniculatus*	2010	Female	Positive	Yes
2114	VE75510	Miranda	Sucre	Petare	*P. geniculatus*	2010	Female	Positive	Yes
2115	VE13010	Miranda	Sucre	Petare	*P. geniculatus*	2010	Female	Positive	Yes
2116	VE42810A	Miranda	Sucre	Petare	*P. geniculatus*	2010	Female	Negative	Yes
2117	VE42810B	Miranda	Sucre	Petare	*P. geniculatus*	2010	Female	Positive	Yes
2118	VE104810	Miranda	Sucre	Petare	*P. geniculatus*	2010	Female	Positive	Yes
2119	VE47010	Miranda	Sucre	Petare	*P. geniculatus*	2010	Female	Positive	Yes
2120	VE17610	Miranda	Sucre	Petare	*P. geniculatus*	2010	Female	Positive	Yes
2121	VE95810	Miranda	Sucre	Petare	*P. geniculatus*	2010	Female	Positive	Yes
2122	VE25010CE	Miranda	Sucre	Petare	*P. geniculatus*	2010	Female	Positive	Yes
2123	VE21510	Miranda	Sucre	Petare	*P. geniculatus*	2010	Female	Positive	Yes
2124	VE6510	Miranda	Sucre	Petare	*P. geniculatus*	2010	Female	Positive	Yes
2125	VE4410A	Miranda	Sucre	Petare	*P. geniculatus*	2010	Female	Positive	Yes
2126	VE111310	Miranda	Sucre	Petare	*P. geniculatus*	2010	Female	Positive	Yes
2127	VE109110	Miranda	Sucre	Petare	*P. geniculatus*	2010	Female	Positive	Yes
2128	VE31610	Miranda	Sucre	Petare	*P. geniculatus*	2010	Female	Positive	Yes
2129	VE114310	Miranda	Sucre	Petare	*P. geniculatus*	2010	Female	Positive	Yes
2130	VE27710	Miranda	Sucre	Petare	*P. geniculatus*	2010	Female	NA	Yes
2131	VE7010	Miranda	Sucre	Petare	*P. geniculatus*	2010	Female	NA	Yes
2132	VE0510	Miranda	Sucre	Petare	*P. geniculatus*	2010	Female	NA	Yes
2133	VE50610	Miranda	Sucre	Petare	*P. geniculatus*	2010	Female	NA	Yes
2134	VE136210A	Miranda	Sucre	Petare	*P. geniculatus*	2010	Female	NA	NA
2135	VE128510C	Miranda	Sucre	Petare	*P. geniculatus*	2010	Female	NA	NA
2136	VE128510D	Miranda	Sucre	Petare	*P. geniculatus*	2010	Female	NA	NA
2137	VE128510E	Miranda	Sucre	Petare	*P. geniculatus*	2010	Female	NA	NA
2138	VE118410	Miranda	Sucre	Petare	*P. geniculatus*	2010	Female	NA	NA
2139	VE129310B	Miranda	Sucre	Petare	*P. geniculatus*	2010	Female	NA	No
2140	VE27810	Miranda	Sucre	Petare	*P. geniculatus*	2010	Female	NA	No

2141	VE7710	Miranda	Sucre	Petare	*P. geniculatus*	2010	Female	NA	No
2142	VE17110	Miranda	Sucre	Petare	*P. geniculatus*	2010	Female	NA	No
2143	VE17310	Miranda	Sucre	Petare	*P. geniculatus*	2010	Female	NA	No
2144	VE4310B	Miranda	Sucre	Petare	*P. geniculatus*	2010	Female	NA	No
2145	VE9810A	Miranda	Sucre	Petare	*P. geniculatus*	2010	Female	NA	No
2146	VE9810B	Miranda	Sucre	Petare	*P. geniculatus*	2010	Female	NA	No
2147	VE9410	Miranda	Sucre	Petare	*P. geniculatus*	2010	Female	NA	No
2148	VE9310	Miranda	Sucre	Petare	*P. geniculatus*	2010	Female	NA	Yes
2149	VE5810	Miranda	Sucre	Petare	*P. geniculatus*	2010	Female	NA	Yes
2150	VE26610	Miranda	Sucre	Petare	*P. geniculatus*	2010	Female	NA	Yes
2151	VE98210	Miranda	Sucre	Petare	*P. geniculatus*	2010	Female	NA	No
2152	VE65510A	Miranda	Sucre	Petare	*P. geniculatus*	2010	Male	NA	NA
2153	VE65510B	Miranda	Sucre	Petare	*P. geniculatus*	2010	Male	NA	NA
2154	VE65510C	Miranda	Sucre	Petare	*P. geniculatus*	2010	Female	NA	NA
2155	VE43310A	Miranda	Sucre	Petare	*P. geniculatus*	2010	Female	NA	NA
2156	VE43310B	Miranda	Sucre	Petare	*P. geniculatus*	2010	Female	NA	NA
2157	VE102210	Miranda	Sucre	Petare	*P. geniculatus*	2010	Female	NA	NA
2158	VE110910	Miranda	Sucre	Petare	*P. geniculatus*	2010	Male	NA	No
2159	VE77610	Miranda	Sucre	Petare	*P. geniculatus*	2010	Male	NA	No
2160	VE79910	Miranda	Sucre	Petare	*P. geniculatus*	2010	Male	NA	No
2161	VE47310	Miranda	Sucre	Petare	*P. geniculatus*	2010	Male	NA	NA
2162	VE94710A	Miranda	Sucre	Petare	*P. geniculatus*	2010	Female	NA	NA
2163	VE94710B	Miranda	Sucre	Petare	*P. geniculatus*	2010	Female	NA	NA
2164	VE94710C	Miranda	Sucre	Petare	*P. geniculatus*	2010	Female	NA	NA
2165	VE94710D	Miranda	Sucre	Petare	*P. geniculatus*	2010	Female	NA	NA
2166	VE94710E	Miranda	Sucre	Petare	*P. geniculatus*	2010	Male	NA	NA
2167	VE94710F	Miranda	Sucre	Petare	*P. geniculatus*	2010	Male	NA	NA
2168	VE94710G	Miranda	Sucre	Petare	*P. geniculatus*	2010	Male	NA	NA
2169	VE140110	Miranda	Sucre	Petare	*P. geniculatus*	2010	Male	NA	NA
2170	VE134610	Miranda	Sucre	Petare	*P. geniculatus*	2010	Male	Negative	No
2171	VE31910	Miranda	Sucre	Petare	*P. geniculatus*	2010	Male	Negative	No
2172	VE33610	Miranda	Sucre	Petare	*P. geniculatus*	2010	Male	Negative	No
2173	VE98910	Miranda	Sucre	Petare	*P. geniculatus*	2010	Male	Negative	No
2174	VE82110	Miranda	Sucre	Petare	*P. geniculatus*	2010	Male	Negative	No
2175	VE105610	Miranda	Sucre	Petare	*P. geniculatus*	2010	Male	Negative	No
2176	VE122110	Miranda	Sucre	Petare	*P. geniculatus*	2010	Male	Negative	No
2177	VE82610	Miranda	Sucre	Petare	*P. geniculatus*	2010	Male	Negative	No
2178	VE9010	Miranda	Sucre	Petare	*P. geniculatus*	2010	Male	Negative	No
2179	VE22210	Miranda	Sucre	Petare	*P. geniculatus*	2010	Male	Negative	No
2180	VE68910A	Miranda	Sucre	Petare	*P. geniculatus*	2010	Male	NA	NA
2181	VE68910B	Miranda	Sucre	Petare	*P. geniculatus*	2010	Male	Negative	No
2182	VE34710	Miranda	Sucre	Petare	*P. geniculatus*	2010	Male	Negative	No

2183	VE51920	Miranda	Sucre	Petare	*P. geniculatus*	2010	Male	Negative	No
2184	VE118110	Miranda	Sucre	Petare	*P. geniculatus*	2010	Male	Negative	No
2185	VE89210	Miranda	Sucre	Petare	*P. geniculatus*	2010	Male	Negative	No
2186	VE39510	Miranda	Sucre	Petare	*P. geniculatus*	2010	Male	Negative	No
2187	VE29810	Miranda	Sucre	Petare	*P. geniculatus*	2010	Male	Negative	No
2188	VE138810	Miranda	Sucre	Petare	*P. geniculatus*	2010	Male	Negative	Yes
2189	VE100610	Miranda	Sucre	Petare	*P. geniculatus*	2010	Male	Negative	Yes
2190	VE122810	Miranda	Sucre	Petare	*P. geniculatus*	2010	Male	Negative	Yes
2191	VE15910	Miranda	Sucre	Petare	*P. geniculatus*	2010	Male	Negative	Yes
2192	VE57510	Miranda	Sucre	Petare	*P. geniculatus*	2010	Male	Negative	Yes
2193	VE41610	Miranda	Sucre	Petare	*P. geniculatus*	2010	Male	Negative	Yes
2194	VE65410	Miranda	Sucre	Petare	*P. geniculatus*	2010	Male	Negative	Yes
2195	VE10210	Miranda	Sucre	Petare	*P. geniculatus*	2010	Male	Negative	Yes
2196	VE73810	Miranda	Sucre	Petare	*P. geniculatus*	2010	Male	Negative	Yes
2197	VE73010	Miranda	Sucre	Petare	*P. geniculatus*	2010	Male	Negative	Yes
2198	VE56410	Miranda	Sucre	Petare	*P. geniculatus*	2010	Male	Negative	Yes
2199	VE138610	Miranda	Sucre	Petare	*P. geniculatus*	2010	Male	Positive	No
2200	VE130510	Miranda	Sucre	Petare	*P. geniculatus*	2010	Male	Positive	No
2201	VE142410	Miranda	Sucre	Petare	*P. geniculatus*	2010	Male	Positive	No
2202	VE132410	Miranda	Sucre	Petare	*P. geniculatus*	2010	Male	Positive	No
2203	VE133010	Miranda	Sucre	Petare	*P. geniculatus*	2010	Male	Positive	No
2204	VE136610	Miranda	Sucre	Petare	*P. geniculatus*	2010	Male	Positive	No
2205	VE54010	Miranda	Sucre	Petare	*P. geniculatus*	2010	Male	Positive	No
2206	VE1610	Miranda	Sucre	Petare	*P. geniculatus*	2010	Male	Positive	No
2207	VE90710	Miranda	Sucre	Petare	*P. geniculatus*	2010	Male	Positive	No
2208	VE96910	Miranda	Sucre	Petare	*P. geniculatus*	2010	Male	Positive	No
2209	VE58810	Miranda	Sucre	Petare	*P. geniculatus*	2010	Male	Positive	No
2210	VE23810	Miranda	Sucre	Petare	*P. geniculatus*	2010	Male	Positive	No
2211	VE91910	Miranda	Sucre	Petare	*P. geniculatus*	2010	Male	Positive	No
2212	VE56810	Miranda	Sucre	Petare	*P. geniculatus*	2010	Male	Positive	No
2213	VE85710A	Miranda	Sucre	Petare	*P. geniculatus*	2010	Male	Positive	No
2214	VE85710B	Miranda	Sucre	Petare	*P. geniculatus*	2010	Male	NA	NA
2215	VE85710C	Miranda	Sucre	Petare	*P. geniculatus*	2010	Male	NA	NA
2216	VE85710D	Miranda	Sucre	Petare	*P. geniculatus*	2010	Male	NA	NA
2217	VE47910	Miranda	Sucre	Petare	*P. geniculatus*	2010	Male	Positive	No
2218	VE47110	Miranda	Sucre	Petare	*P. geniculatus*	2010	Male	Positive	No
2219	VE19810	Miranda	Sucre	Petare	*P. geniculatus*	2010	Male	Positive	No
2220	VE80310	Miranda	Sucre	Petare	*P. geniculatus*	2010	Male	Positive	No
2221	VE57210A	Miranda	Sucre	Petare	*P. geniculatus*	2010	Male	Positive	No
2222	VE57210B	Miranda	Sucre	Petare	*P. geniculatus*	2010	Female	NA	NA
2223	VE57210C	Miranda	Sucre	Petare	*P. geniculatus*	2010	Male	NA	NA
2224	VE57210D	Miranda	Sucre	Petare	*P. geniculatus*	2010	Male	NA	NA

2225	VE73610	Miranda	Sucre	Petare	*P. geniculatus*	2010	Male	Positive	No
2226	VE72210A	Miranda	Sucre	Petare	*P. geniculatus*	2010	Female	Positive	No
2227	VE72210B	Miranda	Sucre	Petare	*P. geniculatus*	2010	Male	Positive	No
2228	VE14710	Miranda	Sucre	Petare	*P. geniculatus*	2010	Male	Positive	No
2229	VE92810	Miranda	Sucre	Petare	*P. geniculatus*	2010	Male	Positive	No
2230	VE61410	Miranda	Sucre	Petare	*P. geniculatus*	2010	Male	Positive	No
2231	VE78010	Miranda	Sucre	Petare	*P. geniculatus*	2010	Male	Positive	No
2232	VE116910	Miranda	Sucre	Petare	*P. geniculatus*	2010	Male	Positive	No
2233	VE12610A	Miranda	Sucre	Petare	*P. geniculatus*	2010	Male	NA	NA
2234	VE12610B	Miranda	Sucre	Petare	*P. geniculatus*	2010	Male	NA	NA
2235	VE12610C	Miranda	Sucre	Petare	*P. geniculatus*	2010	Male	Positive	No
2236	VE25710A	Miranda	Sucre	Petare	*P. geniculatus*	2010	Female	NA	NA
2237	VE25710B	Miranda	Sucre	Petare	*P. geniculatus*	2010	Male	Positive	No
2238	VE95010	Miranda	Sucre	Petare	*P. geniculatus*	2010	Male	Positive	No
2239	VE2510	Miranda	Sucre	Petare	*P. geniculatus*	2010	Male	Positive	No
2240	VE34310	Miranda	Sucre	Petare	*P. geniculatus*	2010	Male	Positive	No
2241	VE78410	Miranda	Sucre	Petare	*P. geniculatus*	2010	Male	Positive	No
2242	VE73710A	Miranda	Sucre	Petare	*P. geniculatus*	2010	Male	Positive	No
2243	VE73710B	Miranda	Sucre	Petare	*P. geniculatus*	2010	Male	Positive	No
2244	VE107410	Miranda	Sucre	Petare	*P. geniculatus*	2010	Male	Positive	No
2245	VE24610	Miranda	Sucre	Petare	*P. geniculatus*	2010	Male	Positive	No
2246	VE73510	Miranda	Sucre	Petare	*P. geniculatus*	2010	Male	Positive	No
2247	VE81810	Miranda	Sucre	Petare	*P. geniculatus*	2010	Male	Positive	No
2248	VE61310	Miranda	Sucre	Petare	*P. geniculatus*	2010	Male	Positive	No
2249	VE84310	Miranda	Sucre	Petare	*P. geniculatus*	2010	Male	Positive	No
2250	VE37410	Miranda	Sucre	Petare	*P. geniculatus*	2010	Male	Positive	No
2251	VE32310	Miranda	Sucre	Petare	*P. geniculatus*	2010	Male	Positive	No
2252	VE27610	Miranda	Sucre	Petare	*P. geniculatus*	2010	Male	Positive	No
2253	VE83510	Miranda	Sucre	Petare	*P. geniculatus*	2010	Male	Positive	No
2254	VE136210B	Miranda	Sucre	Petare	*P. geniculatus*	2010	Male	Positive	Yes
2255	VE134510	Miranda	Sucre	Petare	*P. geniculatus*	2010	Male	Positive	Yes
2256	VE141110	Miranda	Sucre	Petare	*P. geniculatus*	2010	Male	Positive	Yes
2257	VE46610	Miranda	Sucre	Petare	*P. geniculatus*	2010	Male	Positive	Yes
2258	VE66810	Miranda	Sucre	Petare	*P. geniculatus*	2010	Male	Positive	Yes
2259	VE31310	Miranda	Sucre	Petare	*P. geniculatus*	2010	Male	Positive	Yes
2260	VE23310	Miranda	Sucre	Petare	*P. geniculatus*	2010	Male	Positive	Yes
2261	VE26310A	Miranda	Sucre	Petare	*P. geniculatus*	2010	Female	NA	NA
2262	VE26310B	Miranda	Sucre	Petare	*P. geniculatus*	2010	Male	Positive	Yes
2263	VE15810B	Miranda	Sucre	Petare	*P. geniculatus*	2010	Male	Positive	Yes
2264	VE128910	Miranda	Sucre	Petare	*P. geniculatus*	2010	Male	Positive	Yes
2265	VE68310	Miranda	Sucre	Petare	*P. geniculatus*	2010	Male	Positive	Yes
2266	VE120210	Miranda	Sucre	Petare	*P. geniculatus*	2010	Male	Positive	Yes

2267	VE12810	Miranda	Sucre	Petare	*P. geniculatus*	2010	Male	Positive	Yes
2268	VE79610	Miranda	Sucre	Petare	*P. geniculatus*	2010	Male	Positive	Yes
2269	VE87310	Miranda	Sucre	Petare	*P. geniculatus*	2010	Male	Positive	Yes
2270	VE23210	Miranda	Sucre	Petare	*P. geniculatus*	2010	Male	Positive	Yes
2271	VE12210	Miranda	Sucre	Petare	*P. geniculatus*	2010	Male	Positive	Yes
2272	VE62910	Miranda	Sucre	Petare	*P. geniculatus*	2010	Male	Positive	Yes
2273	VE4910	Miranda	Sucre	Petare	*P. geniculatus*	2010	Male	Positive	Yes
2274	VE26210	Miranda	Sucre	Petare	*P. geniculatus*	2010	Male	Positive	Yes
2275	VE3610	Miranda	Sucre	Petare	*P. geniculatus*	2010	Male	Positive	Yes
2276	VE20910	Miranda	Sucre	Petare	*P. geniculatus*	2010	Male	Positive	Yes
2277	VE67410A	Miranda	Sucre	Petare	*P. geniculatus*	2010	Male	NA	NA
2278	VE67410B	Miranda	Sucre	Petare	*P. geniculatus*	2010	Male	Positive	Yes
2279	VE58610	Miranda	Sucre	Petare	*P. geniculatus*	2010	Male	Positive	Yes
2280	VE68610	Miranda	Sucre	Petare	*P. geniculatus*	2010	Male	Positive	Yes
2281	VE18110	Miranda	Sucre	Petare	*P. geniculatus*	2010	Male	Positive	Yes
2282	VE77810	Miranda	Sucre	Petare	*P. geniculatus*	2010	Male	Positive	Yes
2283	VE29010	Miranda	Sucre	Petare	*P. geniculatus*	2010	Male	NA	No
2284	VE·49310	Miranda	Sucre	Petare	*P. geniculatus*	2010	Male	NA	No
2285	VE46410	Miranda	Sucre	Petare	*P. geniculatus*	2010	Male	NA	NA
2286	VE130610	Miranda	Sucre	Petare	*P. geniculatus*	2010	Male	NA	NA
2287	VE134710	Miranda	Sucre	Petare	*P. geniculatus*	2010	Male	NA	NA
2288	VE128510A	Miranda	Sucre	Petare	*P. geniculatus*	2010	Male	NA	NA
2289	VE128510B	Miranda	Sucre	Petare	*P. geniculatus*	2010	Male	NA	NA
2290	VE120310B	Miranda	Sucre	Petare	*P. geniculatus*	2010	Male	NA	NA
2291	VE131910	Miranda	Sucre	Petare	*P. geniculatus*	2010	Male	NA	No
2292	VE9910	Miranda	Sucre	Petare	*P. geniculatus*	2010	Male	NA	No
2293	VE7810	Miranda	Sucre	Petare	*P. geniculatus*	2010	Male	NA	No
2294	VE35710A	Miranda	Sucre	Petare	*P. geniculatus*	2010	Male	NA	No
2295	VE35710B	Miranda	Sucre	Petare	*P. geniculatus*	2010	Female	NA	No
2296	VE18010A	Miranda	Sucre	Petare	*P. geniculatus*	2010	Male	Positive	Yes
2297	VE18010B	Miranda	Sucre	Petare	*P. geniculatus*	2010	Male	NA	NA
2298	VE103510	Miranda	Sucre	Petare	*P. geniculatus*	2010	Male	NA	No
2299	VE101910	Miranda	Sucre	Petare	*P. geniculatus*	2010	Male	NA	No
2300	VE107010	Miranda	Sucre	Petare	*P. geniculatus*	2010	Male	NA	No
2301	VE48410	Miranda	Sucre	Petare	*P. geniculatus*	2010	Male	NA	NA
2302	VE50510	Miranda	Sucre	Petare	*P. geniculatus*	2010	Male	NA	NA
2303	VE49110	Miranda	Sucre	Petare	*P. geniculatus*	2010	Male	NA	NA
2304	VE29310	Miranda	Sucre	Petare	*P. geniculatus*	2010	Nymph III	Positive	Yes
2305	VE19710	Miranda	Sucre	Petare	*P. geniculatus*	2010	Nymph V	Negative	Yes
2306	VE32210	Miranda	Sucre	Petare	*P. geniculatus*	2010	Nymph IV	Negative	No
2307	VE128510F	Miranda	Sucre	Petare	*T. nigromaculata*	2010	Female	NA	NA
2308	VE20410	Miranda	Sucre	Petare	*T. nigromaculata*	2010	Female	NA	No

2309	VE115410	Miranda	Sucre	Petare	*T. nigromaculata*	2010	Male	Negative	Yes
2310	VE81310	Miranda	El Hatillo	Santa Rosalía de Palermo	*P. geniculatus*	2010	Female	NA	No
2311	VE93910	Miranda	El Hatillo	Santa Rosalía de Palermo	*P. geniculatus*	2010	Female	NA	No
2312	VE102910	Miranda	El Hatillo	Santa Rosalía de Palermo	*P. geniculatus*	2010	Female	NA	No
2313	VE62310	Miranda	El Hatillo	Santa Rosalía de Palermo	*P. geniculatus*	2010	Female	NA	Yes
2314	VE46210	Miranda	El Hatillo	Santa Rosalía de Palermo	*P. geniculatus*	2010	Female	Negative	No
2315	VE72410	Miranda	El Hatillo	Santa Rosalía de Palermo	*P. geniculatus*	2010	Female	Negative	No
2316	VE17010	Miranda	El Hatillo	Santa Rosalía de Palermo	*P. geniculatus*	2010	Female	Negative	Yes
2317	VE108010	Miranda	El Hatillo	Santa Rosalía de Palermo	*P. geniculatus*	2010	Female	Negative	Yes
2318	VE127610	Miranda	El Hatillo	Santa Rosalía de Palermo	*P. geniculatus*	2010	Female	Positive	No
2319	VE55510	Miranda	El Hatillo	Santa Rosalía de Palermo	*P. geniculatus*	2010	Female	Positive	No
2320	VE119210	Miranda	El Hatillo	Santa Rosalía de Palermo	*P. geniculatus*	2010	Female	Positive	No
2321	VE11910	Miranda	El Hatillo	Santa Rosalía de Palermo	*P. geniculatus*	2010	Female	Positive	No
2322	VE67810	Miranda	El Hatillo	Santa Rosalía de Palermo	*P. geniculatus*	2010	Female	Positive	No
2323	VE44110	Miranda	El Hatillo	Santa Rosalía de Palermo	*P. geniculatus*	2010	Female	Positive	No
2324	VE124210	Miranda	El Hatillo	Santa Rosalía de Palermo	*P. geniculatus*	2010	Female	Positive	No
2325	VE114110	Miranda	El Hatillo	Santa Rosalía de Palermo	*P. geniculatus*	2010	Female	Positive	No
2326	VE140310A	Miranda	El Hatillo	Santa Rosalía de Palermo	*P. geniculatus*	2010	Female	Positive	Yes
2327	VE140510	Miranda	El Hatillo	Santa Rosalía de Palermo	*P. geniculatus*	2010	Female	Positive	Yes
2328	VE22310A	Miranda	El Hatillo	Santa Rosalía de Palermo	*P. geniculatus*	2010	Female	Positive	Yes
2329	VE22310B	Miranda	El Hatillo	Santa Rosalía de Palermo	*P. geniculatus*	2010	Female	Positive	Yes
2330	VE89010	Miranda	El Hatillo	Santa Rosalía de Palermo	*P. geniculatus*	2010	Female	Positive	Yes
2331	VE42910	Miranda	El Hatillo	Santa Rosalía de Palermo	*P. geniculatus*	2010	Female	Positive	Yes
2332	VE14310	Miranda	El Hatillo	Santa Rosalía de Palermo	*P. geniculatus*	2010	Female	Positive	Yes
2333	VE99010	Miranda	El Hatillo	Santa Rosalía de Palermo	*P. geniculatus*	2010	Female	Positive	Yes
2334	VE4110	Miranda	El Hatillo	Santa Rosalía de Palermo	*P. geniculatus*	2010	Female	Positive	Yes
2335	VE83210	Miranda	El Hatillo	Santa Rosalía de Palermo	*P. geniculatus*	2010	Female	Positive	Yes
2336	VE34810	Miranda	El Hatillo	Santa Rosalía de Palermo	*P. geniculatus*	2010	Female	Positive	Yes
2337	VE93210	Miranda	El Hatillo	Santa Rosalía de Palermo	*P. geniculatus*	2010	Female	Positive	Yes
2338	VE21110	Miranda	El Hatillo	Santa Rosalía de Palermo	*P. geniculatus*	2010	Female	Positive	Yes
2339	VE17210	Miranda	El Hatillo	Santa Rosalía de Palermo	*P. geniculatus*	2010	Female	Positive	Yes
2340	VE125010	Miranda	El Hatillo	Santa Rosalía de Palermo	*P. geniculatus*	2010	Female	NA	NA
2341	VE7210A	Miranda	El Hatillo	Santa Rosalía de Palermo	*P. geniculatus*	2010	Male	NA	No
2342	VE7210B	Miranda	El Hatillo	Santa Rosalía de Palermo	*P. geniculatus*	2010	Female	Negative	No
2343	VE3710	Miranda	El Hatillo	Santa Rosalía de Palermo	*P. geniculatus*	2010	Female	NA	No
2344	VE8010	Miranda	El Hatillo	Santa Rosalía de Palermo	*P. geniculatus*	2010	Female	NA	No
2345	VE89910	Miranda	El Hatillo	Santa Rosalía de Palermo	*P. geniculatus*	2010	Female	NA	No
2346	VE66110	Miranda	El Hatillo	Santa Rosalía de Palermo	*P. geniculatus*	2010	Female	NA	No
2347	VE135710	Miranda	El Hatillo	Santa Rosalía de Palermo	*P. geniculatus*	2010	Male	NA	NA
2348	VE143610	Miranda	El Hatillo	Santa Rosalía de Palermo	*P. geniculatus*	2010	Male	Negative	No
2349	VE48210	Miranda	El Hatillo	Santa Rosalía de Palermo	*P. geniculatus*	2010	Male	Negative	No
2350	VE76610	Miranda	El Hatillo	Santa Rosalía de Palermo	*P. geniculatus*	2010	Male	Negative	No

2351	VE52910	Miranda	El Hatillo	Santa Rosalía de Palermo	*P. geniculatus*	2010	Male	Negative	No
2352	VE93510	Miranda	El Hatillo	Santa Rosalía de Palermo	*P. geniculatus*	2010	Male	Negative	Yes
2353	VE16010A	Miranda	El Hatillo	Santa Rosalía de Palermo	*P. geniculatus*	2010	Female	NA	Yes
2354	VE16010B	Miranda	El Hatillo	Santa Rosalía de Palermo	*P. geniculatus*	2010	Male	Negative	Yes
2355	VE140710	Miranda	El Hatillo	Santa Rosalía de Palermo	*P. geniculatus*	2010	Male	Positive	No
2356	VE140310B	Miranda	El Hatillo	Santa Rosalía de Palermo	*P. geniculatus*	2010	Male	Positive	No
2357	VE135810	Miranda	El Hatillo	Santa Rosalía de Palermo	*P. geniculatus*	2010	Male	Positive	No
2358	VE133310	Miranda	El Hatillo	Santa Rosalía de Palermo	*P. geniculatus*	2010	Male	Positive	No
2359	VE79810	Miranda	El Hatillo	Santa Rosalía de Palermo	*P. geniculatus*	2010	Male	Positive	No
2360	VE31710	Miranda	El Hatillo	Santa Rosalía de Palermo	*P. geniculatus*	2010	Male	Positive	No
2361	VE6410	Miranda	El Hatillo	Santa Rosalía de Palermo	*P. geniculatus*	2010	Male	Positive	No
2362	VE48510	Miranda	El Hatillo	Santa Rosalía de Palermo	*P. geniculatus*	2010	Male	Positive	No
2363	VE115910	Miranda	El Hatillo	Santa Rosalía de Palermo	*P. geniculatus*	2010	Male	Positive	No
2364	VE50310	Miranda	El Hatillo	Santa Rosalía de Palermo	*P. geniculatus*	2010	Male	Positive	No
2365	VE22410B	Miranda	El Hatillo	Santa Rosalía de Palermo	*P. geniculatus*	2010	Male	Positive	No
2366	VE57310	Miranda	El Hatillo	Santa Rosalía de Palermo	*P. geniculatus*	2010	Male	Positive	No
2367	VE65210	Miranda	El Hatillo	Santa Rosalía de Palermo	*P. geniculatus*	2010	Male	Positive	No
2368	VE67710	Miranda	El Hatillo	Santa Rosalía de Palermo	*P. geniculatus*	2010	Male	Positive	No
2369	VE118710	Miranda	El Hatillo	Santa Rosalía de Palermo	*P. geniculatus*	2010	Male	Positive	No
2370	VE115710	Miranda	El Hatillo	Santa Rosalía de Palermo	*P. geniculatus*	2010	Male	Positive	No
2371	VE73210A	Miranda	El Hatillo	Santa Rosalía de Palermo	*P. geniculatus*	2010	Male	Positive	No
2372	VE73210B	Miranda	El Hatillo	Santa Rosalía de Palermo	*P. geniculatus*	2010	Male	Positive	No
2373	VE73210C	Miranda	El Hatillo	Santa Rosalía de Palermo	*P. geniculatus*	2010	Male	Positive	No
2374	VE132610	Miranda	El Hatillo	Santa Rosalía de Palermo	*P. geniculatus*	2010	Male	Positive	Yes
2375	VE129010	Miranda	El Hatillo	Santa Rosalía de Palermo	*P. geniculatus*	2010	Male	Positive	Yes
2376	VE69510	Miranda	El Hatillo	Santa Rosalía de Palermo	*P. geniculatus*	2010	Male	Positive	Yes
2377	VE10610	Miranda	El Hatillo	Santa Rosalía de Palermo	*P. geniculatus*	2010	Male	Positive	Yes
2378	VE109710	Miranda	El Hatillo	Santa Rosalía de Palermo	*P. geniculatus*	2010	Male	Positive	Yes
2379	VE16210A	Miranda	El Hatillo	Santa Rosalía de Palermo	*T. maculata*	2010	Male	NA	No
2380	VE16210B	Miranda	El Hatillo	Santa Rosalía de Palermo	*P. geniculatus*	2010	Male	NA	No
2381	VE16210C	Miranda	El Hatillo	Santa Rosalía de Palermo	*P. geniculatus*	2010	Male	NA	No
2382	VE16210D	Miranda	El Hatillo	Santa Rosalía de Palermo	*P. geniculatus*	2010	Male	NA	No
2383	VE16210E	Miranda	El Hatillo	Santa Rosalía de Palermo	*P. geniculatus*	2010	Male	NA	No
2384	VE15610	Miranda	El Hatillo	Santa Rosalía de Palermo	*P. geniculatus*	2010	Male	NA	No
2385	VE30010	Miranda	El Hatillo	Santa Rosalía de Palermo	*P. geniculatus*	2010	Male	NA	Yes
2386	VE134810	Miranda	El Hatillo	Santa Rosalía de Palermo	*P. geniculatus*	2010	Nymph III	NA	NA
2387	VE134010	Miranda	El Hatillo	Santa Rosalía de Palermo	*T. nigromaculata*	2010	Male	Positive	No
2388	VE97810D	Miranda	El Hatillo	Santa Rosalía de Palermo	*P. geniculatus*	2010	Male	Negative	Yes
2389	VE97810C	Miranda	El Hatillo	Santa Rosalía de Palermo	*P. geniculatus*	2010	Male	Negative	Yes
2390	VE97810B	Miranda	El Hatillo	Santa Rosalía de Palermo	*T. nigromaculata*	2010	Female	NA	Yes
2391	VE97810A	Miranda	El Hatillo	Santa Rosalía de Palermo	*T. nigromaculata*	2010	Female	Positive	Yes
2392	VE97810	Miranda	El Hatillo	Santa Rosalía de Palermo	*T. nigromaculata*	2010	Male	Positive	Yes

2393	VE34910	Miranda	El Hatillo	Santa Rosalía de Palermo	*T. nigromaculata*	2010	Male	NA	No
2394	VE90410	Miranda	El Hatillo	Santa Rosalía de Palermo	*P. geniculatus*	2010	Female	Positive	Yes
2395	VE100010	Miranda	El Hatillo	Santa Rosalía de Palermo	*P. geniculatus*	2010	Female	NA	Yes
2396	VE96310	Miranda	El Hatillo	Santa Rosalía de Palermo	*P. geniculatus*	2010	Female	Positive	No
2397	VE23510	Miranda	El Hatillo	Santa Rosalía de Palermo	*P. geniculatus*	2010	Female	NA	Yes
2398	VE24011	DC	Libertador	23 de Enero	*P. geniculatus*	2011	Male	NA	NA
2399	VE29311	DC	Libertador	23 de Enero	*P. geniculatus*	2011	Female	NA	No
2400	VE31811	DC	Libertador	23 de Enero	*P. geniculatus*	2011	Male	Positive	No
2401	VE11511	DC	Libertador	23 de Enero	*P. geniculatus*	2011	Female	Positive	Yes
2402	VE48411	DC	Libertador	Altagracia	*P. geniculatus*	2011	Male	Positive	No
2403	VE0211	DC	Libertador	Antímano	*P. geniculatus*	2011	Male	NA	NA
2404	VE20611	DC	Libertador	Antímano	*P. geniculatus*	2011	Female	NA	NA
2405	VE17611	DC	Libertador	Antímano	*P. geniculatus*	2011	Female	NA	NA
2406	VE36811	DC	Libertador	Antímano	*P. geniculatus*	2011	Male	Negative	Yes
2407	VE13611	DC	Libertador	Antímano	*P. geniculatus*	2011	Female	Positive	Yes
2408	VE44711	DC	Libertador	Antímano	*P. geniculatus*	2011	Female	Positive	Yes
2409	VE33211	DC	Libertador	Antímano	*P. geniculatus*	2011	Female	Positive	Yes
2410	VE5811	DC	Libertador	Candelaria	*P. geniculatus*	2011	Male	Positive	No
2411	VE32611	DC	Libertador	Candelaria	*P. geniculatus*	2011	Male	Positive	No
2412	VE11111	DC	Libertador	Candelaria	*P. geniculatus*	2011	Female	Positive	Yes
2413	VE1511	DC	Libertador	Caricuao	*P. geniculatus*	2011	Male	NA	NA
2414	VE26811	DC	Libertador	Caricuao	*P. geniculatus*	2011	Female	NA	NA
2415	VE33611	DC	Libertador	Caricuao	*P. geniculatus*	2011	Female	Positive	No
2416	VE8211	DC	Libertador	Caricuao	*P. geniculatus*	2011	Female	Positive	Yes
2417	VE15911	DC	Libertador	Caricuao	*P. geniculatus*	2011	Male	Positive	Yes
2418	VE45411	DC	Libertador	Coche	*P. geniculatus*	2011	Female	NA	Yes
2419	VE100A11	DC	Libertador	El Junquito	*P. geniculatus*	2011	Male	NA	NA
2420	VE26111	DC	Libertador	El Paraiso	*P. geniculatus*	2011	Female	NA	NA
2421	VE6211	DC	Libertador	El Paraiso	*P. geniculatus*	2011	Female	Negative	Yes
2422	VE25411	DC	Libertador	El Paraiso	*P. geniculatus*	2011	Female	Positive	No
2423	VE35911	DC	Libertador	El Paraiso	*P. geniculatus*	2011	Female	Positive	No
2424	VE55211	DC	Libertador	El Paraiso	*P. geniculatus*	2011	Male	Positive	No
2425	VE51811B	DC	Libertador	El Recreo	*P. geniculatus*	2011	Female	NA	NA
2426	VE51811C	DC	Libertador	El Recreo	*P. geniculatus*	2011	Female	NA	NA
2427	VE46011	DC	Libertador	El Recreo	*P. geniculatus*	2011	Female	Positive	No
2428	VE16811	DC	Libertador	El Recreo	*P. geniculatus*	2011	Female	Positive	No
2429	VE51811A	DC	Libertador	El Recreo	*P. geniculatus*	2011	Female	Positive	No
2430	VE32511	DC	Libertador	El Recreo	*P. geniculatus*	2011	Female	Positive	Yes
2431	VE37611A	DC	Libertador	El Valle	*P. geniculatus*	2011	Female	NA	NA
2432	VE37611B	DC	Libertador	El Valle	*P. geniculatus*	2011	Male	NA	NA
2433	VE31311	DC	Libertador	El Valle	*P. geniculatus*	2011	Male	NA	No
2434	VE31711	DC	Libertador	El Valle	*P. geniculatus*	2011	Male	NA	Yes

2435	VE44411	DC	Libertador	El Valle	*P. geniculatus*	2011	Female	Negative	Yes
2436	VE30311	DC	Libertador	El Valle	*P. geniculatus*	2011	Female	Positive	Yes
2437	VE100B11	DC	Libertador	El Junquito	*P. geniculatus*	2011	Male	NA	NA
2438	VE48911A	DC	Libertador	El Junquito	*P. geniculatus*	2011	Female	NA	NA
2439	VE48911B	DC	Libertador	El Junquito	*P. geniculatus*	2011	Female	NA	NA
2440	VE21511	DC	Libertador	El Junquito	*P. geniculatus*	2011	Female	NA	Yes
2441	VE10611	DC	Libertador	El Junquito	*P. geniculatus*	2011	Male	Negative	No
2442	VE41011A	DC	Libertador	El Junquito	*P. geniculatus*	2011	Female	Negative	No
2443	VE41011B	DC	Libertador	El Junquito	*P. geniculatus*	2011	Male	Negative	No
2444	VE51311A	DC	Libertador	El Junquito	*P. geniculatus*	2011	Female	Negative	Yes
2445	VE51311B	DC	Libertador	El Junquito	*P. geniculatus*	2011	Female	Negative	Yes
2446	VE1611	DC	Libertador	El Junquito	*P. geniculatus*	2011	Male	Positive	No
2447	VE14011	DC	Libertador	El Junquito	*P. geniculatus*	2011	Male	Positive	No
2448	VE20511	DC	Libertador	El Junquito	*P. geniculatus*	2011	Female	Positive	No
2449	VE42311	DC	Libertador	El Junquito	*P. geniculatus*	2011	Male	Positive	No
2450	VE27311	DC	Libertador	El Junquito	*P. geniculatus*	2011	Female	Positive	No
2451	VE11811	DC	Libertador	El Junquito	*P. geniculatus*	2011	Male	Positive	Yes
2452	VE14411	DC	Libertador	El Junquito	*P. geniculatus*	2011	Male	Positive	Yes
2453	VE16211	DC	Libertador	El Junquito	*P. geniculatus*	2011	Female	Positive	Yes
2454	VE19311	DC	Libertador	El Junquito	*P. geniculatus*	2011	Female	Positive	Yes
2455	VE28211A	DC	Libertador	El Junquito	*P. geniculatus*	2011	Male	Positive	Yes
2456	VE32311	DC	Libertador	El Junquito	*P. geniculatus*	2011	Female	Positive	Yes
2457	VE43511	DC	Libertador	El Junquito	*P. geniculatus*	2011	Female	Positive	Yes
2458	VE44311	DC	Libertador	El Junquito	*P. geniculatus*	2011	Male	Positive	Yes
2459	VE46911	DC	Libertador	El Junquito	*P. geniculatus*	2011	Male	Positive	Yes
2460	VE24311	DC	Libertador	El Junquito	*P. geniculatus*	2011	Female	Positive	Yes
2461	VE38911	DC	Libertador	El Junquito	*P. geniculatus*	2011	Male	Positive	Yes
2462	VE40311	DC	Libertador	El Junquito	*P. geniculatus*	2011	Female	Positive	Yes
2463	VE31911	DC	Libertador	El Junquito	*P. geniculatus*	2011	Female	Positive	Yes
2464	VE22811	DC	Libertador	El Junquito	*P. geniculatus*	2011	Female	Positive	Yes
2465	VE43111	DC	Libertador	El Junquito	*P. geniculatus*	2011	Female	Positive	Yes
2466	VE17311	DC	Libertador	La Pastora	*P. geniculatus*	2011	Male	NA	NA
2467	VE235A11	DC	Libertador	La Pastora	*P. geniculatus*	2011	Female	NA	NA
2468	VE32411	DC	Libertador	La Pastora	*P. geniculatus*	2011	Female	NA	NA
2469	VE38011	DC	Libertador	La Pastora	*P. geniculatus*	2011	Male	NA	NA
2470	VE48511B	DC	Libertador	La Pastora	*P. geniculatus*	2011	Female	NA	NA
2471	VE4211	DC	Libertador	La Pastora	*P. geniculatus*	2011	Female	NA	NA
2472	VE43611	DC	Libertador	La Pastora	*P. geniculatus*	2011	Female	NA	Yes
2473	VE36411	DC	Libertador	La Pastora	*P. geniculatus*	2011	Male	Negative	No
2474	VE24911	DC	Libertador	La Pastora	*P. geniculatus*	2011	Female	Negative	Yes
2475	VE5611	DC	Libertador	La Pastora	*P. geniculatus*	2011	Male	Positive	No
2476	VE29011B	DC	Libertador	La Pastora	*P. geniculatus*	2011	Male	Positive	No

2477	VE23711	DC	Libertador	La Pastora	*P. geniculatus*	2011	Female	Positive	No
2478	VE34811	DC	Libertador	La Pastora	*P. geniculatus*	2011	Female	Positive	No
2479	VE36211	DC	Libertador	La Pastora	*P. geniculatus*	2011	Female	Positive	No
2480	VE42011	DC	Libertador	La Pastora	*P. geniculatus*	2011	Male	Positive	No
2481	VE45011	DC	Libertador	La Pastora	*P. geniculatus*	2011	Male	Positive	No
2482	VE1811	DC	Libertador	La Pastora	*P. geniculatus*	2011	Male	Positive	No
2483	VE37911	DC	Libertador	La Pastora	*P. geniculatus*	2011	Male	Positive	No
2484	VE52311	DC	Libertador	La Pastora	*P. geniculatus*	2011	Male	Positive	No
2485	VE34111	DC	Libertador	La Pastora	*P. geniculatus*	2011	Male	Positive	No
2486	VE48511A	DC	Libertador	La Pastora	*P. geniculatus*	2011	Male	Positive	No
2487	VE24211	DC	Libertador	La Pastora	*P. geniculatus*	2011	Female	Positive	Yes
2488	VE25611	DC	Libertador	La Pastora	*P. geniculatus*	2011	Male	Positive	Yes
2489	VE28211B	DC	Libertador	La Pastora	*P. geniculatus*	2011	Male	Positive	Yes
2490	VE29011A	DC	Libertador	La Pastora	*P. geniculatus*	2011	Female	Positive	Yes
2491	VE15711	DC	Libertador	La Pastora	*P. geniculatus*	2011	Male	Positive	Yes
2492	VE20711	DC	Libertador	La Pastora	*P. geniculatus*	2011	Female	Positive	Yes
2493	VE235B11	DC	Libertador	La Pastora	*P. geniculatus*	2011	Female	Positive	Yes
2494	VE28511	DC	Libertador	La Pastora	*P. geniculatus*	2011	Female	Positive	Yes
2495	VE38611	DC	Libertador	La Pastora	*P. geniculatus*	2011	Female	Positive	Yes
2496	VE39911	DC	Libertador	La Pastora	*P. geniculatus*	2011	Male	Positive	Yes
2497	VE40111	DC	Libertador	La Pastora	*P. geniculatus*	2011	Male	Positive	Yes
2498	VE51211	DC	Libertador	La Pastora	*P. geniculatus*	2011	Male	Positive	Yes
2499	VE54811	DC	Libertador	La Pastora	*P. geniculatus*	2011	Female	Positive	Yes
2500	VE8811	DC	Libertador	La Pastora	*P. geniculatus*	2011	Female	Positive	Yes
2501	VE97A11	DC	Libertador	La Pastora	*P. geniculatus*	2011	Female	Positive	Yes
2502	VE97B11	DC	Libertador	La Pastora	*P. geniculatus*	2011	Female	Positive	Yes
2503	VE20111	DC	Libertador	La Pastora	*P. geniculatus*	2011	Male	Positive	Yes
2504	VE54511	DC	Libertador	La Pastora	*P. geniculatus*	2011	Female	Positive	Yes
2505	VE35411	DC	Libertador	La Vega	*P. geniculatus*	2011	Female	NA	Yes
2506	VE14711	DC	Libertador	La Vega	*P. geniculatus*	2011	Male	NA	NA
2507	VE21911	DC	Libertador	La Vega	*P. geniculatus*	2011	Female	Negative	Yes
2508	VE8911	DC	Libertador	La Vega	*P. geniculatus*	2011	Male	Positive	No
2509	VE10511	DC	Libertador	La Vega	*P. geniculatus*	2011	Female	Positive	No
2510	VE33711	DC	Libertador	La Vega	*P. geniculatus*	2011	Male	Positive	No
2511	VE39711	DC	Libertador	La Vega	*P. geniculatus*	2011	Male	Positive	No
2512	VE32011	DC	Libertador	La Vega	*P. geniculatus*	2011	Male	Positive	No
2513	VE34011	DC	Libertador	La Vega	*P. geniculatus*	2011	Female	Positive	Yes
2514	VE53311A	DC	Libertador	La Vega	*P. geniculatus*	2011	Female	Positive	Yes
2515	VE53311B	DC	Libertador	La Vega	*P. geniculatus*	2011	Female	Positive	Yes
2516	VE53311C	DC	Libertador	La Vega	*P. geniculatus*	2011	Female	Positive	Yes
2517	VE42211	DC	Libertador	San Agustín	*P. geniculatus*	2011	Female	Positive	Yes
2518	VE14111	DC	Libertador	San Bernardino	*P. geniculatus*	2011	Male	NA	NA

2519	VE2111	DC	Libertador	San Bernardino	*P. geniculatus*	2011	Female	Positive	No
2520	VE171B11	DC	Libertador	San Bernardino	*P. geniculatus*	2011	Male	Positive	No
2521	VE38111	DC	Libertador	San Bernardino	*P. geniculatus*	2011	Female	Positive	No
2522	VE51911	DC	Libertador	San Bernardino	*P. geniculatus*	2011	Female	Positive	No
2523	VE35311	DC	Libertador	San Bernardino	*P. geniculatus*	2011	Female	Positive	No
2524	VE48611	DC	Libertador	San Bernardino	*P. geniculatus*	2011	Male	Positive	No
2525	VE171A11	DC	Libertador	San Bernardino	*P. geniculatus*	2011	Female	Positive	Yes
2526	VE36311	DC	Libertador	San José	*P. geniculatus*	2011	Male	NA	NA
2527	VE22111	DC	Libertador	San José	*P. geniculatus*	2011	Female	NA	Yes
2528	VE30011	DC	Libertador	San José	*P. geniculatus*	2011	Female	Positive	No
2529	VE11411	DC	Libertador	San José	*P. geniculatus*	2011	Female	Positive	Yes
2530	VE5711	DC	Libertador	San Pedro	*P. geniculatus*	2011	Female	Positive	No
2531	VE12111	DC	Libertador	Sucre	*P. geniculatus*	2011	Female	NA	NA
2532	VE45211	DC	Libertador	Sucre	*P. geniculatus*	2011	Female	NA	NA
2533	VE18411	DC	Libertador	Sucre	*P. geniculatus*	2011	Female	NA	NA
2534	VE37011	DC	Libertador	Sucre	*P. geniculatus*	2011	Male	NA	NA
2535	VE46311	DC	Libertador	Sucre	*P. geniculatus*	2011	Male	NA	NA
2536	VE4511	DC	Libertador	Sucre	*P. geniculatus*	2011	Female	NA	Yes
2537	VE241A11	DC	Libertador	Sucre	*P. geniculatus*	2011	Female	NA	Yes
2538	VE45311	DC	Libertador	Sucre	*P. geniculatus*	2011	Female	NA	Yes
2539	VE31011	DC	Libertador	Sucre	*P. geniculatus*	2011	Male	NA	Yes
2540	VE37111	DC	Libertador	Sucre	*P. geniculatus*	2011	Female	NA	Yes
2541	VE48311	DC	Libertador	Sucre	*P. geniculatus*	2011	Female	NA	Yes
2542	VE12611	DC	libertador	Sucre	*P. geniculatus*	2011	Male	Negative	Yes
2543	VE25111	DC	Libertador	Sucre	*P. geniculatus*	2011	Female	Positive	No
2544	VE4311	DC	Libertador	Sucre	*P. geniculatus*	2011	Male	Positive	No
2545	VE13511	DC	Libertador	Sucre	*P. geniculatus*	2011	Male	Positive	No
2546	VE29511	DC	Libertador	Sucre	*P. geniculatus*	2011	Male	Positive	No
2547	VE37411	DC	Libertador	Sucre	*P. geniculatus*	2011	Female	Positive	No
2548	VE28911	DC	Libertador	Sucre	*P. geniculatus*	2011	Female	Positive	No
2549	VE40911	DC	Libertador	Sucre	*P. geniculatus*	2011	Female	Positive	No
2550	VE107A11	DC	Libertador	Sucre	*P. geniculatus*	2011	Male	Positive	No
2551	VE107B11	DC	Libertador	Sucre	*P. geniculatus*	2011	Male	Positive	No
2552	VE8411	DC	Libertador	Sucre	*P. geniculatus*	2011	Male	Positive	No
2553	VE42911	DC	Libertador	Sucre	*P. geniculatus*	2011	Male	Positive	Yes
2554	VE25211	DC	Libertador	Sucre	*P. geniculatus*	2011	Female	Positive	Yes
2555	VE1211	DC	Libertador	Sucre	*P. geniculatus*	2011	Nymph V	Positive	Yes
2556	VE15411	DC	Libertador	Sucre	*P. geniculatus*	2011	Female	Positive	Yes
2557	VE0711	DC	Libertador	Sucre	*P. geniculatus*	2011	Male	Positive	Yes
2558	VE15811	DC	Libertador	Sucre	*P. geniculatus*	2011	Female	Positive	Yes
2559	VE21211	DC	Libertador	Sucre	*P. geniculatus*	2011	Female	Positive	Yes
2560	VE241B11	DC	Libertador	Sucre	*P. geniculatus*	2011	Female	Positive	Yes

2561	VE35611	DC	Libertador	Sucre	*P. geniculatus*	2011	Female	Positive	Yes
2562	VE1311	DC	Libertador	Sucre	*P. geniculatus*	2011	Female	Positive	Yes
2563	VE45511	DC	Libertador	Sucre	*P. geniculatus*	2011	Female	Positive	Yes
2564	VE55111	DC	Libertador	Sucre	*P. geniculatus*	2011	Male	Positive	Yes
2565	VE11211	DC	Libertador	Sucre	*P. geniculatus*	2011	Male	Positive	Yes
2566	VE8511	DC	Libertador	Sucre	*P. geniculatus*	2011	Female	Positive	Yes
2567	VE4911	Miranda	Sucre	Caucagüita	*P. geniculatus*	2011	Female	NA	NA
2568	VE39511	Miranda	Sucre	Caucagüita	*P. geniculatus*	2011	Female	NA	Yes
2569	VE41811	Miranda	Sucre	Caucagüita	*P. geniculatus*	2011	Female	Positive	No
2570	VE16311	Miranda	Sucre	Caucagüita	*P. geniculatus*	2011	Female	Positive	No
2571	VE8711	Miranda	Sucre	Caucagüita	*P. geniculatus*	2011	Female	Positive	Yes
2572	VE11911	Miranda	Sucre	Caucagüita	*P. geniculatus*	2011	Female	Positive	Yes
2573	VE43911	Miranda	Sucre	Caucagüita	*P. geniculatus*	2011	Female	Positive	Yes
2574	VE52111	Miranda	Chacao	Chacao	*P. geniculatus*	2011	Male	NA	NA
2575	VE51611C	Miranda	Chacao	Chacao	*P. geniculatus*	2011	Female	Negative	No
2576	VE51611B	Miranda	Chacao	Chacao	*P. geniculatus*	2011	Male	Positive	No
2577	VE9211	Miranda	Chacao	Chacao	*P. geniculatus*	2011	Male	Positive	Yes
2578	VE46411	Miranda	Chacao	Chacao	*P. geniculatus*	2011	Female	Positive	Yes
2579	VE51611A	Miranda	Chacao	Chacao	*P. geniculatus*	2011	Female	Positive	Yes
2580	VE6411	Miranda	baruta	El Cafetal	*P. geniculatus*	2011	Male	NA	NA
2581	VE41411	Miranda	Baruta	El Cafetal	*P. geniculatus*	2011	Nymph IV	NA	NA
2582	VE27711	Miranda	Baruta	El Cafetal	*P. geniculatus*	2011	Nymph IV	NA	NA
2583	VE45711	Miranda	Baruta	El Cafetal	*P. geniculatus*	2011	Female	NA	Yes
2584	VE35511	Miranda	Baruta	El Cafetal	*P. geniculatus*	2011	Female	NA	Yes
2585	VE22211	Miranda	Baruta	El Cafetal	*P. geniculatus*	2011	Female	Negative	No
2586	VE6011	Miranda	Baruta	El Cafetal	*P. geniculatus*	2011	Male	Negative	No
2587	VE10811	Miranda	Baruta	El Cafetal	*P. geniculatus*	2011	Male	Positive	No
2588	VE17011	Miranda	Baruta	El Cafetal	*P. geniculatus*	2011	Female	Positive	No
2589	VE37211A	Miranda	Baruta	El Cafetal	*P. geniculatus*	2011	Female	Positive	No
2590	VE37211B	Miranda	Baruta	El Cafetal	*P. geniculatus*	2011	Male	Positive	No
2591	VE52811	Miranda	Baruta	El Cafetal	*P. geniculatus*	2011	Female	Positive	No
2592	VE53411	Miranda	Baruta	El Cafetal	*P. geniculatus*	2011	Female	Positive	No
2593	VE8011	Miranda	Baruta	El Cafetal	*P. geniculatus*	2011	Male	Positive	No
2594	VE26911	Miranda	Baruta	El Cafetal	*P. geniculatus*	2011	Female	Positive	No
2595	VE50511	Miranda	Baruta	El Cafetal	*P. geniculatus*	2011	Female	Positive	No
2596	VE13911	Miranda	Baruta	El Cafetal	*P. geniculatus*	2011	Female	Positive	No
2597	VE29611	Miranda	Baruta	El Cafetal	*P. geniculatus*	2011	Female	Positive	No
2598	VE17811	Miranda	Baruta	El Cafetal	*P. geniculatus*	2011	Male	Positive	No
2599	VE27111	Miranda	Baruta	El Cafetal	*P. geniculatus*	2011	Female	Positive	Yes
2600	VE33411	Miranda	Baruta	El Cafetal	*P. geniculatus*	2011	Female	Positive	Yes
2601	VE21611	Miranda	Baruta	El Cafetal	*P. geniculatus*	2011	Female	Positive	Yes
2602	VE23811	Miranda	Baruta	El Cafetal	*P. geniculatus*	2011	Female	Positive	Yes

2603	VE35211	Miranda	Baruta	El Cafetal	*P. geniculatus*	2011	Male	Positive	Yes
2604	VE50111	Miranda	Baruta	El Cafetal	*P. geniculatus*	2011	Male	Positive	Yes
2605	VE22611	Miranda	Sucre	Filas de mariche	*P. geniculatus*	2011	Female	NA	NA
2606	VE263A11	Miranda	Sucre	Filas de mariche	*P. geniculatus*	2011	Male	NA	NA
2607	VE34711	Miranda	Sucre	Filas de mariche	*P. geniculatus*	2011	Female	NA	No
2608	VE263B11	Miranda	Sucre	Filas de mariche	*P. geniculatus*	2011	Female	NA	Yes
2609	VE26411	Miranda	Sucre	Filas de mariche	*P. geniculatus*	2011	Male	NA	Yes
2610	VE45611	Miranda	Sucre	Filas de mariche	*P. geniculatus*	2011	Female	NA	Yes
2611	VE33911	Miranda	Sucre	Filas de mariche	*P. geniculatus*	2011	Female	Negative	No
2612	VE19811	Miranda	Sucre	Filas de mariche	*P. geniculatus*	2011	Female	Negative	Yes
2613	VE48711	Miranda	Sucre	Filas de mariche	*P. geniculatus*	2011	Nymph III	Negative	Yes
2614	VE40811	Miranda	Sucre	Filas de mariche	*P. geniculatus*	2011	Female	Negative	Yes
2615	VE41711	Miranda	Sucre	Filas de mariche	*P. geniculatus*	2011	Female	Negative	Yes
2616	VE23911	Miranda	Sucre	Filas de mariche	*T. maculata*	2011	Male	Positive	No
2617	VE34611B	Miranda	Sucre	Filas de mariche	*P. geniculatus*	2011	Male	Positive	No
2618	VE35111	Miranda	Sucre	Filas de mariche	*P. geniculatus*	2011	Female	Positive	No
2619	VE30611	Miranda	Sucre	Filas de mariche	*T. maculata*	2011	Female	Positive	No
2620	VE2911	Miranda	Sucre	Filas de mariche	*P. geniculatus*	2011	Male	Positive	Yes
2621	VE25311	Miranda	Sucre	Filas de mariche	*P. geniculatus*	2011	Female	Positive	Yes
2622	VE43411	Miranda	Sucre	Filas de mariche	*P. geniculatus*	2011	Female	Positive	Yes
2623	VE53011	Miranda	Sucre	Filas de mariche	*P. geniculatus*	2011	Female	Positive	Yes
2624	VE54011	Miranda	Sucre	Filas de mariche	*P. geniculatus*	2011	Female	Positive	Yes
2625	VE34611A	Miranda	Sucre	Filas de mariche	*P. geniculatus*	2011	Female	Positive	Yes
2626	VE38311	Miranda	Sucre	Filas de mariche	*P. geniculatus*	2011	Female	Positive	Yes
2627	VE28711	Miranda	Sucre	Filas de mariche	*P. geniculatus*	2011	Female	Positive	Yes
2628	VE26011	Miranda	Sucre	La Dolorita	*P. geniculatus*	2011	Male	Negative	No
2629	VE14911	Miranda	Sucre	La Dolorita	*P. geniculatus*	2011	Male	Positive	No
2630	VE19411	Miranda	Sucre	La Dolorita	*P. geniculatus*	2011	Female	Positive	Yes
2631	VE28411	Miranda	Sucre	La Dolorita	*P. geniculatus*	2011	Female	Positive	Yes
2632	VE42611	Miranda	Sucre	La Dolorita	*P. geniculatus*	2011	Female	Positive	Yes
2633	VE7211	Miranda	Baruta	Las Minas de Baruta	*P. geniculatus*	2011	Female	NA	NA
2634	VE10311	Miranda	Baruta	Las Minas de Baruta	*P. geniculatus*	2011	Male	NA	NA
2635	VE42411	Miranda	Baruta	Las Minas de Baruta	*P. geniculatus*	2011	Female	NA	Yes
2636	VE30111	Miranda	Baruta	Las Minas de Baruta	*P. geniculatus*	2011	Female	Negative	No
2637	VE12311	Miranda	Baruta	Las Minas de Baruta	*P. geniculatus*	2011	Nymph IV	Negative	Yes
2638	VE9311	Miranda	Baruta	Las Minas de Baruta	*P. geniculatus*	2011	Male	Positive	No
2639	VE12411	Miranda	Baruta	Las Minas de Baruta	*P. geniculatus*	2011	Male	Positive	No
2640	VE12511	Miranda	Baruta	Las Minas de Baruta	*P. geniculatus*	2011	Female	Positive	No
2641	VE32211	Miranda	Baruta	Las Minas de Baruta	*P. geniculatus*	2011	Female	Positive	No
2642	VE4811	Miranda	Baruta	Las Minas de Baruta	*P. geniculatus*	2011	Female	Positive	No
2643	VE21411	Miranda	Baruta	Las Minas de Baruta	*P. geniculatus*	2011	Male	Positive	No
2644	VE9411	Miranda	Baruta	Las Minas de Baruta	*P. geniculatus*	2011	Female	Positive	Yes

2645	VE28611	Miranda	Baruta	Las Minas de Baruta	*P. geniculatus*	2011	Female	Positive	Yes
2646	VE39811	Miranda	Baruta	Las Minas de Baruta	*P. geniculatus*	2011	Male	Positive	Yes
2647	VE19911	Miranda	Baruta	Las Minas de Baruta	*P. geniculatus*	2011	Female	Positive	Yes
2648	VE44811	Miranda	Sucre	Leoncio Martínez	*P. geniculatus*	2011	Female	NA	NA
2649	VE36711	Miranda	Sucre	Leoncio Martínez	*P. geniculatus*	2011	Male	NA	NA
2650	VE16611	Miranda	Sucre	Leoncio Martínez	*P. geniculatus*	2011	Male	NA	No
2651	VE3311	Miranda	Sucre	Leoncio Martínez	*P. geniculatus*	2011	Male	NA	Yes
2652	VE1111	Miranda	Sucre	Leoncio Martínez	*P. geniculatus*	2011	Female	Positive	No
2653	VE47211	Miranda	Sucre	Leoncio Martínez	*P. geniculatus*	2011	Male	Positive	No
2654	VE50011	Miranda	Sucre	Leoncio Martínez	*P. geniculatus*	2011	Male	Positive	No
2655	VE202B11	Miranda	Sucre	Leoncio Martínez	*P. geniculatus*	2011	Female	Positive	No
2656	VE230B11	Miranda	Sucre	Leoncio Martínez	*P. geniculatus*	2011	Male	Positive	No
2657	VE29811	Miranda	Sucre	Leoncio Martínez	*P. geniculatus*	2011	Female	Positive	No
2658	VE24411	Miranda	Sucre	Leoncio Martínez	*P. geniculatus*	2011	Female	Positive	No
2659	VE40411	Miranda	Sucre	Leoncio Martínez	*P. geniculatus*	2011	Male	Positive	No
2660	VE6711	Miranda	sucre	Leoncio Martínez	*P. geniculatus*	2011	Male	Positive	No
2661	VE15511	Miranda	sucre	Leoncio Martínez	*P. geniculatus*	2011	Male	Positive	No
2662	VE17211	Miranda	Sucre	Leoncio Martínez	*P. geniculatus*	2011	Female	Positive	No
2663	VE29211A	Miranda	Sucre	Leoncio Martínez	*P. geniculatus*	2011	Male	Positive	No
2664	VE29211B	Miranda	Sucre	Leoncio Martínez	*P. geniculatus*	2011	Female	Positive	No
2665	VE36111	Miranda	Sucre	Leoncio Martínez	*P. geniculatus*	2011	Female	Positive	No
2666	VE26511	Miranda	Sucre	Leoncio Martínez	*P. geniculatus*	2011	Male	Positive	No
2667	VE18111	Miranda	Sucre	Leoncio Martínez	*P. geniculatus*	2011	Female	Positive	No
2668	VE55011	Miranda	Sucre	Leoncio Martínez	*P. geniculatus*	2011	Female	Positive	No
2669	VE27611	Miranda	Sucre	Leoncio Martínez	*P. geniculatus*	2011	Male	Positive	Yes
2670	VE36911	Miranda	Sucre	Leoncio Martínez	*P. geniculatus*	2011	Female	Positive	Yes
2671	VE202A11	Miranda	Sucre	Leoncio Martínez	*P. geniculatus*	2011	Female	Positive	Yes
2672	VE230A11	Miranda	Sucre	Leoncio Martínez	*P. geniculatus*	2011	Female	Positive	Yes
2673	VE42511	Miranda	Sucre	Leoncio Martínez	*P. geniculatus*	2011	Female	Positive	Yes
2674	VE25511	Miranda	Sucre	Leoncio Martínez	*P. geniculatus*	2011	Female	Positive	Yes
2675	VE39211	Miranda	Baruta	Nuestra Señora del Rosario de Baruta	*P. geniculatus*	2011	Female	NA	NA
2676	VE48811	Miranda	Baruta	Nuestra Señora del Rosario de Baruta	*P. geniculatus*	2011	Male	NA	NA
2677	VE52011	Miranda	Baruta	Nuestra Señora del Rosario de Baruta	*P. geniculatus*	2011	Female	NA	NA
2678	VE50711	Miranda	Baruta	Nuestra Señora del Rosario de Baruta	*P. geniculatus*	2011	Male	NA	NA
2679	VE13211	Miranda	Baruta	Nuestra Señora del Rosario de Baruta	*P. geniculatus*	2011	Male	NA	NA
2680	VE27411A	Miranda	Baruta	Nuestra Señora del Rosario de Baruta	*P. geniculatus*	2011	Male	NA	NA
2681	VE27411	Miranda	Baruta	Nuestra Señora del Rosario de Baruta	*P. geniculatus*	2011	Male	NA	NA
2682	VE0111A	Miranda	Baruta	Nuestra Señora del Rosario de Baruta	*P. geniculatus*	2011	Female	NA	NA
2683	VE0111B	Miranda	Baruta	Nuestra Señora del Rosario de Baruta	*P. geniculatus*	2011	Male	NA	NA
2684	VE0411B	Miranda	Baruta	Nuestra Señora del Rosario de Baruta	*P. geniculatus*	2011	Female	NA	NA
2685	VE33811	Miranda	Baruta	Nuestra Señora del Rosario de Baruta	*P. geniculatus*	2011	Male	NA	No
2686	VE2611	Miranda	Baruta	Nuestra Señora del Rosario de Baruta	*P. geniculatus*	2011	Female	NA	Yes

2687	VE43811	Miranda	Baruta	Nuestra Señora del Rosario de Baruta	*P. geniculatus*	2011	Female	NA	Yes
2688	VE22411	Miranda	Baruta	Nuestra Señora del Rosario de Baruta	*P. geniculatus*	2011	Female	Negative	No
2689	VE40611	Miranda	Baruta	Nuestra Señora del Rosario de Baruta	*P. geniculatus*	2011	Male	Negative	No
2690	VE46211	Miranda	Baruta	Nuestra Señora del Rosario de Baruta	*P. geniculatus*	2011	Male	Negative	No
2691	VE39311	Miranda	Baruta	Nuestra Señora del Rosario de Baruta	*P. geniculatus*	2011	Male	Positive	No
2692	VE41311	Miranda	Baruta	Nuestra Señora del Rosario de Baruta	*P. geniculatus*	2011	Male	Positive	No
2693	VE50411	Miranda	Baruta	Nuestra Señora del Rosario de Baruta	*P. geniculatus*	2011	Male	Positive	No
2694	VE3911	Miranda	Baruta	Nuestra Señora del Rosario de Baruta	*P. geniculatus*	2011	Female	Positive	No
2695	VE12211	Miranda	Baruta	Nuestra Señora del Rosario de Baruta	*P. geniculatus*	2011	Female	Positive	No
2696	VE19211	Miranda	Baruta	Nuestra Señora del Rosario de Baruta	*P. geniculatus*	2011	Female	Positive	No
2697	VE14211	Miranda	baruta	Nuestra Señora del Rosario de Baruta	*P. geniculatus*	2011	Female	Positive	No
2698	VE3111A	Miranda	Baruta	Nuestra Señora del Rosario de Baruta	*P. geniculatus*	2011	Female	Positive	No
2699	VE6911	Miranda	Baruta	Nuestra Señora del Rosario de Baruta	*P. geniculatus*	2011	Male	Positive	No
2700	VE19011	Miranda	Baruta	Nuestra Señora del Rosario de Baruta	*P. geniculatus*	2011	Female	Positive	No
2701	VE20811	Miranda	Baruta	Nuestra Señora del Rosario de Baruta	*P. geniculatus*	2011	Female	Positive	No
2702	VE28811	Miranda	Baruta	Nuestra Señora del Rosario de Baruta	*P. geniculatus*	2011	Male	Positive	No
2703	VE31511	Miranda	Baruta	Nuestra Señora del Rosario de Baruta	*P. geniculatus*	2011	Female	Positive	No
2704	VE19711	Miranda	Baruta	Nuestra Señora del Rosario de Baruta	*P. geniculatus*	2011	Female	Positive	No
2705	VE3111B	Miranda	Baruta	Nuestra Señora del Rosario de Baruta	*P. geniculatus*	2011	Female	Positive	No
2706	VE25911	Miranda	Baruta	Nuestra Señora del Rosario de Baruta	*P. geniculatus*	2011	Female	Positive	No
2707	VE1711	Miranda	Baruta	Nuestra Señora del Rosario de Baruta	*P. geniculatus*	2011	Male	Positive	No
2708	VE29911	Miranda	Baruta	Nuestra Señora del Rosario de Baruta	*P. geniculatus*	2011	Female	Positive	Yes
2709	VE4011	Miranda	Baruta	Nuestra Señora del Rosario de Baruta	*P. geniculatus*	2011	Male	Positive	Yes
2710	VE17911	Miranda	Baruta	Nuestra Señora del Rosario de Baruta	*P. geniculatus*	2011	Female	Positive	Yes
2711	VE26211	Miranda	Baruta	Nuestra Señora del Rosario de Baruta	*P. geniculatus*	2011	Female	Positive	Yes
2712	VE8111	Miranda	Baruta	Nuestra Señora del Rosario de Baruta	*P. geniculatus*	2011	Female	Positive	Yes
2713	VE3211	Miranda	Baruta	Nuestra Señora del Rosario de Baruta	*P. geniculatus*	2011	Female	Positive	Yes
2714	VE18211	Miranda	Baruta	Nuestra Señora del Rosario de Baruta	*P. geniculatus*	2011	Female	Positive	Yes
2715	VE33111	Miranda	Baruta	Nuestra Señora del Rosario de Baruta	*P. geniculatus*	2011	Female	Positive	Yes
2716	VE32711	Miranda	Baruta	Nuestra Señora del Rosario de Baruta	*P. geniculatus*	2011	Male	Positive	Yes
2717	VE8311A	Miranda	Baruta	Nuestra Señora del Rosario de Baruta	*P. geniculatus*	2011	Female	Positive	Yes
2718	VE8311B	Miranda	Baruta	Nuestra Señora del Rosario de Baruta	*P. geniculatus*	2011	Female	Positive	Yes
2719	VE0411A	Miranda	Baruta	Nuestra Señora del Rosario de Baruta	*P. geniculatus*	2011	Female	Positive	Yes
2720	VE38511	Miranda	Baruta	Nuestra Señora del Rosario de Baruta	*P. geniculatus*	2011	Female	Positive	Yes
2721	VE30911	Miranda	Baruta	Nuestra Señora del Rosario de Baruta	*P. geniculatus*	2011	Female	Positive	Yes
2722	VE227B11	Miranda	Sucre	Petare	*P. geniculatus*	2011	Male	NA	NA
2723	VE41111	Miranda	Sucre	Petare	*P. geniculatus*	2011	Nymph IV	NA	NA
2724	VE23111A	Miranda	Sucre	Petare	*P. geniculatus*	2011	Male	NA	NA
2725	VE37711	Miranda	Sucre	Petare	*P. geniculatus*	2011	Female	NA	NA
2726	VE41211	Miranda	Sucre	Petare	*P. geniculatus*	2011	Female	NA	NA
2727	VE19111	Miranda	Sucre	Petare	*P. geniculatus*	2011	Female	NA	NA
2728	VE225B11	Miranda	Sucre	Petare	*P. geniculatus*	2011	Male	NA	NA

2729	VE2411A	Miranda	Sucre	Petare	*P. geniculatus*	2011	Female	NA	NA
2730	VE2411B	Miranda	Sucre	Petare	*P. geniculatus*	2011	Female	NA	NA
2731	VE2411D	Miranda	Sucre	Petare	*P. geniculatus*	2011	Nymph V	NA	NA
2732	VE20311	Miranda	Sucre	Petare	*P. geniculatus*	2011	Female	NA	NA
2733	VE31611	Miranda	Sucre	Petare	*P. geniculatus*	2011	Male	NA	NA
2734	VE23111B	Miranda	Sucre	Petare	*P. geniculatus*	2011	Male	NA	No
2735	VE12011	Miranda	Sucre	Petare	*P. geniculatus*	2011	Female	NA	Yes
2736	VE36011	Miranda	Sucre	Petare	*P. geniculatus*	2011	Female	NA	Yes
2737	VE31111	Miranda	Sucre	Petare	*P. geniculatus*	2011	Female	Negative	No
2738	VE34511	Miranda	Sucre	Petare	*P. geniculatus*	2011	Male	Negative	No
2739	VE54311	Miranda	Sucre	Petare	*P. geniculatus*	2011	Male	Negative	No
2740	VE0511	Miranda	Sucre	Petare	*P. geniculatus*	2011	Male	Negative	No
2741	VE54711	Miranda	Sucre	Petare	*P. geniculatus*	2011	Female	Negative	Yes
2742	VE6811	Miranda	Sucre	Petare	*P. geniculatus*	2011	Male	Negative	Yes
2743	VE8611	Miranda	Sucre	Petare	*P. geniculatus*	2011	Female	Negative	Yes
2744	VE41511	Miranda	Sucre	Petare	*P. geniculatus*	2011	Female	Negative	Yes
2745	VE2711	Miranda	Sucre	Petare	*P. geniculatus*	2011	Male	Positive	No
2746	VE1011	Miranda	Sucre	Petare	*P. geniculatus*	2011	Female	Positive	No
2747	VE55311	Miranda	Sucre	Petare	*P. geniculatus*	2011	Male	Positive	No
2748	VE1411A	Miranda	Sucre	Petare	*P. geniculatus*	2011	Male	Positive	No
2749	VE1411B	Miranda	Sucre	Petare	*P. geniculatus*	2011	Male	Positive	No
2750	VE48211	Miranda	Sucre	Petare	*P. geniculatus*	2011	Female	Positive	No
2751	VE250A11	Miranda	Sucre	Petare	*P. geniculatus*	2011	Male	Positive	No
2752	VE250B11	Miranda	Sucre	Petare	*P. geniculatus*	2011	Male	Positive	No
2753	VE250C11	Miranda	Sucre	Petare	*P. geniculatus*	2011	Male	Positive	No
2754	VE250D11	Miranda	Sucre	Petare	*P. geniculatus*	2011	Male	Positive	No
2755	VE250E11	Miranda	Sucre	Petare	*P. geniculatus*	2011	Male	Positive	No
2756	VE148B11	Miranda	Sucre	Petare	*P. geniculatus*	2011	Female	Positive	No
2757	VE148C11	Miranda	Sucre	Petare	*P. geniculatus*	2011	Male	Positive	No
2758	VE40011	Miranda	Sucre	Petare	*P. geniculatus*	2011	Male	Positive	No
2759	VE30511	Miranda	Sucre	Petare	*P. geniculatus*	2011	Male	Positive	No
2760	VE23411	Miranda	Sucre	Petare	*P. geniculatus*	2011	Male	Positive	No
2761	VE4111	Miranda	Sucre	Petare	*P. geniculatus*	2011	Male	Positive	No
2762	VE4411	Miranda	Sucre	Petare	*P. geniculatus*	2011	Male	Positive	No
2763	VE6111	Miranda	Sucre	Petare	*P. geniculatus*	2011	Male	Positive	No
2764	VE76A11	Miranda	Sucre	Petare	*P. geniculatus*	2011	Male	Positive	No
2765	VE76B11	Miranda	Sucre	Petare	*P. geniculatus*	2011	Male	Positive	No
2766	VE10111	Miranda	Sucre	Petare	*P. geniculatus*	2011	Male	Positive	No
2767	VE10211	Miranda	Sucre	Petare	*P. geniculatus*	2011	Male	Positive	No
2768	VE14611	Miranda	Sucre	Petare	*P. geniculatus*	2011	Male	Positive	No
2769	VE15211	Miranda	Sucre	Petare	*P. geniculatus*	2011	Female	Positive	No
2770	VE183B11	Miranda	Sucre	Petare	*P. geniculatus*	2011	Female	Positive	No

2771	VE41911	Miranda	Sucre	Petare	*P. geniculatus*	2011	Female	Positive	No
2772	VE43711	Miranda	Sucre	Petare	*P. geniculatus*	2011	Male	Positive	No
2773	VE22311	Miranda	sucre	Petare	*P. geniculatus*	2011	Female	Positive	No
2774	VE225A11	Miranda	Sucre	Petare	*P. geniculatus*	2011	Male	Positive	No
2775	VE5111	Miranda	Sucre	Petare	*P. geniculatus*	2011	Female	Positive	No
2776	VE6311	Miranda	Sucre	Petare	*P. geniculatus*	2011	Female	Positive	No
2777	VE180B11	Miranda	Sucre	Petare	*P. geniculatus*	2011	Female	Positive	No
2778	VE23211	Miranda	Sucre	Petare	*P. geniculatus*	2011	Male	Positive	No
2779	VE23611	Miranda	Sucre	Petare	*P. geniculatus*	2011	Male	Positive	No
2780	VE13411	Miranda	Sucre	Petare	*P. geniculatus*	2011	Female	Positive	No
2781	VE1911	Miranda	Sucre	Petare	*P. geniculatus*	2011	Male	Positive	No
2782	VE53511B	Miranda	Sucre	Petare	*P. geniculatus*	2011	Female	Positive	No
2783	VE51411B	Miranda	Sucre	Petare	*P. geniculatus*	2011	Male	Positive	No
2784	VE34911	Miranda	Sucre	Petare	*P. geniculatus*	2011	Female	Positive	No
2785	VE21811	Miranda	Sucre	Petare	*P. geniculatus*	2011	Female	Positive	Yes
2786	VE3411	Miranda	Sucre	Petare	*P. geniculatus*	2011	Male	Positive	Yes
2787	VE16711	Miranda	Sucre	Petare	*P. geniculatus*	2011	Female	Positive	Yes
2788	VE227A11	Miranda	Sucre	Petare	*P. geniculatus*	2011	Male	Positive	Yes
2789	VE46511	Miranda	Sucre	Petare	*P. geniculatus*	2011	Female	Positive	Yes
2790	VE250F11	Miranda	Sucre	Petare	*P. geniculatus*	2011	Male	Positive	Yes
2791	VE250G11	Miranda	Sucre	Petare	*P. geniculatus*	2011	Male	Positive	Yes
2792	VE250H11	Miranda	Sucre	Petare	*P. geniculatus*	2011	Male	Positive	Yes
2793	VE148A11	Miranda	Sucre	Petare	*P. geniculatus*	2011	Female	Positive	Yes
2794	VE30711	Miranda	Sucre	Petare	*P. geniculatus*	2011	Female	Positive	Yes
2795	VE40211	Miranda	Sucre	Petare	*P. geniculatus*	2011	Female	Positive	Yes
2796	VE9611	Miranda	Sucre	Petare	*P. geniculatus*	2011	Female	Positive	Yes
2797	VE9911	Miranda	Sucre	Petare	*P. geniculatus*	2011	Female	Positive	Yes
2798	VE117A11	Miranda	Sucre	Petare	*P. geniculatus*	2011	Female	Positive	Yes
2799	VE117B11	Miranda	Sucre	Petare	*P. geniculatus*	2011	Female	Positive	Yes
2800	VE117C11	Miranda	Sucre	Petare	*P. geniculatus*	2011	Female	Positive	Yes
2801	VE15611	Miranda	Sucre	Petare	*P. geniculatus*	2011	Male	Positive	Yes
2802	VE16111	Miranda	Sucre	Petare	*P. geniculatus*	2011	Female	Positive	Yes
2803	VE183A11	Miranda	Sucre	Petare	*P. geniculatus*	2011	Female	Positive	Yes
2804	VE21011	Miranda	Sucre	Petare	*P. geniculatus*	2011	Female	Positive	Yes
2805	VE21311	Miranda	Sucre	Petare	*P. geniculatus*	2011	Female	Positive	Yes
2806	VE21711	Miranda	Sucre	Petare	*P. geniculatus*	2011	Female	Positive	Yes
2807	VE34311	Miranda	Sucre	Petare	*P. geniculatus*	2011	Male	Positive	Yes
2808	VE44011	Miranda	Sucre	Petare	*P. geniculatus*	2011	Female	Positive	Yes
2809	VE50311	Miranda	Sucre	Petare	*P. geniculatus*	2011	Female	Positive	Yes
2810	VE53911	Miranda	Sucre	Petare	*P. geniculatus*	2011	Female	Positive	Yes
2811	VE18911	Miranda	Sucre	Petare	*P. geniculatus*	2011	Female	Positive	Yes
2812	VE0811	Miranda	Sucre	Petare	*P. geniculatus*	2011	Male	Positive	Yes

2813	VE2411C	Miranda	Sucre	Petare	*P. geniculatus*	2011	Nymph V	Positive	Yes
2814	VE47011	Miranda	Sucre	Petare	*P. geniculatus*	2011	Female	Positive	Yes
2815	VE180A11	Miranda	Sucre	Petare	*P. geniculatus*	2011	Female	Positive	Yes
2816	VE0311	Miranda	Sucre	Petare	*P. geniculatus*	2011	Female	Positive	Yes
2817	VE44911	Miranda	Sucre	Petare	*P. geniculatus*	2011	Male	Positive	Yes
2818	VE34211	Miranda	Sucre	Petare	*P. geniculatus*	2011	Female	Positive	Yes
2819	VE17711	Miranda	Sucre	Petare	*P. geniculatus*	2011	Female	Positive	Yes
2820	VE30811	Miranda	Sucre	Petare	*P. geniculatus*	2011	Female	Positive	Yes
2821	VE33011	Miranda	Sucre	Petare	*P. geniculatus*	2011	Male	Positive	Yes
2822	VE54111	Miranda	Sucre	Petare	*P. geniculatus*	2011	Female	Positive	Yes
2823	VE21111	Miranda	Sucre	Petare	*P. geniculatus*	2011	Male	Positive	Yes
2824	VE51511	Miranda	Sucre	Petare	*P. geniculatus*	2011	Male	Positive	Yes
2825	VE24711	Miranda	El Hatillo	Santa Rosalía de Palermo	*P. geniculatus*	2011	Female	NA	NA
2826	VE26711	Miranda	El Hatillo	Santa Rosalía de Palermo	*P. geniculatus*	2011	Male	NA	Yes
2827	VE6611	Miranda	El Hatillo	Santa Rosalía de Palermo	*P. geniculatus*	2011	Female	Negative	No
2828	VE19511	Miranda	El Hatillo	Santa Rosalía de Palermo	*P. geniculatus*	2011	Female	Negative	No
2829	VE13311	Miranda	El Hatillo	Santa Rosalía de Palermo	*T. Maculata*	2011	Male	Negative	Yes
2830	VE25811	Miranda	El Hatillo	Santa Rosalía de Palermo	*T. nigromaculata*	2011	Male	Negative	Yes
2831	VE38411	Miranda	El Hatillo	Santa Rosalía de Palermo	*P. geniculatus*	2011	Female	Negative	Yes
2832	VE53711	Miranda	El Hatillo	Santa Rosalía de Palermo	*P. geniculatus*	2011	Nymph V	Negative	Yes
2833	VE6511	Miranda	El Hatillo	Santa Rosalía de Palermo	*P. geniculatus*	2011	Female	Negative	Yes
2834	VE22011	Miranda	El Hatillo	Santa Rosalía de Palermo	*P. geniculatus*	2011	Female	Negative	Yes
2835	VE7911	Miranda	El Hatillo	Santa Rosalía de Palermo	*P. geniculatus*	2011	Male	Positive	No
2836	VE43211	Miranda	El Hatillo	Santa Rosalía de Palermo	*P. geniculatus*	2011	Male	Positive	No
2837	VE53211	Miranda	El Hatillo	Santa Rosalía de Palermo	*P. geniculatus*	2011	Male	Positive	No
2838	VE13111	Miranda	El Hatillo	Santa Rosalía de Palermo	*P. geniculatus*	2011	Female	Positive	No
2839	VE19611	Miranda	El Hatillo	Santa Rosalía de Palermo	*P. geniculatus*	2011	Female	Positive	No
2840	VE44611	Miranda	El Hatillo	Santa Rosalía de Palermo	*P. geniculatus*	2011	Female	Positive	No
2841	VE7411	Miranda	El Hatillo	Santa Rosalía de Palermo	*P. geniculatus*	2011	Male	Positive	Yes
2842	VE17511	Miranda	El Hatillo	Santa Rosalía de Palermo	*P. geniculatus*	2011	Female	Positive	Yes
2843	VE29711	Miranda	El Hatillo	Santa Rosalía de Palermo	*P. geniculatus*	2011	Female	Positive	Yes
2844	VE30211	Miranda	El Hatillo	Santa Rosalía de Palermo	*P. geniculatus*	2011	Female	Positive	Yes
2845	VE33311	Miranda	El Hatillo	Santa Rosalía de Palermo	*P. geniculatus*	2011	Female	Positive	Yes
2846	VE54211	Miranda	El Hatillo	Santa Rosalía de Palermo	*P. geniculatus*	2011	Female	Positive	Yes
2847	VE14812	DC	Libertador	Altagracia	*P. geniculatus*	2012	Female	Positive	Yes
2848	VE31712	DC	Libertador	Altagracia	*P. geniculatus*	2012	Female	Positive	No
2849	VE13712	DC	Libertador	Antímano	*P. geniculatus*	2012	Male	Negative	No
2850	VE25112A	DC	Libertador	Antímano	*P. geniculatus*	2012	Female	Positive	Yes
2851	VE29812	DC	Libertador	Antímano	*P. geniculatus*	2012	Female	Positive	Yes
2852	VE30612	DC	Libertador	Antímano	*P. geniculatus*	2012	Female	Positive	No
2853	VE3912	DC	Libertador	Antímano	*P. geniculatus*	2012	Female	NA	Yes
2854	VE39912	DC	Libertador	Antímano	*P. geniculatus*	2012	Female	Positive	No

2855	VE4312	DC	Libertador	Antímano	*P. geniculatus*	2012	Female	Positive	Yes
2856	VE8712	DC	Libertador	Antímano	*P. geniculatus*	2012	Female	Positive	Yes
2857	VE9812	DC	Libertador	Antímano	*P. geniculatus*	2012	Female	Positive	Yes
2858	VE10612	DC	Libertador	Candelaria	*P. geniculatus*	2012	Female	NA	No
2859	VE15212	DC	Libertador	Caricuao	*P. geniculatus*	2012	Male	Negative	No
2860	VE24212	DC	Libertador	Caricuao	*P. geniculatus*	2012	Male	NA	No
2861	VE29312	DC	Libertador	Caricuao	*P. geniculatus*	2012	Female	Positive	Yes
2862	VE32212	DC	Libertador	Caricuao	*P. geniculatus*	2012	Female	Positive	No
2863	VE6112	DC	Libertador	Caricuao	*P. geniculatus*	2012	Female	NA	NA
2864	VE10012	DC	Libertador	El Junquito	*P. geniculatus*	2012	Female	NA	No
2865	VE19512	DC	Libertador	El Junquito	*P. geniculatus*	2012	Male	NA	NA
2866	VE20012	DC	Libertador	El Junquito	*P. geniculatus*	2012	Female	Positive	No
2867	VE21412A	DC	Libertador	El Junquito	*P. geniculatus*	2012	Male	Positive	No
2868	VE21412B	DC	Libertador	El Junquito	*P. geniculatus*	2012	Female	Positive	No
2869	VE21512	DC	Libertador	El Junquito	*P. geniculatus*	2012	Male	Negative	No
2870	VE24812	DC	Libertador	El Junquito	*P. geniculatus*	2012	Male	Positive	No
2871	VE27412	DC	Libertador	El Junquito	*P. geniculatus*	2012	Female	NA	No
2872	VE36712	DC	Libertador	El Junquito	*P. geniculatus*	2012	Male	Positive	Yes
2873	VE7212	DC	Libertador	El Junquito	*P. geniculatus*	2012	Male	Positive	Yes
2874	VE8112	DC	Libertador	El Junquito	*P. geniculatus*	2012	Female	NA	NA
2875	VE18612	DC	Libertador	El Recreo	*P. geniculatus*	2012	Male	NA	NA
2876	VE18712A	DC	Libertador	El Recreo	*P. geniculatus*	2012	Male	Positive	No
2877	VE18712B	DC	Libertador	El Recreo	*P. geniculatus*	2012	Male	NA	NA
2878	VE28212	DC	Libertador	El Recreo	*P. geniculatus*	2012	Female	NA	Yes
2879	VE3612A	DC	Libertador	El Recreo	*P. geniculatus*	2012	Male	Negative	No
2880	VE38612	DC	Libertador	El Recreo	*P. geniculatus*	2012	Female	Positive	Yes
2881	VE0812B	DC	Libertador	La Pastora	*P. geniculatus*	2012	Female	Positive	Yes
2882	VE12812	DC	Libertador	La Pastora	*P. geniculatus*	2012	Male	Positive	Yes
2883	VE14012	DC	Libertador	La Pastora	*P. geniculatus*	2012	Female	Positive	Yes
2884	VE14112	DC	Libertador	La Pastora	*P. geniculatus*	2012	Male	Negative	No
2885	VE22912	DC	Libertador	La Pastora	*P. geniculatus*	2012	Male	Positive	Yes
2886	VE24712A	DC	Libertador	La Pastora	*P. geniculatus*	2012	Male	Positive	Yes
2887	VE24712B	DC	Libertador	La Pastora	*P. geniculatus*	2012	Male	Positive	Yes
2888	VE25312	DC	Libertador	La Pastora	*P. geniculatus*	2012	Female	Positive	Yes
2889	VE26812	DC	Libertador	La Pastora	*P. geniculatus*	2012	Female	Positive	Yes
2890	VE29012	DC	Libertador	La Pastora	*P. geniculatus*	2012	Male	Positive	No
2891	VE30012	DC	Libertador	La Pastora	*P. geniculatus*	2012	Female	NA	No
2892	VE33412A	DC	Libertador	La Pastora	*P. geniculatus*	2012	Male	Negative	No
2893	VE33412B	DC	Libertador	La Pastora	*P. geniculatus*	2012	Male	Negative	Yes
2894	VE34112	DC	Libertador	La Pastora	*P. geniculatus*	2012	Male	Negative	No
2895	VE37712	DC	Libertador	La Pastora	*P. geniculatus*	2012	Female	Negative	No
2896	VE8912	DC	Libertador	La Pastora	*P. geniculatus*	2012	Female	Positive	No

2897	VE9312	DC	Libertador	La Pastora	*P. geniculatus*	2012	Male	NA	Yes
2898	VE10712A	DC	Libertador	La Vega	*P. geniculatus*	2012	Female	Positive	Yes
2899	VE10712B	DC	Libertador	La Vega	*P. geniculatus*	2012	Female	Positive	Yes
2900	VE14912	DC	Libertador	La Vega	*P. geniculatus*	2012	Nymph V	NA	NA
2901	VE1812	DC	Libertador	La Vega	*P. geniculatus*	2012	Male	Positive	No
2902	VE20412	DC	Libertador	La Vega	*P. geniculatus*	2012	Female	NA	Yes
2903	VE20712	DC	Libertador	La Vega	*P. geniculatus*	2012	Female	Positive	No
2904	VE31312A	DC	Libertador	La Vega	*P. geniculatus*	2012	Male	Positive	No
2905	VE31312B	DC	Libertador	La Vega	*P. geniculatus*	2012	Nymph IV	Positive	No
2906	VE34212A	DC	Libertador	La Vega	*P. geniculatus*	2012	Female	NA	Yes
2907	VE34212B	DC	Libertador	La Vega	*P. geniculatus*	2012	Female	Positive	Yes
2908	VE38112	DC	Libertador	La Vega	*P. geniculatus*	2012	Female	Positive	Yes
2909	VE37612	DC	Libertador	Macarao	*P. geniculatus*	2012	Female	Positive	Yes
2910	VE26112	DC	Libertador	San Agustín	*P. geniculatus*	2012	Male	Positive	No
2911	VE19012	DC	Libertador	San Bernardino	*P. geniculatus*	2012	Female	Positive	No
2912	VE22512	DC	Libertador	San José	*P. geniculatus*	2012	Female	Positive	No
2913	VE2412	DC	Libertador	San José	*P. geniculatus*	2012	Female	NA	No
2914	VE9712	DC	Libertador	San José	*P. geniculatus*	2012	Female	NA	No
2915	VE0212	DC	Libertador	San Juan	*P. geniculatus*	2012	Male	Negative	No
2916	VE21712	DC	Libertador	San Juan	*P. geniculatus*	2012	Male	Positive	No
2917	VE2712	DC	Libertador	San Pedro	*P. geniculatus*	2012	Male	Positive	No
2918	VE31012	DC	Libertador	San Pedro	*P. geniculatus*	2012	Female	NA	No
2919	VE34412	DC	Libertador	San Pedro	*P. geniculatus*	2012	Female	Negative	Yes
2920	VE7012	DC	Libertador	San Pedro	*P. geniculatus*	2012	Female	Positive	Yes
2921	VE0412	DC	Libertador	Sucre	*P. geniculatus*	2012	Female	Positive	Yes
2922	VE10812	DC	Libertador	Sucre	*P. geniculatus*	2012	Male	NA	Yes
2923	VE11012	DC	Libertador	Sucre	*P. geniculatus*	2012	Female	Negative	Yes
2924	VE11112	DC	Libertador	Sucre	*P. geniculatus*	2012	Female	Negative	No
2925	VE1112	DC	Libertador	Sucre	*P. geniculatus*	2012	Male	Positive	No
2926	VE13012	DC	Libertador	Sucre	*P. geniculatus*	2012	Female	Positive	No
2927	VE15412A	DC	Libertador	Sucre	*P. geniculatus*	2012	Male	Negative	No
2928	VE15412B	DC	Libertador	Sucre	*P. geniculatus*	2012	Male	Positive	No
2929	VE15512	DC	Libertador	Sucre	*P. geniculatus*	2012	Female	Positive	Yes
2930	VE16012	DC	Libertador	Sucre	*P. geniculatus*	2012	Male	Positive	No
2931	VE16112	DC	Libertador	Sucre	*P. geniculatus*	2012	Female	Positive	Yes
2932	VE18812	DC	Libertador	Sucre	*P. geniculatus*	2012	Female	Positive	No
2933	VE19112	DC	Libertador	Sucre	*P. geniculatus*	2012	Nymph IV	Negative	Yes
2934	VE19212	DC	Libertador	Sucre	*P. geniculatus*	2012	Male	Positive	No
2935	VE19912	DC	Libertador	Sucre	*P. geniculatus*	2012	Male	Positive	No
2936	VE2012	DC	Libertador	Sucre	*P. geniculatus*	2012	Nymph IV	NA	No
2937	VE23112	DC	Libertador	Sucre	*P. geniculatus*	2012	Female	Positive	Yes
2938	VE23212B	DC	Libertador	Sucre	*P. geniculatus*	2012	Male	NA	No

2939	VE23312	DC	Libertador	Sucre	*P. geniculatus*	2012	Female	NA	No
2940	VE26012	DC	Libertador	Sucre	*P. geniculatus*	2012	Female	Negative	Yes
2941	VE27912	DC	Libertador	Sucre	*P. geniculatus*	2012	Female	Positive	Yes
2942	VE28112	DC	Libertador	Sucre	*P. geniculatus*	2012	Female	NA	NA
2943	VE28312	DC	Libertador	Sucre	*P. geniculatus*	2012	Female	Positive	Yes
2944	VE29912	DC	Libertador	Sucre	*P. geniculatus*	2012	Female	Positive	No
2945	VE30312	DC	Libertador	Sucre	*P. geniculatus*	2012	Female	Positive	Yes
2946	VE30812	DC	Libertador	Sucre	*P. geniculatus*	2012	Male	Positive	Yes
2947	VE31412	DC	Libertador	Sucre	*P. geniculatus*	2012	Female	Positive	Yes
2948	VE31512	DC	Libertador	Sucre	*P. geniculatus*	2012	Female	Negative	No
2949	VE31612	DC	Libertador	Sucre	*P. geniculatus*	2012	Nymph IV	Negative	Yes
2950	VE32112	DC	Libertador	Sucre	*P. geniculatus*	2012	Nymph IV	Positive	Yes
2951	VE32812	DC	Libertador	Sucre	*P. geniculatus*	2012	Female	Positive	Yes
2952	VE33512	DC	Libertador	Sucre	*P. geniculatus*	2012	Male	NA	NA
2953	VE33812	DC	Libertador	Sucre	*P. geniculatus*	2012	Female	Positive	Yes
2954	VE34512A	DC	Libertador	Sucre	*P. geniculatus*	2012	Female	NA	NA
2955	VE34512B	DC	Libertador	Sucre	*P. geniculatus*	2012	Female	NA	NA
2956	VE34512C	DC	Libertador	Sucre	*P. geniculatus*	2012	Female	NA	NA
2957	VE34512D	DC	Libertador	Sucre	*P. geniculatus*	2012	Male	NA	NA
2958	VE34512E	DC	Libertador	Sucre	*P. geniculatus*	2012	Male	NA	NA
2959	VE3512	DC	Libertador	Sucre	*P. geniculatus*	2012	Female	Positive	Yes
2960	VE36212	DC	Libertador	Sucre	*P. geniculatus*	2012	Male	Positive	No
2961	VE36312	DC	Libertador	Sucre	*P. geniculatus*	2012	Female	NA	NA
2962	VE37012	DC	Libertador	Sucre	*P. geniculatus*	2012	Male	Positive	Yes
2963	VE37812	DC	Libertador	Sucre	*P. geniculatus*	2012	Male	NA	NA
2964	VE38012	DC	Libertador	Sucre	*P. geniculatus*	2012	Female	Positive	Yes
2965	VE3812	DC	Libertador	Sucre	*P. geniculatus*	2012	Nymph III	NA	NA
2966	VE38212	DC	Libertador	Sucre	*P. geniculatus*	2012	Female	Positive	Yes
2967	VE39012	DC	Libertador	Sucre	*P. geniculatus*	2012	Female	NA	NA
2968	VE39812	DC	Libertador	Sucre	*P. geniculatus*	2012	Male	Positive	No
2969	VE4512	DC	Libertador	Sucre	*P. geniculatus*	2012	Male	Positive	Yes
2970	VE6412	DC	Libertador	Sucre	*P. geniculatus*	2012	Female	Positive	Yes
2971	VE6812	DC	Libertador	Sucre	*P. geniculatus*	2012	Female	Positive	No
2972	VE6912	DC	Libertador	Sucre	*P. geniculatus*	2012	Male	Positive	No
2973	VE7612	DC	Libertador	Sucre	*P. geniculatus*	2012	Male	Positive	Yes
2974	VE7912	DC	Libertador	Sucre	*P. geniculatus*	2012	Female	Positive	Yes
2975	VE9612	DC	Libertador	Sucre	*P. geniculatus*	2012	Male	Negative	No
2976	VE11612	Miranda	Sucre	Caucagüita	*P. geniculatus*	2012	Female	Positive	Yes
2977	VE12012	Miranda	Sucre	Caucagüita	*P. geniculatus*	2012	Male	Negative	No
2978	VE37212	Miranda	Sucre	Caucagüita	*P. geniculatus*	2012	Male	Negative	No
2979	VE4612	Miranda	Sucre	Caucagüita	*P. geniculatus*	2012	Female	Negative	No
2980	VE15712	Miranda	Chacao	Chacao	*P. geniculatus*	2012	Female	Negative	Yes

2981	VE28712	Miranda	Chacao	Chacao	*P. geniculatus*	2012	Nymph IV	Negative	Yes
2982	VE35912	Miranda	Chacao	Chacao	*P. geniculatus*	2012	Male	NA	NA
2983	VE14712	Miranda	Baruta	El Cafetal	*P. geniculatus*	2012	Male	Positive	No
2984	VE17812	Miranda	Baruta	El Cafetal	*P. geniculatus*	2012	Female	Positive	No
2985	VE18512	Miranda	Baruta	El Cafetal	*P. geniculatus*	2012	Male	Negative	No
2986	VE19412	Miranda	Baruta	El Cafetal	*P. geniculatus*	2012	Female	Positive	No
2987	VE25912	Miranda	Baruta	El Cafetal	*P. geniculatus*	2012	Female	NA	NA
2988	VE28812	Miranda	Baruta	El Cafetal	*P. geniculatus*	2012	Female	Positive	Yes
2989	VE30112	Miranda	Baruta	El Cafetal	*P. geniculatus*	2012	Female	Positive	Yes
2990	VE33712	Miranda	Baruta	El Cafetal	*P. geniculatus*	2012	Male	NA	NA
2991	VE6512	Miranda	Baruta	El Cafetal	*P. geniculatus*	2012	Male	Positive	No
2992	VE7812	Miranda	Baruta	El Cafetal	*P. geniculatus*	2012	Female	Negative	No
2993	VE10412A	Miranda	Sucre	Filas de mariche	*P. geniculatus*	2012	Female	Negative	Yes
2994	VE10412B	Miranda	Sucre	Filas de mariche	*P. geniculatus*	2012	Female	Positive	No
2995	VE11212	Miranda	Sucre	Filas de mariche	*P. geniculatus*	2012	Female	Positive	Yes
2996	VE12112	Miranda	Sucre	Filas de mariche	*P. geniculatus*	2012	Male	Positive	Yes
2997	VE18912A	Miranda	Sucre	Filas de mariche	*P. geniculatus*	2012	Male	NA	NA
2998	VE18912B	Miranda	Sucre	Filas de mariche	*P. geniculatus*	2012	Male	Positive	No
2999	VE23412	Miranda	Sucre	Filas de mariche	*P. geniculatus*	2012	Female	Positive	Yes
3000	VE23512A	Miranda	Sucre	Filas de mariche	*P. geniculatus*	2012	Female	Positive	Yes
3001	VE23512B	Miranda	Sucre	Filas de mariche	*P. geniculatus*	2012	Male	Positive	Yes
3002	VE24912	Miranda	Sucre	Filas de mariche	*P. geniculatus*	2012	Male	Negative	No
3003	VE26712A	Miranda	Sucre	Filas de mariche	*P. geniculatus*	2012	Female	Positive	Yes
3004	VE26712B	Miranda	Sucre	Filas de mariche	*P. geniculatus*	2012	Female	NA	NA
3005	VE26712C	Miranda	Sucre	Filas de mariche	*P. geniculatus*	2012	Female	NA	NA
3006	VE26712D	Miranda	Sucre	Filas de mariche	*P. geniculatus*	2012	Female	NA	NA
3007	VE26712E	Miranda	Sucre	Filas de mariche	*P. geniculatus*	2012	Male	NA	NA
3008	VE26712F	Miranda	Sucre	Filas de mariche	*P. geniculatus*	2012	Male	NA	NA
3009	VE26712G	Miranda	Sucre	Filas de mariche	*P. geniculatus*	2012	Male	NA	NA
3010	VE27812	Miranda	Sucre	Filas de mariche	*P. geniculatus*	2012	Male	Positive	No
3011	VE2912	Miranda	Sucre	Filas de mariche	*P. geniculatus*	2012	Male	Positive	No
3012	VE29412	Miranda	Sucre	Filas de mariche	*P. geniculatus*	2012	Nymph IV	Positive	Yes
3013	VE32612A	Miranda	Sucre	Filas de mariche	*P. geniculatus*	2012	Male	NA	NA
3014	VE32612B	Miranda	Sucre	Filas de mariche	*P. geniculatus*	2012	Male	NA	NA
3015	VE32612C	Miranda	Sucre	Filas de mariche	*P. geniculatus*	2012	Male	NA	NA
3016	VE39712	Miranda	Sucre	Filas de mariche	*P. geniculatus*	2012	Female	Positive	Yes
3017	VE4712A	Miranda	Sucre	Filas de mariche	*P. geniculatus*	2012	Male	NA	NA
3018	VE4712B	Miranda	Sucre	Filas de mariche	*P. geniculatus*	2012	Male	Positive	No
3019	VE4812B	Miranda	Sucre	Filas de mariche	*P. geniculatus*	2012	Nymph III	Negative	Yes
3020	VE5812	Miranda	Sucre	Filas de mariche	*P. geniculatus*	2012	Female	Positive	Yes
3021	VE7112A	Miranda	Sucre	Filas de mariche	*P. geniculatus*	2012	Male	Positive	No
3022	VE7112B	Miranda	Sucre	Filas de mariche	*P. geniculatus*	2012	Female	Positive	No

3023	VE7112C	Miranda	Sucre	Filas de mariche	*P. geniculatus*	2012	Female	Negative	Yes
3024	VE9412	Miranda	Sucre	Filas de mariche	*P. geniculatus*	2012	Female	NA	No
3025	VE33012	Miranda	Sucre	La Dolorita	*P. geniculatus*	2012	Male	NA	NA
3026	VE7512A	Miranda	Sucre	La Dolorita	*P. geniculatus*	2012	Female	NA	NA
3027	VE7512B	Miranda	Sucre	La Dolorita	*P. geniculatus*	2012	Female	NA	NA
3028	VE7512C	Miranda	Sucre	La Dolorita	*P. geniculatus*	2012	Nymph V	NA	NA
3029	VE7512D	Miranda	Sucre	La Dolorita	*P. geniculatus*	2012	Nymph III	Positive	Yes
3030	VE7512E	Miranda	Sucre	La Dolorita	*P. geniculatus*	2012	Female	Positive	Yes
3031	VE1212	Miranda	Baruta	Las Minas de Baruta	*P. geniculatus*	2012	Male	Positive	No
3032	VE17212A	Miranda	Baruta	Las Minas de Baruta	*P. geniculatus*	2012	Male	Positive	Yes
3033	VE17212B	Miranda	Baruta	Las Minas de Baruta	*P. geniculatus*	2012	Male	Negative	No
3034	VE20612	Miranda	Baruta	Las Minas de Baruta	*P. geniculatus*	2012	Male	Positive	No
3035	VE21612	Miranda	Baruta	Las Minas de Baruta	*P. geniculatus*	2012	Male	Positive	No
3036	VE21912	Miranda	Baruta	Las Minas de Baruta	*P. geniculatus*	2012	Female	NA	Yes
3037	VE22312	Miranda	Baruta	Las Minas de Baruta	*P. geniculatus*	2012	Female	Positive	No
3038	VE38512A	Miranda	Baruta	Las Minas de Baruta	*P. geniculatus*	2012	Male	Positive	Yes
3039	VE38512B	Miranda	Baruta	Las Minas de Baruta	*P. geniculatus*	2012	Male	NA	NA
3040	VE38512C	Miranda	Baruta	Las Minas de Baruta	*P. geniculatus*	2012	Male	NA	NA
3041	VE38512D	Miranda	Baruta	Las Minas de Baruta	*P. geniculatus*	2012	Male	NA	NA
3042	VE38512E	Miranda	Baruta	Las Minas de Baruta	*P. geniculatus*	2012	Male	NA	NA
3043	VE38512F	Miranda	Baruta	Las Minas de Baruta	*P. geniculatus*	2012	Male	NA	NA
3044	VE6012	Miranda	Baruta	Las Minas de Baruta	*P. geniculatus*	2012	Female	Positive	No
3045	VE2612	Miranda	Baruta	Las Minas de Baruta	*P. geniculatus*	2012	Male	Positive	No
3046	VE13512	Miranda	Sucre	Leoncio Martínez	*P. geniculatus*	2012	Male	Positive	Yes
3047	VE13612	Miranda	Sucre	Leoncio Martínez	*P. geniculatus*	2012	Female	NA	No
3048	VE13812	Miranda	Sucre	Leoncio Martínez	*P. geniculatus*	2012	Female	Positive	Yes
3049	VE15012	Miranda	Sucre	Leoncio Martínez	*P. geniculatus*	2012	Male	Positive	No
3050	VE15112	Miranda	Sucre	Leoncio Martínez	*P. geniculatus*	2012	Female	Positive	Yes
3051	VE17712	Miranda	Sucre	Leoncio Martínez	*P. geniculatus*	2012	Male	NA	NA
3052	VE19712	Miranda	Sucre	Leoncio Martínez	*P. geniculatus*	2012	Male	Negative	No
3053	VE22112	Miranda	Sucre	Leoncio Martínez	*P. geniculatus*	2012	Female	Positive	Yes
3054	VE2512	Miranda	Sucre	Leoncio Martínez	*P. geniculatus*	2012	Female	Positive	No
3055	VE2812	Miranda	Sucre	Leoncio Martínez	*P. geniculatus*	2012	Male	Positive	No
3056	VE37312	Miranda	Sucre	Leoncio Martínez	*P. geniculatus*	2012	Male	NA	NA
3057	VE4812A	Miranda	Sucre	Leoncio Martínez	*P. geniculatus*	2012	Female	Positive	Yes
3058	VE5412	Miranda	Sucre	Leoncio Martínez	*P. geniculatus*	2012	Male	Positive	No
3059	VE6712	Miranda	Sucre	Leoncio Martínez	*P. geniculatus*	2012	Male	Positive	No
3060	VE7312	Miranda	Sucre	Leoncio Martínez	*P. geniculatus*	2012	Male	Positive	Yes
3061	VE8612	Miranda	Sucre	Leoncio Martínez	*P. geniculatus*	2012	Female	Positive	Yes
3062	VE0912	Miranda	Baruta	Nuestra Señora del Rosario de Baruta	*P. geniculatus*	2012	Female	Positive	No
3063	VE11412	Miranda	Baruta	Nuestra Señora del Rosario de Baruta	*P. geniculatus*	2012	Female	Positive	No
3064	VE12512	Miranda	Baruta	Nuestra Señora del Rosario de Baruta	*P. geniculatus*	2012	Female	NA	No

3065	VE12712	Miranda	Baruta	Nuestra Señora del Rosario de Baruta	*P. geniculatus*	2012	Female	Positive	Yes
3066	VE1312	Miranda	Baruta	Nuestra Señora del Rosario de Baruta	*T. maculata*	2012	Female	Positive	No
3067	VE14212	Miranda	Baruta	Nuestra Señora del Rosario de Baruta	*P. geniculatus*	2012	Female	Negative	Yes
3068	VE17312	Miranda	Baruta	Nuestra Señora del Rosario de Baruta	*P. geniculatus*	2012	Male	NA	NA
3069	VE17412	Miranda	Baruta	Nuestra Señora del Rosario de Baruta	*P. geniculatus*	2012	Female	Positive	No
3070	VE18012	Miranda	Baruta	Nuestra Señora del Rosario de Baruta	*P. geniculatus*	2012	Female	Positive	Yes
3071	VE19812A	Miranda	Baruta	Nuestra Señora del Rosario de Baruta	*P. geniculatus*	2012	Female	Positive	Yes
3072	VE19812B	Miranda	Baruta	Nuestra Señora del Rosario de Baruta	*P. geniculatus*	2012	Female	Positive	No
3073	VE20812	Miranda	Baruta	Nuestra Señora del Rosario de Baruta	*P. geniculatus*	2012	Female	Positive	Yes
3074	VE23612	Miranda	Baruta	Nuestra Señora del Rosario de Baruta	*P. geniculatus*	2012	Male	Positive	No
3075	VE23912	Miranda	Baruta	Nuestra Señora del Rosario de Baruta	*P. geniculatus*	2012	Male	Positive	No
3076	VE24012	Miranda	Baruta	Nuestra Señora del Rosario de Baruta	*P. geniculatus*	2012	Female	Negative	Yes
3077	VE25412	Miranda	Baruta	Nuestra Señora del Rosario de Baruta	*P. geniculatus*	2012	Male	NA	NA
3078	VE27012	Miranda	Baruta	Nuestra Señora del Rosario de Baruta	*P. geniculatus*	2012	Female	Positive	No
3079	VE27112	Miranda	Baruta	Nuestra Señora del Rosario de Baruta	*P. geniculatus*	2012	Female	Positive	No
3080	VE30912	Miranda	Baruta	Nuestra Señora del Rosario de Baruta	*P. geniculatus*	2012	Female	Positive	Yes
3081	VE32412	Miranda	Baruta	Nuestra Señora del Rosario de Baruta	*P. geniculatus*	2012	Female	Positive	No
3082	VE33212	Miranda	Baruta	Nuestra Señora del Rosario de Baruta	*P. geniculatus*	2012	Female	Negative	Yes
3083	VE33312	Miranda	Baruta	Nuestra Señora del Rosario de Baruta	*P. geniculatus*	2012	Female	NA	Yes
3084	VE3412	Miranda	Baruta	Nuestra Señora del Rosario de Baruta	*P. geniculatus*	2012	Female	Positive	Yes
3085	VE34312	Miranda	Baruta	Nuestra Señora del Rosario de Baruta	*P. geniculatus*	2012	Female	Positive	Yes
3086	VE34912	Miranda	Baruta	Nuestra Señora del Rosario de Baruta	*P. geniculatus*	2012	Male	Positive	No
3087	VE35212	Miranda	Baruta	Nuestra Señora del Rosario de Baruta	*P. geniculatus*	2012	Male	Negative	No
3088	VE36112	Miranda	Baruta	Nuestra Señora del Rosario de Baruta	*P. geniculatus*	2012	Female	Negative	Yes
3089	VE37512	Miranda	Baruta	Nuestra Señora del Rosario de Baruta	*P. geniculatus*	2012	Female	NA	NA
3090	VE39612	Miranda	Baruta	Nuestra Señora del Rosario de Baruta	*P. geniculatus*	2012	Nymph V	Negative	Yes
3091	VE5212	Miranda	Baruta	Nuestra Señora del Rosario de Baruta	*P. geniculatus*	2012	Female	Positive	Yes
3092	VE5312	Miranda	Baruta	Nuestra Señora del Rosario de Baruta	*P. geniculatus*	2012	Male	Positive	No
3093	VE5512	Miranda	Baruta	Nuestra Señora del Rosario de Baruta	*P. geniculatus*	2012	Male	Positive	No
3094	VE5712	Miranda	Baruta	Nuestra Señora del Rosario de Baruta	*P. geniculatus*	2012	Female	Negative	Yes
3095	VE5912	Miranda	Baruta	Nuestra Señora del Rosario de Baruta	*P. geniculatus*	2012	Male	Positive	Yes
3096	VE8012	Miranda	Baruta	Nuestra Señora del Rosario de Baruta	*P. geniculatus*	2012	Female	Positive	Yes
3097	VE8212	Miranda	Baruta	Nuestra Señora del Rosario de Baruta	*P. geniculatus*	2012	Female	Positive	Yes
3098	VE9112	Miranda	Baruta	Nuestra Señora del Rosario de Baruta	*P. geniculatus*	2012	Male	Negative	No
3099	VE0712	Miranda	Sucre	Petare	*P. geniculatus*	2012	Male	Positive	No
3100	VE1012	Miranda	Sucre	Petare	*P. geniculatus*	2012	Female	NA	No
3101	VE10312A	Miranda	Sucre	Petare	*P. geniculatus*	2012	Male	Positive	No
3102	VE10312B	Miranda	Sucre	Petare	*P. geniculatus*	2012	Male	Positive	No
3103	VE10512	Miranda	Sucre	Petare	*P. geniculatus*	2012	Female	NA	Yes
3104	VE11712	Miranda	Sucre	Petare	*P. geniculatus*	2012	Male	Negative	Yes
3105	VE11812	Miranda	Sucre	Petare	*P. geniculatus*	2012	Female	Positive	Yes
3106	VE11912	Miranda	Sucre	Petare	*P. geniculatus*	2012	Female	Positive	No

3107	VE1412	Miranda	Sucre	Petare	*P. geniculatus*	2012	Female	Positive	No
3108	VE14412	Miranda	Sucre	Petare	*P. geniculatus*	2012	Female	Negative	Yes
3109	VE14512	Miranda	Sucre	Petare	*P. geniculatus*	2012	Female	Positive	No
3110	VE15612	Miranda	Sucre	Petare	*P. geniculatus*	2012	Female	Positive	Yes
3111	VE1612	Miranda	Sucre	Petare	*P. geniculatus*	2012	Female	Positive	No
3112	VE16212	Miranda	Sucre	Petare	*P. geniculatus*	2012	Male	NA	No
3113	VE16912	Miranda	Sucre	Petare	*P. geniculatus*	2012	Female	Positive	No
3114	VE17012	Miranda	Sucre	Petare	*P. geniculatus*	2012	Male	Positive	Yes
3115	VE17112	Miranda	Sucre	Petare	*P. geniculatus*	2012	Female	Positive	Yes
3116	VE18112	Miranda	Sucre	Petare	*P. geniculatus*	2012	Female	Positive	Yes
3117	VE18212	Miranda	Sucre	Petare	*P. geniculatus*	2012	Male	Positive	No
3118	VE18312	Miranda	Sucre	Petare	*P. geniculatus*	2012	Male	NA	NA
3119	VE1912	Miranda	Sucre	Petare	*P. geniculatus*	2012	Female	Negative	Yes
3120	VE20212	Miranda	Sucre	Petare	*P. geniculatus*	2012	Female	Positive	Yes
3121	VE20312	Miranda	Sucre	Petare	*P. geniculatus*	2012	Female	Positive	No
3122	VE20512	Miranda	Sucre	Petare	*P. geniculatus*	2012	Female	Positive	Yes
3123	VE21112	Miranda	Sucre	Petare	*P. geniculatus*	2012	Female	NA	NA
3124	VE22012	Miranda	Sucre	Petare	*P. geniculatus*	2012	Female	Negative	Yes
3125	VE2212	Miranda	Sucre	Petare	*P. geniculatus*	2012	Male	NA	No
3126	VE22212	Miranda	Sucre	Petare	*P. geniculatus*	2012	Female	Positive	No
3127	VE22612	Miranda	Sucre	Petare	*P. geniculatus*	2012	Male	Negative	Yes
3128	VE22712	Miranda	Sucre	Petare	*P. geniculatus*	2012	Male	Negative	No
3129	VE23012	Miranda	Sucre	Petare	*P. geniculatus*	2012	Female	Positive	Yes
3130	VE2312	Miranda	Sucre	Petare	*P. geniculatus*	2012	Female	Negative	Yes
3131	VE23212A	Miranda	Sucre	Petare	*P. geniculatus*	2012	Male	Positive	No
3132	VE23812	Miranda	Sucre	Petare	*P. geniculatus*	2012	Female	Positive	Yes
3133	VE26412	Miranda	Sucre	Petare	*P. geniculatus*	2012	Nymph V	Positive	Yes
3134	VE26612	Miranda	Sucre	Petare	*P. geniculatus*	2012	Female	Positive	Yes
3135	VE27512A	Miranda	Sucre	Petare	*P. geniculatus*	2012	Male	NA	No
3136	VE27512B	Miranda	Sucre	Petare	*P. geniculatus*	2012	Nymph IV	Positive	Yes
3137	VE27712	Miranda	Sucre	Petare	*P. geniculatus*	2012	Male	Positive	Yes
3138	VE28012	Miranda	Sucre	Petare	*P. geniculatus*	2012	Female	Positive	Yes
3139	VE28412	Miranda	Sucre	Petare	*P. geniculatus*	2012	Nymph V	Positive	Yes
3140	VE28612	Miranda	Sucre	Petare	*P. geniculatus*	2012	Male	Positive	No
3141	VE28912	Miranda	Sucre	Petare	*P. geniculatus*	2012	Female	Positive	Yes
3142	VE29112	Miranda	Sucre	Petare	*P. geniculatus*	2012	Female	Negative	No
3143	VE29612A	Miranda	Sucre	Petare	*P. geniculatus*	2012	Male	Positive	No
3144	VE29612B	Miranda	Sucre	Petare	*P. geniculatus*	2012	Female	Positive	No
3145	VE29712	Miranda	Sucre	Petare	*P. geniculatus*	2012	Female	Positive	Yes
3146	VE3012	Miranda	Sucre	Petare	*P. geniculatus*	2012	Female	Positive	No
3147	VE30412	Miranda	Sucre	Petare	*P. geniculatus*	2012	Female	Positive	Yes
3148	VE30512	Miranda	Sucre	Petare	*P. geniculatus*	2012	Female	Positive	Yes

3149	VE30712	Miranda	Sucre	Petare	*P. geniculatus*	2012	Female	Positive	Yes
3150	VE31812	Miranda	Sucre	Petare	*P. geniculatus*	2012	Female	Positive	Yes
3151	VE31912	Miranda	Sucre	Petare	*P. geniculatus*	2012	Nymph III	Negative	Yes
3152	VE3212	Miranda	Sucre	Petare	*P. geniculatus*	2012	Male	Positive	Yes
3153	VE32312	Miranda	Sucre	Petare	*P. geniculatus*	2012	Female	Positive	No
3154	VE32512	Miranda	Sucre	Petare	*P. geniculatus*	2012	Male	Positive	Yes
3155	VE33612	Miranda	Sucre	Petare	*P. geniculatus*	2012	Female	Negative	Yes
3156	VE34812	Miranda	Sucre	Petare	*P. geniculatus*	2012	Male	Positive	Yes
3157	VE35012	Miranda	Sucre	Petare	*P. geniculatus*	2012	Female	Positive	Yes
3158	VE35112	Miranda	Sucre	Petare	*P. geniculatus*	2012	Female	NA	Yes
3159	VE35612	Miranda	Sucre	Petare	*P. geniculatus*	2012	Female	Negative	Yes
3160	VE35712	Miranda	Sucre	Petare	*P. geniculatus*	2012	Female	NA	NA
3161	VE35812	Miranda	Sucre	Petare	*P. geniculatus*	2012	Female	NA	Yes
3162	VE36512	Miranda	Sucre	Petare	*P. geniculatus*	2012	Male	Positive	No
3163	VE36912	Miranda	Sucre	Petare	*P. geniculatus*	2012	Female	Positive	Yes
3164	VE37112	Miranda	Sucre	Petare	*P. geniculatus*	2012	Female	NA	Yes
3165	VE38712	Miranda	Sucre	Petare	*P. geniculatus*	2012	Male	Positive	No
3166	VE38812	Miranda	Sucre	Petare	*P. geniculatus*	2012	Male	Positive	Yes
3167	VE39112A	Miranda	Sucre	Petare	*P. geniculatus*	2012	Female	Positive	Yes
3168	VE39112B	Miranda	Sucre	Petare	*P. geniculatus*	2012	Male	Positive	Yes
3169	VE39212	Miranda	Sucre	Petare	*P. geniculatus*	2012	Female	Positive	Yes
3170	VE39412	Miranda	Sucre	Petare	*P. geniculatus*	2012	Female	Positive	No
3171	VE39512	Miranda	Sucre	Petare	*P. geniculatus*	2012	Male	NA	NA
3172	VE4012	Miranda	Sucre	Petare	*P. geniculatus*	2012	Female	Positive	Yes
3173	VE4212	Miranda	Sucre	Petare	*P. geniculatus*	2012	Female	Positive	No
3174	VE5012	Miranda	Sucre	Petare	*P. geniculatus*	2012	Male	NA	Yes
3175	VE6212	Miranda	Sucre	Petare	*P. geniculatus*	2012	Male	NA	No
3176	VE6312	Miranda	Sucre	Petare	*P. geniculatus*	2012	Female	Positive	Yes
3177	VE6612	Miranda	Sucre	Petare	*P. geniculatus*	2012	Female	Positive	Yes
3178	VE7412	Miranda	Sucre	Petare	*P. geniculatus*	2012	Female	Positive	Yes
3179	VE9212	Miranda	Sucre	Petare	*P. geniculatus*	2012	Male	Positive	No
3180	VE9912	Miranda	Sucre	Petare	*P. geniculatus*	2012	Female	Negative	Yes
3181	VE0812A	Miranda	El Hatillo	Santa Rosalía de Palermo	*P. geniculatus*	2012	Female	Negative	No
3182	VE10112	Miranda	El Hatillo	Santa Rosalía de Palermo	*P. geniculatus*	2012	Female	Positive	Yes
3183	VE12312A	Miranda	El Hatillo	Santa Rosalía de Palermo	*P. geniculatus*	2012	Male	Positive	No
3184	VE12312C	Miranda	El Hatillo	Santa Rosalía de Palermo	*P. geniculatus*	2012	Female	Positive	No
3185	VE13412	Miranda	El Hatillo	Santa Rosalía de Palermo	*P. geniculatus*	2012	Female	Positive	No
3186	VE23712	Miranda	El Hatillo	Santa Rosalía de Palermo	*P. geniculatus*	2012	Female	Positive	No
3187	VE24412	Miranda	El Hatillo	Santa Rosalía de Palermo	*P. geniculatus*	2012	Female	Positive	Yes
3188	VE25812	Miranda	El Hatillo	Santa Rosalía de Palermo	*P. geniculatus*	2012	Female	NA	NA
3189	VE26512	Miranda	El Hatillo	Santa Rosalía de Palermo	*P. geniculatus*	2012	Female	Positive	Yes
3190	VE26912	Miranda	El Hatillo	Santa Rosalía de Palermo	*T. nigromaculata*	2012	Female	Negative	No

3191	VE27312	Miranda	El Hatillo	Santa Rosalía de Palermo	*P. geniculatus*	2012	Male	Positive	Yes
3192	VE27612	Miranda	El Hatillo	Santa Rosalía de Palermo	*P. geniculatus*	2012	Female	NA	NA
3193	VE29212	Miranda	El Hatillo	Santa Rosalía de Palermo	*P. geniculatus*	2012	Male	Positive	Yes
3194	VE30212	Miranda	El Hatillo	Santa Rosalía de Palermo	*P. geniculatus*	2012	Female	Positive	Yes
3195	VE31212	Miranda	El Hatillo	Santa Rosalía de Palermo	*P. geniculatus*	2012	Nymph IV	Negative	Yes
3196	VE33912	Miranda	El Hatillo	Santa Rosalía de Palermo	*T. nigromaculata*	2012	Female	NA	NA
3197	VE36012	Miranda	El Hatillo	Santa Rosalía de Palermo	*P. geniculatus*	2012	Female	Positive	Yes
3198	VE37912	Miranda	El Hatillo	Santa Rosalía de Palermo	*T. maculata*	2012	Male	Positive	No
3199	VE38912	Miranda	El Hatillo	Santa Rosalía de Palermo	*P. geniculatus*	2012	Male	Positive	No
3200	VE7712	Miranda	El Hatillo	Santa Rosalía de Palermo	*P. geniculatus*	2012	Female	Negative	Yes
3201	VE8812	Miranda	El Hatillo	Santa Rosalía de Palermo	*P. geniculatus*	2012	Male	Negative	Yes
3202	VE26013	DC	Libertador	Altagracia	*P. geniculatus*	2013	Female	Positive	No
3203	VE38213	DC	Libertador	Altagracia	*P. geniculatus*	2013	Male	Negative	No
3204	VE3913	DC	Libertador	Antímano	*P. geniculatus*	2013	Female	Negative	Yes
3205	VE3613	DC	Libertador	Antímano	*P. geniculatus*	2013	Female	Positive	Yes
3206	VE19913	DC	Libertador	Antímano	*P. geniculatus*	2013	Female	Positive	Yes
3207	VE13413	DC	Libertador	Antímano	*P. geniculatus*	2013	Female	NA	Yes
3208	VE27413	DC	Libertador	Antímano	*P. geniculatus*	2013	Male	NA	Yes
3209	VE33513	DC	Libertador	Antímano	*P. geniculatus*	2013	Male	Positive	No
3210	VE25013	DC	Libertador	Caricuao	*P. geniculatus*	2013	Female	Negative	Yes
3211	VE33613	DC	Libertador	Caricuao	*P. geniculatus*	2013	Female	Positive	Yes
3212	VE24613	DC	Libertador	Caricuao	*P. geniculatus*	2013	Female	Positive	Yes
3213	VE4213A	DC	Libertador	Caricuao	*P. geniculatus*	2013	Male	NA	No
3214	VE4213B	DC	Libertador	Caricuao	*P. geniculatus*	2013	Male	Positive	No
3215	VE26313	DC	Libertador	Caricuao	*P. geniculatus*	2013	Male	NA	No
3216	VE5313	DC	Libertador	Caricuao	*P. geniculatus*	2013	Nymph IV	Positive	Yes
3217	VE8413	DC	Libertador	Coche	*P. geniculatus*	2013	Female	Positive	Yes
3218	VE4713	DC	Libertador	El Junquito	*P. geniculatus*	2013	Female	Positive	Yes
3219	VE8913	DC	Libertador	El Junquito	*P. geniculatus*	2013	Female	Positive	Yes
3220	VE9313	DC	Libertador	El Junquito	*P. geniculatus*	2013	Female	Negative	Yes
3221	VE10513	DC	Libertador	El Junquito	*P. geniculatus*	2013	Female	NA	NA
3222	VE22613	DC	Libertador	El Junquito	*P. geniculatus*	2013	Female	Positive	Yes
3223	VE28313	DC	Libertador	El Junquito	*P. geniculatus*	2013	Female	Positive	Yes
3224	VE29113	DC	Libertador	El Junquito	*P. geniculatus*	2013	Female	Positive	Yes
3225	VE29313	DC	Libertador	El Junquito	*P. geniculatus*	2013	Female	NA	No
3226	VE35113	DC	Libertador	El Junquito	*P. geniculatus*	2013	Female	NA	No
3227	VE35713	DC	Libertador	El Junquito	*P. geniculatus*	2013	Female	Positive	Yes
3228	VE35313	DC	Libertador	El Junquito	*P. geniculatus*	2013	Female	Positive	No
3229	VE7513	DC	Libertador	El Junquito	*P. geniculatus*	2013	Male	Positive	No
3230	VE13613	DC	Libertador	El Junquito	*P. geniculatus*	2013	Male	Positive	No
3231	VE16713	DC	Libertador	El Junquito	*P. geniculatus*	2013	Male	Positive	Yes
3232	VE22113	DC	Libertador	El Junquito	*P. geniculatus*	2013	Male	Positive	No

3233	VE26213	DC	Libertador	El Junquito	*P. geniculatus*	2013	Male	Positive	No
3234	VE27013A	DC	Libertador	El Junquito	*P. geniculatus*	2013	Male	NA	Yes
3235	VE27013B	DC	Libertador	El Junquito	*P. geniculatus*	2013	Male	NA	Yes
3236	VE27013C	DC	Libertador	El Junquito	*P. geniculatus*	2013	Male	Positive	Yes
3237	VE27013D	DC	Libertador	El Junquito	*P. geniculatus*	2013	Male	Positive	Yes
3238	VE33413	DC	Libertador	El Junquito	*P. geniculatus*	2013	Male	Positive	Yes
3239	VE33913	DC	Libertador	El Junquito	*T. maculata*	2013	Male	NA	No
3240	VE18313	DC	Libertador	El Paraiso	*P. geniculatus*	2013	Female	Positive	Yes
3241	VE37113	DC	Libertador	El Recreo	*P. geniculatus*	2013	Female	Positive	No
3242	VE36813	DC	Libertador	El Recreo	*P. geniculatus*	2013	Male	Positive	No
3243	VE37313	DC	Libertador	El Recreo	*P. geniculatus*	2013	Male	Positive	Yes
3244	VE25513	DC	Libertador	El Recreo	*P. geniculatus*	2013	Male	Positive	No
3245	VE11913	DC	Libertador	El Recreo	*P. geniculatus*	2013	Male	Positive	Yes
3246	VE7413	DC	Libertador	La Pastora	*P. geniculatus*	2013	Female	Positive	Yes
3247	VE10313	DC	Libertador	La Pastora	*P. geniculatus*	2013	Female	Positive	Yes
3248	VE30013	DC	Libertador	La Pastora	*P. geniculatus*	2013	Female	Positive	Yes
3249	VE34513	DC	Libertador	La Pastora	*P. geniculatus*	2013	Female	Positive	Yes
3250	VE17913	DC	Libertador	La Pastora	*P. geniculatus*	2013	Female	Positive	Yes
3251	VE27313	DC	Libertador	La Pastora	*P. geniculatus*	2013	Female	Positive	No
3252	VE35413	DC	Libertador	La Pastora	*P. geniculatus*	2013	Female	Negative	Yes
3253	VE8313	DC	Libertador	La Pastora	*P. geniculatus*	2013	Male	NA	No
3254	VE13513	DC	Libertador	La Pastora	*P. geniculatus*	2013	Male	Positive	Yes
3255	VE14813A	DC	Libertador	La Pastora	*P. geniculatus*	2013	Male	NA	No
3256	VE14813B	DC	Libertador	La Pastora	*P. geniculatus*	2013	Male	NA	No
3257	VE26813	DC	Libertador	La Pastora	*P. geniculatus*	2013	Male	Positive	No
3258	VE30813	DC	Libertador	La Pastora	*P. geniculatus*	2013	Male	NA	No
3259	VE34313	DC	Libertador	La Pastora	*P. geniculatus*	2013	Male	Positive	No
3260	VE3313	DC	Libertador	La Vega	*P. geniculatus*	2013	Female	Positive	No
3261	VE18613	DC	Libertador	La Vega	*P. geniculatus*	2013	Female	NA	Yes
3262	VE27713	DC	Libertador	La Vega	*P. geniculatus*	2013	Female	Positive	Yes
3263	VE21213	DC	Libertador	La Vega	*P. geniculatus*	2013	Female	Negative	Yes
3264	VE14213	DC	Libertador	La Vega	*P. geniculatus*	2013	Female	Positive	No
3265	VE21613A	DC	Libertador	La Vega	*P. geniculatus*	2013	Male	Positive	No
3266	VE10613	DC	Libertador	La Vega	*P. geniculatus*	2013	Male	Positive	No
3267	VE14113	DC	Libertador	San Agustín	*P. geniculatus*	2013	Female	NA	NA
3268	VE6913	DC	Libertador	San Bernardino	*P. geniculatus*	2013	Female	NA	NA
3269	VE13813	DC	Libertador	San Bernardino	*P. geniculatus*	2013	Female	Positive	Yes
3270	VE12913	DC	Libertador	San José	*P. geniculatus*	2013	Female	NA	NA
3271	VE18113	DC	Libertador	San José	*P. geniculatus*	2013	Female	NA	No
3272	VE36013	DC	Libertador	San Pedro	*P. geniculatus*	2013	Female	Positive	Yes
3273	VE33713	DC	Libertador	San Pedro	*P. geniculatus*	2013	Male	Positive	No
3274	VE29013	DC	Libertador	Santa Rosalía	*P. geniculatus*	2013	Male	NA	No

3275	VE0713	DC	Libertador	Santa Rosalía	*P. geniculatus*	2013	Female	NA	No
3276	VE19513	DC	Libertador	Santa Teresa	*P. geniculatus*	2013	Male	Negative	No
3277	VE3713	DC	Libertador	Sucre	*P. geniculatus*	2013	Female	Positive	Yes
3278	VE15813	DC	Libertador	Sucre	*P. geniculatus*	2013	Female	NA	Yes
3279	VE17613	DC	Libertador	Sucre	*P. geniculatus*	2013	Female	NA	No
3280	VE16113	DC	Libertador	Sucre	*P. geniculatus*	2013	Female	Positive	Yes
3281	VE2113	DC	Libertador	Sucre	*P. geniculatus*	2013	Female	Positive	Yes
3282	VE15913	DC	Libertador	Sucre	*P. geniculatus*	2013	Female	NA	Yes
3283	VE19013	DC	Libertador	Sucre	*P. geniculatus*	2013	Female	NA	NA
3284	VE22413	DC	Libertador	Sucre	*P. geniculatus*	2013	Female	Positive	Yes
3285	VE24913	DC	Libertador	Sucre	*P. geniculatus*	2013	Female	Positive	No
3286	VE25813	DC	Libertador	Sucre	*P. geniculatus*	2013	Female	Positive	Yes
3287	VE26513	DC	Libertador	Sucre	*P. geniculatus*	2013	Female	Positive	Yes
3288	VE32813	DC	Libertador	Sucre	*P. geniculatus*	2013	Female	Negative	No
3289	VE22713B	DC	Libertador	Sucre	*P. geniculatus*	2013	Female	Positive	Yes
3290	VE11413	DC	Libertador	Sucre	*P. geniculatus*	2013	Female	Positive	Yes
3291	VE23413	DC	Libertador	Sucre	*P. geniculatus*	2013	Female	NA	No
3292	VE11013	DC	Libertador	Sucre	*P. geniculatus*	2013	Female	Positive	No
3293	VE18513	DC	Libertador	Sucre	*P. geniculatus*	2013	Female	Positive	Yes
3294	VE12413	DC	Libertador	Sucre	*P. geniculatus*	2013	Female	NA	Yes
3295	VE0913	DC	Libertador	Sucre	*P. geniculatus*	2013	Female	NA	Yes
3296	VE2213	DC	Libertador	Sucre	*P. geniculatus*	2013	Female	Positive	Yes
3297	VE3513	DC	Libertador	Sucre	*P. geniculatus*	2013	Female	Positive	Yes
3298	VE8213	DC	Libertador	Sucre	*P. geniculatus*	2013	Female	Negative	No
3299	VE34613	DC	Libertador	Sucre	*P. geniculatus*	2013	Female	Negative	Yes
3300	VE16513A	DC	Libertador	Sucre	*P. geniculatus*	2013	Female	Positive	Yes
3301	VE16513B	DC	Libertador	Sucre	*P. geniculatus*	2013	Female	Positive	Yes
3302	VE16513C	DC	Libertador	Sucre	*P. geniculatus*	2013	Female	NA	NA
3303	VE21513A	DC	Libertador	Sucre	*P. geniculatus*	2013	Female	NA	Yes
3304	VE21513B	DC	Libertador	Sucre	*P. geniculatus*	2013	Female	Positive	Yes
3305	VE18813A	DC	Libertador	Sucre	*P. geniculatus*	2013	Female	NA	No
3306	VE18813B	DC	Libertador	Sucre	*P. geniculatus*	2013	Female	NA	No
3307	VE18813C	DC	Libertador	Sucre	*P. geniculatus*	2013	Female	Positive	Yes
3308	VE19213A	DC	Libertador	Sucre	*P. geniculatus*	2013	Female	NA	NA
3309	VE19213B	DC	Libertador	Sucre	*P. geniculatus*	2013	Female	NA	NA
3310	VE20713	DC	Libertador	Sucre	*P. geniculatus*	2013	Female	Positive	Yes
3311	VE31413	DC	Libertador	Sucre	*P. geniculatus*	2013	Female	Positive	Yes
3312	VE18413	DC	Libertador	Sucre	*P. geniculatus*	2013	Female	Positive	Yes
3313	VE34013	DC	Libertador	Sucre	*P. geniculatus*	2013	Female	Positive	Yes
3314	VE16913	DC	Libertador	Sucre	*P. geniculatus*	2013	Female	Positive	No
3315	VE30913	DC	Libertador	Sucre	*P. geniculatus*	2013	Male	Positive	No
3316	VE37513	DC	Libertador	Sucre	*P. geniculatus*	2013	Male	Positive	No

3317	VE36413	DC	Libertador	Sucre	*P. geniculatus*	2013	Male	NA	No
3318	VE9713	DC	Libertador	Sucre	*P. geniculatus*	2013	Male	Positive	Yes
3319	VE19813	DC	Libertador	Sucre	*P. geniculatus*	2013	Male	Positive	Yes
3320	VE9613	DC	Libertador	Sucre	*P. geniculatus*	2013	Male	Positive	Yes
3321	VE0313	DC	Libertador	Sucre	*P. geniculatus*	2013	Male	Positive	Yes
3322	VE0813	DC	Libertador	Sucre	*P. geniculatus*	2013	Male	NA	No
3323	VE5013	DC	Libertador	Sucre	*P. geniculatus*	2013	Male	Positive	Yes
3324	VE3113	DC	Libertador	Sucre	*P. geniculatus*	2013	Male	Positive	No
3325	VE19213C	DC	Libertador	Sucre	*P. geniculatus*	2013	Male	Positive	No
3326	VE4613	DC	Libertador	Sucre	*P. geniculatus*	2013	Male	Negative	No
3327	VE24713	DC	Libertador	Sucre	*P. geniculatus*	2013	Male	Positive	Yes
3328	VE24213	Miranda	Baruta	El Cafetal	*P. geniculatus*	2013	Female	Negative	Yes
3329	VE5713	Miranda	Baruta	El Cafetal	*P. geniculatus*	2013	Female	Positive	No
3330	VE23113	Miranda	Baruta	El Cafetal	*P. geniculatus*	2013	Female	NA	No
3331	VE18213	Miranda	Baruta	El Cafetal	*P. geniculatus*	2013	Female	Positive	No
3332	VE30313	Miranda	Baruta	El Cafetal	*P. geniculatus*	2013	Female	NA	No
3333	VE38713	Miranda	Baruta	El Cafetal	*P. geniculatus*	2013	Male	Positive	No
3334	VE3813	Miranda	Baruta	El Cafetal	*P. geniculatus*	2013	Male	Positive	Yes
3335	VE30513	Miranda	Baruta	El Cafetal	*P. geniculatus*	2013	Male	Positive	Yes
3336	VE2613	Miranda	Baruta	El Cafetal	*P. geniculatus*	2013	Male	Positive	No
3337	VE11513	Miranda	Baruta	El Cafetal	*P. geniculatus*	2013	Male	NA	NA
3338	VE17113	Miranda	Baruta	El Cafetal	*P. geniculatus*	2013	Male	Positive	No
3339	VE31913	Miranda	Baruta	El Cafetal	*P. geniculatus*	2013	Male	Positive	No
3340	VE36913	Miranda	Baruta	El Cafetal	*P. geniculatus*	2013	Male	Positive	Yes
3341	VE17413	Miranda	Baruta	El Cafetal	*P. geniculatus*	2013	Male	Positive	No
3342	VE25913	Miranda	Baruta	El Cafetal	*P. geniculatus*	2013	Male	Positive	Yes
3343	VE34413	Miranda	Baruta	Las minas de Baruta	*P. geniculatus*	2013	Female	Positive	No
3344	VE0113	Miranda	Baruta	Las minas de Baruta	*P. geniculatus*	2013	Female	NA	NA
3345	VE12513	Miranda	Baruta	Las minas de Baruta	*P. geniculatus*	2013	Female	Positive	Yes
3346	VE9913	Miranda	Baruta	Las minas de Baruta	*P. geniculatus*	2013	Female	Negative	Yes
3347	VE12313	Miranda	Baruta	Las minas de Baruta	*P. geniculatus*	2013	Female	Positive	Yes
3348	VE21713B	Miranda	Baruta	Las minas de Baruta	*T. maculata*	2013	Female	Negative	No
3349	VE39313	Miranda	Baruta	Las minas de Baruta	*P. geniculatus*	2013	Female	Negative	Yes
3350	VE7313	Miranda	Baruta	Las minas de Baruta	*P. geniculatus*	2013	Female	Positive	No
3351	VE33313	Miranda	Baruta	Las minas de Baruta	*P. geniculatus*	2013	Female	Positive	Yes
3352	VE4113	Miranda	Baruta	Las minas de Baruta	*P. geniculatus*	2013	Female	Negative	Yes
3353	VE1913	Miranda	Baruta	Las minas de Baruta	*P. geniculatus*	2013	Male	Positive	Yes
3354	VE7913	Miranda	Baruta	Las minas de Baruta	*P. geniculatus*	2013	Male	Positive	Yes
3355	VE5113	Miranda	Baruta	Las minas de Baruta	*P. geniculatus*	2013	Nymph IV	Negative	Yes
3356	VE30113	Miranda	Baruta	Nuestra Señora del Rosario de Baruta	*P. geniculatus*	2013	Female	Positive	Yes
3357	VE8013	Miranda	Baruta	Nuestra Señora del Rosario de Baruta	*P. geniculatus*	2013	Female	Negative	No
3358	VE10213	Miranda	Baruta	Nuestra Señora del Rosario de Baruta	*P. geniculatus*	2013	Female	Negative	Yes

3359	VE10813	Miranda	Baruta	Nuestra Señora del Rosario de Baruta	*P. geniculatus*	2013	Female	NA	NA
3360	VE31313	Miranda	Baruta	Nuestra Señora del Rosario de Baruta	*P. geniculatus*	2013	Female	Positive	Yes
3361	VE21813	Miranda	Baruta	Nuestra Señora del Rosario de Baruta	*P. geniculatus*	2013	Female	Positive	No
3362	VE10113A	Miranda	Baruta	Nuestra Señora del Rosario de Baruta	*P. geniculatus*	2013	Female	Positive	Yes
3363	VE11813	Miranda	Baruta	Nuestra Señora del Rosario de Baruta	*P. geniculatus*	2013	Female	NA	NA
3364	VE20013	Miranda	Baruta	Nuestra Señora del Rosario de Baruta	*P. geniculatus*	2013	Female	Positive	Yes
3365	VE28513	Miranda	Baruta	Nuestra Señora del Rosario de Baruta	*P. geniculatus*	2013	Female	Positive	Yes
3366	VE7213	Miranda	Baruta	Nuestra Señora del Rosario de Baruta	*P. geniculatus*	2013	Female	Positive	No
3367	VE17013	Miranda	Baruta	Nuestra Señora del Rosario de Baruta	*P. geniculatus*	2013	Female	NA	Yes
3368	VE23913	Miranda	Baruta	Nuestra Señora del Rosario de Baruta	*P. geniculatus*	2013	Female	Positive	Yes
3369	VE23013	Miranda	Baruta	Nuestra Señora del Rosario de Baruta	*P. geniculatus*	2013	Female	Positive	Yes
3370	VE19413	Miranda	Baruta	Nuestra Señora del Rosario de Baruta	*P. geniculatus*	2013	Female	NA	Yes
3371	VE15513	Miranda	Baruta	Nuestra Señora del Rosario de Baruta	*P. geniculatus*	2013	Female	Positive	No
3372	VE19613	Miranda	Baruta	Nuestra Señora del Rosario de Baruta	*P. geniculatus*	2013	Female	Positive	No
3373	VE24013	Miranda	Baruta	Nuestra Señora del Rosario de Baruta	*P. geniculatus*	2013	Female	Negative	Yes
3374	VE28913	Miranda	Baruta	Nuestra Señora del Rosario de Baruta	*P. geniculatus*	2013	Female	Negative	Yes
3375	VE12013	Miranda	Baruta	Nuestra Señora del Rosario de Baruta	*P. geniculatus*	2013	Female	Positive	No
3376	VE11113	Miranda	Baruta	Nuestra Señora del Rosario de Baruta	*P. geniculatus*	2013	Female	Positive	Yes
3377	VE26413	Miranda	Baruta	Nuestra Señora del Rosario de Baruta	*P. geniculatus*	2013	Female	Positive	Yes
3378	VE20213	Miranda	Baruta	Nuestra Señora del Rosario de Baruta	*P. geniculatus*	2013	Female	NA	NA
3379	VE32513	Miranda	Baruta	Nuestra Señora del Rosario de Baruta	*P. geniculatus*	2013	Female	Positive	Yes
3380	VE32613	Miranda	Baruta	Nuestra Señora del Rosario de Baruta	*P. geniculatus*	2013	Female	Positive	Yes
3381	VE7713	Miranda	Baruta	Nuestra Señora del Rosario de Baruta	*P. geniculatus*	2013	Male	Positive	Yes
3382	VE13313	Miranda	Baruta	Nuestra Señora del Rosario de Baruta	*P. geniculatus*	2013	Male	NA	NA
3383	VE9513	Miranda	Baruta	Nuestra Señora del Rosario de Baruta	*P. geniculatus*	2013	Male	Negative	Yes
3384	VE7813	Miranda	Baruta	Nuestra Señora del Rosario de Baruta	*P. geniculatus*	2013	Male	Negative	No
3385	VE8113	Miranda	Baruta	Nuestra Señora del Rosario de Baruta	*P. geniculatus*	2013	Male	Negative	No
3386	VE24813	Miranda	Baruta	Nuestra Señora del Rosario de Baruta	*P. geniculatus*	2013	Male	Positive	Yes
3387	VE19713	Miranda	Baruta	Nuestra Señora del Rosario de Baruta	*P. geniculatus*	2013	Male	Positive	No
3388	VE10113B	Miranda	Baruta	Nuestra Señora del Rosario de Baruta	*P. geniculatus*	2013	Male	Positive	Yes
3389	VE11613	Miranda	Baruta	Nuestra Señora del Rosario de Baruta	*P. geniculatus*	2013	Male	NA	NA
3390	VE30613	Miranda	Baruta	Nuestra Señora del Rosario de Baruta	*P. geniculatus*	2013	Male	Positive	No
3391	VE14713	Miranda	Baruta	Nuestra Señora del Rosario de Baruta	*P. geniculatus*	2013	Male	Positive	No
3392	VE37913	Miranda	Baruta	Nuestra Señora del Rosario de Baruta	*P. geniculatus*	2013	Male	Positive	No
3393	VE33013	Miranda	Baruta	Nuestra Señora del Rosario de Baruta	*P. geniculatus*	2013	Male	Negative	No
3394	VE15713	Miranda	Baruta	Nuestra Señora del Rosario de Baruta	*P. geniculatus*	2013	Male	NA	No
3395	VE5813	Miranda	Baruta	Nuestra Señora del Rosario de Baruta	*P. geniculatus*	2013	Male	Positive	Yes
3396	VE38313B	Miranda	Chacao	Chacao	*P. geniculatus*	2013	Female	Positive	Yes
3397	VE22213	Miranda	Chacao	Chacao	*P. geniculatus*	2013	Male	Positive	No
3398	VE28013	Miranda	Chacao	Chacao	*P. geniculatus*	2013	Nymph IV	Positive	No
3399	VE22013	Miranda	El Hatillo	Santa Rosalía de Palermo	*P. geniculatus*	2013	Female	Negative	No
3400	VE8813	Miranda	El Hatillo	Santa Rosalía de Palermo	*P. geniculatus*	2013	Female	Positive	Yes

3401	VE10913	Miranda	El Hatillo	Santa Rosalía de Palermo	*P. geniculatus*	2013	Female	Positive	Yes
3402	VE27113	Miranda	El Hatillo	Santa Rosalía de Palermo	*P. geniculatus*	2013	Female	Negative	Yes
3403	VE31613	Miranda	El Hatillo	Santa Rosalía de Palermo	*P. geniculatus*	2013	Female	Positive	Yes
3404	VE13013	Miranda	El Hatillo	Santa Rosalía de Palermo	*P. geniculatus*	2013	Female	Positive	Yes
3405	VE1513	Miranda	El Hatillo	Santa Rosalía de Palermo	*P. geniculatus*	2013	Male	Negative	No
3406	VE38913	Miranda	El Hatillo	Santa Rosalía de Palermo	*P. geniculatus*	2013	Male	Positive	No
3407	VE14613	Miranda	El Hatillo	Santa Rosalía de Palermo	*P. geniculatus*	2013	Male	Positive	No
3408	VE27613	Miranda	El Hatillo	Santa Rosalía de Palermo	*P. geniculatus*	2013	Male	Positive	No
3409	VE35613	Miranda	El Hatillo	Santa Rosalía de Palermo	*P. geniculatus*	2013	Male	Positive	Yes
3410	VE26613	Miranda	El Hatillo	Santa Rosalía de Palermo	*P. geniculatus*	2013	Male	Positive	No
3411	VE11313	Miranda	Sucre	Caucagüita	*P. geniculatus*	2013	Female	Positive	Yes
3412	VE25713	Miranda	Sucre	Caucagüita	*P. geniculatus*	2013	Female	Positive	Yes
3413	VE26113	Miranda	Sucre	Caucagüita	*P. geniculatus*	2013	Female	Positive	Yes
3414	VE29513	Miranda	Sucre	Caucagüita	*P. geniculatus*	2013	Female	NA	Yes
3415	VE2513	Miranda	Sucre	Caucagüita	*P. geniculatus*	2013	Male	Negative	No
3416	VE16613	Miranda	Baruta	El Cafetal	*P. geniculatus*	2013	Female	Positive	Yes
3417	VE12613	Miranda	Sucre	Filas de mariche	*P. geniculatus*	2013	Female	Positive	Yes
3418	VE31013	Miranda	Sucre	Filas de mariche	*P. geniculatus*	2013	Female	NA	NA
3419	VE31213	Miranda	Sucre	Filas de mariche	*P. geniculatus*	2013	Female	Negative	Yes
3420	VE38113	Miranda	Sucre	Filas de mariche	*P. geniculatus*	2013	Female	NA	No
3421	VE39113	Miranda	Sucre	Filas de mariche	*P. geniculatus*	2013	Female	NA	No
3422	VE11713	Miranda	Sucre	Filas de mariche	*P. geniculatus*	2013	Female	Positive	Yes
3423	VE15113A	Miranda	Sucre	Filas de mariche	*P. geniculatus*	2013	Female	NA	NA
3424	VE15113B	Miranda	Sucre	Filas de mariche	*P. geniculatus*	2013	Female	NA	NA
3425	VE16313	Miranda	Sucre	Filas de mariche	*P. geniculatus*	2013	Female	Positive	Yes
3426	VE16413A	Miranda	Sucre	Filas de mariche	*P. geniculatus*	2013	Female	Positive	Yes
3427	VE0613	Miranda	Sucre	Filas de mariche	*P. geniculatus*	2013	Male	Positive	Yes
3428	VE33213	Miranda	Sucre	Filas de mariche	*P. geniculatus*	2013	Male	NA	No
3429	VE16413B	Miranda	Sucre	Filas de mariche	*P. geniculatus*	2013	Male	NA	Yes
3430	VE16213	Miranda	Sucre	Filas de mariche	*P. geniculatus*	2013	Male	Positive	Yes
3431	VE29613	Miranda	Sucre	Filas de mariche	*P. geniculatus*	2013	Nymph IV	NA	Yes
3432	VE36713	Miranda	Sucre	Filas de mariche	*P. geniculatus*	2013	Nymph IV	Negative	Yes
3433	VE38013	Miranda	Sucre	Filas de mariche	*P. geniculatus*	2013	Nymph IV	Positive	Yes
3434	VE37713	Miranda	Sucre	La Dolorita	*P. geniculatus*	2013	Female	Positive	Yes
3435	VE37813	Miranda	Sucre	La Dolorita	*P. geniculatus*	2013	Female	NA	NA
3436	VE14913	Miranda	Sucre	La Dolorita	*P. geniculatus*	2013	Female	NA	Yes
3437	VE18913	Miranda	Sucre	La Dolorita	*P. geniculatus*	2013	Female	Positive	No
3438	VE21013A	Miranda	Sucre	La Dolorita	*P. geniculatus*	2013	Female	Negative	Yes
3439	VE21013B	Miranda	Sucre	La Dolorita	*P. geniculatus*	2013	Female	Positive	Yes
3440	VE37213	Miranda	Sucre	La Dolorita	*P. geniculatus*	2013	Female	NA	Yes
3441	VE36513	Miranda	Sucre	La Dolorita	*P. geniculatus*	2013	Female	Positive	Yes
3442	VE20913	Miranda	Sucre	La Dolorita	*P. geniculatus*	2013	Male	Positive	Yes

3443	VE23813	Miranda	Sucre	La Dolorita	*P. geniculatus*	2013	Male	NA	No
3444	VE24313	Miranda	Sucre	La Dolorita	*P. geniculatus*	2013	Male	Positive	Yes
3445	VE31513	Miranda	Sucre	La Dolorita	*P. geniculatus*	2013	Male	Positive	Yes
3446	VE37613	Miranda	Sucre	La Dolorita	*P. geniculatus*	2013	Male	NA	No
3447	VE33813	Miranda	Sucre	La Dolorita	*P. geniculatus*	2013	Male	Positive	No
3448	VE17813	Miranda	Sucre	Leoncio Martínez	*P. geniculatus*	2013	Female	Positive	No
3449	VE0213	Miranda	Sucre	Leoncio Martínez	*P. geniculatus*	2013	Female	Positive	Yes
3450	VE5913	Miranda	Sucre	Leoncio Martínez	*P. geniculatus*	2013	Female	NA	NA
3451	VE39513	Miranda	Sucre	Leoncio Martínez	*P. geniculatus*	2013	Female	Positive	No
3452	VE14013	Miranda	Sucre	Leoncio Martínez	*P. geniculatus*	2013	Female	Positive	Yes
3453	VE28413	Miranda	Sucre	Leoncio Martínez	*P. geniculatus*	2013	Female	Negative	Yes
3454	VE9813	Miranda	Sucre	Leoncio Martínez	*P. geniculatus*	2013	Female	Negative	No
3455	VE21313	Miranda	Sucre	Leoncio Martínez	*P. geniculatus*	2013	Female	Positive	Yes
3456	VE21413	Miranda	Sucre	Leoncio Martínez	*P. geniculatus*	2013	Female	Positive	No
3457	VE26713	Miranda	Sucre	Leoncio Martínez	*P. geniculatus*	2013	Female	Positive	No
3458	VE21113	Miranda	Sucre	Leoncio Martínez	*P. geniculatus*	2013	Female	Positive	No
3459	VE36113	Miranda	Sucre	Leoncio Martínez	*P. geniculatus*	2013	Female	NA	No
3460	VE27213	Miranda	Sucre	Leoncio Martínez	*P. geniculatus*	2013	Male	NA	No
3461	VE20513	Miranda	Sucre	Leoncio Martínez	*P. geniculatus*	2013	Male	Positive	No
3462	VE1013	Miranda	Sucre	Leoncio Martínez	*P. geniculatus*	2013	Male	Positive	No
3463	VE25613	Miranda	Sucre	Leoncio Martínez	*P. geniculatus*	2013	Male	Positive	No
3464	VE8713	Miranda	Sucre	Leoncio Martínez	*P. geniculatus*	2013	Male	Positive	No
3465	VE28113	Miranda	Sucre	Leoncio Martínez	*P. geniculatus*	2013	Male	Positive	No
3466	VE36213	Miranda	Sucre	Leoncio Martínez	*P. geniculatus*	2013	Male	Positive	Yes
3467	VE23313	Miranda	Sucre	Leoncio Martínez	*P. geniculatus*	2013	Male	Positive	Yes
3468	VE20113	Miranda	Sucre	Petare	*P. geniculatus*	2013	Female	Positive	Yes
3469	VE25113	Miranda	Sucre	Petare	*P. geniculatus*	2013	Female	NA	No
3470	VE14413	Miranda	Sucre	Petare	*P. geniculatus*	2013	Female	NA	NA
3471	VE4513	Miranda	Sucre	Petare	*P. geniculatus*	2013	Female	NA	No
3472	VE38513	Miranda	Sucre	Petare	*P. geniculatus*	2013	Female	Negative	Yes
3473	VE38613	Miranda	Sucre	Petare	*P. geniculatus*	2013	Female	Positive	Yes
3474	VE39013	Miranda	Sucre	Petare	*P. geniculatus*	2013	Female	Positive	No
3475	VE14313	Miranda	Sucre	Petare	*P. geniculatus*	2013	Female	Positive	Yes
3476	VE29213	Miranda	Sucre	Petare	*P. geniculatus*	2013	Female	Positive	Yes
3477	VE4013	Miranda	Sucre	Petare	*P. geniculatus*	2013	Female	NA	No
3478	VE35813	Miranda	Sucre	Petare	*P. geniculatus*	2013	Female	Positive	Yes
3479	VE6313	Miranda	Sucre	Petare	*P. geniculatus*	2013	Female	NA	Yes
3480	VE29413	Miranda	Sucre	Petare	*P. geniculatus*	2013	Female	Positive	Yes
3481	VE34113	Miranda	Sucre	Petare	*P. geniculatus*	2013	Female	Positive	No
3482	VE23713	Miranda	Sucre	Petare	*P. geniculatus*	2013	Female	Negative	Yes
3483	VE1613A	Miranda	Sucre	Petare	*P. geniculatus*	2013	Female	NA	NA
3484	VE1613B	Miranda	Sucre	Petare	*P. geniculatus*	2013	Female	Positive	No

3485	VE4313	Miranda	Sucre	Petare	*P. geniculatus*	2013	Female	Positive	Yes
3486	VE34913	Miranda	Sucre	Petare	*P. geniculatus*	2013	Female	Positive	Yes
3487	VE23513	Miranda	Sucre	Petare	*P. geniculatus*	2013	Female	NA	Yes
3488	VE34713	Miranda	Sucre	Petare	*P. geniculatus*	2013	Female	NA	NA
3489	VE2913	Miranda	Sucre	Petare	*P. geniculatus*	2013	Female	Negative	No
3490	VE13913A	Miranda	Sucre	Petare	*P. geniculatus*	2013	Female	Positive	Yes
3491	VE15313	Miranda	Sucre	Petare	*P. geniculatus*	2013	Female	Positive	Yes
3492	VE6513	Miranda	Sucre	Petare	*P. geniculatus*	2013	Female	Negative	No
3493	VE24413	Miranda	Sucre	Petare	*P. geniculatus*	2013	Female	Positive	Yes
3494	VE14513	Miranda	Sucre	Petare	*P. geniculatus*	2013	Female	Positive	Yes
3495	VE17213	Miranda	Sucre	Petare	*P. geniculatus*	2013	Female	Negative	Yes
3496	VE25413	Miranda	Sucre	Petare	*P. geniculatus*	2013	Female	Positive	Yes
3497	VE38413	Miranda	Sucre	Petare	*P. geniculatus*	2013	Female	Positive	Yes
3498	VE3013	Miranda	Sucre	Petare	*P. geniculatus*	2013	Female	NA	No
3499	VE13113	Miranda	Sucre	Petare	*P. geniculatus*	2013	Female	Positive	Yes
3500	VE27513	Miranda	Sucre	Petare	*P. geniculatus*	2013	Female	Positive	Yes
3501	VE0513	Miranda	Sucre	Petare	*P. geniculatus*	2013	Female	Positive	Yes
3502	VE5513	Miranda	Sucre	Petare	*P. geniculatus*	2013	Female	Positive	Yes
3503	VE9013	Miranda	Sucre	Petare	*P. geniculatus*	2013	Female	Positive	Yes
3504	VE9113	Miranda	Sucre	Petare	*P. geniculatus*	2013	Female	Positive	Yes
3505	VE13213	Miranda	Sucre	Petare	*P. geniculatus*	2013	Female	NA	Yes
3506	VE15013A	Miranda	Sucre	Petare	*P. geniculatus*	2013	Female	Positive	Yes
3507	VE15013B	Miranda	Sucre	Petare	*P. geniculatus*	2013	Female	NA	NA
3508	VE18713	Miranda	Sucre	Petare	*P. geniculatus*	2013	Female	NA	Yes
3509	VE21613B	Miranda	Sucre	Petare	*P. geniculatus*	2013	Female	Positive	Yes
3510	VE30413	Miranda	Sucre	Petare	*P. geniculatus*	2013	Female	Positive	Yes
3511	VE31713	Miranda	Sucre	Petare	*P. geniculatus*	2013	Female	Positive	Yes
3512	VE33113	Miranda	Sucre	Petare	*P. geniculatus*	2013	Female	Positive	Yes
3513	VE36313	Miranda	Sucre	Petare	*P. geniculatus*	2013	Female	Positive	Yes
3514	VE3213	Miranda	Sucre	Petare	*P. geniculatus*	2013	Female	Positive	Yes
3515	VE3413	Miranda	Sucre	Petare	*P. geniculatus*	2013	Female	Positive	Yes
3516	VE34813	Miranda	Sucre	Petare	*P. geniculatus*	2013	Female	Negative	Yes
3517	VE13713A	Miranda	Sucre	Petare	*P. geniculatus*	2013	Female	Positive	Yes
3518	VE13713B	Miranda	Sucre	Petare	*P. geniculatus*	2013	Female	Positive	No
3519	VE28813	Miranda	Sucre	Petare	*P. geniculatus*	2013	Female	Positive	Yes
3520	VE1213A	Miranda	Sucre	Petare	*P. geniculatus*	2013	Female	NA	Yes
3521	VE1213B	Miranda	Sucre	Petare	*P. geniculatus*	2013	Female	NA	No
3522	VE12813	Miranda	Sucre	Petare	*P. geniculatus*	2013	Female	Positive	No
3523	VE19313	Miranda	Sucre	Petare	*P. geniculatus*	2013	Female	Negative	No
3524	VE32013	Miranda	Sucre	Petare	*P. geniculatus*	2013	Female	NA	Yes
3525	VE7613	Miranda	Sucre	Petare	*P. geniculatus*	2013	Female	Positive	Yes
3526	VE20313	Miranda	Sucre	Petare	*P. geniculatus*	2013	Female	Positive	No

3527	VE8513	Miranda	Sucre	Petare	*P. geniculatus*	2013	Female	Positive	Yes
3528	VE15613	Miranda	Sucre	Petare	*P. geniculatus*	2013	Female	NA	No
3529	VE8613	Miranda	Sucre	Petare	*P. geniculatus*	2013	Female	Positive	Yes
3530	VE10713	Miranda	Sucre	Petare	*P. geniculatus*	2013	Male	Positive	Yes
3531	VE20413	Miranda	Sucre	Petare	*P. geniculatus*	2013	Male	Positive	No
3532	VE22313	Miranda	Sucre	Petare	*P. geniculatus*	2013	Male	Positive	No
3533	VE13913B	Miranda	Sucre	Petare	*P. geniculatus*	2013	Male	Positive	Yes
3534	VE28613	Miranda	Sucre	Petare	*P. geniculatus*	2013	Male	Positive	No
3535	VE15413	Miranda	Sucre	Petare	*P. geniculatus*	2013	Male	Positive	Yes
3536	VE32913	Miranda	Sucre	Petare	*P. geniculatus*	2013	Male	Positive	No
3537	VE6713	Miranda	Sucre	Petare	*P. geniculatus*	2013	Male	Positive	Yes
3538	VE30213	Miranda	Sucre	Petare	*P. geniculatus*	2013	Male	Positive	No
3539	VE10013	Miranda	Sucre	Petare	*P. geniculatus*	2013	Male	NA	Yes
3540	VE16813	Miranda	Sucre	Petare	*P. geniculatus*	2013	Male	NA	NA
3541	VE23613	Miranda	Sucre	Petare	*P. geniculatus*	2013	Male	Positive	Yes
3542	VE34213	Miranda	Sucre	Petare	*P. geniculatus*	2013	Male	NA	NA
3543	VE12713	Miranda	Sucre	Petare	*P. geniculatus*	2013	Male	Positive	No
3544	VE35913A	Miranda	Sucre	Petare	*P. geniculatus*	2013	Male	Positive	No
3545	VE35913B	Miranda	Sucre	Petare	*P. geniculatus*	2013	Male	Negative	No
3546	VE6113	Miranda	Sucre	Petare	*P. geniculatus*	2013	Nymph III	NA	Yes
3547	VE28213	Miranda	Sucre	Petare	*P. geniculatus*	2013	Nymph IV	Positive	Yes
3548	VE6613	Miranda	Sucre	Petare	*P. geniculatus*	2013	Nymph IV	Positive	No
3549	VE29913	Miranda	Sucre	Petare	*P. geniculatus*	2013	Nymph IV	Negative	Yes
3550	VE35213	Miranda	Sucre	Petare	*P. geniculatus*	2013	Nymph IV	Positive	Yes
3551	VE1813	Miranda	Sucre	Petare	*P. geniculatus*	2013	Nymph IV	Positive	Yes
