# Supplementary material for: Panstrongylus geniculatus and four other species of triatomine bug involved in the Trypanosoma cruzi enzootic cycle: high risk factors for Chagas’ disease transmission in the Metropolitan District of Caracas, Venezuela
Source: Parasit Vectors. 2014 Dec 23;7:602. doi: 10.1186/s13071-014-0602-7 (PMC4307744; doi:10.1186/s13071-014-0602-7)
Supplement: Additional file 2: Table S2. — Population and area of the 32 parishes that constitute the Metropolitan District of Caracas. [file 13071_2014_602_MOESM2_ESM.pdf]

Table S2 Population and area of the 32 Parishes that constitute the Metropolitan District of Caracas

Municipality	Number *	Parish	Population	Area Km2
**Libertador**	1	23 de enero	77344	2,3
Libertador	2	Altagracia	47922	4,47
Libertador	3	Antímano	131963	20,9
Libertador	4	Candelaria	66486	1,23
Libertador	5	Caricuao	138659	23,8
Libertador	6	Catedral	12777	0,8
Libertador	7	Coche	59889	13
Libertador	8	El Junquito	50470	52,5
Libertador	9	El Paraiso	109622	10,8
Libertador	10	El Recreo	112809	18,1
Libertador	11	El Valle	142893	16,6
Libertador	12	La Pastora	80397	4,5
Libertador	13	La Vega	123863	12,6
Libertador	14	Macarao	47851	10,3
Libertador	15	San Agustin	38476	1,6
Libertador	16	San Bernardino	27353	12,2
Libertador	17	San José	39604	2,6
Libertador	18	San Juan	106507	3,3
Libertador	19	San Pedro	58254	6,7
Libertador	20	Santa Rosalía	101103	6,7
Libertador	21	Santa Teresa	23715	0,7
Libertador	22	Sucre	345944	59,3
**Baruta**	23	Nuestra Señora del Rosario de Baruta	159142	86
Baruta	24	El Cafetal	41543	9
Baruta	25	Las Minas de Baruta	40070	4
**Chacao**	26	Chacao	61213	13
**El Hatillo**	27	Santa Rosalía de Palermo	58156	114
**Sucre**	28	Filas de Mariche	32303	36
Sucre	29	La Dolorita	65159	11
Sucre	30	Leoncio Martínez	63260	23
Sucre	31	Caucagüita	67013	54
Sucre	32	Petare	372616	40

* Numbers correspond to Parishes indicated in Figure 1 and Table 2
