# Supplementary material for: Panstrongylus geniculatus and four other species of triatomine bug involved in the Trypanosoma cruzi enzootic cycle: high risk factors for Chagas’ disease transmission in the Metropolitan District of Caracas, Venezuela
Source: Parasit Vectors. 2014 Dec 23;7:602. doi: 10.1186/s13071-014-0602-7 (PMC4307744; doi:10.1186/s13071-014-0602-7)
Supplement: Additional file 3: Table S3. — Number of triatomine bugs collected per month in the Metropolitan District of Caracas, 2007 to 2013. [file 13071_2014_602_MOESM3_ESM.pdf]

Table S3 Number of triatomine bugs monthly collected in the Metropolitan District of Caracas. 2007-2013

Month	2007	2008	2009	2010	2011	2012	2013	Total
January	6	31	49	16	30	25	34	191
February	2	5	19	51	53	25	31	186
March	12	13	43	80	52	43	40	283
April	14	38	127	86	83	58	74	480
May	16	93	158	460	146	67	70	1010
June	8	76	121	340	32	56	27	660
July	3	52	49	78	12	32	13	239
August	2	7	12	37	2	5	16	81
September	1	26	11	25	3	11	3	80
October	0	19	21	51	17	19	21	148
November	3	29	27	33	11	14	12	129
December	11	11	11	14	8	0	9	64
**Total**	78	400	648	1271	449	355	350	3551
