# Supplementary material for: Panstrongylus geniculatus and four other species of triatomine bug involved in the Trypanosoma cruzi enzootic cycle: high risk factors for Chagas’ disease transmission in the Metropolitan District of Caracas, Venezuela
Source: Parasit Vectors. 2014 Dec 23;7:602. doi: 10.1186/s13071-014-0602-7 (PMC4307744; doi:10.1186/s13071-014-0602-7)
Supplement: Additional file 4: Table S4. — Genotypes of T. cruzi isolates obtained from the 5 species of triatomines found in Caracas. [file 13071_2014_602_MOESM4_ESM.pdf]

Table S4 Genotype of *T. cruzi* isolates obtained from 5 different species of triatomines found in Caracas

Parish	Species	strains	TcI	TcIII
Altagracia	*P.geniculatus*	VE1507 VE2106 VE29809 VE5107A VE5107B VE6406A VE6406B VQC106 VQC206 VQC406 VQC506 VQC606VE1507 VE2106 VE29809 VE5107A VE5107B VE6406A VE6406B VQC106 VQC206 VQC406 VQC506 VQC606	12	
Antímano	*P.geniculatus*	VE32008 VE34010 VE3603 VE7303 VE25112A	5	
Antímano	*R. prolixus*	VE45909	1	
Caricuao	*P.geniculatus*	VE11708	1	
El Junquito	*P.geniculatus*	PGN17 PGN23 VE0402 VE10708 VE12708 VE13209B VE2604 VE27009 VE29309 VE29509 VE3204 VE3504 VE37909 VE4206 VE4207 VE44509 VE48309 VE58609 VE8307	19	
El Paraiso	*P.geniculatus*	VE1002A VE1002B	2	
El Recreo	*P.geniculatus*	VE12508 VE2603 VE5210 VE8607E	4	
El Valle	*P.geniculatus*	VE1007	1	
La Pastora	*P.geniculatus*	VE0708 VE1009 VE10309 VE12008 VE14809 VE1504A VE1504B VE1706 VE2306 VE2406A VE2904 VE2905 VE3307B VE3307C VE3307E VE34409 VE38208 VE38508 VE49709 VE5103 VE5508 VE55909 VE5908 VE5909B VE6603 VE8308 VQ306 VQC406	28	
La Vega	*P.geniculatus*	VE1103A VE1103B VE1107	3	
Macarao	*P.geniculatus*	VE10008 VE9608 VE13408a VE13408b	4	
San Bernardino	*P.geniculatus*	PGN16 VE1004A VE1004B VE1004C VE15208	5	
San Jose	*P.geniculatus*	VE41108 VE26009 SJ1097 VE0706 VE5208	5	
San Juan	*P.geniculatus*	VE1502 VE7208	2	
San Pedro	*P.geniculatus*	PGCHG PgHC07 VE7008 VE7608	4	
Santa teresa	*P.geniculatus*	VE5304	1	
Sucre	*P.geniculatus*	VE1003 VE10408 VE1203 VE12108 VE14108 VE14308 VE14308B VE17208 VE18708 VE19709 VE2008 VE2102 VE21109 VE22009A VE24409 VE24409D VE2803 VE28309 VE3708 VE37209 VE37209B VE4203 VE45609 VE50509 VE5109 VE5204 VE6008 VE6703 VE6706 VE7508A VE7508B VE8607 VE8607A VE8607C VE8607D VE9108 VE9408 VE9508B VE9908 VEclara	40	
El Cafetal	*P.geniculatus*	VE0506 VE2505 VE37008	3	
Las Minas de Baruta	*P.geniculatus*	PGN14 PGN900 VE0806 VE24508 VE2606 VE32508 VE38509	7	
Ns. Sra. R. Baruta	*P.geniculatus*	VE0203 VE10808A VE10808B VE1303 VE15008 VE23008 VE31109B VE3304 VE35208 VE3703 VE51908 VE5807	12	
Ns. Sra. R. Baruta	*T. maculata*	VE1312	1	
Ns. Sra. R. Baruta	*T. nigromaculata*	VE31408	1	
Chacao	*P.geniculatus*	VE18808	1	
Sta. R. P. El Hatillo	*P.geniculatus*	PGN11 VE1308 VE31208 VE33509 VE5804 VE61810	6	
Caucagüita	*P.geniculatus*	VE0208 VE021508 VE1206 VE16208 VE3404 VE5407 **<u>VE3403</u>**	6	**<u>1</u>**
Caucagüita	*T. nigromaculata*	VE14109B	1	
Fila de Mariche	*E. mucronatus*	EM2001 EM400	2	
Fila de Mariche	*P.geniculatus*	FILAS DE M PGN2201 VE0904 VE12808 VE1404 VE14208 VE2204 VE2307 VE2507A VE2507B VE2602 VE2707 VE29009 VE2907 VE3107 VE3207 VE34108 VE3807 VE4808 VE6010 VE9808	21	
Fila de Mariche	*T. maculata*	VE30611	1	
La Dolorita	*P.geniculatus*	VE16108 VE1806A VE23708 VE24009 VE3206 VE3605C	6	
Leoncio Martínez	*P.geniculatus*	PGN18 VE0802 VE13708 VE15908 VE16508 VE18508 VE4204 VE4804 VE55009 VE6508	10	
Petare	*P.geniculatus*	PGN1701 pgn2 PGN200 PGN700 VE0204 VE02608 VE0704 VE0902 VE1102 VE11808 VE13608A VE13608C VE15408A VE15408B VE15408D VE15408E VE16008A VE16008B VE1604 VE1606 VE1608A VE1608B VE16408A VE16408B VE16408C VE16408D VE16408E VE16608 VE17908 VE1804 VE18608 VE1904 VE2005 VE2103 VE2208 VE2305A VE2504 VE2706B VE2808 VE3303 VE3306 VE33409 VE38408 VE43209A VE4604 VE4708 VE4807B VE4807C VE5007 VE50108 VE52609 VE5608 VE6303A VE6303C VE6303D VE6303E VE65309 VE6903 VE7908 VE7908* VEEG05	61	
		**<u>In bold and underlined: specimen infected with TcIII</u>**	276	1
